# Remembering Don Bryant (1950–2024)

**DOI:** 10.1007/s11120-025-01158-1

**Published:** 2025-07-01

**Authors:** Christopher J. Gisriel, Wendy M. Schluchter, Fei Gan, John H. Golbeck, Ming-Yang Ho, Gaozhong Shen, Nathan T. Soulier, Vera Thiel, David M. Ward, Jindong Zhao, Shuyi Zhang

**Affiliations:** 1 Department of Biochemistry, University of Wisconsin-Madison, Madison, WI USA; 2 Department of Biological Sciences, University of New Orleans, New Orleans, LA USA; 3 State Key Laboratory of Metabolism and Regulation in Complex Organisms, Hubei Key Laboratory of Cell Homeostasis, College of Life Science, TaiKang Center for Life and Medical Sciences, Wuhan University, Wuhan, China; 4 Department of Biochemistry and Molecular Biology, The Pennsylvania State University, University Park, PA USA; 5 Department of Life Science, National Taiwan University, Taipei, Taiwan; 6 Department of Molecular Biology, University of California San Diego, La Jolla, CA USA; 7 Leibniz Institute DSMZ - German Collection of Microorganisms and Cell Cultures, Braunschweig, Germany; 8 Department of Land Resources and Environmental Sciences, Montana State University, Bozeman, MT USA; 9 State Key Laboratory of Protein and Plant Genetic Engineering, School of Life Sciences, Peking University, Beijing, 100871 China; 10 School of Pharmaceutical Sciences, Tsinghua University, Beijing, China

**Keywords:** Photosynthesis, Photosystems, Phycobiliproteins, Microbial ecology, Photosynthetic diversity


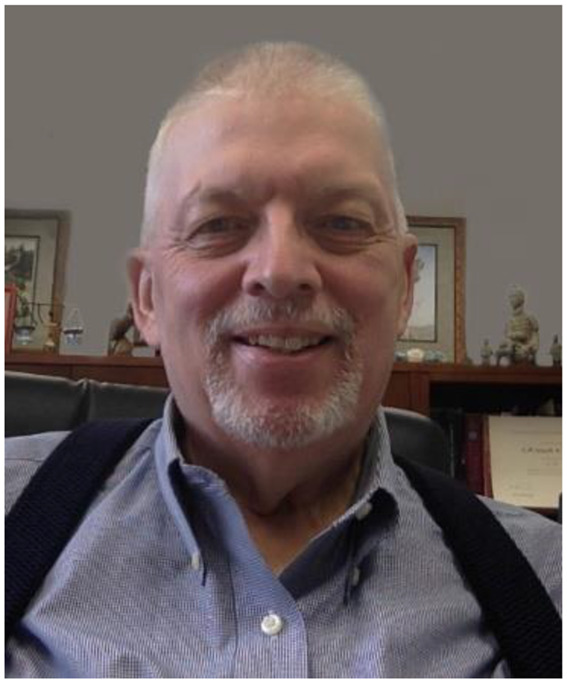


Donald A. Bryant, affectionately known as Don to his colleagues and friends, passed away in August, 2024 after a lifetime devoted to advancing the field of photosynthesis research. During his remarkable and influential career, Don made groundbreaking contributions, including elucidating key mechanisms of bacterial photosynthesis and the physiology of chlorophototrophic microbes. His pioneering work provided deep insights into microbial light-harvesting systems and their ecological significance. Here, colleagues and friends honor Don’s extraordinary scientific legacy, reflecting on his profound impact on the field, his mentorship, and the inspiration he brought to generations of researchers worldwide.

## Don Bryant’s scientific career

### Early years (by Clayton Stoess, jr., Bill Larsen, and Wendy Schluchter)

**Fig. 1:**
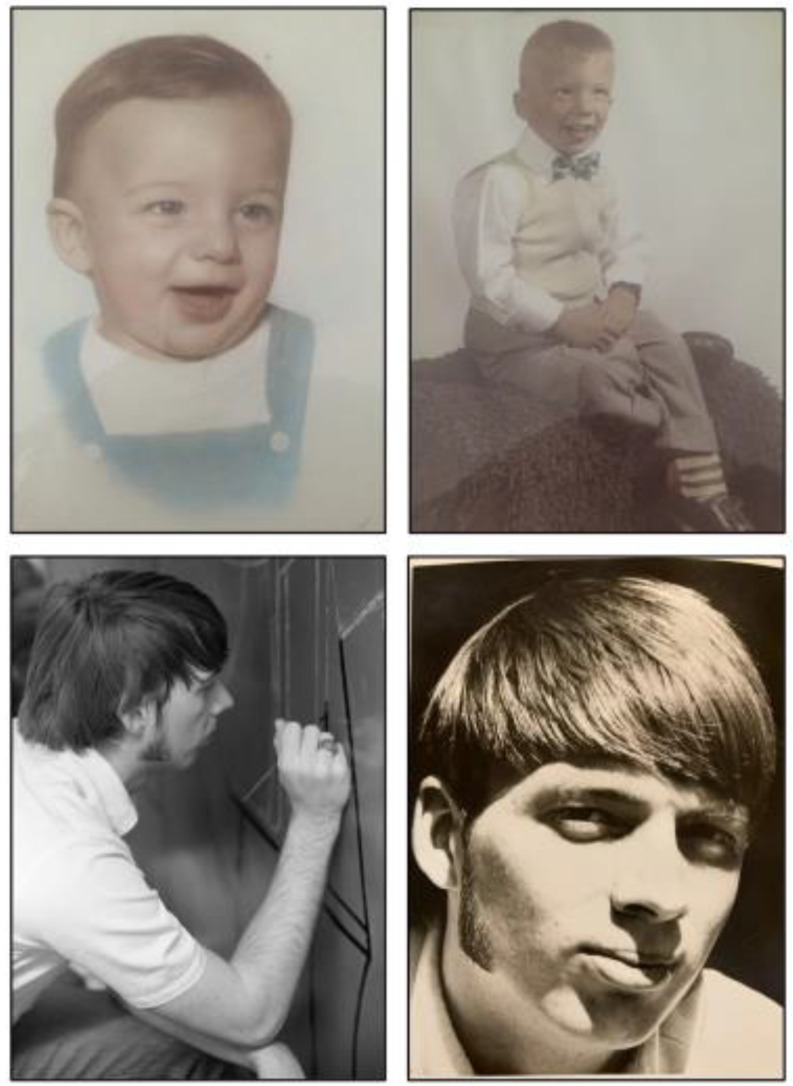
Photos of Don Bryant from the years 1950 (top left), 1953 (top right), and 1972 (both bottom panels)

Don grew up on a Kentucky dairy farm that straddled the Oldham County/Henry County line. A dairy farm is a 24/7 all-consuming occupation that employs whole families starting at a young age. Don decided very early that he was not going to stay on the farm. Don attended Oldham County High School, the first and only high school in this region. Don’s best friend in high school was Clayton Stoess, Jr. Clayton met his wife, Claudia, who was a grade school friend of Don’s, in high school. Don was a groomsman in their wedding in 1971. Some early photos of Don are shown in Fig. 1.

Clayton and Don had many classes together through their years in high school. It was never a competition between them because both had an innate passion for learning, which resulted in Don being valedictorian and Clayton being salutatorian. Clayton was class president, and Don was president of the student government.

Mr. Benson was the science teacher that challenged and inspired Don. Don did not come across as a “science geek” in high school, but when Don talked about his interest in science, one could tell that he had much more than a pedestrian interest in all parts of science. He was also very talented in math, and Mrs. McKechnie was his math teacher that challenged him.

**Fig. 2:**
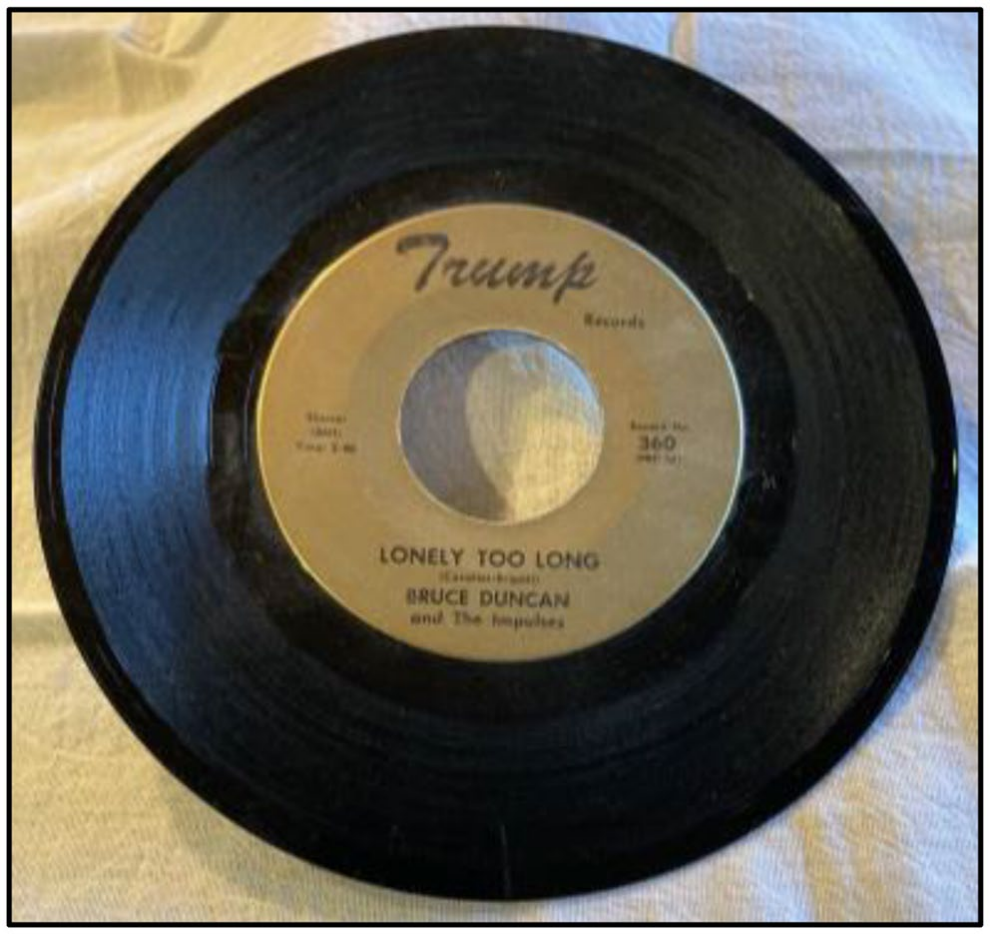
Picture of a 45 rpm record for Bruce Duncan and the Impulses (Audio S1)

Don and Clayton played in a rock band called “The Four Contrasts” in high school. Don played guitar, saxophone and sang, Clayton played keyboards and sang, Clayton’s cousin played bass and sang, and another friend played drums. Rumor has it that this band was actually pretty good. Over their junior and senior years, they spent many hours practicing and playing gigs, including fraternity parties. During their senior year, some of these band members, including Don, also played in another band called “Bruce Duncan and the Impulses”, and they made a record in 1967 (Fig. 2, Audio S1). Don’s mother, Wanda Bryant, worked hard to make sure that he had every opportunity with his education and his music.

For his undergraduate studies, Don attended the Massachusetts Institute of Technology, joining a mostly male freshman class of 1,000. Don experienced some culture shock at first, but he knew he had his work cut out for him. However, farm environment habits such as getting up early and getting your work done on time were well-ingrained, and he soon found out that he had nothing to fear.

Don’s hobbies in college were bridge, softball, tennis, and rock music (an early and lifelong fan of Bonnie Raitt). His best friends during this time were Bill L. (from IL), Dave B. (PA), Dennis B. (IL), George B. (KS), Mike D. (VT), and Ron M. (NM). Don was solely a chemistry major at first, but he began adding biology classes during his junior year. The landmark educational course for him was General Microbiology in which Professor Boris Magasanik regaled all with blow-by-blow descriptions of the many discoveries both large and small that led up to the current state of molecular biology knowledge (think Chargaff). The class textbook (Microbial World, 3rd edition, by R. Stanier) was one that would affect his scientific trajectory. By June of 1972, Don amassed sufficient credits to earn a bachelor’s degree in biology in addition to chemistry.

**Fig. 3:**
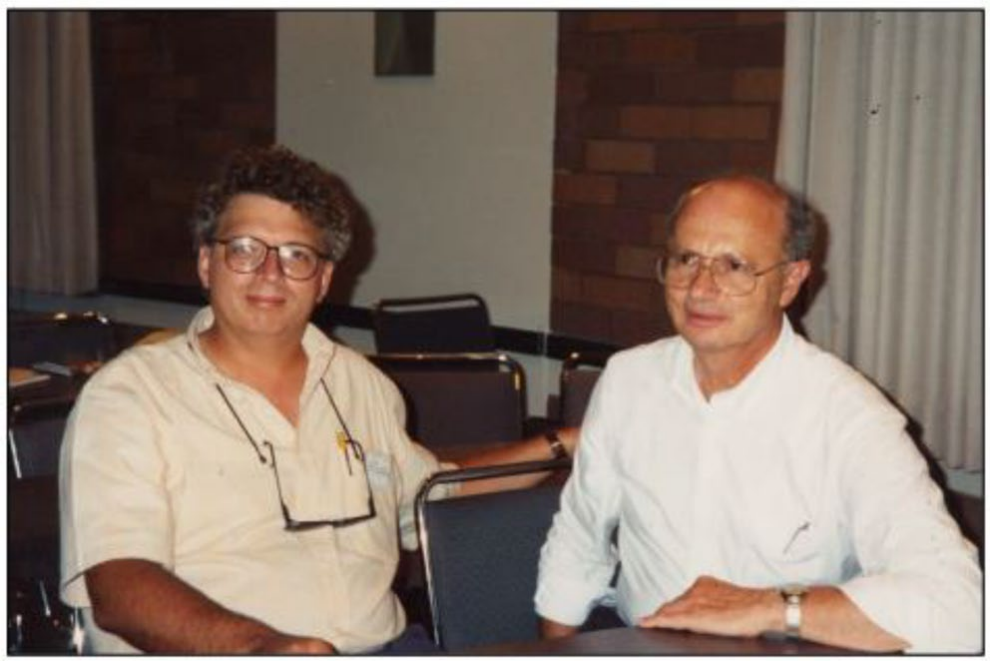
Don with Alex Glazer

Because he had been fascinated by bioenergetics, Don decided to attend the University of California-Los Angeles for graduate school, where he was a member of the first class of students in a new National Institutes of Health-sponsored training program in molecular biology. He was drawn to photosynthesis and started working with Alexander N. Glazer (Fig. 3) and Frederick Eiserling, thus beginning a lifelong research study of cyanobacteria. After completing his Ph.D. training where he studied the phycobiliproteins of cyanobacteria, he did a postdoctoral fellowship, working with Roger Stanier (author of the microbiology textbook) and Germaine Cohen-Bazire at the Institut Pasteur in Paris from 1977 to 1979, where he characterized the phycobiliproteins of many of the cyanobacteria in the Pasteur Culture Collection, which had been established by Dr. Stanier in the 1960s. Don joined the laboratory of Roderick K. Clayton at Cornell University from 1979 to 1981 for a second postdoctoral fellowship. In 1981, he started his tenure-track position in the Department of Biochemistry and Molecular Biology at The Pennsylvania State University, rising to full professor in 1991.

In 1992, Don was appointed the Ernest C. Pollard Professor of Biotechnology. He also held adjunct and research appointments at Montana State University 2009–2020, studying microbial mat ecology in Yellowstone thermal features, and was a visiting professor at the Singapore Center on Environmental Life Sciences Engineering at Nanyang Technological University, 2013–2018. He retired from Penn State in 2022, but he remained an emeritus faculty member until his passing.

### Phycobilisomes and phycobiliproteins (by Jindong Zhao)

**Fig. 4:**
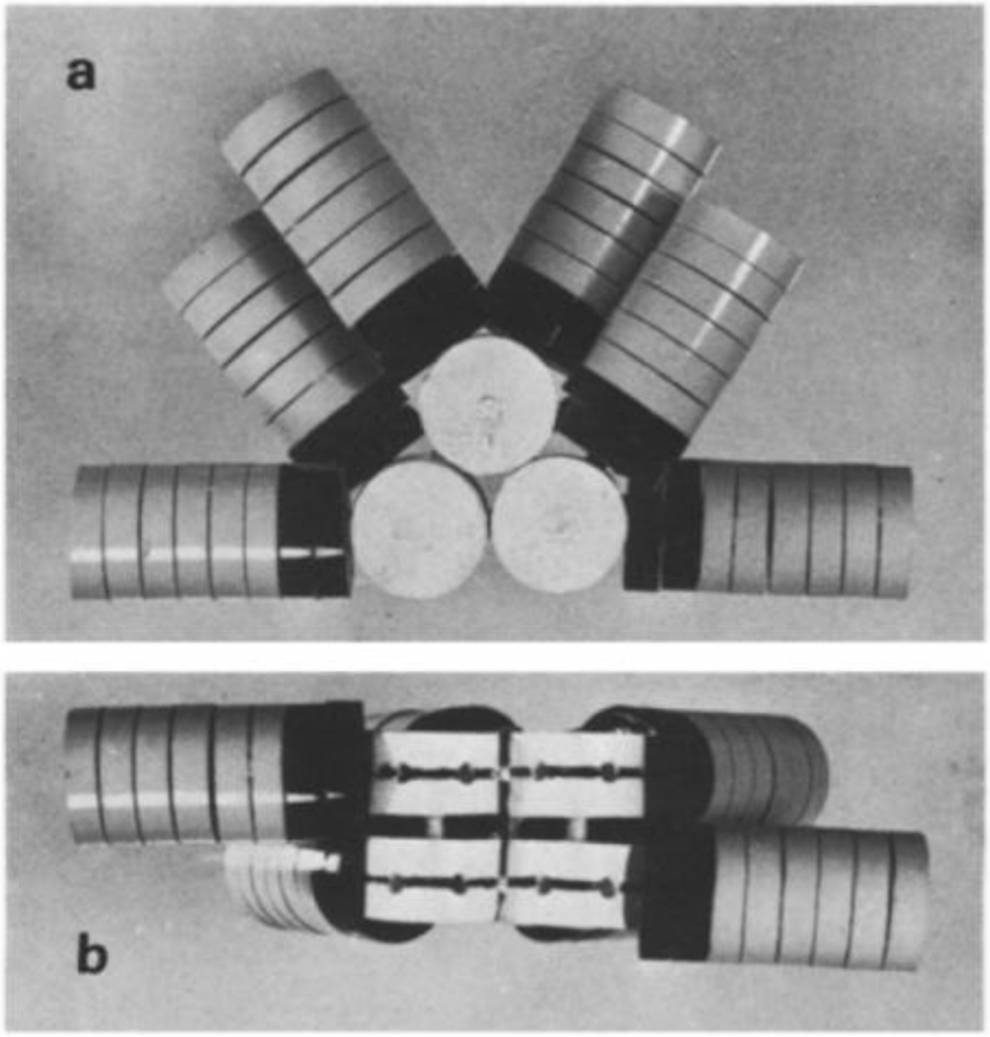
The phycobilisome model proposed by Bryant et al. (1979)

Phycobiliproteins are light-harvesting proteins found in cyanobacteria, often forming large complexes called phycobilisomes (PBS), whose purpose is to expand the number and wavelength range of photons that can be used for charge separation by the photosystems. Don was first introduced to phycobiliproteins as a graduate student in Alex Glazer’s group at UCLA, and he was still studying them after his retirement. Don’s first publication reported the discovery and characterization of allophycocyanin-B (ApcD), an important variant of allophycocyanin (AP) α-subunit (Glazer and Bryant 1975). Its extraordinary long wavelength emission let the authors predict correctly that ApcD was involved in energy transfer from PBS to chlorophyll (Chl) *a* of photosynthetic reaction centers (Glazer and Bryant 1975; Ley et al. 1977). But it took several decades before the roles of ApcD in energy transfer from PBS to Photosystem I (PSI) were revealed (Maxson et al. 1989; Zhou et al. 1990; Dong et al. 2009). Don continued working on ApcD in the later phase of his tenure and he and his colleagues revealed how ApcD was involved in the far-red light photoacclimation in some cyanobacteria (see section on FaRLiP below). During his graduate study, Don also discovered a novel phycobiliprotein: phycoerythrocyanin, which is present in some filamentous cyanobacteria and is more efficient in absorbing green light than phycocyanin due to the presence of a phycoviolobilin (Bryant et al. 1978; Bryant 1982). Another significant achievement in Don’s early career was the determination of PBS architecture that consists of a central core and peripheral rods attached to the core (Bryant et al. 1979) when he was a postdoctoral fellow in Paris. It has been recognized that the morphology of PBS is diversified to adapt to different light environments during evolution. Among the five types of PBS morphology that are known (Bryant and Gisriel 2024), Don was involved in the discovery of three types: the hemidiscoidal type (Bryant et al. 1979), the bundle-shaped type (Guglielmi et al. 1981) and the paddle-shaped (Jiang et al. 2023). Although these types of PBS appear to be different in shapes and compositions, the basic organization of their structures is conserved based on the cryo-EM structures of PBS. The PBS model (Fig. 4) proposed by Bryant et al. (Bryant et al. 1979) or its modified versions are used for description of PBS in all current textbooks.

After Don opened his lab in Penn State, he focused on PBS in the first several years and he was one of the earliest researchers to study light harvesting in photosynthesis with the tools of molecular biology. Combined with his masterful skills in microbiology and biochemistry, the progress in PBS studies in Don’s lab was rapid. By the late 1980’s, he had cloned and sequenced all the genes of PBS components from *Synechococcus* sp. PCC 7002 (hereafter *Synechococcus* 7002) and constructed mutants of these genes (Bryant 1991). This approach proved to be very fruitful in understanding phycobiliprotein biosynthesis, PBS assembly, and regulation of their gene expression. By the early 1990s, high level production of recombinant phycobiliproteins in *Escherichia coli* was achieved in Don’s lab and the in vitro reconstruction of biological systems (Zhao et al. 1990) became a powerful tool in PBS studies. The phenotypes of the mutants *cpcE* and *cpcF* led to the prediction that these two genes were involved in the attachment of bilins to apo-phycobiliproteins. After successful production of recombinant proteins of CpcE and CpcF in *E. coli*, Don and Alex Glazer were able to demonstrate for the first time that CpcE/CpcF were a lyase responsible for attaching a bilin to phycocyanin (Fairchild et al. 1992; Swanson et al. 1992; Zhou et al. 1992).

Since the discovery of PBS about six decades ago, many scientists contributed to the understanding of PBS biosynthesis, assembly, structure and energy transfer. Don was one of the few who stood out and was recognized internationally. The review article (Bryant and Gisriel 2024) published in Plant Cell in 2024 summarized his views on PBS and PBS research, and some of his important contributions to this field.

### Chlorosomes and BChl biosynthesis (by Christopher J. Gisriel)

Another of Don’s numerous contributions to science was his work on the chlorosome antenna structures of green sulfur bacteria. Green sulfur bacteria are anaerobic photoautotrophs that metabolize sulfur, and they contain chlorosomes, large bacteriochlorophyll (BChl)-containing structures, as antenna (Frigaard et al. 2003). Unlike other antenna systems where protein subunits provide a scaffold for chromophores, chlorosomes contain hundreds of thousands of chromophores that stack within a lipid monolayer envelope. Don’s interest was piqued by this topic after a 1988 conference where he witnessed a heated debate over whether proteins exist in chlorosomes. Today, we know that proteins are a minor component of chlorosomes and only one, CsmA, is directly associated with BChl molecules. Don began his investigations into green sulfur bacteria during a 1989–1990 sabbatical at ETH Zürich, and subsequently incorporated the research into his laboratory at Penn State. Consequently, Don’s ensuing studies advanced the understanding of chlorosomes by identifying key structural proteins, elucidating their genetic organization, and exploring their functional roles. His was the first lab to show that the green sulfur bacterium *Chlorobaculum tepidum* was naturally transformable. This, along with the availability of the complete genome, established *C. tepidum* as a model organism for studies on green sulfur bacteria.

Two studies from Don’s lab characterized several *csm* genes which encode envelope proteins exposed on the chlorosome surface, as demonstrated through protease susceptibility assays and antibody-based agglutination experiments. These proteins were shown to undergo post-translational processing and, along with specific lipids like monogalactosyl diglycerol, contribute to the structure of the chlorosome envelope. Transcriptional analyses revealed both monocistronic and dicistronic mRNA arrangements, indicating complex regulation of chlorosome gene expression (Chung and Bryant 1996a, b). Another study applied targeted mutagenesis to demonstrate that while *csmA* is essential for cell viability and chlorosome function, *csmC* is dispensable but influences chlorosome absorption properties, energy transfer efficiency, and growth rate. Importantly, this work established genetic tools such as homologous recombination for functional analysis in *Chlorobium* species (Chung et al. 1998). Building on these genetic insights, Don later published a comprehensive book chapter covering the unique adaptations of green sulfur bacteria, emphasizing the minimal protein content of chlorosomes, vast pigment density, and the role of self-assembled BChl aggregates (Frigaard and Bryant 2006).

Don’s lab also made great contributions to the pathway for BChl biosynthesis, especially in green sulfur bacteria (Frigaard et al. 2006; Chew and Bryant 2007a, b; Bryant et al. 2020a). In fact, the ability to manipulate BChl biosynthesis genetically in *C. tepidum* ultimately led to elucidation of how BChl organizes in chlorosomes (Ganapathy et al. 2009). Don’s group identified *bciA* as the gene encoding a C-8 vinyl reductase in *Chlorobium tepidum*, essential for converting divinyl Chl intermediates into their ethylated forms (Chew and Bryant 2007b) and identified *bchK* as the gene encoding the BChl *c* synthase (Frigaard N-U et al. 2002). Furthermore, his lab showed that BciD is a radical SAM enzyme capable of converting BChlide *c* and *d* into their corresponding formylated derivatives, BChlide *e* and *f*, through a proposed hydroxylation mechanism (Thweatt et al. 2017). Together, these studies revealed the enzymatic basis for multiple key modifications in BChl biosynthesis.

### Ecology of hot spring microbial mats (by Dave Ward and Vera Thiel)

Don also made incredible contributions to our understanding of microbial ecology in hot springs, especially in collaboration with the Ward Lab at Montana State University. This collaboration began in 2004, when he called Dave to request a letter of support for a proposal he was developing to sequence the genomes of several phototrophic bacteria. They had never met. Dave agreed to write a letter and encouraged Don to include a strain of *Roseiflexus* sp., which his group had recently cultivated from a Yellowstone hot spring microbial mat community that he, his students, and his collaborators had been studying since 1977. Dave argued that, in addition to it being a newly discovered member of the *Chloroflexota*, it was known to be an important member of the community, which could not be said for almost all isolates obtained from this well-studied system. The Ward lab’s prior 16 S rRNA analyses had revealed that the microorganisms that had been cultivated from hot spring mats, though certainly present in the mat, were not the predominant members of the community (Ward et al. 1990). A sporting discussion of this ensued, during which Don claimed to know exactly where his “model organism” (*Synechococcus* 7002) came from; a muddy nearshore marine sediment. Dave argued that their work had shown that it isn’t enough to know the general locale or even the specific community from which an organism was cultivated, because cultivation methods are so selective that the true importance of an isolate to the community from which it came couldn’t be known without further study. Don and Dave enjoyed the conversation and agreed that getting together would be a great idea.

Don and Dave had in common a classical microbiology education. In particular, Don had done a postdoc with Roger Stanier at the Institute Pasteur, which gave him a real “Delft School” upbringing. The “Delft School” began when Martinus Beijerinck was appointed as Professor at the Delft Institute of Technology in 1895 (Bos et al. 1983). According to van Niel (1949), a fourth-generation scientific descendent of Beijerinck, “By introducing the principle of enrichment cultures [Beijerinck] opened the way for a rational approach to microbial ecology.” The idea was to cultivate microorganisms that could grow under certain defined environments provided by culture media and incubation conditions. This led to a lineage of students and mentors, who literally discovered the diversity hidden within the microbial world and kept the cultivation approach alive. Roger Stanier was a student of van Niel. Dave’s exposure to a “Delft School” way of thinking was acquired during his graduate studies at the University of Wisconsin-Madison by taking a course in microbial diversity taught by Jerry Ensign. Importantly, Dave’s mentor, Thomas Brock, also taught him the importance of focusing on nature directly for guidance as to what was really going on in situ. Don’s career path led him toward the biochemistry of photosynthesis, while Dave’s led him toward concerns that the traditional approaches in microbiology and microbial ecology were giving a false impression of the composition of natural microbial communities. Dave aimed toward developing cultivation-independent methods that allowed nature herself to tell us which organisms were present. Over 20 years of collaboration, Don came to appreciate how this in situ focus had changed his perspective on microbial ecology and physiology from a traditional view toward the in situ realities of natural communities. This intrigued Don and led him into fertile ground in which he discovered many new phototrophic prokaryotes (Tank et al. 2017), broadening the body of knowledge of diversity in this group and thereby providing a clearer understanding of the evolution of photosynthesis. In turn, Don’s vast knowledge of microbial metabolisms combined with his understanding of emerging omic technologies, dramatically improved our understanding of microbial mat communities, the predominant members, and the in situ physiologies of these community members.

**Fig. 5:**
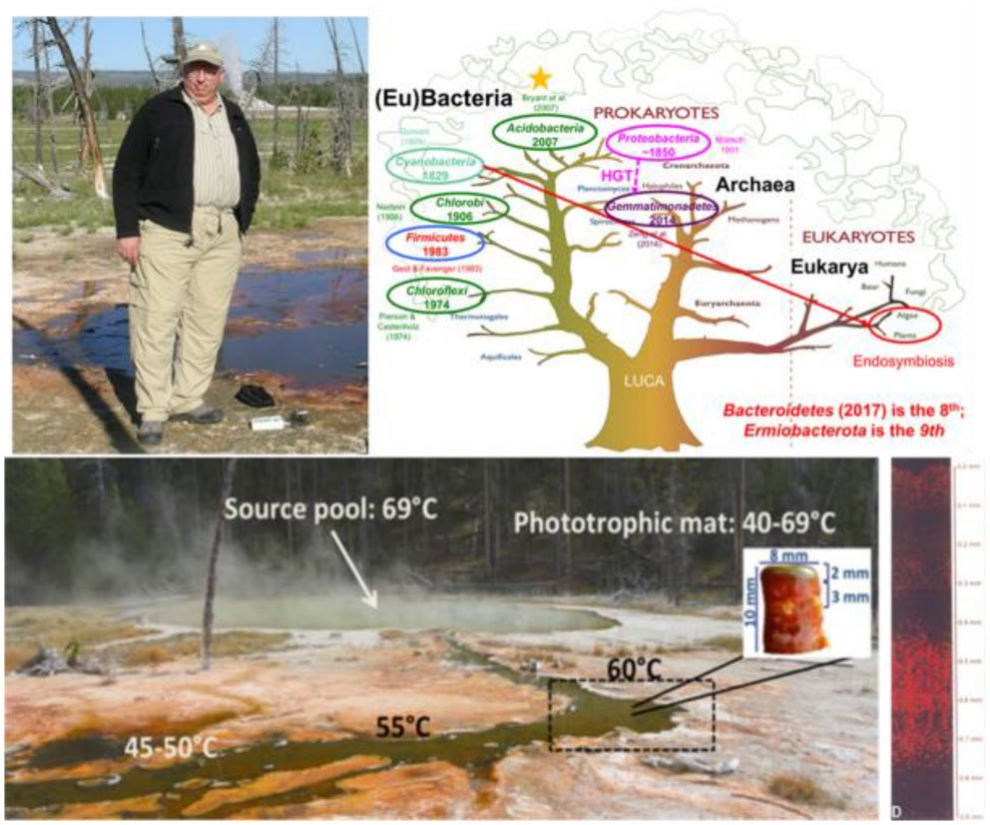
Don “in situ” at the Mushroom Spring microbial mat in Yellowstone National Park. Inset shows a core from the mat and to the right is an autofluorescence microscopy image of the vertical distribution of *Synechococcus* spp. within the 1 mm-thick green layer. A slide from one of Don’s lectures depicts the tree of life, indicating when phototrophs in different lineages were reported (taken from Tank et al. 2017 and Ramsing et al. 2000). Gold star indicated the lineage in which Don discovered the first phototrophic member

Since 1977, Dave’s goal had been to understand the composition, structure and function of hot spring microbial mats as relatively simple, stable and structured, but *natural* communities, from which to observe principles of community ecology. Specifically, his group studied Octopus Spring and Mushroom Spring, alkaline silicious hot springs in Yellowstone National Park that had been previously studied mainly by Brock and Castenholz (Brock 1978). Below ~ 72 °C mats with cyanobacteria (*Synechococcus* spp.) and Chloroflexi (*Chloroflexus* spp.) grow as benthic communities along a thermal gradient in the effluent channels and, at any point, they exhibit a strong vertical structure with cyanobacteria above Chloroflexi (Fig. 5).

To address the issue of determining community composition without biases from enrichment culture, Dave developed cultivation-independent methods involving retrieving and analyzing their 16S rRNA sequences. Between 1990 and Don’s initial phone call, Dave’s lab had shown that (i) the predominant cyanobacterial and Chloroflexi community members were previously undiscovered *Synechococcus* spp. and *Roseiflexus* spp. that were only distantly related to *S. lividus* and *C. aurantiacus* isolates, (ii) cultivated *S. lividus* were many orders of magnitude less abundant than the predominant *Synechococcus* spp. (Ferris et al. 1996), and (iii) the mat likely contained phototrophs distantly related to Chlorobi (Ward et al. 1998). The Ward Lab had initiated a prior collaboration that had provided analysis of the genomic sequences of *Synechococcus* isolates representative of predominant community members, as well as a metagenomic database of the mats (Bhaya et al. 2007). The value of representative isolates and metagenomes was documented by the observation that the predominant *Synechococcus* spp. in the mat possessed genes for nitrogen fixation (Steunou et al. 2006), which *S. lividus* did not contain. That rare *S. lividus* isolate would certainly not have been a good model for the community from which it came! At the outset, Dave saw Don’s genome sequencing of their *Roseiflexus* sp. strain RS-1 isolate as providing insight into this predominant organism’s physiology and fitting into the analysis of the metagenome.

#### Metagenomic analyses of community composition

Because of Don’s comprehensive knowledge of all things phototrophic and his “omics” expertise, Don and Dave’s collaboration took them far beyond this. Of course, genes in a taxon can be observed through genomic analyses, but for this to be relevant to a community requires that one has sequenced a representative culture. Metagenomic analyses provides information about the genes within populations of specific predominant taxa. Gene assemblies comprised of genome segments having similar nucleotide usage frequencies can be separated resulting in metagenome assembly clusters (MAGS) for different taxa (Klatt et al. 2011). Whereas genomes from isolates represent the genes of a single strain of a particular taxon, MAGs represent genes of all related members of a taxon, whether cultivated or not. One of the MAGs represented a heretofore unknown Chlorobi (i.e., *Candidatus* Thermochlorobacter aerophilus), whose 16 S rRNA sequence had been observed. MAG genes suggested it to be an aerobic, BChl *d*-containing photoheterotroph that cannot oxidize sulfur compounds and does not fix bicarbonate autotrophically (Liu et al. 2012).

Dave and Don were able to use metagenomics to better define and understand the major taxa inhabiting the mat, some new to science. Picture the day in 2005, when Don, then a visiting fellow of the Montana State University Thermal Biology Institute, proudly strode into Dave’s office, slapped a phylogenetic tree image on his desk, and declared “*that’s a Science paper*”! While helping Dave’s student Chris Klatt unravel the complexity of MAGs detected in the mats, Don had observed photosynthesis genes in a MAG containing phylogenetic marker genes most closely resembled those of *Acidobacterota*. Don was really excited, as this was a phylum that heretofore had no known photosynthetic members, and he did report it in Science (Bryant et al. 2007). It was the first new bacterial taxon containing phototrophic members discovered in 20 years! The initial “single phototroph” enrichment of the novel organism by Don and his student, Amaya Garcia Costas, already allowed analyses of its photosynthetic apparatus, its chlorosomes and a genome sequencing – partly done in collaboration with the Golbeck lab at Penn State (Tsukatani et al. 2012; Garcia Costas et al. 2012). As it synthesized BChls *a* and *c*, it was presumed to occupy a different niche than *Candidatus* Thermoclorobacter aerophilus. Later, Don’s postdoc Marcus Tank performed a meticulous investigation of the genome and diel (meta)transcriptome results, which informed them of physiological needs of the organism. Marcus worked hard to convince Don, that in addition to a complete change in nutrients (e.g., using amino acids and reduced organic sulfur sources) low levels of oxygen were key to the fastidious nature of this new and unusual phototroph. This approach in combination with careful laboratory studies, eventually yielded a pure culture of *Chloracidobacterium thermophilum* (Tank and Bryant 2015a, b). Importantly, and in their own words: “*This study is an excellent example of how classical microbiology*,* in combination with modern–omics methods (*Bryant et al. 2007; Liu et al. 2011, 2012; Garcia Costas et al. 2012), *led to the discovery and eventually to a fairly comprehensive characterization of this previously unknown bacterium*.” Although now available as axenic and pure culture, *Chloracidobacterium* (*Cab*.) and *Cab. thermophilum* was still not a validly published name, as Yellowstone National Park and the National Park Service require information about and signature of a transfer agreement whenever the strains are passed on to new researchers, which does not align with the rules of the “International Code of Nomenclature of Prokaryotes”; a circumstance and rule which Don openly disapproved of vocally, vividly and regularly. The isolation of a related strain outside from Yellowstone National Park, representing a novel *Chloracidobacterium* species, was meant to fix that. *Chloracidobacterium validum*, with strain BV2-C isolated from a hot spring in Bulgaria representing the new type strain of its species and genus, will finally be validly published in IJSEM this year, sadly enough posthumously (Saini et al. 2025).

In 2012, Vera Thiel joined Don’s lab as a postdoc. She learned metagenomic methods while analyzing the orange colored undermat, the organisms “living in the shadow of the phototrophs” in the Mushroom Spring mat (Thiel et al. 2016, 2017). They learned that although this was expected to be the “dark side” of the mat, which was (almost) void of (visible) light, the most abundant members of the community were phototrophic *Roseiflexus* spp. As the mat environment is highly variable, especially in the vertical dimension and temporally, they were not surprised to find several *Roseiflexus* spp. populations, which most likely show adaptations to different ecological niches (e.g., different oxygen, light or temperature conditions), similar to the cyanobacterial ecotypes detected in the upper mat community (see below).

When Vera first presented her undermat metagenome binning results in their internal lab seminar, Don– with his unerring instinct for finding the highlights - immediately pointed out a MAG containing dissimilatory sulfate metabolism genes in combination with taxonomic markers of the *Bacteroidetes*/*Chlorobi* (now *Chlorobiota*) as something novel and worth looking further into. Vera and Don later described this unusual and novel thermophilic sulfate-reducer as “*Candidatus* Thermonerobacter thiotrophicus” (Thiel et al. 2019).

Don enjoyed collaborations involving metagenomic analyses of other mats with other members of the Yellowstone Research Coordination Network, which was created and managed by Montana State University colleague Bill Inskeep (Inskeep et al. 2013a, b).

#### Community structure

Don helped Dave’s students understand the nature of *Synechococcus* species, which Eric Becraft, in collaboration with Fred Cohan (Wesleyan University) had demonstrated were found at different depths in the mat (Becraft et al. 2015). Dave’s student Shane Nowack had shown that representative isolates of these species were adapted to different irradiance levels (Nowack et al. 2015). Don then guided Dave’s student Millie Olsen to discover through comparative genomics, that low-light adapted species, which resided deeper in the mat green layer, contain a gene cassette encoding unique allophycocyanin proteins (Olsen et al. 2015). These strains expressed these genes at low irradiance levels, allowing them to shift their light absorption spectrum into the near-infrared region of the light spectrum and enabling faster growth than organisms residing above. This exemplified how the acquisition of a few genes can lead to the evolution of a new ecological species. Similarly, Dave’s student Jess Allewalt had observed that strains representative of *Synechococcus* populations that were distributed to different temperatures along the effluent channel were adapted to different temperatures (Allewalt et al. 2006), Marcus was able to cultivate strains of *Cab. thermophilum* that were adapted to different temperatures (Saini et al. 2021). Again, adaptations were subtle and involved extensions of the upper and lower temperature limits rather than wholesale shifts of temperature optima.

#### Community function

Don and his student Jay Liu pioneered the analysis of the mat community through metatranscriptomics (Liu et al. 2011). In September 2011, the Ward and Bryant labs performed a comprehensive collection of mat samples made at hourly intervals throughout an entire diel cycle and let the mRNA messages tell them what genes the different taxa were expressing when. For Dave, this was simply amazing! He had first learned about transcription and “the central dogma” in high school and here they were doing it for all the predominant microorganisms inhabiting an entire microbial community at once! This was possible because the MAG sequences defining major taxa served as a reference database such that individual transcripts could be associated through sequence similarity with the taxon that produced them. In addition, Michael Kühl (University of Copenhagen) provided microsensor analyses of irradiance and oxygen (Becraft et al. 2015), Young-Mo Kim (Pacific Northwest National Laboratory) provided metametabolomics analysis of organic compounds in the mat, Shane Nowack and his mentor Isaac Klapper (Mathematics Department, Montana State University)provided analyses of gases in the water overflowing the mat (Kim et al. 2015), and Laurey Steinke (University of Nebraska Medical School) provided metaproteomics analyses (Schaffert et al. 2012). Metatranscriptomics datasets provided diel transcription profiles for *Synechococcus* spp., three members of the Chloroflexi (*Roseiflexus* spp., *Chloroflexus* spp. and *Ca.* Roseilinea spp.), *Cab. thermophilum*, and *Cand*. Thermochlorobacter aerophilum genes, which could be interpreted relative to these databases (Liu et al. 2012; Klatt et al. 2013).

Here are some examples of what they found the major phototrophic community members doing: Liu et al. (2011) reported that *Synechococcus* transcription patterns for photosynthesis and nitrogen fixation genes tracked with irradiance and anoxic conditions in the mat, as expected (Liu et al. 2012). Eric Becraft was able to separate transcripts associated with *Synechococcus* species residing at the top of the mat and those of underlying species and demonstrate that these genes associated with the upper species were transcribed sooner than those associated with deeper species. This was likely because light penetration to deeper mat regions occurs after light impinges on the surface of the mat. Surprisingly, Jay Liu found that transcripts for *Synechococcus* fermentation genes, which were most highly expressed at night, began to accumulate beginning in mid-afternoon, well before the mat became anoxic in the evening. Metametabolomics showed a coincident rise in lactate. Dave’s postdoc Niels Ramsing had previously shown using oxygen microsensors that photosynthesis is highest at the mat surface until late morning, when the photosynthesis peak drops to deeper subsurface layers (Ramsing et al. 2000). The midday shift to fermentation might be a result of photoinhibition or inhibition by ultraviolet light (Miller et al. 1998) of *Synechococcus* species at or near the mat surface. A midday spike of glycolate also indicated that surface *Synechococcus* species were physiologically stressed, when extreme high oxygen and pH levels were observed.

Don helped Chris Klatt discover genes of the 3-hydroxypropionate (3OHP) in the *Roseiflexus* sp. strain RS1 genome and MAG (Klatt et al. 2007, 2011; van der Meer et al. 2010). Metatranscriptomic analyses revealed the unexpected result that these genes are expressed throughout the day (Klatt et al. 2013). Relying too much on canonical thinking about anoxygenic phototrophs, the Ward and Bryant labs had dismissed the idea that *Roseiflexus* could incorporate bicarbonate during the day, when the mat is oxic and reductants are absent (van der Meer et al. 2005; Revsbech et al. 2016). Based on the coordinated transcription patterns of key genes in the 3OHP, TCA cycle, and genes encoding enzymes involved in β-oxidation of fatty acids, and metametabolomics observations that polyhydroxyalkanoites produced at night declined during the day (Kim et al. 2015), Chris hypothesized that *Roseiflexus* spp. performed photomixotrophy during the day which involved simultaneous fixation of bicarbonate and organic matter in a manner to that described by Zacharya and Fuchs for *Chloroflexus aurantiacus* (Zarzycki and Fuchs 2011). Dave, Don and collaborators at The Pacific Northwest National Laboratory were able to test this hypothesis using stable-isotope probing of the proteins of mat taxa (Schaffert et al. 2012). ^13^C from ^13^C-labeled substrates caused mass spectral shifts in peptides of proteins unique to specific mat taxa (Steinke et al. 2020). They showed that ^13^C-bicarbonate and ^13^C-acetate and -propionate, glycolate and -lactate are incorporated into *Roseiflexus* proteins during the daytime (Moran et al. sumitted). ^13^C-bicarbonate incorporated under infrared light accounted for over half of the total fixed in full-light, indicating that this process is as or more important than photoautotrophy by *Synechococcus* spp. This is important because this process is conducted using the 3-hydroxypropionate pathway, not the Calvin-Benson-Basham Cycle used by cyanobacteria. These pathways have different degrees of fractionation of stable carbon isotopes, the former resulting in fixed carbon that is depleted in the heavier isotope (^13^C) by ~ 13‰, and the latter by ~ 20‰ “lighter”. If cyanobacteria alone were fixing bicarbonate, the mat organic matter should be 2 “lighter” than the bicarbonate from which it is made. Their finding helped to understand why mat organic matter is isotopically “heavier” than that. This matters to geochemists looking for evidence of bicarbonate fixation in ancient rock formations, including stromatolites, which are considered fossilized photosynthetic microbial mats.

Don’s ambition, intellectual capacity and breadth of knowledge certainly helped us move into a new era of understanding the ecology of these microbial communities. In the process we learned that it is wisest to let nature, as opposed to “conventional wisdom”, guide you as to what goes on in situ. Neither Don nor Dave saw modern molecular approaches as replacing classical methods. Indeed, Don and his students found ways to exploit molecular results in order to design culture media that would nurture representative isolates into culture, where they could be studied in detail by conventional means.

### Far-red light photoacclimation (FaRLiP) (by Christopher J. Gisriel, Gaozhong Shen, Fei Gan, and Ming-Yang Ho)

One of Don’s most influential contributions to science was the discovery and characterization of far-red light photoacclimation (FaRLiP), a facultative cyanobacterial acclimation mechanism that allows extension of the absorbance cross section into the far-red. The road to this discovery began due to Don’s long-term interest in the biogenesis and regulation of photosynthesis. He wondered how cyanobacteria would contribute to iron redox cycling while coping with iron-related oxidative stress. Collaborating with Dr. Igor Brown at NASA, they isolated and characterized multiple cyanobacterial strains from iron-depositing hot springs. One of these strains, *Leptolyngbya* sp. JSC-1 (hereafter JSC-1) which was isolated from a floating mat in an iron-rich thermal feature associated with La Duke Hot Spring near Gardiner, MT and Yellowstone National Park, exhibited a requirement of high levels of Fe for growth (≥ 40 µM), but also accumulated large amounts of extracellular and intracellular iron (Brown et al. 2010). They also sequenced the genome to find that the strain JSC-1 encodes multiple copies of *isiA* (*i*ron-*s*tress *i*nduced protein *A*) and a fusion gene of *isiA* and *psaL*, which were later confirmed to be expressed and assembled into PSII and/or PSI to facilitate light harvesting upon iron starvation (Shen et al. 2016).

Interestingly, based on the yet-unassembled genome contigs at that time, the JSC-1 genome contained a unique gene cluster composed of 21 genes encoding proteins that include subunits of PSI, PSII, and PBS. This phenomenon caught Don’s attention immediately. It is not uncommon for cyanobacteria to have multiple copies of photosynthesis genes (e.g., *psbA* and *isiA*), especially for PSI core components, and it would be unreasonable for JSC-1 to encode the genes but not make use of them. The question then became: under what growth conditions would cells express those genes in the alternate gene cluster and thus assemble different phycobilisomes and photosystems? Efforts were made to characterize the photosynthetic apparatus assembled in JSC-1 cells when cultivated under common white, fluorescent light or nutrient (e.g., iron and nitrogen) replete or depleted conditions, which collectively demonstrated that JSC-1 used the same set of genes (e.g., *psaA1* and *psaB1*) to assemble the photosynthetic apparatus. Collaborating with Dr. J. Clark Lagarias at UC Davis, Don found that the photosensory RfpA is a knotless red/far-red light phytochrome. While a broad range of visible light converts it into far-red-absorbing (P_fr_) form, only FRL (700–800 nm) triggers its reversion to the red-absorbing (P_r_) form. This suggests that JSC-1 would behave differently only when cultivated in FRL. JSC-1 was thus cultivated under different light conditions, including FRL. Using combined approaches, including transcription profiling, biochemical and spectroscopic analyses, and proteomics, it was revealed that JSC-1 exhibits an extensive photo-acclimative response to growth in FRL, including the synthesis of Chls *d* and *f*, and remodeling of the whole photosynthetic apparatus with the products of the paralogous genes in the 21-gene cluster. This acclimative response enhances light harvesting for wavelengths complementary to the growth light and enhances oxygen evolution in FRL. This is when Don first coined the term “FaRLiP”, and named the red/far-red phytochrome and its two regulatory factors RfpA, RfpB, and RfpC (*r*egulator of *f*ar-red *p*hotoacclimation), respectively (Gan et al. 2014).

With the discovery of FaRLiP in JSC-1, Don asked more questions regarding the ecological significance of FaRLiP-capable cyanobacteria in nature and the underlying mechanisms of oxygenic photosynthesis driven by FRL. To understand how the remodeled photosynthetic apparatus functions to use FRL, Don first systematically analyzed the paralogous photosynthesis gene sequences in cyanobacterial genome databases. Searching the genome database for homologs to the characteristic genes, including *psbA4* and *apcE2*, in the FaRLiP gene cluster led to the discovery of more strains from diverse natural environments, many of which were experimentally confirmed to be capable of FaRLiP (Gan et al. 2015). It became clear that FaRLiP occurs widely in natural environments enriched in FRL, such as soils, under-canopy/shade, microbial mats, and aquatic blooms. Phylogenetic analysis of PsbA sequences, which normally coordinates the Mn_4_Ca_1_O_5_ cluster in PSII, demonstrated that the *psbA4* within the FaRLiP gene cluster encodes a “super-rogue” PsbA lacking ligands to the Mn_4_Ca_1_O_5_ cluster. Later, Don’s group generated knockout mutants of *psbA4* in *C. fritschii* PCC 9212 and *Synechococcus* sp. PCC 7335 (hereafter *Synechococcus* 7335), revealing that PsbA4 functions as a Chl *f* synthase, and *psbA4* was renamed as *chlF* (Ho et al. 2016). The deletion of *psbA4* causes cells incapable of synthesizing Chl *f*, while the expression of other genes within the FaRLiP gene cluster remains unaffected in FRL. The heterologous expression of PsbA4 in the model cyanobacterium *Synechococcus* 7002, which is incapable of synthesizing Chl *f*, allows the light-dependent conversion of Chl *a* to Chl *f*. Consequently, this gene was renamed *chlF*, indicating it encodes a light-dependent Chl *f* synthase (Ho et al. 2016). Don’s additional characterization indicates that ChlF forms homodimers that bind Chl *a* and pheophytin *a* when expressed in *Synechococcus* 7002 (Shen et al. 2019). Don pointed out that the discovery of ChlF represents a significant advancement, as a paralog of an enzyme evolves to acquire a novel function. Following Don’s discovery of ChlF, several research articles characterizing ChlF have been published (Trinugroho et al. 2020; Chen et al. 2023; Agostini et al. 2023; Qi et al. 2025).

Regarding phycobiliproteins, Don observed that some *apc* genes in the FaRLiP gene cluster lack a broadly conserved Cys residue that typically forms a covalent bond to a phycocyanobilin chromophore. He hypothesized that a lack of this Cys would red-shift the chromophores by extending their conjugated double bond systems which was later confirmed by biochemical (Soulier and Bryant 2021; Soulier et al. 2022) and structural (Gisriel et al. 2023e) studies.

To further investigate the function of each subunit within the FaRLiP gene cluster, Don developed a genetic system to generate knockouts. The initial successful cases involved *Chlorogloeopsis fritschii* PCC 9212 and *Chroococcidiopsis thermalis* PCC 7203, where RfpA, RfpB, and RfpC were individually knocked out via conjugation and homologous recombination (Zhao et al. 2015). An equivalent method was subsequently employed to knockout RfpA, RfpB, and RfpC in *Synechococcus* 7335 (Ho et al. 2017a). Subsequent transcriptomic analysis indicated that the absence of any of the three Rfp proteins impairs the activation of genes within the FaRLiP gene cluster (Ho and Bryant 2019). The findings confirmed that RfpA, RfpB, and RfpC are critical regulators for activating FaRLiP in FRL. Additionally, Don published findings on knockout mutagenesis of the five APC subunits, demonstrating their correlation with Chl *d* synthesis (Bryant et al. 2020b).

After much of the biochemical characterization of the photosystems present during FaRLiP, Don set his sights on determining their molecular structures to identify the Chl *d* and *f* binding sites and structures of the novel FRL-specific subunits using cryo-electron microscopy (cryo-EM). Don’s first cryo-EM structure was of FRL-PSI, from the thermophilic cyanobacterium *Fischerella thermalis* PCC 7521 (Gisriel et al. 2020a). Despite the somewhat low resolution, this work provided some of the first evidence as to where Chl *f* molecules bind in FRL-PSI and revealed the structures of FRL-specific subunits, called PsaA2, PsaB2, PsaF2, PsaI2, PsaJ2, and PsaL2 in complex with subunits not regulated by FaRLiP, PsaC, PsaD, PsaE, PsaK, and PsaM. This structure of FRL-PSI, and one from *Halomicronema hongdechloris* published by another group (Kato et al. 2020), also led to important insight on the challenges of differentiating small cofactors exhibiting minor differences in cryo-EM maps (Gisriel et al. 2020b). From this arose techniques to address those challenges (Gisriel et al. 2021, 2023d; Ranepura et al. 2023; Consoli et al. 2024). Don also contributed another FRL-PSI structure, from the mesophilic marine cyanobacterium *Synechococcus* 7335 (Gisriel et al. 2022b), which together with the structures from *F. thermalis* PCC 7521 and *H. hongdechloris* provided valuable insight into the variation of Chl *f* binding sites among species. This further led to structure- and phylogenetic-based analyses of FRL-PSI, providing insight into the molecular evolution of FaRLiP (Gisriel et al. 2023b).

Especially based on the fact that a FRL-absorbing Chl molecule was expected to bind in the electron transfer chain of FRL-PSII, Don also led efforts in determining structures of that complex. He published two structures, both from *Synechococcus* 7335. The first was a monomeric PSII complex (Gisriel et al. 2022c). Although the structure lacked several subunits, most notably the FRL-specific PsbH2, it contained the major FRL-specific subunits PsbA3, PsbB2, PsbC2, and PsbD3 and all four expected FRL-absorbing Chl sites were assigned, the one Chl *d* and three Chl *f* molecules. Soon after, Don published the second cryo-EM structure of FRL-PSII from this organism, which was a dimeric complex, although still missing some non-FRL-specific subunits (Gisriel et al. 2023f). However, this structure allowed for the resolving of PsbH2, which completed structure determination of all known FRL-specific photosystem subunits involved in FaRLiP. It was shown that PsbH2 alters the site energy of a Chl *a* molecule probably important in energy transfer. Especially based on his structural work on FRL-PSII, Don also helped to publish an evolutionary analysis on FRL-PSII (Gisriel et al. 2022a).

As mentioned above, both the photosystems and the PBS are altered during FaRLiP. Don’s final direct contribution to understanding the structural basis of FaRLiP was in determining the cryo-EM structure of a FRL-absorbing phycobiliprotein complex from *Synechococcus* 7335 (Gisriel et al. 2023e). In vivo, the complex is thought to have a bicylindrical organization with each cylinder containing 14 allophycocyanin subunits (Ho et al. 2017b). Although this structure was only of a single cylinder, and lacked four of the expected subunits thought to be present in vivo, the complete structure could easily be extrapolated by comparison to other core cylinder structures (for example (Zheng et al. 2021) and (Domínguez-Martín et al. 2022) and subunit composition analyses (Ho et al. 2017b). The structure showed that the lowest energy phycocyanobilin chromophores are located near the interface of where the complex would be expected to interface with FRL-PSII, providing the structural basis for previously-determined time-resolved fluorescence spectroscopy data (Ho et al. 2020). Don was greatly amused by the fact that his career both started (Glazer and Bryant 1975) and ended with studying red-shifted allophycocyanins.

### Low light photoacclimation (LoLiP) (by Nathan T. Soulier, Christopher J. Gisriel, and Gaozhong Shen)

Don’s research appointment at Montana State University from 2009 to 2020 yielded many exciting discoveries in the complex world of microbial mat ecology. As described above, Don and coworkers teased apart inner workings of the green photic layer in mat samples from Mushroom Spring, Yellowstone National Park. In a series of three publications in 2015 collectively titled “The Molecular Dimension of Microbial Species” (Becraft et al. 2015; Nowack et al. 2015; Olsen et al. 2015), the group described unique adaptations of thermophilic *Thermostichus* (formerly *Synechococcus*; Strunecký et al. 2023) ecotypes isolated from different depths within the mat. Among other discoveries, it was found that low-light ecotypes (LLEs) isolated from a depth of about 2 mm within the green photic layer of the mat grew more rapidly at low irradiance than high-light ecotypes (HLEs) isolated nearer the mat surface (Becraft et al. 2015; Nowack et al. 2015). Unlike the HLEs, cells of LLEs also possessed a far-red absorption feature (Nowack et al. 2015) and a unique 4-gene cluster encoding a putative cyanobacteriophytochrome photoreceptor, two paralogous allophycocyanin (AP) subunits, and a putative Chl-binding protein (Olsen et al. 2015). Over the next decade (2015–2024), strains representing both ecotypes, the regulation of the unique gene cluster, and the products of the cluster were the focus of several studies uniting two of Don Bryant’s primary research themes: microbial ecology and photosynthesis.

Don and coworkers first showed that the AP encoded by the LLE gene cluster absorbed FRL (Soulier et al. 2020) and was similar to APs associated with FaRLiP (Gan et al. 2014; Ho et al. 2017b). They also demonstrated that prolonged growth at low irradiance resulted in emergence of a FRL absorbance feature, corresponding to the far-red absorbance and fluorescence emission observed by the Ward lab (Nowack et al. 2015; Soulier et al. 2022). After these discoveries, the new photoacclimation mechanism in cyanobacterial strains with expression of the 4-gene cluster was termed “LoLiP”, for *Lo*w-*Li*ght *P*hotoacclimation (Ho et al. 2017c; Soulier et al. 2022). Unusually, the LoLiP AP appeared to be associated with Chl-binding proteins rather than the visible light-absorbing PBS megacomplexes. Co-localization in sucrose density gradient centrifugation suggested formation of a novel complex, perhaps with the putative Chl-binding protein from the LLE cluster, IsiX (Soulier et al. 2022). Gisriel and coworkers later collaborated with Don to show that ApcD4 and ApcB3 from the LoLiP gene cluster form helical nanotubes in solution, rather than the trimeric toroids characteristic of AP in the PBS core (Gisriel et al. 2023a, c).

The final predicted open reading frame in the LoLiP cluster encodes a putative cyanobacteriophytochrome (CBCR) photoreceptor with two GAF domains. Don and coworkers determined that the first GAF domain covalently bound a bilin chromophore, making it a verified photoreceptor they named LcyA (“*L*oLiP *Cy*anobacteriophytochrome”). In collaboration with John Golbeck’s lab at Penn State, they also showed that the second GAF domain coordinates a 4Fe-4 S cluster, the first described CBCR to do so. Coupled with a C-terminal histidine kinase, LcyA was hypothesized to regulate transcription of the rest of the LoLiP cluster in response to one or more environmental cues sensed by its GAF domains (Soulier et al. 2022).

Regulation of LoLiP remains an open question. RT-PCR performed in Don’s lab demonstrated that the four LoLiP cluster genes are co-transcribed, but RNA sequencing only showed a slight increase in LoLiP transcripts after growth in LL compared to cells harvested from HL. Considering this and the two very different GAF domains in LcyA, Don and coworkers suggested that strong induction of LoLiP gene expression might require more than one environmental cue (Soulier et al. 2022). It is presently unknown how common LoLiP or similar phenomena are in cyanobacteria due to limited studies of genetic data from cyanobacterial mats, but related operons with dissimilar or no putative photoreceptor have been identified in other terrestrial cyanobacteria, including some capable of FaRLiP (Soulier 2021). Despite these lingering questions, Don’s work on LoLiP expanded the known diversity of cyanobacterial photoreceptors (Soulier et al. 2022) and phycobiliproteins (Soulier and Bryant 2021; Gisriel et al. 2023a) and deepened our understanding of niche adaptation and acclimation in microbial mat communities.

### Photosystem I and much more (by John H. Golbeck)

It goes without saying that Don Bryant was a scientist of exceptional ability. I will focus this narrative on our collaborative work on PSI and leave it to others to speak about his other accomplishments. Although some of the details including times and dates have grown fuzzy with time, I will try my best to represent our work together in a chronological fashion. I first met Don in 1986 at the VIII^th^ International Congress on Photosynthesis held that summer at Brown University. Beth Gantt approached me and said that there is somebody I should meet. Both of us walked over to Don, who was sitting in the shade under a tree, and we were introduced with (I paraphrase) ‘I think you two guys might be able to do some work together’. Don and I exchanged pleasantries, and we talked about our work. While at Martin Marietta Laboratories, I had developed a method to denature and remove the F_A_/F_B_ containing protein (later known as PsaC) from the P700-F_X_ core of cyanobacterial PSI. In 1985, the Biosciences Department was eliminated by the newly formed Lockheed Martin Corp, and I joined the Chemistry Department at Portland State University where I was fortunate to be offered an adjunct appointment at the Oregon Graduate Center and have access to an EPR spectrometer at the Oregon Health Sciences University. Working with visiting professor Isamu Ikegami, my graduate students Kevin Parrett and Tetemke Mehari developed a protocol to isolate the intact F_A_/F_B_ containing protein and to bind it back onto a purified P700-F_X_ core. This resulted in a fully functional reaction center. Around this time, the N-terminal sequences of what later became known as the PsaC, PsaD and PsaE stromal subunits of PSI were being reported by John Gray, as well as his identification of the *psaC* gene that coded for the F_A_/F_B_-containing protein. Don had been working on PBS, but he had become interested in PSI and was learning *E. coli* cloning techniques and applying them to cyanobacteria with Ron Porter. Beth knew this and it likely motivated her to make our introduction.

In 1988, Don invited me to the Penn State Summer Symposium in Molecular Biology on ‘Light-Energy Transduction in Photosynthesis’. After we each gave our talks, we realized that a path was open to reconstitute my P700-F_X_ cores with his native and altered stromal subunits. Our first publication appeared in 1990, in which we showed that PSI activity could be restored by binding the products of the *psaC* and *psaD* genes expressed in *E. coli* onto purified P700-F_X_ cores (Zhao et al. 1990). We found that the assembly was cooperative: PsaC would not bind in the absence of iron-sulfur clusters F_A_ and F_B_, and PsaD and PsaE would not bind in the absence of PsaC. No structures were yet available for PSI (a 4 Å structure was published in 1996, and a 2.5 Å structure only in 2001), so this work provided the first hints into how the stromal subunits were assembled. Our individual National Science Foundation grants were soon up for renewal. Don, who was on sabbatical leave in Zürich, and I decided to write a review article that could serve, in part, as the background section for our proposals. We communicated by way of Bitnet, a relatively new protocol that enabled fast e-mail and file transfers between universities. I remember arriving at my office and finding beautifully written sections on the genetics of PSI awaiting me each morning. We merged our sections and the review, published in 1991 in *Current Topics in Bioenergetics* (Golbeck and Bryant 1991), has become one of our most cited publications.

**Fig. 6:**
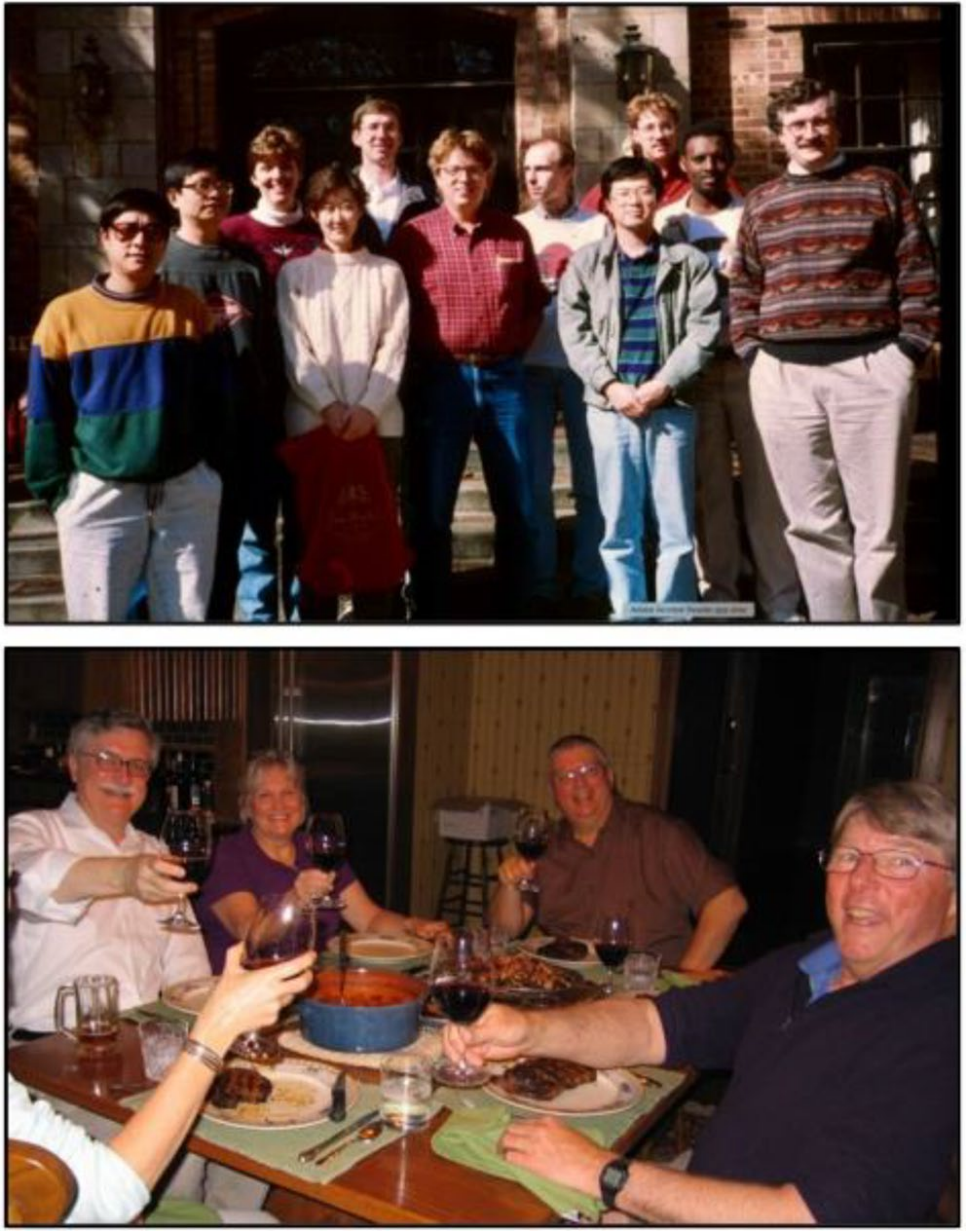
Golbeck and Bryant research group (top) and dinner at Don’s (bottom). Top: The Golbeck and Bryant research groups at the Midwest Photosynthesis Conference, Turkey Run State Park at Marshall, Indiana circa 1992/1993. Pictured are (left-to-right) Lian Yu, Jindong Zhao, Wendy Schluchter, Soohee Chung, David Nellis, Don Bryant, Uli Mühlenhoff, Yean-Sung Jung, Patrick Warren, Tetemke Mehari, and John Golbeck. Bottom: Dinner at Don’s house on May 2, 2009 when Dave Ward was visiting Penn State to give a seminar. Pictured are (left-to-right) John Golbeck, Nancy Ward, Don Bryant and Dave Ward. Don and Dave were close collaborators on the *C. thermophilum* project

In 1990, the Ph.D. programs at Portland State were under threat of elimination by the larger state universities and the governor, so I accepted a position in the Biochemistry Department at the University of Nebraska-Lincoln. One outcome of this move was that Don’s lab and mine were now sufficiently close to allow our research groups to meet annually at the Midwest Photosynthesis Conference held at Turkey Run State Park, IN (Fig. 6, top). Our individual National Science Foundation grants were again up for renewal. It occurred to Don and me that because of the interdependence of our research programs, we both could be in jeopardy should one of our proposals be turned down. We discussed the problem with our program directors in the Molecular Biophysics and Metabolic Biochemistry directorates and the solution found was to have both of our proposals reviewed in a single panel with experts from both areas. Once funded, we explored in the years from 1990 to 1996 the properties and roles of PsaD forward electron transfer (Li et al. 1991) and PsaE in cyclic electron transfer (Lian et al. 1993), and we studied the effect when the Cys ligands to the [4Fe-4 S] clusters constituting F_A_ and F_B_ of PsaC were altered. The latter was particularly fruitful because the spin-state crossover from S=½ to S ≥ 3/2 that occurred on the substitution of a Cys for a Ser allowed us to assign the F_A_ and F_B_ clusters relative to the protein frame (Zhao et al. 1992; Mehari et al. 1995). We also realized that on the substitution of a Cys for a Gly in PsaC, a 2-mercaptoethanol was retained on the [4Fe-4S] cluster at the open coordination site, functioning as a rescue ligand (Jung et al. 1996). This would become important for our later work on solar biofuels.

Our research programs were by now increasingly interdependent, and Don and I considered moving to be at the same university. In 1996, Don interviewed in the Biochemistry Department at the University of Nebraska, and I interviewed in the Department of Biochemistry and Molecular Biology at Penn State. The latter worked. Moving to Penn State allowed us to share graduate students and postdoctoral scientists; to co-serve on our student’s committees; and to have impromptu meetings. Our grants were again up for renewal, and our program directors suggested that we combine our proposals to carry out what the National Science Foundation considered at that time a new and bold experiment. Following favorable reviews, we were offered five years of funding with the comment (again, I paraphrase) ‘we want to see if you two guys from such different disciplines can work together without killing each other’. Don served as PI and I served as Co-PI, and in total, we obtained five 5-year cycles of funding that lasted 25 years. In the interval from 1996 to 2009, we largely worked on issues dealing with PSI. One highlight is that graduate student Mikhail Antonkine traveled to Ivano Bertini’s laboratory in Florence and to Dietmark Stehlik’s laboratory in Berlin to solve the NMR solution structure of unbound PsaC (Antonkine et al. 2002), thereby providing clues as to how PsaC docks asymmetrically on a symmetrical P700-F_X_ core (Jagannathan and Golbeck 2009). Lee McIntosh and I had a grant from the United States Department of Agriculture to study iron-sulfur cluster assembly, and Don joined us to uncover the function of the product of *sll0088* in *Synechocystis* sp. PCC 6803 as the transcriptional repressor of the *suf* operon involved in iron-sulfur cluster biogenesis (Wang et al. 2004). When Lee died in 2004, Don replaced Lee as co-PI and the two of us subsequently found that SufR contains a [4Fe-4S] cluster that senses the cellular titer of iron-sulfur clusters, making SufR an autoregulator (Shen et al. 2007).

In 2005, Don and I became aware of a new funding opportunity from the Materials Science Division of the U.S. Department of Energy that called for entirely new strategies to produce dihydrogen. I realized that if we could ligand-exchange the 2-mercaptoethanol in the Gly_13_ position of PsaC with a molecular wire, we should be able to attach a catalyst at its terminus and generate hydrogen in the light. Our proposal was one of the few biological projects funded in that Division. I served as PI and Don served as Co-PI; in total, we obtained five 3-year cycles of funding that lasted 15 years. In this work, we isolated P700-F_X_ cores, attached PsaD and a Cys_13_Gly variant of PsaC, displaced the rescue 2-mercaptoethanol ligand at that position with a 1,6-hexanedithiol molecular wire, and bound a Pt nanoparticle to the terminal thiol group. The construct generated dihydrogen in the light at a respectable rate of 50 µmol H_2_ mg Chl^− 1^ h^− 1^ (Grimme et al. 2008). We learned that the distal [4Fe-4S] cluster of the [Fe-Fe] hydrogenase from *Clostridium acetobutylicum* was surface-located. When graduate student Carolyn Lubner bound the sulfhydryl group of the molecular wire with a Cys_97_Gly variant of [Fe-Fe]H_2_ase provided by Thomas Happe, she achieved an extraordinary rate of 2,200 µmol H_2_ mg Chl^− 1^ h^− 1^ (Lubner et al. 2011). This electron transfer rate exceeded that of natural photosynthesis by more than a factor of two, largely due to the elimination of diffusion mediated steps. In the decade from 2010 to 2018, our basic research interests grew to include the characterization of enzymes involved in assembly. We worked on VIPP1 as an essential protein for the biogenesis of PSI (Zhang et al. 2014); on ChlR as a [4Fe-4S]-containing transcriptional activator of pigment biosynthesis (Ludwig et al. 2014); on BciB as a ferredoxin-dependent 8-vinyl protochlorophyllide reductase (Saunders et al. 2013); and on BciD as a radical SAM enzyme involved in bacteriochlorophyllide *e* biosynthesis (Thweatt et al. 2017). We also carried out biochemical and biophysical studies on the homodimeric Type I reaction center in *C. thermophilum*, a chlorophototroph discovered earlier by Don and Dave Ward, and showed that the primary donor absorbs at 840 nm (Tsukatani et al. 2012). We later worked with K.V. Lakshmi to show using 2D HYSCORE that the primary donor P840 is a Zn-BChl *a* dimer (Charles et al. 2020).

**Fig. 7:**
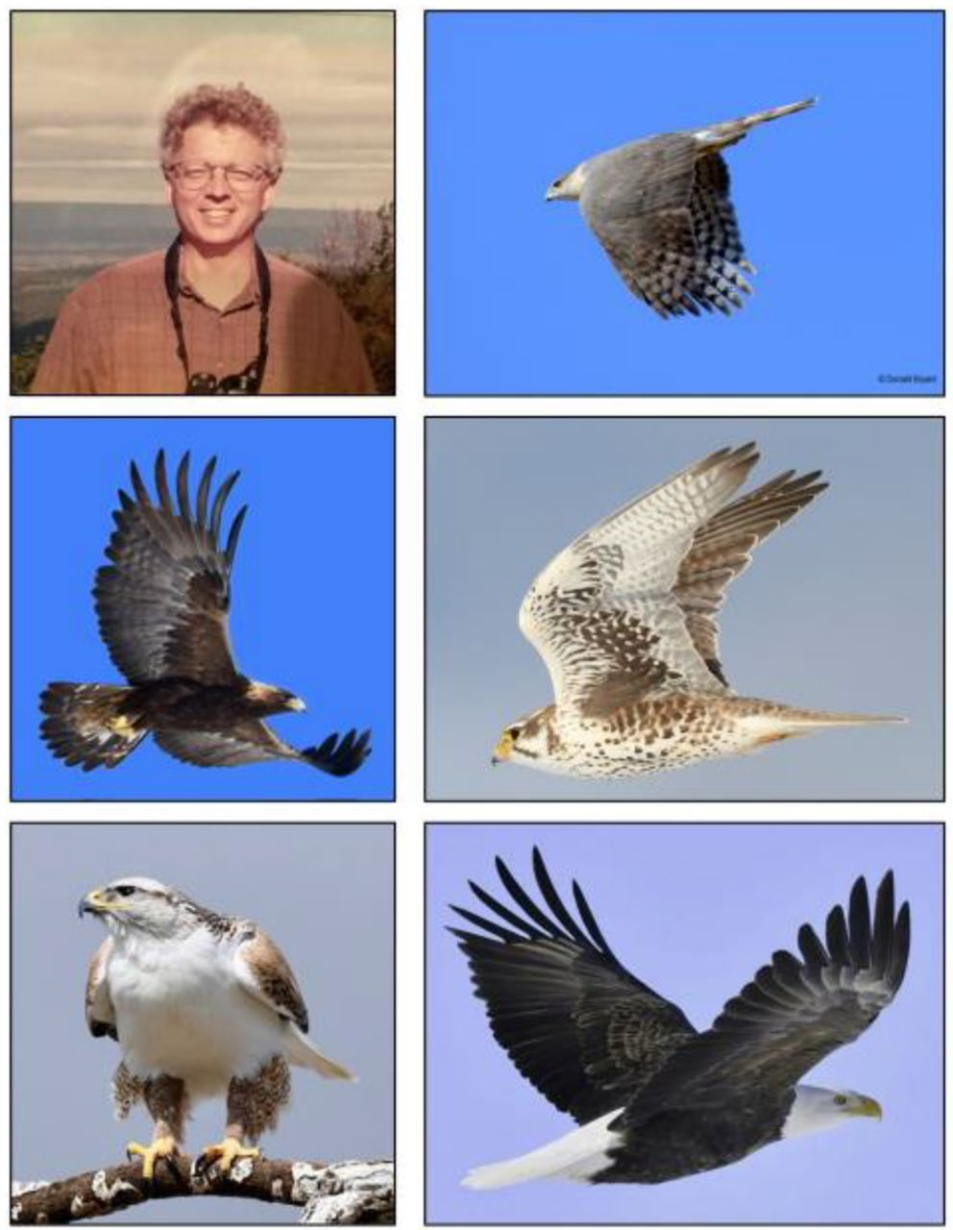
Don and some of his raptor photography. Top left: Don at his favorite raptor spotting site in PA. Top right: Rough-legged Hawk. Center left: Golden Eagle. Center right: Prairie Falcon. Bottom left: Ferruginous Hawk. Bottom right: Bald Eagle

Don and I both began phased retirement in 2019 and we fully retired in 2022 although this simply meant that we no longer taught or did committee work. Don had become interested in FRL photosynthesis. The earlier discovery of pigments such as Chl *d* and Chl *f* that absorb in the near-IR had created excitement in the field because their incorporation into crop plants has the potential to increase agricultural productivity. Our joint effort focused on the structure and function of FRL absorbing reaction centers. We worked with Chris Gisriel, who solved the structure of Chl *f*-containing FRL PSI (see section on FaRLiP) (Gisriel et al. 2020a). We collaborated with Dmitry Cherepanov and Alexey Semenov, who carried out femtosecond kinetic studies of FRL PSI from *Synechococcus* 7335 and showed that Chl *f* functions not in the reaction center core but in the antenna (Cherepanov et al. 2020). Postdoctoral scientist Vasily Kurashov engineered Chl *f* into PSI of a wild type cyanobacterium, and demonstrated energy transfer from the Chl *f* pigments to the trapping center (Kurashov et al. 2019). In our last paper together, Don and I worked again with the femtosecond group in Moscow to characterize energy redistribution between Chl *a* and Chl *f* and to measure the kinetics of the primary charge separated pair in Chl *d* and Chl *f*-containing Photosystem II (Cherepanov et al. 2024).

In our years of working together Don and I published 61 peer-reviewed papers and two review articles. Our work ranged in scope from the biological, published in American Society for Microbiology’s *Journal of Bacteriology* to the physical, published in the Royal Society Journal *Physical Chemistry Chemical Physics.* The greatest lesson Don taught me was that microbiology and genetics are the engines of discovery in biology. This came as quite a shock to someone who was trained as a chemist. We both acknowledged that biophysics and spectroscopy would be the means by which these new discoveries are understood.

**Fig. 8:**
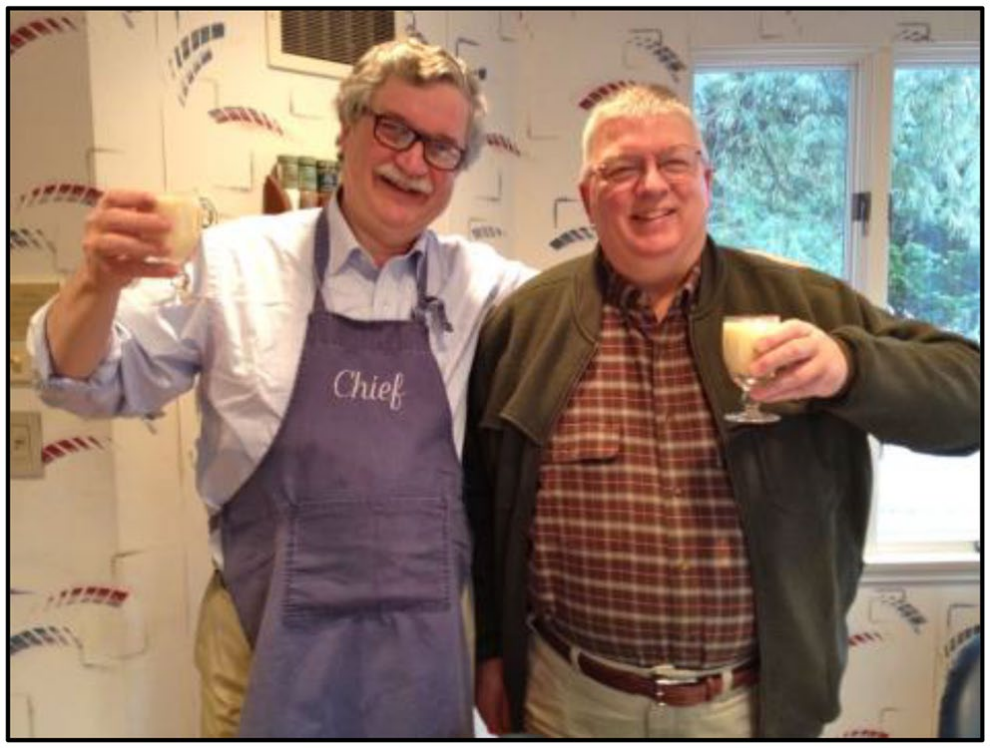
The author of this section (head chef) and Don (sous-chef) at my house enjoying a glass of egg nog prior to preparing Christmas dinner on December 25, 2011

Don was an avid birder who enjoyed sharing his photographs of hawks (Fig. 7) on the introductory slide of his many scientific presentations. He was also an excellent cook, especially of Italian food, and he was a serious wine and bourbon connoisseur. His Christmas present to Carolyn and me was typically a jumbo box containing imported Italian olive oils, quality *balsamico*, spices, jellies, herbs and wines (Fig. 8).

It was a great 36-year collaboration and friendship that started with a chat under a tree on a hot summer day.

### Branched TCA cycle (by Shuyi Zhang)

One of Don’s most significant contributions was the groundbreaking research on the Tricarboxylic Acid (TCA) cycle in cyanobacteria, a study that addressed a long-standing misconception in microbial physiology.

For decades, it was widely accepted that cyanobacteria possess an incomplete or branched TCA cycle due to the absence of 2-oxoglutarate dehydrogenase (2-OGDH), a key enzyme responsible for converting 2-oxoglutarate to succinyl-CoA. However, through meticulous bioinformatics analyses and experimental validation, Don and his student Shuyi Zhang identified novel genes encoding a 2-oxoglutarate decarboxylase (2-OGDC) and succinic semialdehyde dehydrogenase (SSADH) in the cyanobacterium *Synechococcus* 7002. These enzymes effectively replace the function of 2-OGDH and succinyl-CoA synthetase, converting 2-oxoglutarate to succinate.

The study demonstrated that these genes are present in all cyanobacterial genomes, except those of *Prochlorococcus* and marine *Synechococcus* species. Additionally, closely related genes were found in the genomes of some methanogens and anaerobic bacteria, further supporting the presence of alternative pathways in organisms with incomplete TCA cycles.

This study, published in *Science*, fundamentally changed our understanding of the TCA cycle in cyanobacteria (Zhang and Bryant 2011). The findings not only corrected a misconception that persisted for over four decades but also highlighted the importance of re-evaluating established scientific beliefs with new evidence. Don’s unwavering dedication to uncovering the truth and his ability to inspire rigorous scientific inquiry made this achievement possible and left an enduring impact on the field of microbial physiology.

**Fig. 9:**
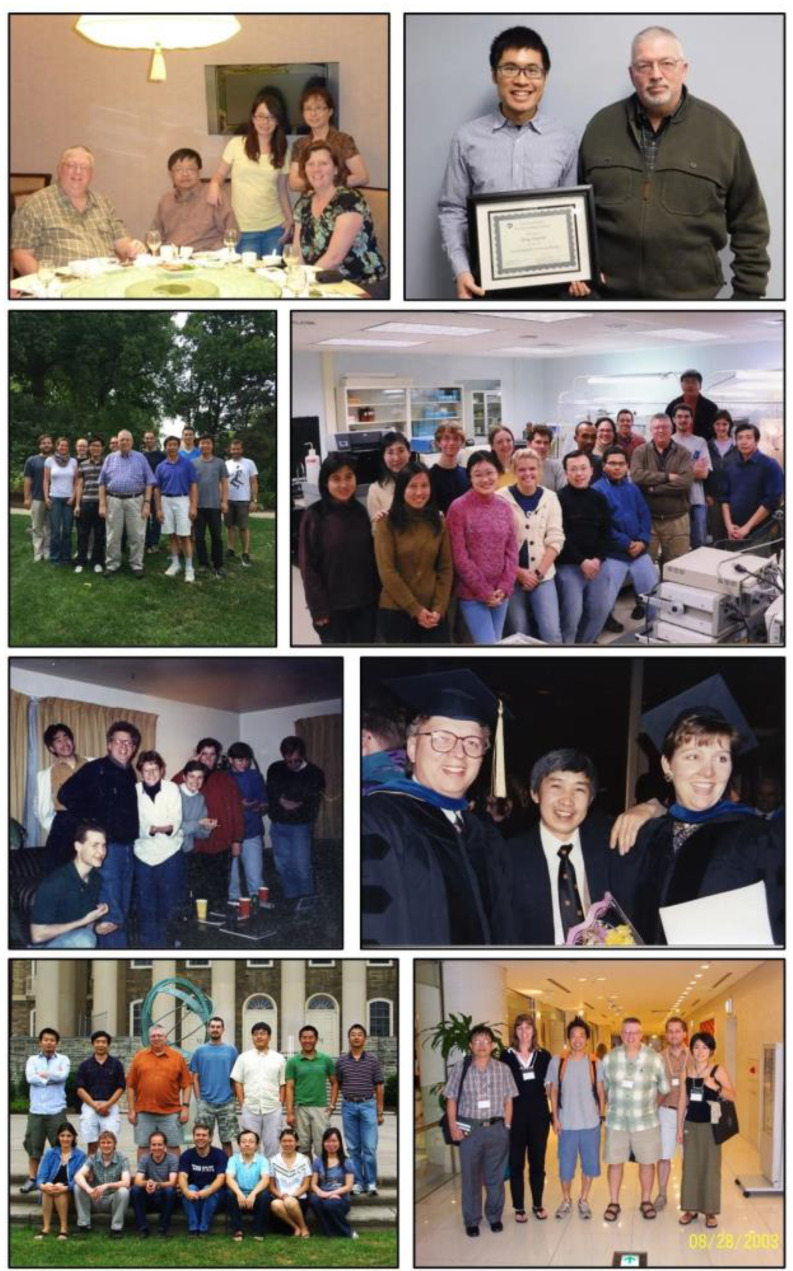
Pictures of Don’s group over the years

## **Prizes and achievements** (by Wendy M. Schluchter)

Don was a very productive scientist, authoring ~ 500 publications and book chapters over his career. Probably one of his most cited works was the book he edited called *The Molecular Biology of Cyanobacteria*, published in 1994. Thus far, his work has been cited more than 33,000 times with an h-index of 101.

During his career in the Department of Biochemistry and Molecular Biology at Penn State as a faculty member, he secured over 23 million dollars in grants as PI or Co-PI, primarily from federal funding agencies. He also had appointments as a Research Professor in the Department of Chemistry and Biochemistry at Montana State University and as a Visiting Professor in the Singapore Centre for Environmental Life Sciences Engineering at Nanyang Technological University, Singapore.

Don accumulated many honors and awards over his career. He graduated with a BS in Chemistry and Biology with honors from the Massachusetts Institute of Technology. He earned his PhD at University of California in Los Angeles, working in the laboratories of Drs. Alexander N. Glazer and Frederick A. Eiserling, supported by a U.S. Public Health Service Pre-doctoral Traineeship. He was awarded an NSF-CNRS postdoctoral fellowship which supported his work at the Institut Pasteur, in Paris, France, studying with Drs. Roger Y. Stanier and Germaine Cohen-Bazire, two preeminent microbiologists. Don was the Ernest C. Pollard Professor of Biotechnology from 1992 until he retired. He was elected a fellow in the American Academy of Microbiology in 1995 and a fellow for the American Advancement for Sciences in 2011. He received the Daniel R. Tershak Memorial Teaching Award in the Department of Biochemistry and Molecular Biology at Penn State in 2010. He was also elected to the Board of Governors of the American Academy of Microbiology, serving from 2012 to 2018. In 2018, he was awarded the D.C. White Award for Interdisciplinary Research and Mentoring by the American Society for Microbiology. This award was created to honor David C. White, known for his interdisciplinary scientific approach and for being a dedicated and inspiring mentor. Don won this award in recognition of his impact on mentoring the 48 graduate students and 38 postdoctoral fellows over his career and on the fact that his approach was always interdisciplinary to understand the biochemistry, physiology and ecology of the many photosynthetic prokaryotes he studied over his career (Fig. 9). In 2020, he received the Charles K. Kettering Award from the American Society for Plant Biologists for excellence in photosynthesis research, and in 2022, he won the Award for Basic Research by the American Society for Microbiology which is meant to recognize an outstanding scientist whose work contributed to advancing our understanding of microbiology.

**Fig. 10:**
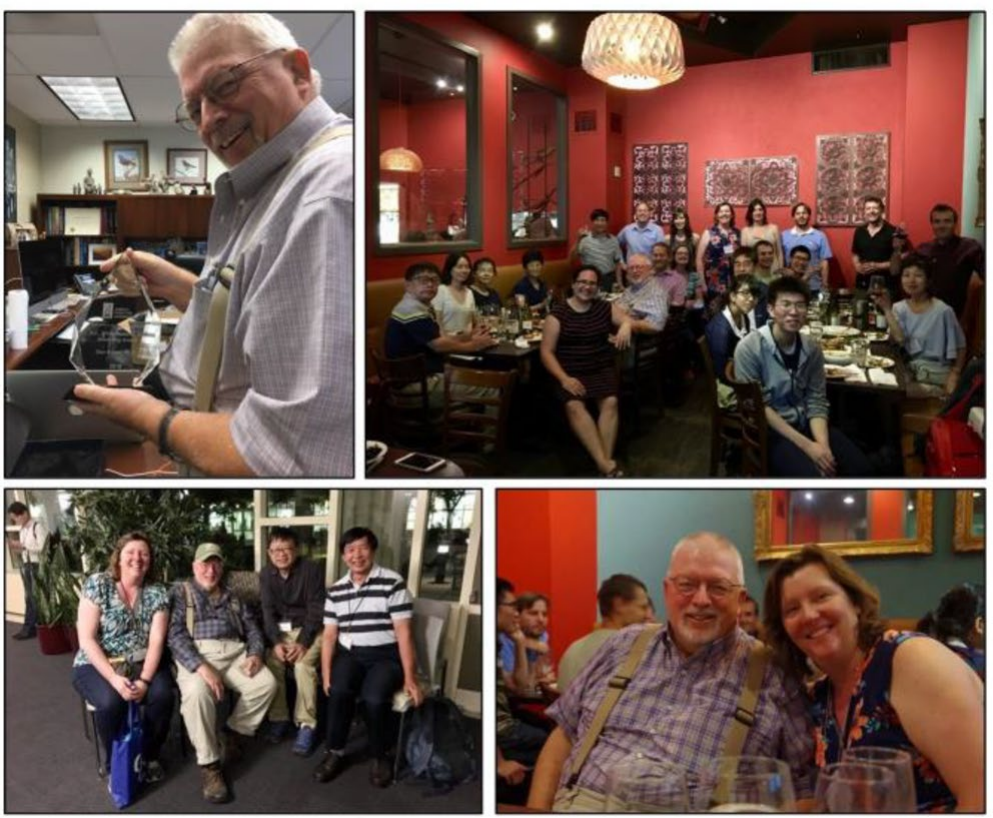
Don with his D.C. White Research and Mentoring award (top left), celebration of Don’s achievements in Vancouver, BC with many former students, postdocs and visiting scientists attending (top and bottom right, 2018), and Don at the cyanobacterial workshop in 2019 (bottom left)

Many of his former students and postdocs gathered in Vancouver in 2018 to celebrate Don’s accomplishments (and the DC White award; Fig. 10). Afterward, Don wrote in an email to Wendy Schluchter and Amaya Garcia Costas: “I always tried to do what I thought was best for my students—even if they didn’t always like it and even if it wasn’t always easy.”

## Reminiscences

*Christopher J. Gisriel (University of Wisconsin-Madison*,* USA)* It seems strange that I only knew Don for about six years. For much of that time, we communicated, one way or another, many times a day. I estimate that we exchanged about 5,000 emails in those six years, mostly in the last 3–4 years. By the time he passed, Don was not only a collaborator and mentor, but a close friend. Don and I first met in Oracle, Arizona at the 27th Western Photosynthesis Conference while I was a postdoc in Petra Fromme’s group. It was toward the beginning of his discoveries in FaRLiP and after seeing his invited presentation, I was heavily intrigued by the topic. That is where Don and I first struck up a collaboration, and soon after he was sending me far-red light photosystem I for cryo-EM studies. I guess Don couldn’t have been a structural biologist in a past life, because I don’t think that field existed before Don was born, but Don was incredibly talented at inferring three dimensional structures of macromolecules from even the most obscure written descriptions. Despite this, he would frequently say “I never had a good sense of orientation with these things”, about which he was incorrect.

At least in the time that I knew him, Don was highly respected, opinionated, passionate, and confrontational. He was a harsh critic and a fierce ally. When speaking to a crowd of scientists, Don was especially captivating. After our friendship matured, I realized that Don was one of the most thoughtful and caring people I had ever met, helping me through some tough times and often asking about my spouse and child. Don had an unwavering passion for photosynthesis and the photosynthesis research community. I remember him one time telling me that a big reason he was so immersed in the field was because he loved its members. I miss Don but am also proud of the science we were able to perform together, and I know I am just one of many that carry his contributions with me to a new generation of scientists.

*Govindjee (University of Illinois Urbana-Champaign*,* USA)* It was in the early 1990s that I had decided to initiate the publication of a series of books on the “Advances in Photosynthesis and Respiration”, for the benefit of scientists in biology, biochemistry and biophysics, interested in doing in-depth research to unravel the steps involved in photosynthesis, from femtoseconds to kiloseconds. It was then that I discovered a brilliant scientist, Donald Ashley Bryant, Professor of Biochemistry and Molecular Biology at the Pennsylvania State University, doing exciting top-of-the-line research on the biochemistry and the molecular biology of cyanobacteria, which included in-depth research on phycobilisomes. What impressed me most was his essay on the puzzles of chloroplast ancestry and his remarkable insight into the biophysics of the coupling of electrons and phonons. On top of that, he had an extraordinary understanding of a key enzyme, the ferredoxin-NADP^+^ oxidoreductase, and the genetics behind it all. Thus, I invited Don to edit the very first book in the Series: Advances in Photosynthesis and Respiration. To my great pleasure he accepted it, and the rest is history. Mohanty (2006) wrote: “The first volume of the series Advances in Photosynthesis and Respiration” ‘The Molecular Biology of Cyanobacteria’, edited by Donald A Bryant, made a thunderous appearance in 1994. It was an instant hit at the ‘target’. Photosynthesis researchers globally were looking for such a book.” This book has 28 top-of-the-line chapters and 48 authors of international repute. Even today, authors of chapters in this book are thankful to him. On January 16, 2025, Akio Murakami, a coauthor of Chap. 22 wrote “We are greatly indebted to Don Bryant for editing and proofreading, in his book, our chapter that dealt with ‘short term and long-term adaptation of the photosynthetic apparatus, particularly the homeostatic properties of thylakoids’. We honor him in our own ways”.

Since Don Bryant was one of the topmost authorities in the world in the area of the molecular biology, biochemistry, biophysics and genetics of oxygenic photosynthesis- especially of cyanobacteria, we are publishing another Tribute to him in India where we discuss how we (and others in India) perceive his research contributions from the 1970s till 2024!

*Scott R. Miller (University of Montana*,* USA)* My first encounter with Don was through *The Molecular Biology of Cyanobacteria*, which he edited. Published while I was a junior grad student with Dick Castenholz at the University of Oregon, the book was somewhat of a sacred text, prominently displayed for years on the end of a bench with back issues of *AEM* and *Journal of Bacteriology*. I spent countless hours with it in the lab, procrastinating, and it played an outsized role in shaping my development as a scientist.

It was only later that I got to know Don himself, first at meetings, then, in the last couple of years, as a collaborator in my graduate student Nikea Ulrich’s investigation of the diversification of *Acaryochloris* light-harvesting systems. Don’s generosity, and that of his group, in welcoming Nikea into his lab was instrumental in taking her research to a new level. Throughout, he was always quick to reply to my many questions, to which he once responded, perhaps too kindly: “Good questions, and no question is from ignorance.” I’m not so sure about that, but I was grateful for his patience and insight. And, like many, I suspect, I still often find myself wondering what Don would think about something we’re currently puzzling over.

The thing about Don that struck me the most was his rare combination of fascination with photosynthetic diversity and his rigorous approach to understanding its underlying genetic basis and biochemical mechanism. This is clear from his group’s body of work, such as the discoveries of Chloracidobacteria and the chlorophyll *f* synthase. Though Don is irreplaceable, his example of fundamental curiosity about bacterial photosynthesis in the natural world points the way forward for the next generation of discoveries.

*Neil Hunter (University of Sheffield*,* UK)* It’s still hard to believe that Don has passed away. We were in the middle of a series of emails last summer about life, science, and the upcoming US election, and then the emails just stopped. So the sense of loss is still very strong. Many people writing these appreciations of Don will be familiar with how the email exchanges went. You would send a brief message asking ‘how are you?’ Then, only a short time afterwards, a very long email would arrive so full of science, life and politics that you hardly knew where to begin replying. How had he typed all that so quickly? But it was wonderful to feel that you had got to know this amazing person and scientist, and in fact Don and I went back a long way. We first met in 1979 when I visited Rod Clayton’s lab with Bob Niederman to carry out some fluorescence experiments on a new light-harvesting complex. In the following years Don and I frequently met at conferences, and we became good friends. The only thing I didn’t look forward to at meetings was following Don as speaker. His talks were so organized, so complete, thorough and incisive, that one’s own talk seemed a bit thin and incomplete. Don was formidable, driven, and intellectually without any peers. He was an awesome scientist, and the best I have encountered, which I did tell him last summer. Because Don could be a bit gruff, a bit short, it was all the more welcome when his underlying warmth came through in conversations. He really cared about his many friends and I’m proud to count myself in that group. Like everyone else, I still really miss him. What a loss to all of us, and to science.

*Toshio Sakamoto (Kanazawa University*,* JP)* In 1994, I was in my first year after receiving my PhD under Norio Murata ‘s supervision and was a JSPS postdoctoral fellow in Masayuki Ohmori ‘s lab. Both Japanese mentors encouraged me to study abroad. So, I had to find a place to study abroad as a postdoc, and at that time Masahiko Ikeuchi recommended to join Don’s lab. I contacted Don and we repeatedly discussed our research plan via e-mail. Don was very responsive and always replied to my e-mails immediately. Don asked me to design my research plan to fit the ongoing projects. I learned that it was a big lab with a wide range of research directions and multiple projects underway and decided to join Don’s lab. I learned from Don the importance of responding to e-mails promptly. Learning from Don’s behavior, I always try to respond promptly to any e-mails sent to me. I first met Don at International Symposium on Photosynthetic Prokaryotes held in Urbino, Italy in 1994, which was the first international conference I attended, before I went to the US.

I visited Don’s lab as a visiting scholar for three months from November 1994 and stayed at the basement of the house that Tanja Gruber shared with other graduate students. There were some memorable moments during this time. I was invited to his home on Thanksgiving Day. I watched the live broadcast of the Rose Ball for the New Year. I attended Wendy Schluchter’s Graduation Ceremony. On January 17, 1995, the day of the Great Hanshin - Awaji Earthquake, ironically Don was on a business trip to Tokyo and I was in the US. The Internet was not as widespread as it is today, and the information available from Japanese news was limited. Don understood the seriousness of the situation and was concerned about me and my family. Thanks to Soohee Chung ‘s assistance, I was able to find a place to live with my wife Kaori Inoue-Sakamoto starting from April 1995. “If you help someone, someone else will help you.” Helping each other is a common thing, and Don would say occasionally. International students who are treated with care while studying abroad return to their home countries with a feeling of love for the country. I always treat the international students who visit my lab with the same respect that Don and the lab members treated me.

As for Don’s style of running his lab, Don didn’t mind that Chris Nomura and his fellow students meet to discuss things other than research in the lab. Don said to me, “It’s my role to make the lab a comfortable place. Chris spends a lot of time in the lab, not just doing research. People don’t stay in a place they don’t like.” This is what I learned about lab management from Don. There were no group meetings nor journal clubs, and research was done on one’s own responsibility. In this research environment, I always visited Don ‘s office to discuss my research results. The door was always open, and he was always available to discuss with me. It was a very comfortable research environment for me. I was originally scheduled to stay for two years, but I ended up staying for almost six years, learning a lot from Don ‘s lab, until I got a job at Kanazawa University in 2000.

The last time I saw Don was at ISPP in Vancouver, Canada in 2018. We didn’t have much time to talk, so I e-mailed him after I got back home. He gave me a detailed reply about his nearly fatal battle with cancer. “Life is short. Make fun of it! " This is the words attached to Don ‘s e-mail during a certain period from around 2000. I know that hard times won’t last forever, but the perspective of others gives people the courage to face difficulties. When the Noto Peninsula earthquake occurred on January 1, 2024, I received an email from Don inquiring about the situation. Even now that he has passed away, I still dream that I might receive a reply to the e-mail I send to dab14@psu.edu. The heavy, wet snow in Kanazawa reminds me of the weather in Pennsylvania, when the harsh winter is over, and spring is approaching.

*Shuyi Zhang (Tsinghua University*,* CN)* Reflecting on my time with Don, I am filled with profound gratitude and admiration for my Ph.D. advisor, whose mentorship has deeply influenced my academic and professional journey.

My journey with Don began in the fall of 2009 when I joined his lab for a rotation study. The depth of his knowledge and the effectiveness of his training were immediately apparent. His innovative teaching methods and conceptual views on bacteria and microbiology revolutionized my understanding of the subject, leaving an indelible mark on my academic perspective. He encouraged me to take his undergraduate course, Microbial Physiology and Structure, which proved instrumental in my subsequent research on cyanobacteria metabolic engineering. His ability to seamlessly integrate traditional microbiology with cutting-edge scientific discoveries created a dynamic learning environment that broadened my knowledge and fueled my curiosity.

In the lab, Don’s mentorship was equally impactful. Although he was on sabbatical shortly after I joined, his guidance and support were never on sabbatical. Even from afar, he led me to groundbreaking discoveries about the TCA cycle in cyanobacteria, resulting in textbook-changing results published in *Science*. I still vividly remember our phone call where we discussed expressing the new TCA cycle genes in *Synechococcus* 7002, a conversation that took place 15 years ago (2010). Don’s meticulous bioinformatics analyses and insightful hypotheses were a testament to his brilliance and dedication. Upon his return, Don’s open-door policy and collaborative spirit fostered an environment of continuous learning and growth. Don’s support extended beyond research. He provided me with the opportunity to mentor other students, imparting valuable lessons on effective mentorship. His belief in my potential was instrumental in securing prestigious awards and fellowships, propelling my academic career forward.

Don officially retired on August 31, 2022, marking the exact 50th anniversary of his research on cyanobacteria and 41 years of dedicated service at Penn State—both milestones he began on September 1 (in 1972 and 1981, respectively). Even in retirement, Don’s commitment to science remains unwavering. He continues to contribute to the field through collaborations and publications. His recognition with the Kettering Prize for photosynthesis research is a testament to his outstanding contributions and the respect he commands in the scientific community.

Don’s legacy is one of excellence, curiosity, and mentorship. His impact on the field of microbiology and the lives of his students is profound and lasting. It is with the utmost respect and gratitude that I celebrate his remarkable career and enduring influence. Thank you, Don, for being an extraordinary mentor, an inspiring scientist, and a cherished advisor. Your spirit and dedication will continue to inspire generations to come.

*Wendy M. Schluchter (University of New Orleans*,* USA)* I was Don’s PhD student at Penn State from 1989 to 1994. I did my rotation through Don’s lab while he was on sabbatical in Switzerland, and I always joked to him that it was his wife, Vicki, a technician in his lab, who recruited me. My research project involved the cloning and characterization of Photosystem I genes in cyanobacteria. My first project involved the cloning and sequencing of *petH*, a gene encoding the ferredoxin NADP reductase (FNR). We realized that the ORF for this FNR had an N-terminal domain that was similar to CpcD, a linker for the PBS. In order to prove that the protein was larger in many cyanobacterial cells, I did a Western Blot using antibodies against spinach FNR. I will never forget Don and I hunched over that Western, late on a Friday afternoon, watching it develop and seeing that all cyanobacterial extracts we tested did have an FNR that was much larger than the spinach enzyme and that FNR was a component of purified PBS in cyanobacteria. Don was grinning ear to ear! Don had that habit of knowing when someone in the lab was doing a key experiment and casually walking by to check on the results. I just remember that his hunches about how things worked were usually right. His intellect and intuition about science were unsurpassed.

Don had many hobbies such as cooking, music, birding (especially raptors; see Fig. 7), and traveling. Just as he was with his science, he was very intense about his hobbies. If he was interested in something, he was “all in” and became an expert on that thing. This intensity about science or his other hobbies often intimidated others. However, once you got him talking about science or his beloved raptors, for example, his sense of humor might be revealed, putting people more at ease.

I stayed in academia and started my own lab, studying the biosynthesis of the light-harvesting complex in cyanobacteria. We continued to collaborate on and off, throughout my career. I was lucky enough to see him often at conferences. I cannot even express how much his mentorship meant to me or my career. He was very generous, offering to take me and my graduate student into his lab after Hurricane Katrina closed my lab for several months in 2005, and I spent a sabbatical in his lab in 2008. I knew a side of Don that maybe not all his former students did because of my friendship with Vicki. He attended my wedding in 2003. He was most proud of his former mentees’ accomplishments. I saw evidence of this many times (including in his CV, see Fig. S1), but I realized how much he kept track of all of us recently. Just after he had passed away, I was helping his executor organize his birding photos on his computer to facilitate his wish to donate them to a local birding organization. Don had folders for almost every graduate student and postdoctoral fellow who came through his lab. He would save every picture sent to him by each former mentee of their kids or a trip that they went on and articles they published after they had left the lab. When I had a cool result in the lab, he was one of the first I would want to share that with. I am really going to miss him, and I am truly at a loss to express my profound gratitude for all he did for me and for his massive contributions to the field of photosynthesis. Rest easy, Don. I like to think your spirit is out there with your favorite raptors, gliding on thermals.

*Conrad W. Mullineaux (Queen Mary University of London*,* UK)* I think I first met Don at a legendary light-harvesting satellite meeting in Sanda, Japan, in 1992, where we got talking about phycobilisomes. This probably happened in or near the communal hot bath. I was a research fellow in Sheffield at the time, and I was flattered that Don took an interest in some results I had that indicated that phycobilisomes could act as light-harvesting for Photosystem I as well as Photosystem II. Don had found a mutant phenotype that suggested the same thing, and he saw some interesting connections. The contact eventually led to a generous open-ended offer to spend some time in his lab in Penn State. Arguably, I owe my whole career to that offer. I ran out of funding in Sheffield at the end of 1994 and looked to be out of any long-term options to continue an academic career. However, whilst working in Daresbury on rat testicles I was lucky enough to get an interview for a lecturer position at University College London – the only catch was that the appointee was supposed to teach molecular biology, a subject about which I was completely ignorant at the time. I was able to reassure the panel that I had an option to visit Penn State, where Don would teach me all about molecular biology. On the strength of that, I was offered the UCL position. Don was as good as his word, which was typical of his generosity to younger scientists. I went to Penn State in the summer of 1995 and started the UCL job in January 1996. The stay in Penn State was a great experience. I certainly learnt a lot about molecular biology and much else. I am not sure that Don ever got much return from it, but he never complained. He bravely hosted my young family in his house at the start of the trip, and he tolerated the chaos for about a week before working hard to ensure that we found somewhere else to stay longer term. Later I came down with a nasty facial erysipelas infection, and I well remember Don’s reaction when I phoned him to explain: “Conrad, it’s too bad you didn’t get it sooner!”. His logic was that he had already dealt with the Gram-positive bacteria in his lecture course. If I could just have got infected in a more timely manner I could have been a fantastic walk-on exhibit. Don was a giant in our field, with a unique breadth of knowledge. But, beyond that, I think that he thought of anyone working on phototrophic prokaryotes as family. We didn’t always agree, we didn’t always get on, but we were family and deserved whatever support he could give. It would be a great memorial to Don if we can carry that spirit forward in our research community.

*Christopher Falzone and Juliette Lecomte (Johns Hopkins University*,* USA)* “Look deep into nature and you will understand everything better.” In the early nineties, Chris and I were members of the chemistry department at the Pennsylvania State University. Our training as protein NMR spectroscopists caught Don’s attention. He approached us with a simple problem. Would we be able to solve the structure of a polypeptide found in the stromal cap of a microbial photosystem I? He would supply ample quantities of purified and ^15^N-labeled protein. Who could refuse such an offer? Photosystem I accessory protein E from *Synechococcus* 7002 thus became our entry into Don’s fascinating world of cyanobacteria and photosynthesis. Followed decades of steady friendship, occasional publications, and continuous scientific exchanges.

**Fig. 11:**
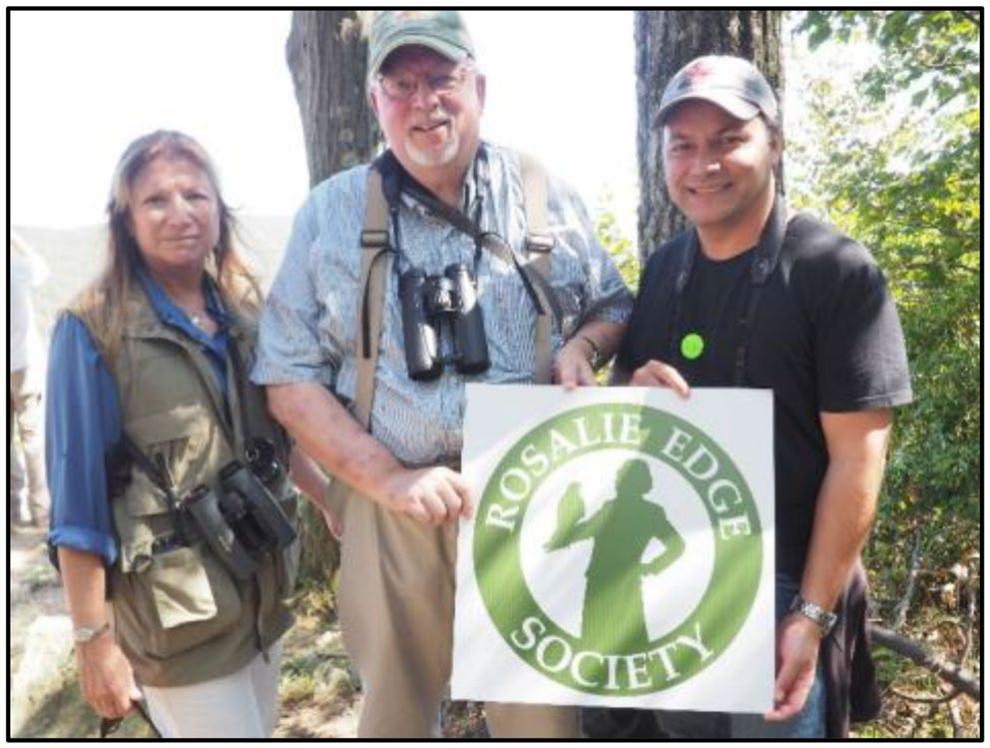
Don (center), in 2019 on the day of his induction in The Rosalie Edge Society, of the Hawk Mountain Global Raptor Conservation, with Laurie Goodrich (left) and Sergio Seipke (right)

“Dans les champs de l’observation le hasard ne favorise que les esprits préparés.” All of Don’s coworkers and students are familiar with his stamina and talent for noticing what few others would. His skills of observation and openness to the unexpected were also on display as a bird-of-prey expert and wild-life photographer. Don traveled the world to sight all manner of raptors and big cats. He shared astonishing pictures with us and, during trips to Pennsylvania, Maryland, and Montana, taught us how to identify eagles and hawks. He was so attuned to avian habits and territories that from year to year he would recognize individual birds in the locations he visited. Spotting rare species, spending time with fellow bird watchers in-person (Fig. 11) or online, and sorting through photographs from his many expeditions were uplifting activities he maintained to the end. The raptor community too mourns his loss.

“Thankfully, persistence is a great substitute for talent.” In a famous and apt autocorrection of his name, Don Bryant was known to many as Bon Vivant. He had the same drive for perfecting a recipe as he did for mapping the biosynthesis of modified tetrapyrroles. Be it for authentic amatriciana sauce or kung pao chicken, he would have the palate of a food critic and the patience of an aspiring chef to get things just right. His culinary prowess shone on many memorable dinners. He was a prolific scholar, yet the bon vivant in him managed to share in the good times of others and to persist in their bad times as well.

“Messieurs, c’est les microbes qui auront le dernier mot…” After our work with PsaE, we engaged in several projects with him. Each, whether successful or not, brought opportunities for new musing on microbial physiology and bioenergetics. To work or simply interact with Don was to commit to rapid-fire exchanges, at once exacting and exhilarating. His profound knowledge of biochemistry and microbiology guided our own work. He was a generous mentor and a constant presence, dispensing daily commentaries on chlorophylls and xanthophylls, birds and bears, arts and crafts, weather, wine, and politics…, but the microbes always had the last word.

The readers who have communicated with Don over the years have recognized some of his email signatures in quotes. For us, his subtext remains an exhortation–“Life is short. Make fun of it!”

**Fig. 12:**
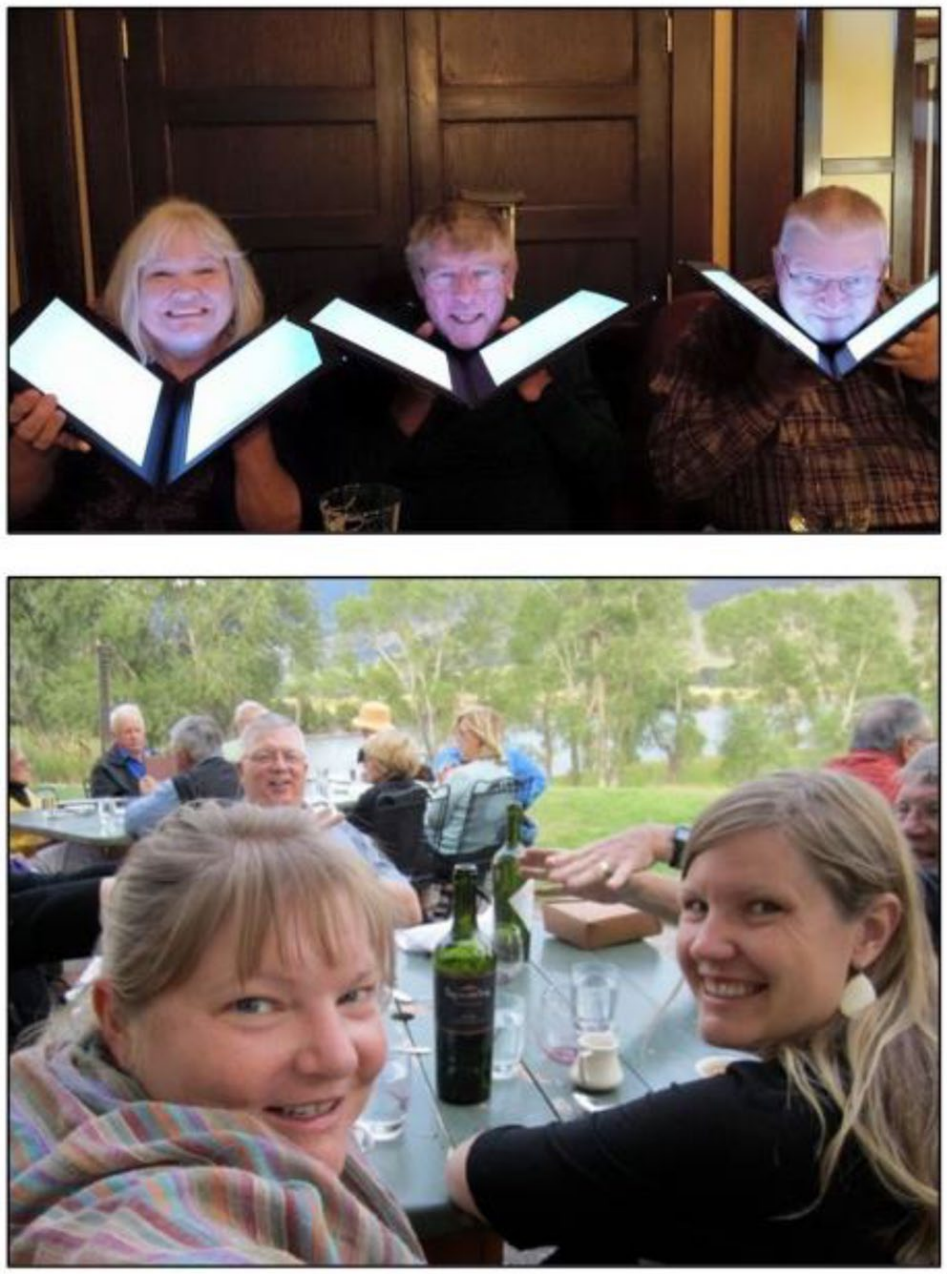
Nancy, Dave, and Don at “The Sac”; Molly, Don and Abby at a restaurant along the Yellowstone River

*Dave Ward (Montana State University*,* USA)* Our 20-year collaboration drew Don to Bozeman many times (Fig. 12), sometimes for extended periods. He loved to view and photograph the raptors of The Gallatin Valley where I live. This presented many opportunities to get to know him personally. We enjoyed getting together for whisky and conversation with Montana State University colleagues. My wife Nancy, our daughters Molly and Abby, and even neighbors included Don whenever we could. I guess we kind of adopted him and he was grateful for that. My daughters even called him Unca Don. We spent many evenings on our back deck enjoying each other’s company, playing Scrabble, and laughing at Seinfeld jokes. Once, Don participated in a talk I gave in which I had promised to include three lines from that TV show. He sat in the middle of the audience and raised his hand with 1, 2 and 3 fingers to account for each line I delivered. We were just trying to add a bit of humor to the science we were reporting. Don was very generous to us. Each Christmas, he gave us a whopper gift certificate so that we could enjoy dinner at one of his favorite restaurants, The Sacajawea Inn in Three Forks, MT. He always remembered our birthdays. I enjoyed the numerous phone calls we had, which always led to a lot of laughter. After Don’s health forced him to stop travelling, I was able to visit him on several occasions in his comfortable niche atop the Pennsylvania mountains near Happy Valley. During my last visit, Don told me that I was one of a few colleagues that had significantly influenced his career. I was honored and asked him why. Don answered that it was because our collaboration had refocused his view of microbiology to a more natural one than the view he and I had learned during our training. It is pleasant to think of Don’s ashes being up there where he watched migrating raptors. I sure miss the scientific interactions and friendship we had.

*Gaozhong Shen (University of Wisconsin-Madison*,* USA)* I had a long phone conversation with Don last July (also my last chance hearing Don’s voice), mostly discussing about my planning to join in the Gisriel lab in the University of Wisconsin. Don told me that he was doing well with his health but had to take some medicine for his abdominal pain. Don discussed with me about a couple of manuscripts that were almost ready to submit, and more good research results he planned to put into new manuscript drafts (those unfinished manuscripts’ titles are listed in Don’s 2023 version CV). Mostly Don suggested more research experiments to continue after joining the Gisriel lab.

I first met Don at the 1993 Cyanobacteria workshop in Asilomar when I was studying for my Ph.D. in Dr. Wim Vermaas’ lab. Don liked my poster presentation on characterization of a *Synechocystis* 6803 mutant with deletion of the *apcE* gene and deficiency of photosystem I. During our first conversation, I asked Don for a postdoc opportunity. So, after I graduated from Dr. Vermaas’ lab, I joined Don’s lab in early 1994. Since then, I worked with Don even after he closed his lab in 2022 - that’s almost 30 years. Although my employment titles changed many times for me, discussion on research projects and consultation on experimental designing and problem solving were always the same in a pleasant and productive atmosphere for years. I never had any doubt in my mind because of my respect for Don’s talent, knowledge, achievements, and friendship. I feel very lucky in my life working with Don, with the opportunity of doing what I have loved: research and developing my scientific knowledge and skills. I believe that I have enjoyed every new discovery in conducting research experiments and every moment in working together with Don and others around me in the group. I will never forget that, with Don’s protein fragmentation suggestion by formic acid, I have learned and achieved very neat and convincing experimental results in identification of the new bilin lyase genes. In collaborating with other groups in genomic sequencing of several newly isolated cyanobacterial strains, a special gene cluster composed of genes encoding different PSI, PSII, PBS and regulatory proteins in *Leptolyngbya* JSC-1 and *Ficherella thermalis* JSC-1 had caught Don’s eye. For exploring the gene expression of those genes, I really believe that Don’s idea to achieve the far-red light illumination through combination of the red and green filters is so bright for our archiving in discovery of far-red acclimation (FaRLiP) in cyanobacteria. Working with Don, I have learned the importance of collaboration in research projects for teaming up with others, which is simply reflected in the coauthor list of Don’s publications.

*Tanja Gruber (Pennsylvania*,* USA)* I first met Don as an incoming graduate student when I did a rotation in his lab. Coming from Austria, it was initially difficult for me to call my professor by his first name, but Don cured me of that quickly! Even though I never imagined myself in a photosynthesis lab, Don’s infectious enthusiasm and impressive intellect, coupled with the amazing lab members, made it an easy decision to join his lab.

In Don’s lab, I gained the independence to explore new areas and approaches while always being guided when needed. My thesis focused on the genetics, biochemistry, and evolution of sigma factors in cyanobacteria and other photosynthetic bacteria. One of the main areas of focus was cloning and determining the function of sigma factors in *Synechococcus* 7002. Don was always the most excited when it seemed like a new sigma factor had been cloned. He would eagerly wait outside the darkroom while I was developing an ^35^S-labeled sequencing gel. As soon as I had the autoradiograph ready, Don would rip it from my hands, rush to the lightbox, and translate the nucleotide sequence in his head. He could tell right away whether or not we had discovered something new. I have always been impressed by his AI-like processing speed!

Don was incredibly productive, and publishing papers was a way of life for him. But beyond just the work, he instilled a sense of rigor in his students and lab members. I’m proud to say that not a single one of my research papers required additional experiments to get published—a fact that my scientist husband remains very envious of! Among all the papers I’ve published, the one I’m still most proud of is my first paper from the Bryant lab.

**Fig. 13:**
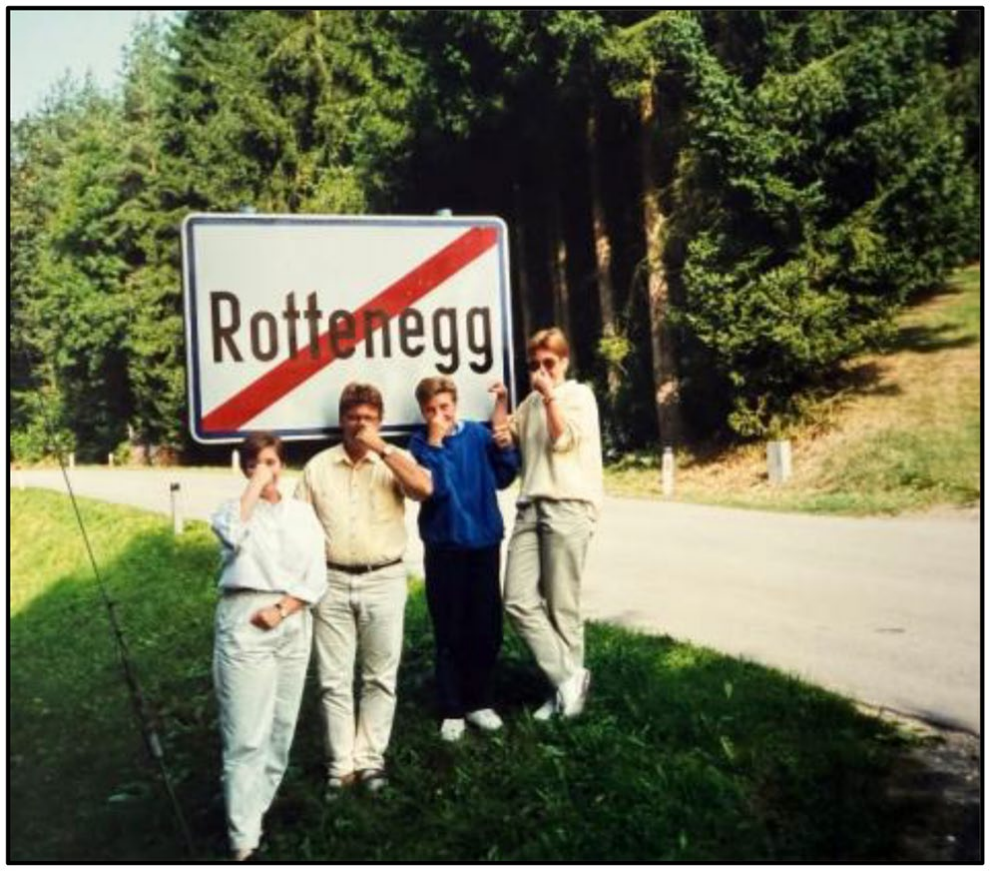
Don would never let Tanja forget that the name of her hometown is “Rottenegg”

The five or so years I spent in Don’s lab were some of the most enjoyable of my career. We all worked hard, but we also made time for fun. Don was a fiercely competitive athlete, and we spent many hours on the basketball and tennis courts. A fond memory is when several lab members went to a meeting in Urbino, Italy, and we took a detour to Austria. My parents were thrilled to meet Don during that visit. He would never let me forget that the name of my hometown is ‘Rottenegg’ (Fig. 13).

When Don was on sabbatical in Australia, I had the pleasure of pet-sitting for him. His house was perched on top of a ridge, and the road to get there was quite treacherous in the snow and ice. I’d receive emails and texts from Don from literally the other side of the world, reminding me to get home before a snowstorm hit. It was clear he was concerned for my safety, but it also reflected his obsession with the weather!

Don was a ‘Doktorvater’ in the truest sense of the word. His mentorship went far beyond academic guidance, and I will always be grateful for the time I spent in his lab and for all the lessons he taught me—both in science and in life.

*Niels-Ulrik Frigaard (University of Copenhagen*,* Denmark)* I joined Don’s lab in 1999 as a postdoc because he was working with green sulfur bacteria, and I wanted to learn molecular biology. Don was obviously famous for his work on cyanobacteria, but ironically, I never worked with cyanobacteria while in his lab. As a PhD student in Odense, I had worked on the pigments and chlorosomes of green sulfur bacteria, but I had no experience whatsoever with molecular biology. Even so, Don generously invited me to his lab. At the time, he was involved in the genome sequencing of *Chlorobium tepidum*, and I was eager to be part of it. Honestly, the field of green sulfur bacterial physiology was (and is) not large, and I have always had to work hard to explain to others why these organisms are interesting. But not to Don. He was excited about anything related to photosynthesis and microbes, and he had an exceptional instinct for recognizing ideas worth pursuing. He once told me how he got into green sulfur bacteria. He was at a conference where two scientists got into a heated debate about chlorosomes. After that, he figured that this field must be worth going into. Several years later, when I had started in his lab, I clearly remember him being extremely happy when we managed to knock out bacteriochlorophyll *c* biosynthesis in *Chlorobium*, because when he saw the first orange-colored colonies of the mutant on the agar plate, he could immediately envision all the exciting experiments and papers that would follow from this. Don was full of ideas and knowledge that he happily shared. He was uncompromising in his science and driven by curiosity and intuition. He had a rare ability to distinguish between what was interesting and what was not, and he inspired others to do the same. I have tried to follow his approach to science ever since.

*Yumiko Sakuragi (University of Copenhagen*,* DK)* It was the summer of 1998 at a conference in Vienna when I first met Don. I stood in front of my poster, nervously presenting, while Don stood silently—arms crossed, wearing that classic grumpy, questioning look. Don didn’t say much at first, and I was growing very nervous. But the moment I shared my intention—to do a PhD with him—his face lit up. He smiled wide, laughed heartily, and welcomed me into the conversation.

Since that day, Don was more than a mentor. He became my science father, my inspiration. Don gave me so much - sharp critiques, encouragements, and most of all, belief in me.

I remember the day before my PhD defense. I was incredibly nervous and was in full doubt about if I was truly prepared. In panic, I started reading up on the most basic things - like hydrogen bond distances, of all things. That’s when Don passed by my desk and said to me: “*you are incredibly smart - but sometimes*,* you are incredibly dumb*”.

Naturally, I only heard the dumb part and ruminated on it, fuming. But in doing so, I forgot all about being nervous and reading hydrogen bonds - and I defended my thesis just fine the next day.

Don was right, of course. I do sometimes get side-tracked by things that are secondary or even tertiary. I still remember that moment, and it still makes me laugh - and it helps me focus in many situations.

I miss Don. I thank him for everything—for the encouragement, the challenges, the inspirations, and for simply being there. I know that Don is no longer here physically, but he is with us. He is with me.

*Robert Blankenship (Washington University in St. Louis*,* USA)* I first met Don Bryant in 1984 at the very first Eastern Regional Photosynthesis Conference, held at Woods Hole, Massachusetts. We were both young assistant professors at the time. He came from a microbiology background and I came from a chemistry background, so we approached questions in somewhat different ways, but had significant overlap in interests. We became good friends over the years, yet only started working together in 2010, first with a series of papers on chlorosomes and FMO proteins from the green sulfur bacteria, and later some papers on far red-utilizing cyanobacteria. Don was an enthusiastic member of the Photosynthetic Antenna Research Center, and it was always fun to see him at the yearly All-Hands meetings for PARC in St. Louis.

**Fig. 14:**
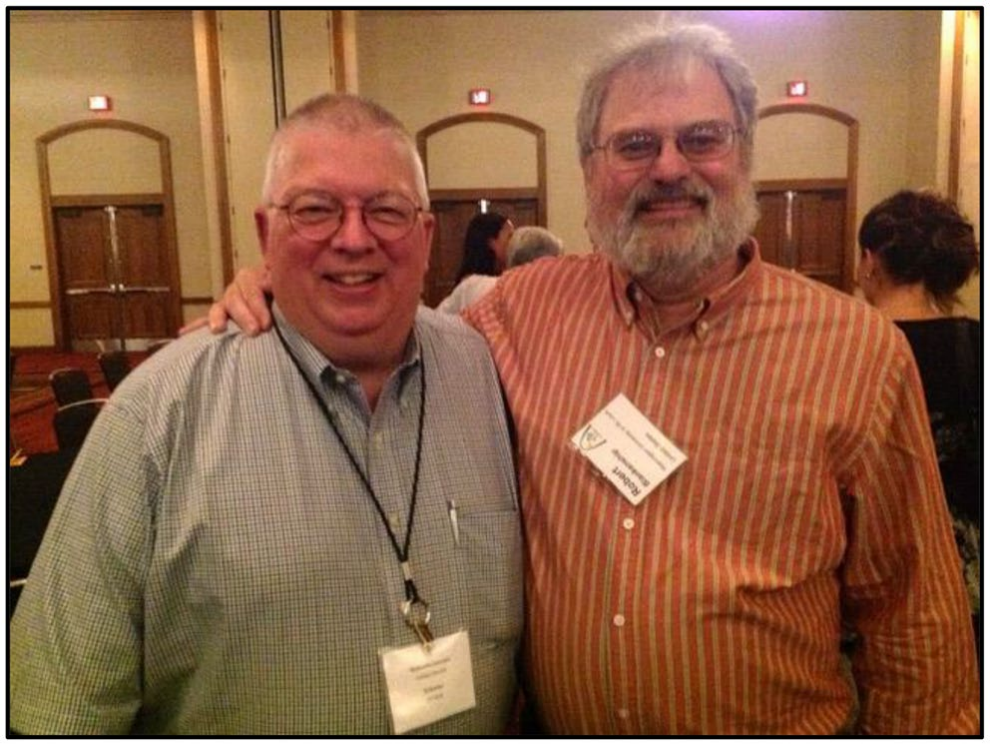
Don Bryant and Bob Blankenship at the 16th International Congress on Photosynthesis Research, held in St. Louis, Missouri in 2013. Photo courtesy of Bob Blankenship

Don was a giant in the field of photosynthesis. His first love was cyanobacteria, and he studied them in incredible detail for his entire academic life. Later he worked on green sulfur bacteria with equal enthusiasm. He also had a special place for thermophiles in his heart. It was a pleasure to count myself as a close friend and collaborator and I miss him tremendously (Fig. 14).

*Gary W. Brudvig (Yale University*,* USA)* I first met Don at a Photosynthesis Gordon Research Conference in 1981. This was my first photosynthesis conference, and I knew very few of the participants. With our common background, having both worked at Berkeley, Don and I connected at this first meeting. I got to know Don well at the Eastern Regional Photosynthesis Conferences (ERPCs) that have been held each spring at Woods Hole, MA since 1984. Don and I both attended the ERPC annually for many years. I much enjoyed our discussions during these meetings. Don’s knowledge and contributions to our understanding of photosynthesis, especially green sulfur bacteria and cyanobacteria, biliproteins, biosynthetic pathways, and PSI are amazing. I always found his contributions to our discussions incredibly insightful. Don and I finally had the opportunity to collaborate when Chris Gisriel joined my group at Yale University as a postdoc. Prior to moving to Yale, Chris and Don had started a collaboration to use cryogenic electron microscopy (cryo-EM) to determine the structure of far-red light (FRL) acclimated PSI. Chris continued this work at Yale, expanding the effort to include cryo-EM structures of not only PSI but also PSII and biliproteins expressed during FRL acclimation and low-light adaptation. This proved to be a tremendously successful and productive collaboration that resulted in 12 joint publications. Chris also organized a series of Zoom meetings with many of the leading investigators working on this topic. Don regularly attended. It was inspiring to meet during these meetings and to work together. Don Bryant’s untimely passing is a sad conclusion to a tremendous career. However, his legacy lives on with his many contributions to the field of photosynthesis.

*Amaya Garcia Costas (Colorado State University-Pueblo*,* USA)* The first time I met Don was during the Microbial Diversity course at the Marine Biological Laboratory in Woods Hole, MA. He gave two one-hour lectures back-to-back covering everything from electron microscopy of chlorosomes, bacteriochlorophyll biosynthetic pathways, evolution of the reaction centers, genetics of cyanobacteria, genomics of green sulfur bacteria and more. At some point he asked the class “do you know how many proteins *E. coli* can make?” We didn’t, but we knew we were about to learn (about 4,000 different ones based on the number of genes; about 5 million total based on abundance). The rest of the students were a bit overwhelmed, if not stunned, by the encyclopedic information, but I loved it. I had no idea that day that Don, besides being inspiring, was also going to change my life.

He hired me as a lab technician the next year. I came with a Master’s degree, a husband, and two young daughters. Having spent the previous years as a stay-at-home mom with an occasional adjunct stint, I had so little to offer, but Don took me in and gave me a couple of projects to work on without questioning my very non-traditional background. One of the projects involved isolating a new phototroph from mat samples from Yellowstone Park (*Chloracidobacterium thermophilum*). When the phototroph started growing at significant levels, Don took me in as a graduate student to further study it and helped me get a PhD in the most unorthodox way: I spent three years in the lab, and then commuted from Montana in the summers, as I finished bioinformatic analyses and thesis writing. Again, never did Don remotely even once lament how much easier it would be to have a traditional student work on this project. He was fully supportive. But, if Don didn’t mind my personal life, he did mind my data. Once, I showed him a draft table of bacteriochlorophyll biosynthesis genes from this new phototroph, and I missed a few of the core steps in the pathway. Here, there was no grace bestowed. I was shamed and reminded in no uncertain terms to not send him unfinished or drafts of anything. A day like that would be difficult, of course, but it also meant that, once my heart rate would return to normal, I had to believe in myself to be capable to do a task well; Don clearly expected me to, so why wouldn’t I?

Don came to my rescue once again years later. He was back in Montana doing a sabbatical, and I was switching postdocs. Again, he took me in. Without the benefit of a full lab, I had Don’s nearly full attention and enjoyed chats on anything from politics to raptors of course, to trips he was taking, and to every project and publication that he was working on at the time. That year I was applying for faculty positions and, after every phone interview, Don would go over the questions with me and coach me for the next one, again building confidence. I did get a job and was also able to finish a paper with Don during my first two years of teaching. But I wasn’t able to do others as the demands of my new position increased, even more once the pandemic started. I am not sure that Don ever forgave me for that.

I have many images in my mind of Don in the lab and in conferences. I will finish with just two of them: in one he is hunched over in his office, going over one of the 50 + manuscripts that he would review each year, in the other he is at ASM, walking around with his buddy Dave Ward, smile from ear to ear as he would recall the highlights of whatever talk he had just attended. Rest in peace, Don. I will always be grateful for the many opportunities that you gave me, I will always be inspired by your commitment to excellence.

*Dan Canniffe (University of Liverpool*,* UK)* I first met Don at a tetrapyrrole meeting in Monterey in 2009, when I was a PhD student in Neil Hunter’s lab. I was working on chlorophyll biosynthesis in cyanobacteria, so had read many of Don’s papers and knew of his fierce reputation. I was initially filled with dread when he came to speak to me at my poster, but was relieved to find that Don was much more conversational than confrontational. Later that evening, Don gave an engrossing talk on his group’s efforts to make *Synechococcus* 7002 a synthetic biology platform organism – Don had no time for waffle, using short statements without a wasted word to describe his data so that everyone in the audience could follow. At the end of the talk, I turned to Neil who puffed out his cheeks and remarked, “power science” – a phrase I heard him affectionately use on many occasions afterwards to describe Don’s work – and I was in awe.

A few years later I was considering moving to the US as a postdoc. Don’s lab would have been the perfect fit for me, but being a city boy, I was put off moving to Penn State after discovering its rural location. In 2014, with this move in mind, I had written a prospective fellowship proposal that Neil took with him to a US Department of Energy meeting, where I had asked him to share it with colleagues who might be interested in supporting my application. On the evening that Neil arrived, I received a long email from Don (which turned out to be one of Don’s shorter emails) outlining why I should join his group and why I had State College all wrong – you didn’t say “no” to Don, so it was decided (and a great decision it was).

I moved to Penn State in 2015, a few days after Don’s cancer diagnosis. He was to start chemotherapy a couple of weeks later. From that point, he only came to campus on Wednesday afternoons for our group meetings. He looked increasingly gaunt each week as his treatment progressed, but despite how he must have been feeling, our meetings were lively affairs; hearing what was going on in the lab and debating interpretations of our results were keeping his spirits up. At the time, around half of the group were working on aspects of far-red light photoacclimation, discovered by Don and published the previous year. Don went home excited the day that Ming-Yang Ho discovered that his mutant in *psbA4*, encoding the ‘super-rogue’ D1, did not produce chlorophyll *f* but still produced chlorophyll *d* when cultured under far-red light. The following day he shared a manuscript with us, written for *Science* in one evening, despite us only having this preliminary data. Over the next couple of months we were able to collect the data that Don expected we would, proving that PsbA4 was a photooxidoreductase that could synthesise chlorophyll *f* in a heterologous system; Don’s prescience and work ethic were remarkable – this is just one example from a career full of them.

Since returning to the UK and starting my own group, I continued to receive inexhaustible and valuable support from Don. Whenever I had a question – be it chemistry, microbial physiology, or US politics – a long, insightful answer would follow, almost instantaneously. Often it appeared that Don was faster than Google. Along with his detailed replies would come updates about other former members of his lab and collaborators, whom he made great efforts to stay in contact with. Despite his sometimes hard reputation, Don was a caring mentor, colleague, and friend – if you were on Don’s team, he was on yours. Being supported by and working with a true polymath like Don was a privilege, his way of thinking could not help but broaden your horizons, and his influence on me and generations of mentees is immeasurable – he is truly missed.

**Fig. 15:**
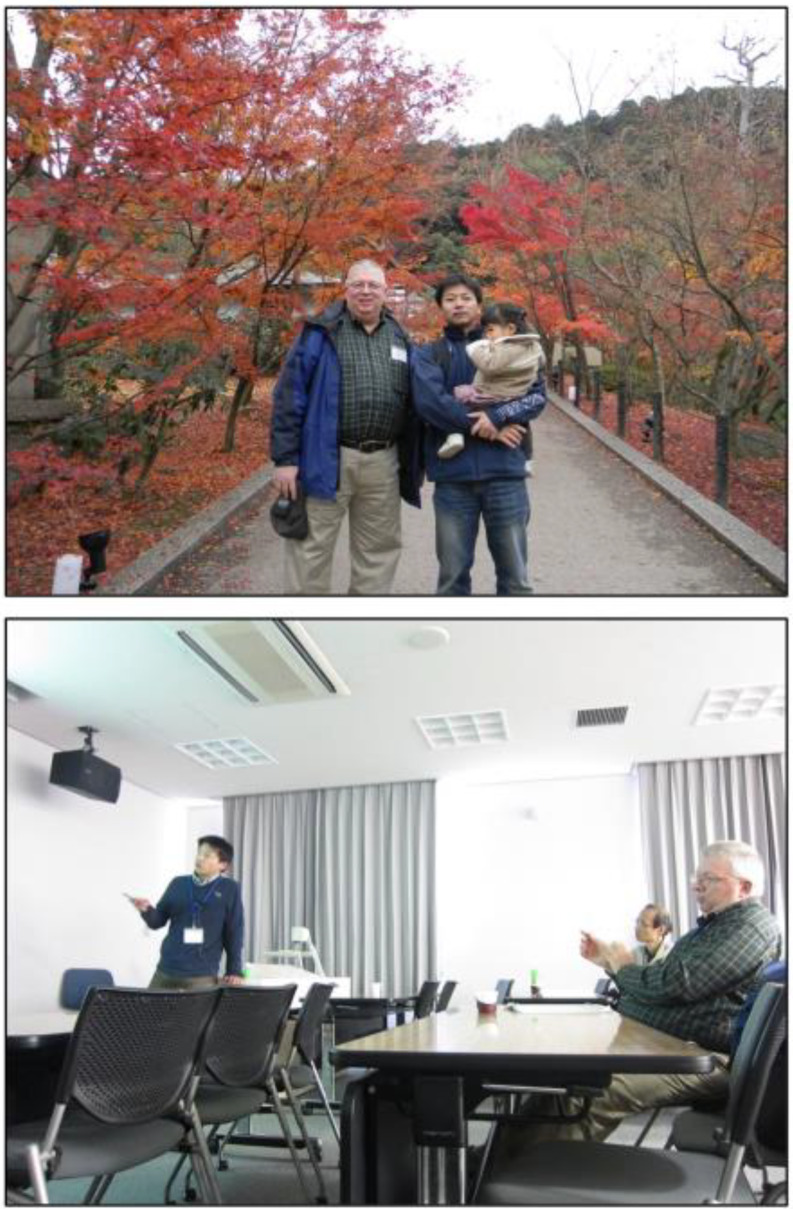
Top: At first, Don said he didn’t understand the beauty of autumn leaves as Japanese people do, but by the time we left, he said, “Indeed, autumn in Japan isn’t so bad!” which left a strong impression on me (2012). Bottom: At a seminar at Ritsumeikan University. Don and my boss at the time, Professor Tamiaki, listened to my presentation (2012)

*Yusuke Tsukatani (JAMSTEC*,* JP)* “I have nothing but good memories of my four years here!” I still remember Don’s big smile when I said that to him on my last day of the postdoctoral fellowship in the Bryant Lab. I have many memories of Don (Fig. 15). But what stays with me most are the events and memories that occurred after I left his lab, rather than during the time there. I invited him to Japan to give a keynote talk at a conference. After the conference, my family and I toured Kyoto with Don. We enjoyed Japanese cuisine at a small restaurant and visited many temples in Kyoto, taking a taxi 13 times in one day—a personal record that still stands. Additionally, a few days earlier, while traveling by plane from Tokyo to Osaka, I forgot my bag on the train, forcing me to return to a certain terminal station to retrieve it, leaving Don alone at Tokyo Haneda Airport. By the time I returned to the airport, the flight we had planned to take had already departed. The lonely figure of Don waiting at the nearly empty boarding gate is still vividly etched in my memory (I can only imagine how anxious he must have felt. At least it became a good story to share later).

During one of the four years I spent at Penn State, Don was on sabbatical at Montana State University. During that year, Kajetan Vogl, a postdoctoral member of Bryant Lab, lived in Don’s huge house on a hill. Actually, just before I returned to Japan, I had to move out of my apartment and had nowhere to stay, so I stayed at Don’s huge house for two weeks (with Kajetan). When I asked Kajetan if he had properly informed Don that I would be staying there, he always gave vague answers, so I think Don probably didn’t know. I had always intended to ask Don about it someday (perhaps when I revisited Penn State), but I never got the chance. It was a very comfortable and pleasant place to stay, and I really appreciated it. Thank you, Don!

*Vera Thiel (DSMZ*,* Germany)* I first met Don in Berlin, Germany in August 2011 where he was attending a conference at the time. I had travelled to see him from Kiel that day, where I was a postdoc at Johannes Imhoff’s group. I had earned my PhD by studying sponge associated bacteria and later moved on to learn the art of cultivation and isolation of anoxygenic phototrophic purple and green bacteria. Now I was inclined to learn all about genome analyses of this particular group, which no one would be better suited to teach than Don. He had just hired my “significant other half”, Marcus Tank (although they had not met as Don was away when Marcus started) and I was hoping to be able to follow him to Penn State for a postdoc position, too. I later learned that he had gathered good experiences hiring couples to the lab before – and I guess that was his way of ensuring a productive work-life balance. Don and I had agreed to a personal meeting while Don was in Berlin after he had already agreed to “hire me somehow”, as he wrote in one email, and although based on his word I had “nothing to worry about”, I was tremendously nervous. I talked about my research findings, indications for a novel group of phototrophic purple bacteria in Chilean salt lakes, which I was hoping to get funded for a follow up project. He listened attentively and asked the pointed and pithy questions I would learn to be his specialty throughout the years. I started my postdoc with him in January 2012 and a few months later had to learn that my project working on Chilean salt lakes was not funded. I was devastated, but Don pragmatically moved me to join his research project on hot springs in Yellowstone National Park. I couldn’t be more grateful for that opportunity. I was thrilled and feel blessed for the three and a half years that I got to spend in Don’s lab on that project. It was full of excitement and special moments. Several day and even overnight samplings in Yellowstone National Park with Dave Ward, Michael Kühl, Marcus Tank, Shane Nowack, Millie Olson, and many others, including a bunch of PNNL researchers were each an unforgettable experience. I met so many wonderful knowledgeable, sharp, kind and witty researchers, each expert in their fields and visited some of the most impressive sceneries and places in the US. We obtained so many samples and metagenome and metatranscriptome data, teaching me valuable analyses skills and laying the basis for many papers written - and if I ever have the time to focus on data analyses and writing, for many more to come. This project and all the experience I gained laid the basis for our wonderful four year stay in Japan, where we studied the Japanese pendant, the Nakabusa hot springs. Don was still by our side throughout the time in Japan, and the time difference would make it just more efficient. I would send my draft after a full day of work, and receive the edits and comments the next morning, ready to be worked on, again. We further met up back in the US for more sampling and meetings. When I was not able to fly to Vancouver to meet him at the ISPP back in 2018 while being pregnant with our first child, I was disappointed and all the more looking forward to meeting him again in the US for his retirement celebration. Unfortunately, Covid-19 put a stop to the plan, and I was devastated to hear I will not get a chance to introduce Don, the friend and mentor we owe so much and who has taught Marcus and me so much about science, birds and loyalty, to our son. Thank you, Don, for all you taught me, all the opportunities you gave me, all the brilliant answers, and all the pointy questions that led me to a deeper understanding of my data and life. I miss you dearly!

## Electronic supplementary material

Below is the link to the electronic supplementary material.

Supplementary Material 1

Supplementary Material 2

## Data Availability

No datasets were generated or analysed during the current study.

## References

[CR1] Agostini A, Shen G, Bryant DA et al (2023) Optically detected magnetic resonance and mutational analysis reveal significant differences in the photochemistry and structure of chlorophyll *f* synthase and photosystem II. Biochim Biophys Acta Bioenerg 1864:149002. 10.1016/j.bbabio.2023.149002 37562512 10.1016/j.bbabio.2023.149002

[CR2] Allewalt JP, Bateson MM, Revsbech NP, et al (2006) Effect of temperature and light on growth of and photosynthesis by *Synechococcus* isolates typical of those predominating in the Octopus Spring microbial mat community of Yellowstone National park. Appl Environ Microbiol 72:544–550. 10.1128/AEM.72.1.544-550.2006 16391090 10.1128/AEM.72.1.544-550.2006 PMC1352173

[CR3] Antonkine ML, Liu G, Bentrop D et al (2002) Solution structure of the unbound, oxidized photosystem I subunit PsaC, containing [4Fe-4S] clusters F_A_ and F_B_: A conformational change occurs upon binding to photosystem I. J Biol Inorg Chem 7:461–472. 10.1007/s00775-001-0321-3 11941504 10.1007/s00775-001-0321-3

[CR4] Becraft ED, Wood JM, Rusch DB et al (2015) The molecular dimension of microbial species: 1. Ecological distinctions among, and homogeneity within, putative ecotypes of *Synechococcus* inhabiting the cyanobacterial mat of mushroom spring, Yellowstone National park. Front Microbiol 6:590. 10.3389/fmicb.2015.00590 10.3389/fmicb.2015.00590 PMC4475828 26157420

[CR5] Bhaya D, Grossman AR, Steunou A-S et al (2007) Population level functional diversity in a microbial community revealed by comparative genomic and metagenomic analyses. ISME J 1:703–713. 10.1038/ismej.2007.46 18059494 10.1038/ismej.2007.46

[CR6] Bos P, Theunissen B, van Iterson GL (1983) Beijerinck and the Delft school of microbiology. Delft University, CN Delft, the Neitherlands

[CR7] Brock TD (1978) Thermophilic microorganisms and life at high temperature. Springer, Heidelberg

[CR8] Brown II, Bryant DA, Casamatta D et al (2010) Polyphasic characterization of a thermotolerant siderophilic filamentous cyanobacterium that produces intracellular iron deposits. Appl Environ Microbiol 76:6664–6672. 10.1128/AEM.00662-10 20709851 10.1128/AEM.00662-10 PMC2950469

[CR9] Bryant DA (1982) Phycoerythrocyanin and phycoerythrin: properties and occurrence in cyanobacteria. Microbiology 128:835–844

[CR10] Bryant DA (1991) Cyanobacterial phycobilisomes: progress toward complete structural and functional analysis via molecular genetics. In: Bogorad L, Vasil IK (eds) The photosynthetic apparatus: molecular biology and operation. Academic, pp 257–300

[CR11] Bryant DA, Garcia Costas AM, Maresca JA et al (2007) *Candidatus* Chloracidobacterium thermophilum: an aerobic phototrophic Acidobacterium. Science 317:523–526. 10.1126/science.1143236 17656724 10.1126/science.1143236

[CR12] Bryant DA, Gisriel CJ (2024) The structural basis for light harvesting in organisms producing phycobiliproteins. Plant Cell 36:4036–4064. 10.1093/plcell/koae126 38652697 10.1093/plcell/koae126 PMC11449063

[CR13] Bryant DA, Guglielmi G, de Marsac NT et al (1979) The structure of cyanobacterial phycobilisomes: a model. Arch Microbiol 123:113–127. 10.1007/BF00446810

[CR14] Bryant DA, Hixson CS, Glazer AN (1978) Structural studies on phycobiliproteins III. Comparison of bilin-containing peptides from the beta subunits of C-phycocyanin, R-phycocyanin, and phycoerythrocyanin. J Biol Chem 253:220–225. 10.1016/S0021-9258(17)38291-1 412849

[CR15] Bryant DA, Hunter CN, Warren MJ (2020a) Biosynthesis of the modified tetrapyrroles-the pigments of life. J Biol Chem 295:6888–6925. 10.1074/jbc.REV120.006194 32241908 10.1074/jbc.REV120.006194 PMC7242693

[CR16] Bryant DA, Shen G, Turner GM et al (2020b) Far-red light allophycocyanin subunits play a role in chlorophyll *d* accumulation in far-red light. Photosynth Res 143:81–95. 10.1007/s11120-019-00689-8 31760552 10.1007/s11120-019-00689-8

[CR17] Charles P, Kalendra V, He Z et al (2020) Two-dimensional ^67^Zn HYSCORE spectroscopy reveals that *a* Zn-bacteriochlorophyll *a*′ dimer is the primary donor (P_840_) in the type-1 reaction centers of *Chloracidobacterium thermophilum*. Phys Chem Chem Phys 22:6457–6467. 10.1039/C9CP06556C 32152610 10.1039/c9cp06556c

[CR18] Chen M, Sawicki A, Wang F (2023) Modeling the characteristic residues of chlorophyll *f* synthase (ChlF) from *Halomicronema hongdechloris* to determine its reaction mechanism. Microorganisms 11:2305. 10.3390/microorganisms11092305 37764149 10.3390/microorganisms11092305 PMC10535343

[CR19] Cherepanov DA, Kurashov V, Gostev FE et al (2024) Femtosecond optical studies of the primary charge separation reactions in far-red photosystem II from *Synechococcus* sp. PCC 7335. Biochim Biophys Acta Bioenerg 1865:149044. 10.1016/j.bbabio.2024.149044 38588942 10.1016/j.bbabio.2024.149044

[CR20] Cherepanov DA, Shelaev IV, Gostev FE et al (2020) Evidence that chlorophyll *f* functions solely as an antenna pigment in far red-light photosystem I from *Fischerella thermalis* PCC 7521. Biochim Biophys Acta Bioenerg 1861:148184 32179058 10.1016/j.bbabio.2020.148184

[CR21] Chew AGM, Bryant DA (2007a) Chlorophyll biosynthesis in bacteria: the origins of structural and functional diversity. Annu Rev Microbiol 61:113–129 17506685 10.1146/annurev.micro.61.080706.093242

[CR22] Chew AGM, Bryant DA (2007b) Characterization of a plant-like protochlorophyllide *a* divinyl reductase in green sulfur bacteria. J Biol Chem 282:2967–2975. 10.1074/jbc.M609730200 17148453 10.1074/jbc.M609730200

[CR23] Chung S, Bryant DA (1996a) Characterization of *csmB* genes, encoding a 7.5-kDa protein of the chlorosome envelope, from the green sulfur bacteria *Chlorobium vibrioforme* 8327D and *Chlorobium tepidum*. Arch Microbiol 166:234–244. 10.1007/s002030050379 8824146 10.1007/s002030050379

[CR24] Chung S, Bryant DA (1996b) Characterization of the *csmD* and *csmE* genes from *Chlorobium tepidum*. The CsmA, CsmC, CsmD, and CsmE proteins are components of the chlorosome envelope. Photosynth Res 50:41–59. 10.1007/BF00018220 24271821 10.1007/BF00018220

[CR25] Chung S, Shen G, Ormerod J, Bryant DA (1998) Insertional inactivation studies of the *csmA* and *csmC* genes of the green sulfur bacterium *Chlorobium vibrioforme* 8327: the chlorosome protein CsmA is required for viability but CsmC is dispensable. FEMS Microbiol Lett 164:353–361. 10.1111/j.1574-6968.1998.tb13109.x 9682485 10.1111/j.1574-6968.1998.tb13109.x

[CR26] Consoli G, Tufail F, Leong HF et al (2024) Locating the missing chlorophylls *f* in far-red photosystem I. 10.1101/2024.08.06.606606. bioRxiv 10.1126/science.ado6830 41066538

[CR27] Domínguez-Martín MA, Sauer PV, Kirst H et al (2022) Structures of a phycobilisome in light-harvesting and photoprotected states. Nature 609:835–845. 10.1038/s41586-022-05156-4 36045294 10.1038/s41586-022-05156-4

[CR28] Dong C, Tang A, Zhao J et al (2009) ApcD is necessary for efficient energy transfer from phycobilisomes to photosystem I and helps to prevent photoinhibition in the cyanobacterium *Synechococcus* sp. PCC 7002. Biochim Biophys Acta Bioenerg 1787:1122–1128. 10.1016/j.bbabio.2009.04.007 10.1016/j.bbabio.2009.04.007 19397890

[CR29] Fairchild CD, Zhao J, Zhou J et al (1992) Phycocyanin alpha-subunit phycocyanobilin lyase. Proc Natl Acad Sci USA 89:7017–7021. 10.1073/pnas.89.15.7017 1495995 10.1073/pnas.89.15.7017 PMC49636

[CR30] Ferris MJ, Ruff-Roberts AL, Kopczynski ED et al (1996) Enrichment culture and microscopy conceal diverse thermophilic *Synechococcus* populations in a single hot spring microbial mat habitat. Appl Environ Microbiol 62:1045–1050. 10.1128/aem.62.3.1045-1050.1996 11536748 10.1128/aem.62.3.1045-1050.1996 PMC167868

[CR32] Frigaard N-U, Bryant DA (2006) Chlorosomes: antenna organelles in photosynthetic green bacteria. In: Shively JM (ed) Complex intracellular structures in prokaryotes. Springer Berlin Heidelberg, Berlin, Heidelberg, pp 79–114

[CR33] Frigaard N-U, Chew AGM, Li H et al (2003) *Chlorobium tepidum*: insights into the structure, physiology, and metabolism of a green sulfur bacterium derived from the complete genome sequence. Photosynth Res 78:93–117. 10.1023/B:PRES.0000004310.96189.b4 16245042 10.1023/B:PRES.0000004310.96189.b4

[CR34] Frigaard N-U, Maqueo Chew AG, Maresca JA, Bryant DA (2006) Bacteriochlorophyll biosynthesis in green bacteria. In: Grimm B, Porra RJ, Rüdiger W, Scheer H (eds) Chlorophylls and bacteriochlorophylls: biochemistry, biophysics, functions and applications. Springer Netherlands, Dordrecht, pp 201–221

[CR31] Frigaard N-U, Voigt GD, Bryant DA (2002) *Chlorobium tepidum* mutant lacking bacteriochlorophyll *c* made by inactivation of the *bchK* gene, encoding bacteriochlorophyll *c* synthase. J Bacteriology 184:3368–3376. 10.1128/jb.184.12.3368-3376.2002 10.1128/JB.184.12.3368-3376.2002 PMC135091 12029054

[CR37] Ganapathy S, Oostergetel GT, Wawrzyniak PK et al (2009) Alternating *syn-anti* bacteriochlorophylls form concentric helical nanotubes in chlorosomes. Proc Natl Acad Sci USA 106:8525–8530. 10.1073/pnas.0903534106 19435848 10.1073/pnas.0903534106 PMC2680731

[CR35] Gan F, Shen G, Bryant DA (2015) Occurrence of far-red light photoacclimation (FaRLiP) in diverse cyanobacteria. Life 5:4–24. 10.3390/life5010004 10.3390/life5010004 PMC4390838 25551681

[CR36] Gan F, Zhang S, Rockwell NC et al (2014) Extensive remodeling of a cyanobacterial photosynthetic apparatus in far-red light. Science 345:1312–1317. 10.1126/science.1256963 25214622 10.1126/science.1256963

[CR38] Garcia Costas AM, Liu Z, Tomsho LP et al (2012) Complete genome of *Candidatus* Chloracidobacterium thermophilum, a chlorophyll-based photoheterotroph belonging to the phylum *Acidobacteria*. Environ Microbiol 14:177–190. 10.1111/j.1462-2920.2011.02592.x 21951563 10.1111/j.1462-2920.2011.02592.x

[CR39] Gisriel C, Elias E, Shen G et al (2023a) Helical allophycocyanin nanotubes absorb far-red light in a thermophilic cyanobacterium. Sci Adv 9:sciadvadg0251 10.1126/sciadv.adg0251 PMC10038336 36961897

[CR40] Gisriel CJ, Bryant DA, Brudvig GW, Cardona T (2023b) Molecular diversity and evolution of far-red light-acclimated photosystem I. Front Plant Sci 14:1289199. 10.3389/fpls.2023.1289199 10.3389/fpls.2023.1289199 PMC10694217 38053766

[CR41] Gisriel CJ, Cardona T, Bryant DA, Brudvig GW (2022a) Molecular evolution of far-red light-acclimated photosystem II. Microorganisms 10:1270. 10.3390/microorganisms10071270 35888987 10.3390/microorganisms10071270 PMC9325196

[CR42] Gisriel CJ, Elias E, Shen G et al (2023c) Structural comparison of allophycocyanin variants reveals the molecular basis for their spectral differences. Photosynth Res 162:157–170. 10.1007/s11120-023-01048-4 37773575 10.1007/s11120-023-01048-4 PMC11614940

[CR43] Gisriel CJ, Flesher DA, Long Z et al (2023d) A quantitative assessment of (bacterio)chlorophyll assignments in the cryo-EM structure of the *Chloracidobacterium thermophilum* reaction center. Photosynth Res 162:187–196. 10.1007/s11120-023-01047-5 37749456 10.1007/s11120-023-01047-5 PMC13714760

[CR44] Gisriel CJ, Flesher DA, Shen G et al (2022b) Structure of a photosystem I-ferredoxin complex from a marine cyanobacterium provides insights into far-red light photoacclimation. J Biol Chem 298:101408. 10.1016/j.jbc.2021.101408 34793839 10.1016/j.jbc.2021.101408 PMC8689207

[CR45] Gisriel CJ, Huang H-L, Reiss KM et al (2021) Quantitative assessment of chlorophyll types in cryo-EM maps of photosystem I acclimated to far-red light. BBA Adv 1:100019. 10.1016/j.bbadva.2021.100019 37082022 10.1016/j.bbadva.2021.100019 PMC10074859

[CR46] Gisriel CJ, Shen G, Brudvig GW, Bryant DA (2023e) Structure of the antenna complex expressed during far-red light photoacclimation in *Synechococcus* sp. PCC 7335. J Biol Chem 300:105590. 10.1016/j.jbc.2023.105590 10.1016/j.jbc.2023.105590 PMC10810746 38141759

[CR47] Gisriel CJ, Shen G, Flesher DA et al (2023f) Structure of a dimeric photosystem II complex from a cyanobacterium acclimated to far-red light. J Biol Chem 299:102815. 10.1016/j.jbc.2022.102815 36549647 10.1016/j.jbc.2022.102815 PMC9843442

[CR48] Gisriel CJ, Shen G, Ho M-Y et al (2022c) Structure of a monomeric photosystem II core complex from a cyanobacterium acclimated to far-red light reveals the functions of chlorophylls *d* and *f*. J Biol Chem 298:101424. 10.1016/j.jbc.2021.101424 34801554 10.1016/j.jbc.2021.101424 PMC8689208

[CR49] Gisriel CJ, Shen G, Kurashov V et al (2020a) The structure of photosystem I acclimated to far-red light illuminates an ecologically important acclimation process in photosynthesis. Sci Adv 6:eaay6415. 10.1126/sciadv.aay6415 32076649 10.1126/sciadv.aay6415 PMC7002129

[CR50] Gisriel CJ, Wang J, Brudvig GW, Bryant DA (2020b) Opportunities and challenges for assigning cofactors in cryo-EM density maps of chlorophyll-containing proteins. Commun Biol 3:408. 10.1038/s42003-020-01139-1 32733087 10.1038/s42003-020-01139-1 PMC7393486

[CR51] Glazer AN, Bryant DA (1975) Allophycocyanin B (λ_max_ 671, 618 nm): A new cyanobacterial phycobiliprotein. Arch Microbiol 104:15–22. 10.1007/BF00447294 808186 10.1007/BF00447294

[CR52] Golbeck JH, Bryant DA (1991) Photosystem I. Curr Top Bioenergetics 16:83–177

[CR53] Grimme RA, Lubner CE, Bryant DA, Golbeck JH (2008) Photosystem I/molecular wire/metal nanoparticle bioconjugates for the photocatalytic production of H_2_. J Am Chem Soc 130:6308–6309. 10.1021/ja800923y 18439011 10.1021/ja800923y

[CR54] Guglielmi G, Cohen-Bazire G, Bryant DA (1981) The structure of *Gloeobacter violaceus* and its phycobilisomes. Arch Microbiol 129:181–189. 10.1007/BF00425248

[CR55] Ho M-Y, Bryant DA (2019) Global transcriptional profiling of the cyanobacterium *Chlorogloeopsis fritschii* PCC 9212 in far-red light: insights into the regulation of chlorophyll *d* synthesis. Front Microbiol Volume. 10:465 10.3389/fmicb.2019.00465 10.3389/fmicb.2019.00465 PMC6424891 30918500

[CR57] Ho MY, Gan F, Shen G, Bryant DA (2017b) Far-red light photoacclimation (FaRLiP) in *Synechococcus* sp. PCC 7335. II. Characterization of phycobiliproteins produced during acclimation to far-red light. Photosynth Res 131:187–202. 10.1007/s11120-016-0303-5 27623780 10.1007/s11120-016-0303-5

[CR56] Ho MY, Gan F, Shen G et al (2017a) Far-red light photoacclimation (FaRLiP) in *Synechococcus* sp. PCC 7335: I. Regulation of FaRLiP gene expression. Photosynth Res 131:173–186. 10.1007/s11120-016-0309-z 27638320 10.1007/s11120-016-0309-z

[CR58] Ho M-Y, Niedzwiedzki DM, MacGregor-Chatwin C et al (2020) Extensive remodeling of the photosynthetic apparatus alters energy transfer among photosynthetic complexes when cyanobacteria acclimate to far-red light. Biochim Biophys Acta Bioenerg 1861:148064. 10.1016/j.bbabio.2019.148064 31421078 10.1016/j.bbabio.2019.148064

[CR59] Ho MY, Shen G, Canniffe DP et al (2016) Light-dependent chlorophyll *f* synthase is a highly divergent paralog of PsbA of photosystem II. Science 353:aaf9178. 10.1126/science.aaf9178 27386923 10.1126/science.aaf9178

[CR60] Ho M-Y, Soulier NT, Canniffe DP et al (2017c) Light regulation of pigment and photosystem biosynthesis in cyanobacteria. Curr Opin Plant Biol 37:24–33. 10.1016/j.pbi.2017.03.006 28391049 10.1016/j.pbi.2017.03.006

[CR61] Inskeep WP, Jay ZJ, Tringe SG et al (2013) The YNP metagenome project: environmental parameters responsible for microbial distribution in the Yellowstone geothermal ecosystem. Front Microbiol 4:67 10.3389/fmicb.2013.00067 10.3389/fmicb.2013.00067 PMC3644721 23653623

[CR63] Jagannathan B, Golbeck JH (2009) Breaking biological symmetry in membrane proteins: the asymmetrical orientation of PsaC on the pseudo-C_2_ symmetric photosystem I core. Cell Mol Life Sci 66:1257–1270. 10.1007/s00018-009-8673-x 19132290 10.1007/s00018-009-8673-x PMC11131447

[CR64] Jiang H-W, Wu H-Y, Wang C-H et al (2023) A structure of the relict phycobilisome from a thylakoid-free cyanobacterium. Nat Commun 14:8009. 10.1038/s41467-023-43646-9 38049400 10.1038/s41467-023-43646-9 PMC10696076

[CR65] Jung Y-S, Vassiliev IR, Qiao F et al (1996) Modified ligands to F_A_ and F_B_ in photosystem I: proposed chemical rescue of a [4Fe-4S] cluster with an external thiolate in alanine, glycine, and serine mutants of PsaC. J Biol Chem 271:31135–31144. 10.1074/jbc.271.49.31135 8940111 10.1074/jbc.271.49.31135

[CR66] Kato K, Shinoda T, Nagao R et al (2020) Structural basis for the adaptation and function of chlorophyll *f* in photosystem I. Nat Commun 11:238. 10.1038/s41467-019-13898-5 31932639 10.1038/s41467-019-13898-5 PMC6957486

[CR67] Kim Y-M, Nowack S, Olsen MT et al (2015) Diel metabolomics analysis of a hot spring chlorophototrophic microbial mat leads to new hypotheses of community member metabolisms. Front Microbiol 6:209. 10.3389/fmicb.2015.00209 10.3389/fmicb.2015.00209 PMC4400912 25941514

[CR68] Klatt CG, Bryant DA, Ward DM (2007) Comparative genomics provides evidence for the 3-hydroxypropionate autotrophic pathway in filamentous anoxygenic phototrophic bacteria and in hot spring microbial mats. Environ Microbiol 9:2067–2078. 10.1111/j.1462-2920.2007.01323.x 17635550 10.1111/j.1462-2920.2007.01323.x

[CR62] Klatt CG,Inskeep WP, Herrgard MJ et al (2013b) Community structure and function of high-temperature chlorophototrophic microbial mats inhabiting diverse geothermal environments. Front Microbiol. 4:106 10.3389/fmicb.2013.00106 23761787 10.3389/fmicb.2013.00106 PMC3669762

[CR69] Klatt CG, Liu Z, Ludwig M et al (2013) Temporal metatranscriptomic patterning in phototrophic Chloroflexi inhabiting a microbial mat in a geothermal spring. ISME J 7:1775–1789. 10.1038/ismej.2013.52 23575369 10.1038/ismej.2013.52 PMC3749495

[CR70] Klatt CG, Wood JM, Rusch DB et al (2011) Community ecology of hot spring cyanobacterial mats: predominant populations and their functional potential. ISME J 5:1262–1278. 10.1038/ismej.2011.73 21697961 10.1038/ismej.2011.73 PMC3146275

[CR71] Kurashov V, Ho MY, Shen G et al (2019) Energy transfer from chlorophyll *f* to the trapping center in naturally occurring and engineered photosystem I complexes. Photosynth Res 141:151–163. 10.1007/s11120-019-00616-x 30710189 10.1007/s11120-019-00616-x

[CR72] Ley AC, Butler WL, Bryant DA, Glazer AN (1977) Isolation and function of allophycocyanin B of *Porphyridium cruentum*. Plant Physiol 59:974–980. 10.1104/pp.59.5.974 16659979 10.1104/pp.59.5.974 PMC543342

[CR74] Lian Y, Zhao J, Mühlenhoff U et al (1993) PsaE is required for *in vivo* cyclic electron flow around photosystem I in the cyanobacterium *Synechococcus* sp. PCC 7002. Plant Physiol 103:171–180 12231924 10.1104/pp.103.1.171 PMC158960

[CR73] Li N, Zhao J, Warren PV et al (1991) PsaD is required for the stable binding of PsaC to the photosystem I core protein of *Synechococcus* sp. PCC 6301 Biochemistry 30:7863–7872. 10.1021/bi00245a028 10.1021/bi00245a028 1651109

[CR75] Liu Z, Klatt CG, Ludwig M et al (2012) ‘*Candidatus* Thermochlorobacter aerophilum:’ an aerobic chlorophotoheterotrophic member of the phylum *Chlorobi* defined by metagenomics and metatranscriptomics. ISME J 6:1869–1882. 10.1038/ismej.2012.24 22456447 10.1038/ismej.2012.24 PMC3446795

[CR76] Liu Z, Klatt CG, Wood JM et al (2011) Metatranscriptomic analyses of chlorophototrophs of a hot-spring microbial mat. ISME J 5:1279–1290. 10.1038/ismej.2011.37 21697962 10.1038/ismej.2011.37 PMC3146272

[CR77] Lubner CE, Applegate AM, Knörzer P et al (2011) Solar hydrogen-producing bionanodevice outperforms natural photosynthesis. Proc Natl Acad Sci USA 108:20988–20991. 10.1073/pnas.1114660108 22160679 10.1073/pnas.1114660108 PMC3248548

[CR78] Ludwig M, Pandelia M-E, Chew CY et al (2014) ChlR protein of *Synechococcus* sp. PCC 7002 is a transcription activator that uses an oxygen-sensitive [4Fe-4S] cluster to control genes involved in pigment biosynthesis. J Biol Chem 289:16624–16639. 10.1074/jbc.M114.561233 24782315 10.1074/jbc.M114.561233 PMC4059106

[CR79] Maxson P, Sauer K, Zhou J et al (1989) Spectroscopic studies of cyanobacterial phycobilisomes lacking core polypeptides. Biochim Biophys Acta Bioenerg 977:40–51. 10.1016/S0005-2728(89)80007-6 10.1016/s0005-2728(89)80007-6 2508754

[CR80] Mehari T, Qiao F, Scott MP et al (1995) Modified ligands to F_A_ and F_B_ in photosystem I: I. Structural constraints for the formation of iron-sulfur clusters in free and rebound PsaC. J Biol Chem 270:28108–28117. 10.1074/jbc.270.47.28108 7499299 10.1074/jbc.270.47.28108

[CR81] Miller SR, Wingard CE, Castenholz RW (1998) Effects of visible light and UV radiation on photosynthesis in a population of a hot spring cyanobacterium, a *Synechococcus* sp., subjected to high-temperature stress. Appl Environ Microbiol 64:3893–3899. 10.1128/AEM.64.10.3893-3899.1998 9758816 10.1128/aem.64.10.3893-3899.1998 PMC106575

[CR202] Moran JJ, Ilhardt PD, Couvillion SP, Lipton MS, Metz TO, Tolic N, Bryant DA, Ward DM. Mid-day photomixotrophy by Roseiflexus spp. and implications for the ^13^C content of hot spring cyanobacterial mats. Appl Environ Microbiol (in revision) 10.1128/aem.00909-25 PMC12542760 40899803

[CR82] Nowack S, Olsen MT, Schaible GA et al (2015) The molecular dimension of microbial species: 2. *Synechococcus* strains representative of putative ecotypes inhabiting different depths in the Mushroom Spring microbial mat exhibit different adaptive and acclimative responses to light. Front Microbiol 6:626. 10.3389/fmicb.2015.00626 10.3389/fmicb.2015.00626 PMC4484337 26175719

[CR83] Olsen M, Nowack S, Wood J et al (2015) The molecular dimension of microbial species: 3. Comparative genomics of *Synechococcus* strains with different light responses and *in situ* diel transcription patterns of associated putative ecotypes in the Mushroom Spring microbial mat. Front Microbiol 6:604. 10.3389/fmicb.2015.00604 10.3389/fmicb.2015.00604 PMC4477158 26157428

[CR87] Revsbech NP, Trampe E, Lichtenberg M et al (2016) *In situ* hydrogen dynamics in a hot spring microbial mat during a diel cycle. Appl Environ Microbiol 82:4209–4217. 10.1128/AEM.00710-16 27208140 10.1128/AEM.00710-16 PMC4959218

[CR84] Qi M, Taunt HN, Bečková M et al (2025) Enhancing the production of chlorophyll *f* in the cyanobacterium *Synechocystis* sp. PCC 6803. Physiol Plant 177:e70169. 10.1111/ppl.70169 40139952 10.1111/ppl.70169 PMC11946780

[CR85] Ramsing NB, Ferris MJ, Ward DM (2000) Highly ordered vertical structure of *Synechococcus* populations within the one-millimeter-thick photic zone of a hot spring cyanobacterial mat. Appl Environ Microbiol 66:1038–1049. 10.1128/AEM.66.3.1038-1049.2000 10698769 10.1128/aem.66.3.1038-1049.2000 PMC91940

[CR86] Ranepura GA, Mao J, Vermaas JV et al (2023) Computing the relative affinity of chlorophylls *a* and *b* to light-harvesting complex II. J Phys Chem B 127:10974–10986. 10.1021/acs.jpcb.3c06273 38097367 10.1021/acs.jpcb.3c06273

[CR88] Saini MK, Sebastian A, Shirotori Y et al (2021) Genomic and phenotypic characterization of *Chloracidobacterium* isolates provides evidence for multiple species. Front Microbiol 12:704168. 10.3389/fmicb.2021.704168 10.3389/fmicb.2021.704168 PMC8245765 34220789

[CR89] Saini MK, Villena-Alemany C, Kuzyk SB et al (2025) *Chloracidobacterium validum* sp. nov., a thermophilic chlorophotoheterotrophic bacterium of the phylum *Acidobacteriota* from an alkaline hot spring microbial mat, emended descriptions of *Chloracidobacterium* and *Chloracidobacterium thermophilum*, and descriptions of *Chloracidobacteriaceae* fam. nov. and *Chloracidobacteriales* ord. nov. Int J Syst Evol Microbiol. In revision 10.1099/ijsem.0.007003 PMC12816986 41553838

[CR90] Saunders AH, Golbeck JH, Bryant DA (2013) Characterization of BciB: A ferredoxin-dependent 8-vinyl-protochlorophyllide reductase from the green sulfur bacterium *Chloroherpeton thalassium*. Biochemistry 52:8442–8451. 10.1021/bi401172b 24151992 10.1021/bi401172b

[CR91] Schaffert CS, Klatt CG, Ward DM et al (2012) Identification and distribution of high-abundance proteins in the Octopus Spring microbial mat community. Appl Environ Microbiol 78:8481–8484. 10.1128/AEM.01695-12 23001677 10.1128/AEM.01695-12 PMC3497366

[CR92] Shen G, Balasubramanian R, Wang T et al (2007) SufR coordinates two [4Fe-4S]^2+, 1+^ clusters and functions as a transcriptional repressor of the *sufBCDS* Operon and an autoregulator of *sufR* in cyanobacteria. J Biol Chem 282:31909–31919. 10.1074/jbc.M705554200 17827500 10.1074/jbc.M705554200

[CR93] Shen G, Canniffe DP, Ho M-Y et al (2019) Characterization of chlorophyll *f* synthase heterologously produced in *Synechococcus* sp. PCC 7002. Photosynth Res 140:77–92. 10.1007/s11120-018-00610-9 30607859 10.1007/s11120-018-00610-9

[CR94] Shen G, Gan F, Bryant DA (2016) The *siderophilic cyanobacterium** Leptolyngbya* sp. strain JSC-1 acclimates to iron starvation by expressing multiple *isiA*-family genes. Photosynth Res 128:325–340. 10.1007/s11120-016-0257-7 27071628 10.1007/s11120-016-0257-7

[CR95] Soulier N, Bryant DA (2021) The structural basis of far-red light absorbance by allophycocyanins. Photosynth Res 147:11–26. 10.1007/s11120-020-00787-y 33058014 10.1007/s11120-020-00787-y

[CR96] Soulier N, Laremore TN, Bryant DA (2020) Characterization of cyanobacterial allophycocyanins absorbing far-red light. Photosynth Res 145:189–207. 10.1007/s11120-020-00775-2 32710194 10.1007/s11120-020-00775-2

[CR98] Soulier NT (2021) Light-harvesting proteins and complexes in cyanobacteria acclimated to far-red and low-light conditions. The Pennsylvania State University

[CR97] Soulier N, Walters K, Laremore TN et al (2022) Acclimation of the photosynthetic apparatus to low light in a thermophilic *Synechococcus* sp. strain. Photosynth Res 153:21–42. 10.1007/s11120-022-00918-7 35441927 10.1007/s11120-022-00918-7

[CR99] Steinke L, Slysz GW, Lipton MS et al (2020) Short-term stable isotope probing of proteins reveals taxa incorporating inorganic carbon in a hot spring microbial mat. Appl Environ Microbiol 86:e01829–e01819. 10.1128/AEM.01829-19 31953342 10.1128/AEM.01829-19 PMC7082580

[CR100] Steunou A-S, Bhaya D, Bateson MM et al (2006) *In situ* analysis of nitrogen fixation and metabolic switching in unicellular thermophilic cyanobacteria inhabiting hot spring microbial mats. Proc Natl Acad Sci USA 103:2398–2403. 10.1073/pnas.0507513103 16467157 10.1073/pnas.0507513103 PMC1413695

[CR101] Strunecký O, Ivanova AP, Mareš J (2023) An updated classification of cyanobacterial orders and families based on phylogenomic and polyphasic analysis. J Phycol 59:12–51. 10.1111/jpy.13304 36443823 10.1111/jpy.13304

[CR102] Swanson RV, Zhou J, Leary JA et al (1992) Characterization of phycocyanin produced by cpcE and cpcF mutants and identification of an intergenic suppressor of the defect in bilin attachment. J Biol Chem 267:16146–16154. 10.1016/S0021-9258(18)41979-5 1644802

[CR103] Tank M, Bryant DA (2015a) *Chloracidobacterium thermophilum* gen. nov., sp. nov.: an anoxygenic microaerophilic chlorophotoheterotrophic acidobacterium. Int J Syst Evol Microbiol 65:1426–1430. 10.1099/ijs.0.000113 25667398 10.1099/ijs.0.000113

[CR104] Tank M, Bryant DA (2015b) Nutrient requirements and growth physiology of the photoheterotrophic acidobacterium, *Chloracidobacterium thermophilum*. Front Microbiol 6:226. 10.3389/fmicb.2015.00226 10.3389/fmicb.2015.00226 PMC4376005 25870589

[CR105] Tank M, Thiel V, Ward DM, Bryant DA (2017) A panoply of phototrophs: an overview of the thermophilic chlorophototrophs of the microbial mats of alkaline siliceous hot springs in Yellowstone National park, WY, USA. In: Hallenbeck PC (ed) Modern topics in the phototrophic prokaryotes: environmental and applied aspects. Springer, Berlin, pp 87–137

[CR106] Thiel V, Garcia Costas AM, Fortney NW et al (2019) *Candidatus* Thermonerobacter thiotrophicus, a non-phototrophic member of the *Bacteroidetes*/*Chlorobi* with dissimilatory sulfur metabolism in hot spring mat communities. Front Microbiol 9:3159. 10.3389/fmicb.2018.03159 10.3389/fmicb.2018.03159 PMC6338057 30687241

[CR107] Thiel V, Hügler M, Ward DM, Bryant DA (2017) The dark side of the Mushroom Spring microbial mat: life in the shadow of chlorophototrophs. II. Metabolic functions of abundant community members predicted from metagenomic analyses. Front Microbiol 8:943. 10.3389/fmicb.2017.00943 10.3389/fmicb.2017.00943 PMC5459899 28634470

[CR108] Thiel V, Wood JM, Olsen MT et al (2016) The dark side of the Mushroom Spring microbial mat: life in the shadow of chlorophototrophs. I. Microbial diversity based on 16S rRNA gene amplicons and metagenomic sequencing. Front Microbiol 7:919. 10.3389/fmicb.2016.00919 10.3389/fmicb.2016.00919 PMC4911352 27379049

[CR109] Thweatt JL, Ferlez BH, Golbeck JH, Bryant DA (2017) BciD is a radical S-adenosyl-l-methionine (SAM) enzyme that completes bacteriochlorophyllide *e* biosynthesis by oxidizing a methyl group into a formyl group at C-7. J Biol Chem 292:1361–1373. 10.1074/jbc.M116.767665 27994052 10.1074/jbc.M116.767665 PMC5270479

[CR110] Trinugroho JP, Bečková M, Shao S et al (2020) Chlorophyll *f* synthesis by a super-rogue photosystem II complex. Nat Plants 6:238–244. 10.1038/s41477-020-0616-4 32170286 10.1038/s41477-020-0616-4

[CR111] Tsukatani Y, Romberger SP, Golbeck JH, Bryant DA (2012) Isolation and characterization of homodimeric type-I reaction center complex from *Candidatus* Chloracidobacterium thermophilum, an aerobic chlorophototroph. J Biol Chem 287:5720–5732. 10.1074/jbc.M111.323329 22184116 10.1074/jbc.M111.323329 PMC3285344

[CR112] van der Meer MTJ, Schouten Stefan, Bateson MM et al (2005) Diel variations in carbon metabolism by green nonsulfur-like bacteria in alkaline siliceous hot spring microbial mats from Yellowstone National park. Appl Environ Microbiol 71:3978–3986. 10.1128/AEM.71.7.3978-3986.2005 16000812 10.1128/AEM.71.7.3978-3986.2005 PMC1168979

[CR200] van der Meer MTJ, Klatt CG, Wood J, et al (2010) Cultivation and genomic, nutritional, and lipid biomarker characterization of *Roseiflexus* strains closely related to predominant in situ populations inhabiting Yellowstone hot spring microbial mats. J Bacteriol 192:3033–3042. 10.1128/jb.01610-09 10.1128/JB.01610-09 PMC2901690 20363941

[CR201] van Niel CB (1949) The Delft School and the rise of general microbiology. Bacteriol Rev 13:161–174 10.1128/br.13.3.161-174.1949 PMC180708 16350131

[CR113] Wang T, Shen G, Balasubramanian R et al (2004) The *sufR* gene (*sll0088* in *Synechocystis* sp. strain PCC 6803) functions as a repressor of the *sufBCDS* operon in iron-sulfur cluster biogenesis in cyanobacteria. J Bacteriol 186:956–967. 10.1128/jb.186.4.956-967.2004 14761990 10.1128/JB.186.4.956-967.2004 PMC344230

[CR114] Ward DM, Ferris MJ, Nold SC, Bateson MM (1998) A natural view of microbial biodiversity within hot spring cyanobacterial mat communities. Microbiol Mol Biol Rev 62:1353–1370. 10.1128/mmbr.62.4.1353-1370.1998 9841675 10.1128/mmbr.62.4.1353-1370.1998 PMC98949

[CR115] Ward DM, Weller R, Bateson MM (1990) 16S rRNA sequences reveal numerous uncultured microorganisms in a natural community. Nature 345:63–65. 10.1038/345063a0 1691827 10.1038/345063a0

[CR116] Zarzycki J, Fuchs G (2011) Coassimilation of organic substrates via the autotrophic 3-hydroxypropionate bi-cycle in *Chloroflexus aurantiacus*. Appl Environ Microbiol 77:6181–6188. 10.1128/AEM.00705-11 21764971 10.1128/AEM.00705-11 PMC3165406

[CR117] Zhang S, Bryant DA (2011) The tricarboxylic acid cycle in cyanobacteria. Science 334:1551–1553. 10.1126/science.1210858 22174252 10.1126/science.1210858

[CR118] Zhang S, Shen G, Li Z et al (2014) Vipp1 is essential for the biogenesis of photosystem I but not thylakoid membranes in *Synechococcus* sp. PCC 7002. J Biol Chem 289:15904–15914. 10.1074/jbc.M114.555631 24764304 10.1074/jbc.M114.555631 PMC4047364

[CR119] Zhao C, Gan F, Shen G, Bryant DA (2015) RfpA, RfpB, and RfpC are the master control elements of far-red light photoacclimation (FaRLiP). Front Microbiol 6:1303. 10.3389/fmicb.2015.01303 10.3389/fmicb.2015.01303 PMC4658448 26635768

[CR120] Zhao J, Li N, Warren PV et al (1992) Site-directed conversion of a cysteine to aspartate leads to the assembly of a N iron-sulfur [3Fe-4S] cluster to PsaC of photosystem I. The photoreduction of F_A_ is independent of F_B_. Biochemistry 31:5093–5099. 10.1021/bi00137a001 1318744 10.1021/bi00137a001

[CR121] Zhao J, Warren PV, Li N et al (1990) Reconstitution of electron transport in photosystem I with PsaC and PsaD proteins expressed in *Escherichia coli*. FEBS Lett 276:175–180. 10.1016/0014-5793(90)80536-R 2125006 10.1016/0014-5793(90)80536-r

[CR122] Zheng L, Zheng Z, Li X et al (2021) Structural insight into the mechanism of energy transfer in cyanobacterial phycobilisomes. Nat Commun 12:5497. 10.1038/s41467-021-25813-y 34535665 10.1038/s41467-021-25813-y PMC8448738

[CR123] Zhou J, Gasparich GE, Stirewalt VL et al (1992) The *cpcE* and *cpcF* genes of *Synechococcus* sp. PCC 7002. Construction and phenotypic characterization of interposon mutants. J Biol Chem 267:16138–16145. 10.1016/S0021-9258(18)41978-3 1644801

[CR124] Zhou J, Stirewalt VL, Bryant DA (1990) Molecular cloning, nucleotide sequencing and mutagenesis of the *apcD* gene of *Synechococcus* sp. PCC 7002. In: Stevens SEJ, Bryant DA (eds) Light-Energy transduction in photosynthesis: higher plant and bacterial models. American Society for Plant Physiologists, Rockville, MD, pp 340–343

