# Supplementary material for: Remembering Don Bryant (1950–2024)
Source: Photosynth Res. 2025 Jul 1;163(4):37. doi: 10.1007/s11120-025-01158-1 (PMC12214013; doi:10.1007/s11120-025-01158-1)
Supplement: Supplementary file 2 — Supplementary Material 2 [file 11120_2025_1158_MOESM2_ESM.docx]

Curriculum Vitae: Donald A. (Don) Bryant

**Academy Professor, Emeritus Academy**

**Ernest C. Pollard Professor Emeritus of Biotechnology Professor Emeritus of Biochemistry and Molecular Biology The Pennsylvania State University, University Park, PA**

## Professional Preparation

| Massachusetts Institute of Technology | Chemistry/Biology | B. Sc. | 1972 |
| --- | --- | --- | --- |
| Univ. of California, Los Angeles (AN Glazer; FA Eiserling) | Molecular Biology | Ph. D. | 1977 |
| Institut Pasteur (R. Y. Stanier, G. Cohen-Bazire) | Microbiology | Postdoc | 1977-1979 |
| Cornell University (R. K. Clayton) | Biophysics | Postdoc | 1979-1981 |

### Thesis

*Comparative Studies on Cyanobacterial and Rhodophytan Biliproteins.*

University of California, Los Angeles, 425 pp.

## Appointments

2023—2024 Academy Professor, The Pennsylvania State University

2022—2024 Ernest C. Pollard Professor Emeritus of Biotechnology, The Pennsylvania State University

2022—2024 Professor Emeritus of Biochemistry and Molecular Biology, The Pennsylvania State University

2015—2020 Research Professor, Chemistry & Biochemistry, Montana State University 2013—2018 Visiting Professor, Singapore Centre for Environmental Life Sciences

Engineering, Nanyang Technological University, Singapore 2009—2020 Affiliate Member, Thermal Biology Institute, Montana State University

2009—2015 Adjunct Research Professor, Chemistry & Biochemistry, Montana State Univ. 2006—2022 Member, NSF-sponsored Research Coordination Network, Yellowstone Nat. Park 2005 Visiting Fellow, Thermal Biology Inst., Montana State Univ. (David M. Ward) 1996—1997 Anniversary Fellow, The Australian National University, Canberra, Australia 1992—2022 Ernest C. Pollard Professor of Biotechnology, The Pennsylvania State University 1989—1990 Visiting Professor, Eidgenössische Technische Hochschule, Institute für

Molekularbiologie und Biophysik, Zürich, Switzerland

1981—2022 Assistant, Associate, (1986) and Full Professor (1991), Department of Biochemistry and Molecular Biology, The Pennsylvania State University

1979—1981 DOE Postdoctoral Research Fellow, Cornell University (Dr. Roderick K. Clayton) 1977—1979 N.S.F.-C.N.R.S. Postdoctoral Fellow (U.S.--France Exchange program), Institut Pasteur, Unité de Physiologie Microbienne (Drs. Roger Y. Stanier and Germaine

Cohen-Bazire

1975 Teaching Assistant, Dept. of Bacteriology, University of California, Los Angeles 1972—1976 U.S. Publ. Health Service, Predoc. Trainee, University of California-Los Angeles (Advisors: Alexander N. Glazer and Frederick A. Eiserling)

## Honors

2023 Member, Emeritus Academy, The Pennsylvania State University

### 2022 Award for Basic Research, American Society for Microbiology,

**2020 Charles F. Kettering Award (excellence in photosynthesis research), American Society of Plant Biologists**

2020—present Member, Sigma Xi, The Scientific Research Honor Society 2019—present Member, Rosalie Edge Society, Hawk Mountain Sanctuary, PA

### 2018 D. C. White Award for Interdisciplinary Research and Mentoring, American Society for Microbiology

2012—2018 Member (elected), Board of Governors, American Academy of Microbiology 2012 Daniel I. Arnon Lecturer, University of California, Berkeley

### 2011—2024 Fellow, American Association for the Advancement of Science

2010 Daniel R. Tershak Memorial Teaching Award, Dept. of Biochemistry and Molecular Biology, The Pennsylvania State University

2010 Brown & Williamson Distinguished Lecturer, University of Louisville, Department of Biology

2009—2020 Member, Board of Directors, Rebeiz Foundation for Basic Research

2008 Annual Research Paper Prize for 2007, Rebeiz Foundation for Basic Research 2004—2010 Geographical Representative for the Americas, International Society for

Photosynthesis Research

2002 Chairman, Gordon Conference, Biochemical Aspects of Photosynthesis 2000—2004 Member, Department of Energy, Energy Biosciences Advisory Council 2000 First Pasakarnis-Buchanan Lecturer, Bowling Green State University

2000 Vice-Chairman, Gordon Conference, Biophysical Aspects of Photosynthesis 1996—1997 Anniversary Fellow, The Australian National University

### 1995—2024 Fellow, American Academy of Microbiology 1992—2022 Ernest C. Pollard Professor of Biotechnology

1995—1997 Chairman, NIH-GMS Study Section, Microbial Physiology (MBC-1) 1992—1996 Faculty Senator, Eberly College of Science, The Pennsylvania State University 1989—1990 Visiting Professor, Eidgenössische Technische Hochschule, Institute für

Molekularbiologie und Biophysik, Zürich, Switzerland

1986 Early Promotion and Tenure, The Pennsylvania State University 1977—1979 N.S.F.-C.N.R.S. Postdoctoral fellowship (U.S.--France exchange program).

1972—1976 U. S. Public Health Service Pre-doctoral Traineeship, Univ. of California-Los Angeles (Advisors: Alexander N. Glazer and Frederick A. Eiserling)

1972 B. Sc. in Chemistry with Honors, Massachusetts Institute of Technology 1971 Member, Phi Lambda Upsilon, Chemistry Honor Fraternity

## Academic and Professional Service

1972—1976 U. S. Public Health Service predoctoral trainee (National Institutes of Health) Dissertation advisors, Alexander N. Glazer and Frederick A. Eiserling

1975 Teaching Assistant, Department of Bacteriology, University of California, Los Angeles 1977—1979 N.S.F.-C.N.R.S. Postdoctoral fellow (U. S.—France exchange program), Institut Pasteur,

Unité de Physiologie Microbienne, (Drs. Roger Y. Stanier and Germaine Cohen-Bazire) 1979—1981 DOE Postdoctoral fellow, Cornell University, Department of Botany, Genetics, and

Development, (laboratory of Roderick K. Clayton).

### 1981—1984 Assistant Professor of Microbiology, The Pennsylvania State University

1983— Adjunct faculty member in the intercollege program of genetics

1983— Adjunct faculty member in the intercollege program of plant physiology/plant biology 1984— Member of the Cooperative Program in Recombinant DNA Technology

1984—1988 Director and Operator, DNA Synthesis Facility for the College of Science, The Pennsylvania State University

1985 *Ad hoc* Contract Review Committee, National Cancer Institute

### 1985 Assistant Professor of Molecular & Cell Biology, The Pennsylvania State University

1986—1991 Editorial Board, ***Journal of Bacteriology***

1986—1987 Editorial Board, ***Photosynthesis Research***

### 1986—1991 Associate Professor of Molecular & Cell Biology, The Pennsylvania State University

1986— Member, Biotechnology Institute, The Pennsylvania State University 1988 Panel Member, U.S. Dept. of Agriculture, Photosynthesis 1988—1998 Associate Editor, ***Archives of Microbiology***

1988—1998 Associate Editor, ***Photosynthesis Research***

1989—1990 Visiting Professor, Eidgenössische Technische Hochschule, Institute für Molekularbiologie und Biophysik, Zürich, Switzerland

1990—1991 Local Organizing Committee, VIIth International Symposium on Phototrophic Procaryotes (to be held in Amherst, MA, 07/91).

1990—1991 Co-Chairman, Tenth Summer Symposium in Molecular Biology, The Pennsylvania State University

1990 Panel Member, Dept. of Energy Grants Review

### 1991—2022 Professor of Biochemistry & Molecular Biology, The Pennsylvania State University

1992—1996 Faculty Senator, Eberly College of Science, The Pennsylvania State University

1992 Review Committee for Research Directions, Energy Biosciences Program, Department of Energy

1992 Co-Chairman, 18th Annual Midwest Photosynthesis Meeting, Turkey Run, Indiana (with Dr. J. H. Golbeck).

1992—1997 NIH-GMS Study Section Member, Microbial Physiology (MBC-1) 1992— Member, Center for Biomolecular Structure and Function

### 1992—2022 Ernest C. Pollard Professor of Biotechnology, The Pennsylvania State University

1993 DOE, Panel Member, Workshop on Physiological Ecology of Photosynthesis

1993- Faculty Advisory Board, The Pennsylvania State University Biotechnology Institute 1994 Convener, Structure/Function Relationships in Photosynthesis, Annual meeting of the

American Society of Plant Physiologists, Portland, Oregon (August 1994)

1994 Department of Energy, Program Reviewer for "Electron Transport and Photosynthesis," Argonne National Laboratory (Nov. 14-16, 1994)

1994—2000 Elected member, International Scientific Committee, Intl. Symposium on Phototrophic Prokaryotes

1995 Co-organizer, Vth Workshop on Cyanobacterial Molecular Genetics (Asilomar, CA, July 21-25, 1995)

1995—1997 Chairman, NIH-GMS Study Section Microbial Physiology 1995—2002 American Society for Plant Physiology, Kettering Award Committee 1996—1997 Anniversary Visiting Fellow, The Australian National University

1996—1999 Publications Committee, The International Society of Photosynthesis Research 1999 Dept. of Energy, Energy Biosciences, Grant Review Panel

2000—2001 Chairman, Kettering Award Committee, American Society for Plant Physiology 2000—2015 Schreyer Honors College, Academic Advisor, Microbiology

2000 Vice-Chairman, Gordon Conference, Biophysical Aspects of Photosynthesis 2000 1st Paskarnis-Buchanan Lecturer, Bowling Green State University

2000 Chairman, External Review Committee, Unité de Physiology Microbienne, Institut Pasteur

2001 Co-organizer (with Satoshi Tabata) Workshop on Genomics of Phototrophic Organisms.

March 19-20, 2001 Kazusa DNA Research Institute, Chiba, Japan.

2001 Member, External Review Committee, Dept. of Biology, Texas A&M University

2001 Member, External Review Committee, Plant Biology Program, Arizona State University 2001— Member, Molecular Biochemistry Panel, National Science Foundation

2000—2004 Member, Department of Energy, Energy Biosciences Advisory Council

2002 Chairman, 19^th^ Eastern Regional Photosynthesis Conference, Woods Hole, MA 2002 Chairman, Gordon Conference, Biochemical Aspects of Photosynthesis 2002—2003 Eberly College of Science, Promotion and Tenure Committee

2003— PSU Nucleic Acid and Microarray Advisory Committee, Huck Institute of Life Sciences 2003 National Institutes of Health, National Center for Research Resources, Special Emphasis

Panel, COBRE Research Infrastructure

2004 Member, National Science Foundation—U. S. Dept. of Agriculture, Grant Review Panel, Interagency Microbial Genome Sequencing Program, March 2004.

2004 Member, Dept. of Energy Grant Review Panel, Microbial Genomes Panel, June 2004 2004 Member, Pre-proposal Review Panel, National Science Foundation: Integrative Graduate

Education and Research Traineeship Program, July 2004

2004—2007 Microbiology Group Leader, Dept. Biochemistry and Molecular Biology

2004—2010 International Society for Photosynthesis Research, Geographical Representative for the Americas

2004—2007 International Society for Photosynthesis Research, Publications Committee

2005 Invited Participant, BioSolar Workshop, DOE Basic Energy Sciences (April 18-20, 2005) 2005 Member, Dept. of Energy, Genomes to Life Proposal Panel, April/May, 2005 2005—2010 Member, Editorial Board, ***Journal of Biological Chemistry***

2005 Visiting Fellow, Thermal Biology Institute, Montana State University

2006 Member, NSF-sponsored Research Coordination Network, Yellowstone National Park 2006 Member, Joint Genome Institute, Dept. of Energy, Community Sequencing Program

Review Panel (May 1-2, 2006)

2007 Member, Dept. of Energy, Grant Review Panel, Feb. 4-6, 2007 (Natural Photosynthesis) 2007 Member, Joint Genome Institute, Dept. of Energy, Community Sequencing Program

Review Panel (April 23-24, 2007)

2007—2010 Member, International Organizing Committee, Intl. Congress on Photosynthesis, Beijing, China, August 2010 (Intl. Soc. Photosynthesis Research)

2008 Member, Annual Review Team, DOE BioEnergy Science Center (BESC), Oak Ridge National Laboratory

2009— Research/Adjunct Professor, Department of Chemistry and Biochemistry, Montana State University, Bozeman, MT

2009— Affiliate Member, Thermal Biology Institute, Montana State University

2009 Panelist, Dept. of Energy/Joint Genome Institute, Walnut Creek, CA, May 15, 2009 Community sequencing program (Metagenomics)

2009—2014 Member, JGI/DOE Standing Review Panel, Community Sequencing Program for Microbial Isolates.

2009—2011 Member, User Committee (Advisory), Joint Genome Institute, Dept. of Energy 2009— Member, Board of Directors, Rebeiz Foundation for Basic Research

2010—2011 Editor-in-Chief, ***Frontiers in Microbiology, Microbial Physiology and Metabolism***

2010—2011 Sabbatical leave, Dept. of Chemistry and Biochemistry and Dept. of Land Resources and Environmental Sciences, Montana State University

2011 Member, NASA Astrobiology/Exobiology review panel (February 22-25, 2011) 2011—2013 Member, Steering Committee, Center for Environmental Geochemistry and Genomics 2011— Member, Graduate Faculty, Astrobiology Dual Title Program

2011—2015 Associate Editor, ***Frontiers in Microbial Physiology and Metabolism***

2012 Member, Strategic Planning for Genomic Sciences, DOE Office of Biological and Environmental Research

2013—2018 Visiting Professor, School of Biological Sciences, Singapore Centre for Environmental Life Sciences Engineering (SCELSE), Nanyang Technological University, Singapore

2015—2020 Member, Editorial Board, ***Journal of Biological Chemistry***

2015—2018 Member, Local Organizing Committee, 18^th^ International Symposium on Phototrophic Prokaryotes, Vancouver, Canada; August 2018.

2017 Panel Member, Molecular Biophysics, Division of Molecular and Cellular Biosciences, National Science Foundation

2017—2023 Elected member, International Scientific Committee, International Symposium on Phototrophic Prokaryotes

2017—2018 Member, Local Organizing Committee, International Conference on Microbial Photosynthesis (ISPR Vancouver 2018)

2017—2018 Sabbatical leave, Montana State University: Visiting Fellow, Thermal Biology Institute, Dept. of Chemistry and Biochemistry, and Dept. of Land Resources and Environmental Sciences (Sept.1, 2017 to December 31, 2017)

2021—2022 Member, Selection Committee for the Charles F. Kettering Award, American Society of Plant Biologists

### Society Memberships

Sigma Xi

American Association for the Advancement of Science (AAAS) American Society of Microbiology (ASM)

American Academy of Microbiology (AAM) International Society for Microbial Ecology (ISME) International Society of Photosynthesis Research (ISPR)

American Society for Biochemistry and Molecular Biology (ASBMB) American Society of Plant Biologists (ASPB)

Hawk Migration Association of North America (HMANA)

### Teaching Experience

**Lecture Courses: Thesis Supervision/Laboratory Courses**

BiSci 001 (1 time) Micro 502

Micro 201 (12 times) Micro 512

Micro 476/576 (5 times*) Micro 496

Micro 503 (1 time) MCB 510 (1 time)

MCB 251/251H (1 time) Micro/MCB 600

Plant Physiology 512 (10 times) Genetics 600

PSU 016 (2 times) Micro 010 (1 time)

Plant Physiology 512 (10 times)

Micro 401 (20 times between 1996 to 2019, except in 2010, 2015, 2016, 2017)

MCB 590 (2 times)

BMMB 597A/501 (5 times; 8 lectures on topics in microbial physiology and metabolism) MCB 597B (1 time)

BMMB 598C (5 times)

Instructor, SCELSE Summer Course on biofilm microbial communities; 7 to 13 hours of lecture; (July, 2013, 2014, 2015)

(*taught Micro 476 equivalent (Photosynthesis) once while on sabbatical leave at ETH-Zürich)

Research advisor to 1991 Paul Berg Award Winner as outstanding 2^nd^/3^rd^ year student in Molecular Biology in the Eberly College of Science, Wendy M. Schluchter

Research advisor to 1994 Paul Berg Award Winner as outstanding 2^nd^/3^rd^ year student in Molecular Biology in the Eberly College of Science, Tanja Gruber

Research advisor to 2003 Alumni Association Dissertation Award Winner ($5000 prize), Yumiko Sakuragi

Research advisor to 2004 Wedler Outstanding Doctoral Dissertation Award Winner, Dr. Yumiko Sakuragi, Dept. of Biochemistry and Molecular Biology.

Advisor to Dr. Yumiko Sakuragi, who won a Marie Curie International Postdoctoral Fellowship from the European Union for 2006-2008.

Advisor to Dr. Yumiko Sakuragi, who won a Steno grant from the Danish Natural Science Research Council, establishing her as an Assistant Professor at the University of Copenhagen.

Research advisor to 2006 Alumni Association Dissertation Award Winner ($5000 prize), Dr. Julia A. Maresca

Research advisor to 2012 Alumni Association Dissertation Award Winner ($5000 prize), Dr. Zhenfeng Liu

Research advisor to 2012 Wedler Outstanding Doctoral Dissertation Award Winner, Dr. Zhenfeng Liu, Dept. of Biochemistry and Molecular Biology.

Research advisor to the 2012 McCarl Fellowship winner in the Dept. of Biochemistry and Molecular Biology ($2500 prize), Mr. Shuyi Zhang

Research advisor to 2012 Paul Berg Award Winner as the outstanding 2^nd^/3^rd^ year student in Molecular Biology in the Eberly College of Science, Mr. Shuyi Zhang ($1000 prize).

Research advisor to Mr. Adam Peréz, who was selected for the NSF-sponsored CarbonEarth assistantship/teaching fellowship program at PSU for 2012-2013 (one year NSF assistantship); Adam was selected for a second year for 2013-2014), and became instructor coordinator in (2014-2015), thereby gaining 3 years of support for his degree work. Adam also recently received an outreach grant from PARC and assisted in teaching an outreach session for middle school teachers in St. Louis and surroundings.

Research advisor to Mr. Shuyi Zhang, who was named a Distinguished Graduate Scholar by the China Scholarship Council (2013-2014).

Research advisor to Dr. Shuyi Zhang, winner of the 2016 Wedler Outstanding Doctoral Dissertation Award, Dept. of Biochemistry and Molecular Biology.

Research advisor to Mr. Ming-Yang Ho, winner of the 2016 Paul Berg Prize as the outstanding 2^nd^/3^rd^ year student in Molecular Biology in the Eberly College of Science ($1000 prize).

Research advisor to Dr. Adam Peréz, who won a prestigious Marie Curie Postdoctoral Fellowship from the European Union. He will use this 2-year fellowship for studies in the laboratory of Dr. Klaas

J. Hellingwerf at the University of Amsterdam, The Netherlands.

Research Advisor to Mr. Nathan Soulier, 2020 runner-up ($500 award) Simpson Award for Innovative Research and runner-up for the McCarl Fellowship in 2020 ($500 award).

# University Service

### Committee Assignments: Department of Biochemistry and Molecular Biology (BMB)

1981—1982 Microbiology Program, Candidacy Committee 1982—1983 Microbiology Program, Candidacy Committee, Chairman 1983—1984 Departmental Committee, Research/Resources Committee

1986—1988 Department Committee, Graduate Student Recruiting and Admissions 1988—1989 Departmental Faculty Search Committee (Chairman, prokaryotic subcommittee) 1988—1989 Promotion and Tenure, Dept. of Molecular and Cell Biology

1990—1991 Promotion and Tenure, Dept. of Molecular and Cell Biology 1990—1991 Departmental Faculty Search Committee

1991—1992 Departmental Search Committee for Endowed Faculty 1991—1992 Chairman, Departmental Promotion and Tenure Committee 1992—1996 Chairman, Departmental Search Committee for Endowed Faculty 1992—1993 Candidacy Exam Committee, Dept. of Molecular and Cell Biology

1992—1993 Promotion and Tenure Committee, Dept. of Molecular and Cell Biology 1993—1995 Graduate Student Recruiting, Dept. of Biochemistry and Molecular Biology 1993—1994 Departmental Faculty Search Committee (Structure/Function Subcommittee) 1995—1996 Promotion and Tenure Committee, Dept. of Biochemistry and Molecular Biology 1995—2004 Endowed Faculty Search Committee, Dept. of Biochemistry and Molecular Biology 1997—1998 Promotion and Tenure Committee, Dept. of Biochemistry and Molecular Biology 1999—2000 Graduate Affairs Committee

1999—2014 Honors Advisor, Microbiology, Schreyer Honors College

1999—2002 Chairman, Faculty Search Committee, Microbial Physiologist Position

1999—2001 *Ad hoc* Faculty Search Committee, X-ray crystallographer, Dept. of Biochemistry and Molecular Biology

2000—2002 Chairman, Faculty Search Committee, Prokaryotic Development (LSC/IBIOS) 2001—2002 Promotion and Tenure Committee, Dept. of Biochemistry and Molecular Biology 2003—2004 Chairman, Faculty Search Committee, Molecular/environmental microbial ecology 2004—2005 Promotion and Tenure Committee, Dept. of Biochemistry and Molecular Biology 2004—2005 Chairman, Faculty Search Committee, Microbiology

2004—2007 Department Head Advisory Committee, Microbiology 2004—2007 Microbiology Interest Group Leader

2005—2006 Promotion and Tenure Committee, Dept. of Biochemistry and Molecular Biology 2007—2008 Promotion and Tenure Committee, Dept. of Biochemistry and Molecular Biology 2007—2009 Faculty Search Committee, Dept. of Biochemistry and Molecular Biology 2009—2010 Graduate Affairs Committee, Dept. of Biochemistry and Molecular Biology

2010—2016 Peer Teaching Evaluation Committee, Dept. of Biochemistry and Molecular Biology 2011—2013 Graduate Affairs Committee, Dept. of Biochemistry and Molecular Biology 2011—2013 Faculty Search Committee, Dept. of Biochemistry and Molecular Biology

2013—2014 Chairman, Microbiology Search Committee, Dept. Biochemistry and Molecular Biology 2013—2014 Chairman, Peer Teaching Evaluation Comm., Dept. Biochemistry & Molecular Biology 2015—2016 Honors Committee, Dept. of Biochemistry and Molecular Biology

2015—2016 Post-tenure Review, Dept. of Biochemistry and Molecular Biology

2018—2019 Peer Teaching Evaluation Committee, Dept. of Biochemistry and Molecular Biology 2018—2019 Faculty Search Committee, Dept. of Biochemistry and Molecular Biology 2018—2019 Safety Committee, Dept. of Biochemistry and Molecular Biology

2019—2020 Microbiology Search Committee, Dept. of Biochemistry and Molecular Biology 2019—2021 Promotion and Tenure Sub-Committee, Dept. of Biochemistry and Molecular Biology 2021—2022 Post-tenure Review (Chairperson), Dept. of Biochemistry and Molecular Biology

### Committee Assignments: Eberly College of Science (ECoS)

1984—1986 College of Science, Courses of Study Committee

1985—1986 College of Science, Marker Lectures Speaker Selection Committee 1986—1987 College of Science, Paul Berg Prize Selection Committee

1986—1987 College of Science, Marker Lectures Speaker Selection Committee (Chairman) 1987—1988 Biotechnology Institute, Faculty Search Committee

1991—1992 Eberly College of Science, Marker Lectures Selection Committee (Chairman) 1994—1995 Eberly College of Science Promotion and Tenure Committee

1997—1998 Eberly College of Science Sabbatical Proposal Review Committee 2002—2003 Eberly College of Science, Promotion and Tenure Committee

2021—2022 Eberly College of Science, Review Committee for Endowed Chairs and Professorships

### Committee Assignments: University

1992—1996 Faculty Senate, (Member, University Libraries and Research Committees) 1999—2014 Honors Advisor, Microbiology, Schreyer Honors College

2003—2022 Advisory Committee, PSU Nucleic Acid and Microarray Facility, Huck Institutes for the Life Sciences (now Genomics Core Steering Committee)

2005—2007 Energy Task Force, The Pennsylvania State University, Office for Research

2015—2022 Advisory Committee, Proteomics and Mass Spectrometry Facility, Huck Institutes for the Life Sciences

2016 Microbiome Executive Planning Committee, College of Agriculture and Huck Institutes for the Life Sciences

## Graduate Student Committees (86 total)

### The Pennsylvania State University

| Mary Balovich | No degree | Microbiology |
| --- | --- | --- |
| Eberhard Essich | Ph. D. | Microbiology |
| David Vandenbergh | Ph. D. | Biochemistry |
| Vikram Roongta | M. Sc. | Chemistry |
| Randy Murphy | Ph. D. | Molecular and Cell Biology |
| Amanda Cantrell | Ph. D. | Microbiology |
| Gail Gasparich | Ph. D. | Microbiology |
| Veronica Stirewalt | M. Sc. | Genetics |
| Carol W. Maddox | Ph. D. | Microbiology |
| Jiann-Hwa Chen | Ph. D. | Microbiology |
| Aprile Pilon | Ph. D. | Molecular and Cell Biology |
| James M. Dubbs | Ph. D. | Genetics |
| David Szymkowski | Ph. D. | Molecular and Cell Biology |
| Jianhui Zhou | Ph. D. | Molecular and Cell Biology |
| Jeffrey Carter | Ph. D. | Microbiology |
| Wilfredo Hernandez | Ph. D. | Molecular and Cell Biology |
| Laurie F. Caslake | Ph. D. | Molecular and Cell Biology |
| Wendy M. Schluchter | Ph. D. | Molecular and Cell Biology |
| Carol Baker | Ph. D. | Genetics |
| Soohee Chung | Ph. D. | Biochemistry and Molecular Biology |
| Ross Whitwam | Ph. D. | Biochemistry and Molecular Biology |
| Hin-Cheung Lee | Ph. D. | Biochemistry and Molecular Biology |
| Paula J. Beyer | Ph. D. | Chemistry |
| Kristen McLaughlin | Ph. D. | Chemistry |
| Pete Long | M. Sc. | Biochemistry and Molecular Biology |
| Tanja Maria Gruber | Ph. D. | Biochemistry and Molecular Biology |
| John Chicca | Ph. D. | Biochemistry and Molecular Biology |
| Chris Nomura | Ph. D. | Biochemistry and Molecular Biology |
| Kathleen M. Scott | Ph. D. | Biology |
| Siyang Zheng | Ph. D. /transferred | Biochemistry and Molecular Biology |
| Sara Parkin | M. Sc. | Biochemistry and Molecular Biology |
| Mikhail Antonkine | Ph. D. | Biochemistry and Molecular Biology |
| George Heine | No degree/transferred | Biochemistry and Molecular Biology |
| Hong Zhang | No degree/transferred | Biochemistry and Molecular Biology |
| Elham Behshed | Ph. D. | Biochemistry and Molecular Biology |
| Rachelle Wood | No degree/transferred | Biochemistry and Molecular Biology |
| Boris Zybailov | Ph. D. | Biochemistry and Molecular Biology |
| Pallavi Pandit | No degree (deceased) | Biochemistry and Molecular Biology |
| Tao Wang | Ph. D. | Plant Physiology |
| Joel E. Graham | Ph. D. | Biochemistry and Molecular Biology |
| Yumiko Sakuragi | Ph. D. | Biochemistry and Molecular Biology |
| Hui Li | Ph. D. | Biochemistry and Molecular Biology |
| Rama Balasubramanian | Ph. D. | Biochemistry and Molecular Biology |

Rachel Cohen Ph. D. Biochemistry and Molecular Biology

Julia Ann Maresca Ph. D. Biochemistry and Molecular Biology

Aline Gomez Maqueo-Chew Ph. D. Biochemistry and Molecular Biology

Mark Heinnickel Ph. D. Biochemistry and Molecular Biology

David Vuletich Ph. D. Chemistry

Heather E. Jordan No degree Transferred to Astrobiology

Bhramara Tirupati Ph. D. Biochemistry and Molecular Biology

Zhao Jin M. Sc. Biochemistry and Molecular Biology

Yingxian Wu M. Sc. Biochemistry and Molecular Biology

Fang Shen M. Sc. Plant Physiology

Gretchen Koch M. Sc. Biochemistry and Molecular Biology

Nithya Srinivasan Ph. D. Biochemistry and Molecular Biology

Jay H. Russell Ph. D. Biochemistry and Molecular Biology

Bharat Jagannathan Ph. D. Biochemistry and Molecular Biology

Mingyu Wang Ph. D. Biochemistry and Molecular Biology

Yu Xu Ph. D. Biochemistry and Molecular Biology

Olubolaji Akinterinwa Ph. D. Chemical Engineering

Matthew D. Fodor Ph. D. Chemistry

Amaya M. Garcia Costas Ph. D. Biochemistry and Molecular Biology

Wei Xiong M. Sc. Biochemistry and Molecular Biology

Yili Sun M. Sc. Biochemistry and Molecular Biology

Steven P. Romberger Ph. D. Biochemistry and Molecular Biology

Zhengfeng Liu Ph. D. Biochemistry and Molecular Biology

Simon Yeung M. Sc. Biochemistry and Molecular Biology

Sijie Hao M. Sc. Biochemistry and Molecular Biology

Matthew Pagel No degree Biochemistry and Molecular Biology

Fei Gan Ph. D. Biochemistry and Molecular Biology

Michael G. Gresock Ph. D. Biochemistry and Molecular Biology

Junlei Sun Ph. D. Biochemistry and Molecular Biology

Shuyi Zhang Ph. D. Biochemistry and Molecular Biology

Amanda M. Applegate M. Sc. Chemistry

John Michael Gorka Ph. D. Chemistry

Becky McCauley Ph. D. Geosciences and Astrobiology

Adam A. Pérez Ph. D. Biochemistry and Molecular Biology

Bryan H. Ferlez Ph. D. Biochemistry and Molecular Biology

Karim Walters Ph. D. Chemistry

Karla Piedl M. Sc. Biochemistry and Molecular Biology

Ming-Yang Ho Ph. D. Plant Biology

Jennifer L. Thweatt Ph. D. Biochemistry and Molecular Biology

Nathan Soulier Ph. D. Biochemistry and Molecular Biology

Tristan M. Cofer Ph. D. Entomology Emily Featherston Ph. D. Chemistry

John G. Gardiner IV Ph. D. Biochemistry and Molecular Biology

### Advisees and Students from Other Universities or Programs (14 total)

Thierry Damerval Ph. D., Institut Pasteur and University of Paris, Microbiology; Paris,

France (Dr. Nicole Tandeau de Marsac, advisor). External examiner.

David Laudenbach Ph. D., University of Toronto, Dept. of Botany; Toronto, Canada (Dr. Neil Strauss, advisor). Committee member.

John Jenkins Ph. D., McQuarie University, Dept. of Biological Sciences, Sydney,

Australia (Dr. Roger Hiller, advisor). Ph. D. thesis external examiner.

Manuel Glauser Ph. D. (1992), Eidgenössische Technische Hochschule, Institut für Molekularbiologie und Biophysik, Zürich Switzerland (Dr. Herbert Zuber, advisor; DAB, co-advisor)

Alicia Esteban Ph. D. (1993), Eidgenössische Technische Hochschule, Institut für Molekularbiologie und Biophysik, Zürich, Switzerland (Dr. Herbert Zuber, advisor; DAB, co-advisor)

Tao Li Ph. D. (2005), College of Life Sciences, Peking University, Beijing, China (Jindong Zhao, advisor; DAB, co-advisor)

Tuo Shi Rutgers University (Dr. Paul Falkowski, advisor). Committee member.

Yue-hui Zhu Ph. D. (2010), South China Univ. of Technology, Guangzhou, China (DAB, co-advisor)

Christian G. Klatt Ph. D. (2011), Land Resources and Environmental Sciences, Montana State University, Bozeman, MT (Dr. David M. Ward, advisor; DAB, committee member).

Millie T. Olsen M. Sc. (2015) Land Resources and Environmental Sciences, Montana State University, Bozeman, MT (Dr. David M. Ward, advisor; DAB, committee member)

Dan Roizman Ph. D., SCELSE, Nanyang Technological University, Singapore (Dr.

Yehuda Cohen, advisor; DAB, committee member). 2013–2015.

Alistair Laos School of Chemistry, University of New South Wales, Australia. Ph. D. external thesis examiner (2016).

Jason M. Wood Ph. D. (2017) Land Resources and Environmental Sciences, Montana State University, Bozeman, MT (Dr. David M. Ward, advisor; D.A.B., committee member, 2014–2017).

Tristan M. Cofer Ph. D. (2022) Entomology (visiting PSU Ph. D. student)

**Visiting Scientists (9 total)**

| Dr. Nicole Tandeau de Marsac, Institut Pasteur | 09/84—10/84; 07/85—08/85; 1986 |
| --- | --- |
| Dr. D. V. Amla, National Botanical Institute, Lucknow, India | 06/86—12/86 |
| Dr. Klaus Kowallik, Heinrich-Heine-Universität, Düsseldorf | 06/96—07/96 |
| Dr. Carlos Gomez-Lojero, Mexico City, Mexico | 01/99—12/99 |
| Dr. Susan K. Reimer, St. Francis College, Loretto, PA | 06/99—08/99 |
| Dr. Jindong Zhao, Peking University, China | 06/99—08/99 |
| Amaya M. Garcia Costas, Montana State University-Billings | 09/05—12/06 |
| Dr. Wendy M. Schluchter, University of New Orleans | 10/05—11/05; 08/08—11/08 |
| Dr. John W. Peters, Montana State University | 09/09—06/10 |

## Postdoctoral Associates

### (38 total; period at PSU; current or last known position)

**Barbara Illman** 11/83—06/84 Staff Scientist (retired), University of Wisconsin,

USDA/FS Forest Products Lab, Madison, WI

**Robert de Lorimier** 09/83—07/86; 01/89—07/89 Formerly, Senior Research Associate,

Dept. of Biochemistry, Duke University **Gerard Guglielmi** 02/84—06/85 Senior Research Scientist, Pasteur Institute **Jeffrey C. Gingrich** 06/85—06/87 Principal consultant, biotechnology, retired

Halteres Associates, Emeryville, CA

**Erhard Rhiel** 10/87—09/88 Professor, Carl von Ossietzky University,

Oldenberg, Germany

**David H. Lambert** 01/84—12/85 (formally with Dr. S. E. Stevens, Jr.; collaborative

efforts/space-sharing in my lab); Associate Professor of Plant Pathology, University of Maine, Orono, ME

**R. K. Singh** 01/86—12/87 (formally with Dr. S. E. Stevens, Jr.; collaborative

efforts/space-sharing in my lab); Senior Research Officer, Institute for Marine Biosciences, Halifax, Canada

**Jindong Zhao** 01/90—07/93 Professor, and former Assoc. Dean, College of Life

Sciences, Beijing University, Beijing, China **Member, Chinese Academy of Sciences** (2007—); **Director, Hydrobiology Institute, Wuhan** (2007—2018)

**Ulrich Mühlenhoff** 12/91—12/92 Associate Professor, Inst. for Cytobiology and

Cytopathology, Phillips University, Marburg, Germany

| **Gaozhong Shen** | 02/94—01/98 | Research professor, Penn State University |
| --- | --- | --- |
| **Ailsa Carmichael** | 06/94—07/96 | High School Teacher, Northeastern Scotland |
| **Christiane Jakobs** | 07/94—07/96 | Research associate, University of Bonn |
| **Conrad W. Mullineaux** | 08/95—12/95 | Professor, Queen Mary University, London, UK |
| **Toshio Sakamoto** | 11/94—08/00 | Associate Professor of Natural Sciences |
|  |  | Kanazawa University, Kanazawa, Japan |
| **Elena V. Vassilieva** | 09/96—07/01 | Senior research scientist |
|  |  | Emory University School of Medicine |
|  |  | Atlanta, GA |
| **Alexander Ganago** | 01/97—12/98 | Adjunct Asst. Professor and Laboratory Coordinator |
|  |  | University of Michigan, Ann Arbor, MI |
| **Kaori Inoue-Sakamoto** | 05/98—08/00 | Associate Professor of Applied Bioscience |
|  |  | Kanazawa Inst. of Technology, Kanazawa, Japan |
| **Niels-Ulrik Frigaard** | 08/99—09/04 | Associate Professor of Biological Chemistry, |
|  |  | University of Copenhagen, Copenhagen, Denmark |
| **Jürgen Marquardt** | 08/00—08/02 | Scientific Instrument Repair, Karlsruhe, Germany |
| **Tao Li** | 06/05—12/07 | Assistant Professor of Bioinformatics, |
|  |  | Institute for Hydrobiology Wuhan, China |

| **Hui Li** | 02/06—06/06 | Currently: Data Scientist, Facebook; Seattle, WA; |
| --- | --- | --- |
|  |  | Formerly: Senior Research Fellow, Dept. of |
|  |  | Pathology, Univ. of Washington, Seattle, WA |
|  |  | (2006-2015) |
| **Fangqing Zhao** | 06/06—12/07 | Professor, Beijing Institute of Life Science |
|  |  | Chinese Academy of Sciences |
| **Paulo Silva** | 01/06—03/09 | Whereabouts unknown |
| **Richard M. Alvey** | 05/06—07/11 | Assistant Professor of Biology, Illinois Wesleyan |
|  |  | University |
| **Yusuke Tsukatani** | 07/07—08/11 | Tenure-track research scientist, Japan Agency for |
|  |  | Marine-Earth Science & Tech., Yokosuka, Japan |
| **Joel E. Graham** | 01/08—06/08 | Currently: Research Associate, University of  Maryland, School of Medicine, Baltimore, MD |
| **Kajetan Vogl** | 03/08—07/12 | Unaffiliated; currently living in Bamburg, Germany |
| **Zhongkui Li** | 06/08—05/13 | NSERC Fellow, Ottawa, Canada |
| **Marcus Ludwig** | 09/08—05/13 | Senior Scientist, Protein Engineering Group, Bayer- |
|  |  | Cologne, Germany |
| **Allison M. Saunders** | 06/11—05/13 | Asst. Professor of Chemistry, Mansfield University; |
|  |  | July 1, 2019; presently Asst. Prof., Chemistry, |
|  |  | Lycoming College |
| **Zhenfeng Liu** | 02/12—10/12 | Bioinformatics Scientist, Quantgene, Berkeley, CA |
| **Marcus Tank** | 08/11—3/16 | Associate Professor, Tokyo Metropolitan |
|  |  | University; Dec. 2019: Senior Scientist, Leibniz- Institut DSMZ-Deutsche Sammlung von  Mikroorganismen und Zellkulturen GmbH |
| **Vera Thiel** | 01/12—9/15 | Formerly: Associate Professor, Tokyo Metropolitan |
|  |  | University; currently, Senior Scientist, DSMZ- |
|  |  | Deutsche Sammlung von Mikroorganismen und |
|  |  | Zellkulturen Gmb |
| **Chi Zhao** | 05/14—06/16 | Associate professor, Qibebt, Qingdao, China |
| **Fei Gan** | 10/14—07/15 | Formerly, Postdoc, Scripps Institute, San Diego and |
|  |  | Postdoc at Univ. of California, Berkeley; |
|  |  | Currently: Assistant Professor, Wuhan University |
| **Daniel P. Canniffe** | 07/15—12/17 | Lecturer/Asst. Professor, Univ. of Liverpool, UK |
| **Zhihui He** | 10/16—04/19 | Currently: Postdoc, Washington Univ. in St. Louis |
| **Amaya M. Garcia Costas** | 09/17—08/18 | Associate Professor and Chair, Dept. of Biology, |
|  |  | Colorado State University-Pueblo, Pueblo, CO |
| **Ming-Yang Ho** | 07/18—09/18 | Associate Professor, Life Science, National Taiwan |
|  |  | University, Taipei, Taiwan |

## Graduate Students Supervised (48 total): Degree, Year Awarded, Major, Current Position

| **Steven Keating** | No degree | Transferred to PSU Entomology |
| --- | --- | --- |
|  |  | Currently, Senior Instructor, |
|  |  | Biochemistry and Mol. Biology, Penn State |
| **Reid Frederick** | No degree | Transferred to Ohio State University |
|  |  | Research Molecular Biologist, USDA/ARS |
|  |  | Foreign Disease- Weed Science Research Unit, Ft. Detrick, MD |
| **Veronica L. Stirewalt** | No degree | M. Sc., Genetics (completed, except thesis) |
|  |  | (Senior Research Associate, Penn State) |
|  |  | Critical Care Nurse, Mt. Nittany Medical Ctr |
| **Amanda Cantrell** | M. Sc. 1987 | Microbiology; Clinical Pharmacology |
|  |  | Consultant, Eli Lilly, Indianapolis, IN |
| **Randy C. Murphy** | Ph. D. 1989 | Molecular and Cell Biology |
|  |  | **Formerly:** Asst. Prof. Biological Sciences |
|  |  | University of Memphis |
|  |  | **Currently:** Software Engineer, UNISYS |
| **Gail E. Gasparich** | Ph. D. 1989 | Microbiology |
|  |  | **Formerly:** Professor of Microbiology, |
|  |  | Towson University; Co-Director Biology |
|  |  | Graduate Program Director, CSM Women in |
|  |  | Science Program; Asst. Dean, Fisher |
|  |  | College of Science and Mathematics; Acting |
|  |  | Asst. Provost, Towson Univ., MD; Dean, |
|  |  | College of Arts and Sciences, Salem State |
|  |  | University. **Currently:** Provost, & VP for |
|  |  | Academic Affairs, Millersville University |
| **James M. Dubbs** | Ph. D. 1990 | Genetics; Staff Research Scientist, |
|  |  | Laboratory of Biotechnology, Chulabhorn |
|  |  | Research Institute, Bangkok, Thailand |
| **Manuel Glauser** | Ph. D. 1991 | Molecular Biology, ETH, Zürich, Molecular |
|  |  | Biology Corporate Safety and Environ- |
|  |  | mental Protection Officer, Hoffmann- |
|  |  | LaRoche, Basel, Swizerland |
| **Jianhui Zhou** | Ph. D. 1992 | Molecular and Cell Biology |
|  |  | Sr. Scientist, Genentech, San Francisco, CA |
| **Laurie F. Caslake** | Ph. D. 1994 | Molecular and Cell Biology |
|  |  | Professor of Biology |
|  |  | Chairperson); **Currently** Gideon R., Jr. and |
|  |  | Alice L. Kreider Professor of Biology |
| **Wendy M. Schluchter^1^** | Ph. D. 1994 | Dept. of Biology, Lafayette College  Biochemistry and Molecular Biology; |
|  |  | **Currently:** Eurofins Professor of Molecular |
|  |  |  |

|  | | Biology and Chairperson of Dept of  Biological Sciences, Univ. of New Orleans;  **Formerly:** Acting Dean, College of |
| --- | --- | --- |
|  |  | Sciences; NSF Career Award (2002) |
| **Soohee Chung** | Ph. D. 1995 | Biochemistry and Molecular Biology |
|  |  | Research Scientist, Inst. of Biotechnology, |
|  |  | Yuengnam University, Gyeongsan, Korea |
| **William P. Long** | M. Sc. 1996 | Biochemistry and Molecular Biology |
|  |  | Ph. D., Penn State; Last known position: |
| **Tanja M. Gruber^2^** | Ph. D. 1998 | Patent Lawyer & Associate, DLA Piper Biochemistry and Molecular Biology |
|  |  | **Formerly:** Functional Leader of |
|  |  | Biochemistry & Industrial Science, |
|  |  | International Flavors & Fragrances, |
|  |  | Wilmington, DE; **Currently:** Vice President  for Research & Development, Origin Materials, Sacramento, CA |
| **Sara Parkin** | M. Sc. 1998 | Biochemistry and Molecular Biology |
|  |  | (co-advisor with Dr. J. M. Bollinger) |
| **Katja Schlink** | Visiting Ph. D. student | Heinrich-Heine University, Germany |
|  |  | Senior Scientist, Forest Genetics, Technical |
|  |  | University, Munich, Germany |
| **Silvia Miletti** | Visiting Ph. D. student | Univ. Undine, Italy; Whereabouts unknown |
| **Christopher T. Nomura** | Ph. D. 2001 | Biochemistry and Molecular Biology |
|  |  | **Formerly:** Professor of Chemistry, and Vice |
|  |  | President for Research, SUNY, College of |
|  |  | Environmental Science and Forestry, |
|  |  | Syracuse, NY; **Currently:** Vice President |
|  |  | for Research and Economic Development, |
|  |  | University of Idaho, Moscow, ID |
| **Rachelle Wood** | No degree | Biochemistry and Molecular Biology |
|  |  | Whereabouts unknown |
| **George Heine** | No degree | Transferred to Ohio State as Ph. D. student |
|  |  | Whereabouts unknown |
| **Heather E. Jordan** | Transferred/no degree | Transferred to PSU—Astrobiology |
| **Yumiko Sakuragi^,3,4,5,6^** | Ph. D. 2004 | Whereabouts unknown  **Formerly:** Associate Professor and Group |
|  |  | Leader, Section of Molecular Plant Biology |
|  |  | and Biotechnology, Univ. of Copenhagen, |
|  |  | Copenhagen, Denmark; **Currently:** Senior |
|  |  | Scientist, FMC Coroporation, Copenhagen |
| **Tao Li (co-advisor)** | Ph. D. 2005 | Bioinformatics and Biochemistry |
|  |  | College of Life Sciences, Peking University |
|  |  | Currently: Assistant Professor, Institute for |
|  |  | Hydrobiology, Wuhan, China |
| **Rama Balasubramanian** | Ph. D. 2005 | Biochemistry and Molecular Biology |

|  | | Lead Chemist, Nalco Champion, Sugar |
| --- | --- | --- |
|  |  | Land, TX |
| **Hui Li** | Ph. D. 2006 | Biochemistry & Molecular Biology |
|  |  | **Formerly:** Senior Res. Fellow, Dept. of |
|  |  | Pathology, University of Washington, |
|  |  | Seattle, WA (American Liver Foundation |
|  |  | Fellow); 2006-2015). |
|  |  | **Currently:** Research Scientist, Altius Inst. |
| **Julia Ann Maresca^7^** | Ph. D. 2007 | for Biomedical Sciences, Seattle, WA  Biochemistry and Molecular Biology  Currently: Associate professor of  At SUNY College of New York |

**Aline Gomez Maqueo Chew** Ph. D. 2007 Biochemistry and Molecular Biology

Postdoc: Ohio State University;

Faculty member and Research coordinator, Health Sciences, School at UVM Campus Hermosillo de Investigación, Escuela de Ciencias de la Salud, Universidad del Valle de México, Hermosillo, México

**Zhao Jin** M. Sc. 2007 Biochemistry and Molecular Biology

Ph. D., Microbiology, Cornell University

**Currently**: Scientist, µBiome

**Joel E. Graham** Ph. D. 2008 Biochemistry and Molecular Biology

Formerly: Research Associate, University of Maryland, School of Medicine, Baltimore, MD; Currently: Senior scientist, AstraZeneca

**Yingxian Wu** M. Sc. 2008 Biochemistry and Molecular Biology

Ph.D., Agricultural, Environmental Regional Economics, Penn State University

**Fang Shen** M. Sc. 2008 Plant Biology

Scientist II, Abbvie Inc., Cambridge, MA

**Hao Fan** No degree Whereabouts unknown

**Yu Xu** Ph. D. 2010 Biochemistry and Molecular Biology **Currently:** Senior Scientist, Roche Molecular Diagnostics, San Francisco, CA

**Amaya M. Garcia Costas** Ph. D. 2010 Biochemistry and Molecular Biology

**Formerly** Postdoc, Montana State University and Penn State University, **Currently:** Associate Professor and Chair, Dept. of Biology, Colorado State University-Pueblo, Pueblo, CO

**Yue-hui Zhu (co-advisor)** Ph. D. 2010 (2009-2010) Biotechnology

South China University of Technology

|  |  | **Currently:** Postdoctoral Fellow, |
| --- | --- | --- |
|  |  | Washington University in St. Louis |
| **Wei Xiong** | M. Sc. 2011 | Biochemistry and Molecular Biology |
|  |  | Research Scientist, Protein Chemistry, |
|  |  | Regeneron, Tarrytown, NY |
| **Steven P. Romberger** | Ph. D. 2011 | Biochemistry and Molecular Biology, |
|  |  | **Formerly**: Visiting Asst. Prof., Dept. of |
|  |  | Chemistry, Bucknell Univ.; Currently |
|  |  | Associate Prof. of Chemistry and Director of |
| **Zhenfeng Liu^8,9^** | Ph. D. 2012 | Biochemistry Program, Hiram College  Biochemistry and Molecular Biology |
|  |  | **Formerly:** Bioinformaticist, University of |
|  |  | Southern California, Los Angeles, CA |
|  |  | **Currently:** Bioinformatics Scientist, Zymo |
|  |  | Research Corp, Irvine, CA |
| **Yi (Julia) Zhu** | No degree | Left PSU for personal reasons |
| **Simon Yeung** | M. Sc. 2013 | Biochemistry and Molecular Biology |
|  |  | Res. Associate, Regeneron, Tarrytown, NY |
| **Matthew D. Pagel** | No degree | **Currrently:** Staff Research Informatics |
|  |  | Analyst, Emory University, Atlanta, GA |
|  |  | **Formerly:** Dept. of Wildlife, Fish, and |
|  |  | Conservation Biology; University of |

California-Davis, Davis, CA

**Fei Gan** Ph. D. 2014 Biochemistry and Molecular Biology; **Formerly:** Postdoc, Scripps Research Inst., San Diego; Postdoc, Univ. California- Berkeley; **Currently:** Assist. Professor of Biology, Wuhan University

**Shuyi Zhang^10,^ ^11,^ ^15,^ ^16,^ ^21^** Ph. D. 2015 Biochemistry and Molecular Biology

**Formerly:** Postdoc, Massachusetts Institute of Technology; 2018: **Currently:** Assistant Professor (2019), Pharmaceutical Sciences, Tsinghua University, Beijing, China

**Adam A. Pérez^12,^ ^13,^ ^17,^ ^18,^ ^19^** Ph. D. 2016 Biochemistry and Molecular Biology

**Formerly:** Postdoc, Univ. Louisville; 2^nd^ Postdoc, Univ. Amsterdam; **Currently:** Biologist, US Geological Survey, Gainesville, Florida

**Ming-Yang Ho^20^** Ph. D. 2018 Plant Biology; **Currently:** Associate

Professor of Life Science, Inst. of Plant Biology, National Taiwan University Technology; Taiwanese Ministry of Science and Einstein Program Awardee

**Jennifer L. Thweatt^14^** Ph. D. 2019 Biochemistry & Molecular Biology, and

Astrobiology; **Currently**: Biocatalyst Engineer, Genomatica, San Diego, CA

**Nathan T. Soulier^22^** Ph. D. 2021 Biochemistry and Molecular Biology

**Currently:** Postdoctoral associate, University of California San Diego (with Susan S. Golden); Previously: Quality Control Specialist, Trilink BioTechnologies, San Diego, CA

1Dr. Wendy M. Schluchter won the Paul Berg Prize as the Outstanding 2^nd^/3^rd^ year student in Molecular Biology in the Eberly College of Science ($1000 prize) in 1991.

2Dr. Tanja M. Gruber won the Paul Berg Prize as the Outstanding 2^nd^/3^rd^ year student in Molecular Biology in the Eberly College of Science ($1000 prize) in 1994.

3Dr. Yumiko Sakuragi won the 2003 PSU Alumni Association Dissertation Award ($5000 prize)

4Dr. Yumiko Sakuragi won the 2004 Wedler Outstanding Doctoral Dissertation Award, Biochemistry and Molecular Biology

5Dr. Yumiko Sakuragi won a Marie Curie International Postdoctoral Fellowship from the European Union for 2006—2008

6Dr. Yumiko Sakuragi was awarded a Steno grant by the Danish Natural Science Research Council, establishing her as an Assistant Professor at the University of Copenhagen

7Dr. Julia A. Maresca won the 2006 PSU Alumni Association Dissertation Award ($5000 prize)

8Dr. Zhenfeng Liu won the 2012 PSU Alumni Association Dissertation Award ($5000 prize)

9Dr. Zhenfeng Liu won the 2012 Wedler Outstanding Doctoral Dissertation Award, Biochemistry and Molecular Biology

10Mr. Shuyi Zhang was selected as the 2012 McCarl Fellowship winner in the Dept. of Biochemistry and Molecular Biology ($2500 prize)

11Mr. Shuyi Zhang was selected as the 2012 Paul Berg Prize winner in the Eberly College of Science ($1000 prize)

12Adam Pérez was selected as for the NSF-sponsored CarbonEarth assistantship program at PSU for 2012-2013 (one-year NSF assistantship).

13Adam Pérez was selected again for the NSF-sponsored CarbonEarth assistantship program at PSU for 2013-2014 (2nd year-long NSF assistantship).

14Jennifer L. Thweatt received full support to attend a summer course in microbial ecology at Nanyang Technical University, Singapore (2012)

15Mr. Shuyi Zhang was named a Distinguished Graduate Student by the China Scholarship Council for 2013-2014

16Dr. Shuyi Zhang won the 2016 Wedler Outstanding Doctoral Dissertation Award, Biochemistry and Molecular Biology.

17Dr. Adam Pérez was selected again for the NSF-sponsored CarbonEarth assistantship program at PSU for 2014-2015 (3rd year-long NSF assistantship). He served as graduate student administrator of

the program and was responsible for scheduling and training of student participants in the program. He also ran a workshop for teachers at PSU.

18The Ph. D. thesis and defense of Dr. Adam Pérez was uniformly scored as “outstanding” by his thesis committee.

19Dr. Adam Peréz won a prestigious Marie Skladowska-Curie Global Postdoctoral Fellowship from the European Union. He will use this 2-year fellowship for studies in the laboratory of Dr. Klaas J. Hellingwerf at the University of Amsterdam, The Netherlands.

20Mr. Ming-Yang Ho won the 2016 Paul Berg Prize ($1000 prize) as outstanding 2^nd^/3^rd^ year student in molecular biology. He also received a travel/scientific exchange fellowship to go to St. Louis to work in the laboratory of Dr. Robert E. Blankenship at Washington University in St. Louis. In 2019 he received a 5-year Award from the Einstein Program from the Taiwanese Ministry of Science and Technology

21Dr. Shuyi Zhang received a Thousand Talents Young Scholar Award in 2019

22Mr. Nathan Soulier was selected as 2020 runner-up ($500 award) for the Simpson Award for Innovative Research and runner-up ($500 award) for the Richard and Norma McCarl Graduate Scholarship.

### Undergraduate Honors Students (with Thesis) Supervised (16 total)

| **Julie Y. Farley (Reuther)** | B. Sc. with honors (1992) | Microbiology; Ph. D., University of North Carolina (1999); postdoc, Lineberger Comprehensive Cancer Center, U. N. C. |
| --- | --- | --- |
| **Nicole Bianco** | B. Sc. with honors (1999) | Biochemistry and Molecular Biology  Ph. D., Case Western Reserve University Postdoctoral fellow, Dept. of Microbiology and Molecular Genetics, University of Pittsburgh; Current: Clinical Research Scientist, at Zoll Lifecor |
| **Matthew Gerstberger** | B. Sc. with honors (2001) | Biochemistry and Molecular Biology  M.D., Penn State University (Hershey); Current: Family Practice, Pennsburg, PA |
| **1Kirstin J. Milks** | B. Sc. with honors (2002) | Microbiology; Ph. D., Department of Biochemistry, Ph. D., Stanford University;  M. A. in Education, Stanford University; currently Natl. Board Certif. Teacher in AP Biology & Earth Science, Bloomington, IN |
| **Kelly D. Elder** | B. Sc. with honors (2002) | Biochemistry and Molecular Biology Formerly: Laboratory technician, Penn State  (with Eric T. Harvill) |

| **Catherine E. Wharry** | B. Sc. with honors (2003) | Biochemistry and Molecular Biology  Ph. D.; Dept. of Animal Biology, U. Pennsylvania; Senior Scientific Director at ETHOS Health Communications, Philadelphia, PA |
| --- | --- | --- |
| **Beth A. Rada** | B. Sc. with honors (2003) | Microbiology; M. Sc., Georgetown Univ.; Executive Director, Federal Government Affairs & Policy at Horizon Pharma, Washington, DC |
| **Colleen E. Yunker** |  | Changed major after a year in 2004 |
| **Sara L. Jubelirer** | B. Sc. with honors (2005) | Biochemistry & Molecular Biology  M. Sc., Michigan State University, Forensic Biology (2008); Current: employed by the  Pennsylvania State Police |

**Christina Cress (Lindenmeyer)** B. Sc. with honors (2006) Biochemistry & Molecular Biology

Research Fellow, National Institutes of Health, Laboratory of Infectious Diseases and U. Penn.; then M. D., Thomas Jefferson University; Currently: Associate Staff Member, Dept. of Gastroenterology and Hepatology, Cleveland Clinic, OH

**Aleksandr Lewicki** Changed major in 2007; M. D. Geriatrics Kaiser Permanente, Los Angeles, CA

**Elizabeth A. Harvie** B. Sc. with honors (2009) Microbiology, Ph. D. student, Dept. of

Bacteriology, University of Wisconsin; Medical Writer at MedThink SciCom, Durham NC

**Patrick O. Byrne** B. Sc. with honors (2010) Biochemistry & Molecular Biology; Ph. D.;

Molecular Biophysics, Johns Hopkins University; Currently: Postdoc, Univ. of Texas, Austin

**2Chyue Yie Chew** B. Sc. with honors (2012) Zuellig Pharma, Regional Regulatory

|  | | Affairs Officer, Selangor, Malaysia |
| --- | --- | --- |
| **3Yang Liu** | B. Sc. with honors (2014) | Biotechnology; Ph. D. student, Dept. of Biochemistry, Univ. of Wisconsin-Madison |
| **4,5Gavin M. Turner** | B. Sc. with Honors (2019) | Johns Hopkins, Research Associate, 2019- 2021; Currently: University of |

Pennsylvania Medical School; 2022 Walter Byers Scholarship Awardee from NCAA

**1Dr. Kirstin J. Milks** was a Braddock Scholar and then a Fulbright Scholar (2002-2003) and spent a year at Southern Denmark University, Odense Denmark, before starting graduate school in biochemistry and biophysics at Stanford University. She was awarded an NSF Predoctoral Fellowship in 2004 and later held a National Defense Science and Engineering Graduate Fellowship. She was a Hertz Foundation Graduate Fellowship finalist in 2004. After earning a M. A. in Education at Stanford, she became a board-certified high school biology/earth sciences teacher in a college setting, teaching at Bloomington, IN.

**2,3Ms. Chyue Yie Chew** and **Mr. Yang Liu** received Summer Undergraduate Research Fellowships (SURF) to conduct Honors Research in Microbiology.

**4Mr. Gavin M. Turner** was the recipient of an Erickson Discovery Grant ($3500) from PSU in 2018

**5Mr. Gavin M. Turner** was a Big Ten Distinguished Scholar from 2017 to 2018 and received a Wayne Duke--Big Ten Postgraduate Fellowship in 2019 ($7500); 2022 Walter Byers Scholarship from the NCAA ($48,000).

### A few other undergraduates of note (*e. g.*, with publications)

| **William B. Snyder** | B. Sc. (1992) | Ph. D. in Molecular Biology with T.  J. Silhavy, Princeton University (1996); Postdoc: Univ. San Diego; Currently: Senior Director, Pfizer |
| --- | --- | --- |
| **Allyson Turner** | B. Sc. (1996) | M. Sc. (1998; Biology) Boise State Univ.; Currently: U. S. Fish and Wildlife Service Biologist, Pacific Region, Boise ID.; mostly studies bird populations |
| **Melissa A. Baker** | B. Sc. (2001);  M. Sc. (BMMB, 2004) | Glaxo-Smith-Kline |
| **Jill M. See** | (B. Sc. 2001, Juniata) | Summer NSF REU student; Ph. D., Neuroscience, U. Penn., (2007); Postdoc,  Drexel Univ. College of Medicine; |

Currently: Assoc. Director of Medical Publications, Sage Therapeutics, Philadelphia, PA

| **Nicole R. Dodd** | B. Sc. (2003) | Immunofluorescence lab technician |
| --- | --- | --- |
| **Ginny D. Voigt** | B. Sc. (2003) | Univ. of Pittsburgh Medical Center  Manufacturing Supervisor, Merck |

| **Kathleen C. Prins** | B. Sc. (2003) | Ph. D., Microbiology; Mt. Sinai School of Medicine; Postdoc, NYU Medical School, Dept. of Pathology  Currently: unknown |
| --- | --- | --- |
| **Jessica M. Mann** | B. Sc. (2007, Juniata) | Summer NSF REU student  NIH Undergraduate Scholarship; Ph. D. in microbiology, New Jersey Medical School, Newark, NJ; Currently: Laboratory of Systems Biology, NIH |
| **Tiing Tiing Chua**  **Daniel Shapiro** | B. Sc. (2013)  B. Sc. (2020) | Undergrad Researcher; Formerly: M. Sc., Molecular Virology, Univ. of Alberta; Currently: Research Officer at Clinical Research Center, Sibu Hospital, Sarawak, Malaysia  Veterinary School, Univ. of California- |
|  |  | Davis (exp. Degree: 2024) |

### Research Assistants (13 total)

| **James M. Dubbs** | Research Assistant | 1983—1986 | Ph. D. (see above) |
| --- | --- | --- | --- |
| **Veronica L. Stirewalt** | Research Assistant | 1984—1989 | Critical Care Nurse (Mt. Nittany |
|  |  |  | Medical Center; retired) |
| **Søren Persson** | Research Associate | 1998—2001 | Ph. D., Dept. of Bacteriology, Statens Serum Institute, Copenhagen |
| **Suzanne Kjær Christensen** | Research Assistant | 1999—2001 | Patent Officer, Copenhagen, DK |
| **Katja Kornetzky** | Research Assistant | 2000—2002 | Consultant, EU Projects, Fraunhofer |
|  |  |  | Inst. for Optronics, System Technology and |
|  |  |  | Image Exploitation, Karlsruhe, Germany |
| **Tao Li** | Research Assistant | 2003—2005 | (Ph. D., see above) |
| **Amaya M. Garcia Costas** | Research Assistant | 2005—2006 | Ph. D. student (2007 to 2010) |
|  |  |  | (Ph. D., Oct. 2010; postodoc, PSU |
| **Chunli Wu** | Research Assistant | 2009—2010 | GlaxoSmithKline |
| **Misato Tsukatani** | Research Assistant | 2009—2010 | Housewife and mother |
| **Lili Zhang** | Research Assistant | 2011—2012 | Currently research assistant, U. |
|  |  |  | Massachusetts-Amherst |
| **Iris Hvanndal** | Research Assistant | 2011—2013 | Res. Assistant; Currently, mother |
|  |  |  | and housewife |

**Scott Gay** Research Assistant 2013—2014 Research Asst.; West Virginia

School of Osteopathic Medicine; resident physician specializing in Emergency Medicine, Geisinger Medical Center, Danville, Pennsylvania

**Yang Liu** Research Assistant 2013—2015 Research Asst.; Dept. Biochemistry,

University of Wisconsin

**Senior Research Associates/Research Professors (3 total)**

| **Veronica L. Stirewalt** | 1989—1998 | Critical Care Nurse, Mt. Nittany Medical Center, State College, PA: retired |
| --- | --- | --- |
| **Dr. Gaozhong Shen** | 01/99—03/2011  04/2013—2023 | Research Professor (prev. Assist. & Assoc.) [Senior Scientist, Essentient, Cambridge, MA, 04/2011 - 03/2013] |
| **Dr. Carol S. Baker** | 09/17— 06/21 | Associate Research Professor (retired) |

# Invited talks, seminars, lectures, and meetings attended

### 2024

1. Invited keynote speaker and particpant, Chemistry and Biology of Tetrapyrroles Gordon Research Conference, July 14-19, 2024. Declined due to retirement and medical situation.

### 2023

1. Seminar ERC “PhotoRedesign” Synergy consortium: “*How terrestrial cyanobacteria sense, respond to, and utilize far-red light for oxygenic photosynthesis.”* December 5, 2023.

### 2022

1. Invited speaker and participant, ASM Microbe, Washington, D.C. June 10, 2022. *“Life on the far-red-edge of darkness: how terrestrial cyanobacteria sense, respond to, and utilize far-red light for oxygenic photosynthesis.”* Unable to attend due to Covid Pandemic and because of medical situation.
2. Invited speaker and participant, 17^th^ International Symposium on Phototrophic Prokaryotes, August 21-25, 2022, Liverpool, United Kingdom. Unable to attend due to Covid Pandemic and because of medical situation. Dr. Chris Gisriel replaced me.
3. Discussion Leader and speaker, Session on Light Harvesting and its Regulation in Prokaryotic Systems, 18^th^ International Congress on Photosynthesis Research, Rotorua, New Zealand. Unable to attend due to Covid Pandemic and because of medical situation.

### 2021

1. Department of Biochemistry and Molecular Biology, Faculty Lunchtime Seminar, March 2, 2021. “*An illuminated life: my 50-year affair with chlorophototrophs—mentors, students, postdocs, collaborators and friends (1972-2021)*.”
2. Juniata Valley Audubon Society, April 20, 2021. “*Hanging on by claw and talon: endangered raptors and charismatic megafauna of southern Africa*.”

### 2020

1. Juniata Valley Audubon Society, Spring Banquet, Keynote speaker. April 22, 2020. *“A pictorial tour of South African fauna: critically endangered and hanging on by claw and talon.”* Postponed until April 2021 due to Corona Virus.
2. Invited keynote speaker, 2020. Light-harvesting workshop 2020, Sudima Hotel, Rotorua, New Zealand. July 30 to August 1, 2020. *“A short history of* Chlorobium *vesicles: chlorosomes.”* Postponed until August 2022 due to coronavirus.
3. Discussion Leader and speaker, Session on Light Harvesting and its Regulation in Prokaryotic Systems, 18^th^ International Congress on Photosynthesis Research, Rotorua, New Zealand. Postponed until August 6 to 12, 2022 due to coronavirus.
4. Center for Marine and Environmental Technology, University of Maryland School of Medicine (host Frank Robb), September 09, 2020. *“Life on the (far-) red-edge of darkness: how terrestrial cyanobacteria sense and respond to far-red light.”*
5. State College Bird Club, September 23, 2020. *“Claws and paws: a pictoral survey of endangered raptors and megafauna of southern Africa.”*

### 2019

1. Seminar speaker, Carnegie Institution of Washington/Stanford University, Department of Plant Biology, May 26, 2019. *“Life on the (far-)red-edge of darkness: how terrestrial cyanobacteria sense and respond to far-red light.”*
2. Invited keynote speaker and participant, 13^th^ Cyanobacterial Molecular Biology Workshop, University of Colorado-Boulder. June 6–9, 2019. *“Life on the (far-) red-edge of darkness: how terrestrial cyanobacteria sense and respond to far-red light.”*
3. Invited symposium speaker and participant, Federation of European Microbiology Societies (FEMS) Congress, July 7–11, 2019, Glasgow, Scotland. Cancelled for medical reasons.
4. Department of Biochemistry and Molecular Biology, Faculty Lunchtime Seminar, September 10, 2019.
5. Invited Speaker and participant, Closing Symposium, Center of Excellence in “Molecular Biology of Primary producers”, Scientific Academy of Finland, Helsinki, Finland. November 6– 9, 2019. Cancelled for medical reasons.

### 2018

1. Invited Keynote Speaker and participant, 27^th^ Western Photosynthesis Conference, January 4-7 2018, Biosphere II, *Algal Research* (1 declined); Oracle, AZ. *“Life on the (far-red)-edge of darkness: how terrestrial cyanobacteria sense and respond to far-red light.”*
2. Invited Speaker and participant, Gordon Research Conference, “*Photosensory Receptors and Signal Transduction,”* Il Ciocco, Barga, Italy. March 4-9, 2018. *“Life on the red edge of darkness: how terrestrial cyanobacteria sense and respond to far-red light.”*
3. Dept. of Biochemistry, University of Nebraska-Omaha, University of Nebraska Medical Center, Omaha, Nebraska. March 12, 2018. *“Life on the (far-red) edge of darkness: how terrestrial cyanobacteria sense and respond to far-red light.”*
4. Dept. of Immunology and Microbiology, Montana State University, March 27, 2017. *“A panoply of phototrophs: metagenomics and metatranscriptomic analyses of the microbial mat community of Mushroom Spring, Yellowstone National Park, WY.”*
5. Invited speaker and participant, ASM Microbe, Atlanta, GA, June 7–11, 2018. D. C. White Research and Mentoring Award Lecture: *“Mushroom Spring: A panoply of phototrophs and their friends.”*
6. Invited speaker and principal investigator, PARC-2, *“Mushroom Spring: a panoply of phototrophs and their friends.”* PARC All-Hands Meeting, Washington University in St. Louis, June 18-21, 2018.
7. Invited symposium speaker, International Conference on Porphyrins and Phthalocyanins ICPP- 10, Munich, Germany, July 1-6, 2018. Declined for medical reasons.
8. Invited speaker and participant, Chemistry and Biology of Tetrapyrroles, Gordon Research Conference, July 15-20, 2018. Salve Regina University, Newport, Rhode Island. *“Life on the (far-red) edge of darkness: how terrestrial cyanobacteria sense and respond to far-red light.”*
9. Keynote Speaker, International Symposium on Phototrophic Prokaryotes, Vancouver, British Columbia, August 5–9, 2018. *“A Life Illuminated: my 47-year affair with chlorophototrophs, students, collaborators and friends (1972-2018)”*
10. Invited Speaker and participant, ISPR Conference on Microbial Photosynthesis, Vancouver, British Columbia, August 9-12, 2018. *“A panoply of phototrophs: metagenomics and metatranscriptomic analyses of the microbial mat community of Mushroom Spring, Yellowstone National Park, WY.”*
11. American Society of Microbiology, Microbiology Club, The Pennsylvania State University, September 10, 2018. *“A panoply of phototrophs: metagenomics and metatranscriptomic analyses of the microbial mat community of Mushroom Spring, Yellowstone National Park, WY.”*
12. Dept. of Biochemistry and Molecular Biology, Lunchtime seminar series, November 27, 2018. *“A panoply of phototrophs: metagenomics and metatranscriptomic analyses of the microbial mat community of Mushroom Spring, Yellowstone National Park, WY.”*

### 2017

1. Eastern Regional Photosynthesis Conference, Keynote Speaker, Woods Hole, MA. April 28-30, 2017. *“Photosynthesis on the edge of darkness…Photoacclimation responses of terrestrial cyanobacteria to far-red and low light*.”
2. PARC-2, All-Hands Meeting, Washington University in St. Louis, June 19-22, 2017. *“Photosynthesis on the edge of darkness…Photoacclimation response of terrestrial cyanobacteria to far-red light and Chl* f *synthesis.”*
3. Invited speaker, session chair, and participant, 13^th^ International Conference on Tetrapyrrole Photoreceptors from Photosynthetic Organisms (ICTPPO 2017), July 9-13, 2017, Chicago, IL. *“Methyl group oxidation in the synthesis of chlorophylls and bacteriochlorophylls: multiple solutions for the same problem in chlorophyll biosynthesis.”*
4. Washington State University, Institute of Biological Chemistry, Pullman, WA. September 21, 2017*. “Photosynthesis on the (red) edge… photoacclimation to far-red and low light in terrestrial cyanobacteria.”*
5. Thermal Biology Institute, Montana State University, Bozeman, MT, November 20, 2017. *“Photosynthesis on the edge of darkness…Photoacclimation response of terrestrial cyanobacteria to far-red light.”*
6. University of Colorado-Boulder, Department of Chemistry and Biochemistry and RASEI, Two talks. December 13-14, 2017. Talk 1: *“Photosynthesis on the (far-red) edge… mechanisms for acclimation to far-red and low light in terrestrial cyanobacteria.”* Talk 2: *“Mushroom Spring: a panoply of phototrophs and their friends.”*

### 2016

1. Invited speaker and attendee, Gordon Research Conference, Photosensory receptors and signal transduction: *Natural & Synthetic Photoreceptor Systems: From Microbes to Man.* Galveston, TX, January 24-29, 2016. Cancelled for medical reasons.
2. Invited seminar speaker, Dept. of Biochemistry and Molecular Biology, University of Nebraska- Omaha Medical Center, February 8, 2016. Cancelled for medical reasons.
3. American Society for Biochemistry and Molecular Biology, ASBMB, 2016 Annual Meeting, April 2-6, 2016. *"Cyanobacteria: new opportunities for photosynthesis and systems biology".* Cancelled for medical reasons.
4. Invited Keynote Speaker and attendee, 12^th^ Cyanobacterial Workshop, Arizona State University, Tempe, AZ. May 19-22, 2016. *“FaRLiP and LoLiP: alternative mechanisms for acclimation to far red and low light in terrestrial cyanobacteria.”* Cancelled for medical reasons.
5. PARC-2, All-Hands Meeting, Washington University in St. Louis, June 21-23, 2016. Cancelled for medical reasons.
6. Invited speaker and participant, European Bioenergetics Conference, July 2-7, 2016, Riva del Garda, Italy. Cancelled for medical reasons.
7. Invited speaker and participant, Gordon Research Conference, Chemistry and Biology of Tetrapyrroles, Newport, RI, July 17-22, 2016. *“FaRLiP and LoLiP: alternative mechanisms for acclimation to far red and low light in terrestrial cyanobacteria.”* Cancelled for medical reasons.
8. Millennium Café, Materials Research Institute, July 27, 2016. The Pennsylvania State University. *“Harnessing the power of photosynthesis.”*
9. Invited Plenary Speaker, 17^th^ International Congress on Photosynthesis, Maastricht, The Netherlands, August 7-12, 2016. *“FaRLiP and LoLiP: alternative mechanisms for acclimation to far red and low light in terrestrial cyanobacteria.”* Cancelled for medical reasons.
10. Dept. of Biochemistry and Molecular Biology, The Pennsylvania State University, September 6, 2016*.* Faculty Lunchtime Seminar series. *“Mechanisms for acclimation to far red and low light in terrestrial cyanobacteria: identification of chlorophyll* f *synthase and implications for evolution of photosystem II.”*
11. Invited speaker, Microbiology and Infectious Disease Asia, Singapore, October 11-12, 2016. Declined for medical reasons.

### 2015

1. Plant Research Laboratory, Michigan State University. March 25, 2015. *“Extensive acclimative remodeling of the photosynthetic apparatus in* cyanobacteria *in far-red light.”*
2. Thermal Biology Institute, Montana State University. April 13, 2015. *“FaRLiP and LoLiP: alternative mechanisms for acclimation to far red and low light in terrestrial cyanobacteria.”*
3. Joule Unlimited, Inc., Bedford, MA, June 8, 2015. *“FaRLiP and LoLiP: alternative mechanisms for acclimation to far red and low light in terrestrial cyanobacteria.”*
4. PARC-2, All-hands Meeting, Washington University in St. Louis, June 22-25, 2014. *“Life beyond the (red) edge… FaRLiP and LoLiP: novel acclimative responses in terrestrial cyanobacteria.”*
5. Invited plenary lecturer and participant, 6th European Conference on Prokaryotic and Fungal Genomics (ProkaGENOMICS 2015), Göttingen, Germany, September 29 to October 2, 2015. Cancelled for medical reasons.
6. Invited speaker and participant, International Conference on Tetrapyrrole Photoreceptors and Photosynthetic Organisms (ICTPPO 2015). Hagoshrim Resort Hotel, Israel. October 11-16, 2015. Cancelled for medical reasons.

### 2014

1. University of Turku, Turku, Finland, May 15, 2014. Cyanobacterial biofuel production/synthetic biology workshop. “Synechococcus *sp. PCC 7002: a robust cyanobacterial platform for biofuels and biotechnological development*.”
2. University of Turku, Turku, Finland, May 16, 2014. *“Unculturable but not undiscoverable: identification & isolation of novel chlorophototrophs from hot spring microbial mats by genomics, metagenomics, and metatranscriptomics.”*
3. University of Copenhagen, Copenhagen Microbiology Center, May 19, 2014. *“Unculturable but not undiscoverable: identification & isolation of novel chlorophototrophs from hot spring microbial mats by genomics, metagenomics, and metatranscriptomics.”*
4. PARC All Hands Meeting. Washington University in St. Louis, June 17-18, 2014. *“Extensive acclimative remodeling of the photosynthetic apparatus in* Leptolyngbya *sp. JSC-1 in far-red light.”*
5. Symposium organizer and invited speaker, 7^th^ International Conference on Porphyrins and Phthalocyanines (ICPP-8), June 22-27, 2014, Istanbul, Turkey. *“Extensive acclimative remodeling of the photosynthetic apparatus in* Leptolyngbya *sp. JSC-1 in far-red light.”*
6. Summer course in Eco-physiology of biofilms. 12 hours of lectures on microbial physiology. SCELSE, Nanyang Technological University, Singapore. July 6 to July 25, 2014.
7. Invited speaker and participant, Gordon Research Conference, Photosynthesis: From evolution of fundamental mechanisms to radical re-engineering. Mount Snow Resort, VT. August 10-15, 2014. *“Light niche adaptation and acclimation mechanisms in hot spring cyanobacteria.”*
8. Washington University in St. Louis, PARC Seminar Series, Sept. 22, 2014. *“Extensive acclimative remodeling of the photosynthetic apparatus in* Leptolyngbya *sp. JSC-1 in far-red light.”*
9. Washington University in St. Louis, Dept. of Biology, Sept. 23, 2014. *“Unculturable but not undiscoverable: identification & isolation of novel chlorophototrophs from hot spring microbial mats by genomics, metagenomics, and metatranscriptomics.”*
10. Dept. of Biochemistry and Molecular Biology, Faculty Lunchtime Seminar series. October 14, 2014. *“Extensive acclimative remodeling of the photosynthetic apparatus in* Leptolyngbya *sp. JSC-1 in far-red light.”*
11. State College Bird Club, December 10, 2014. *“Raptors of the Argentina and Spain… A Raptours Report.”*

### 2013

1. Invited Discussion Leader, Gordon Research Conference, “Carotenoids,” Ventura, CA. January 6-11, 2013. Declined (scheduling conflict).
2. Center for Bioenergy and Photosynthesis, Arizona State University, March 7, 2013. , “Synechococcus *sp. PCC 7002: a robust cyanobacterial platform for biofuels and biotechnological development*.”
3. School of Life Sciences, Genomics seminar series, Arizona State University. March 8, 2012. *“Unculturable but not undiscoverable: identification (& enrichment) of novel chlorophototrophs from hot spring microbial mats by genomics, metagenomics, and metatranscriptomics.”*
4. Invited plenary speaker, Light-Harvesting Processes-2013. Kloster Banz, Germany, April 7-11, 2013. “*Biosynthesis and assembly of bacteriochlorophylls into chlorosomes.”*
5. Joule Unlimited, Bedford, MA. April 29, 2013. “Synechococcus *sp. PCC 7002: a robust cyanobacterial platform for biofuels and biotechnological development*.”
6. American Society for Microbiology, National Meeting, Denver, CO. Invited symposium co- convener, speaker, and participant, May 18-21, 2013. *“Modular design of light reactions driving dark reactions that shaped Earth’s evolution.”*
7. Department of Chemistry and Geochemistry, Colorado School of Mines, Golden, CO. May 22, 2013. “Synechococcus *sp. PCC 7002: a robust cyanobacterial platform for biofuels and biotechnological development*.”
8. Department of Chemistry and Geochemistry, Colorado School of Mines, Golden, CO. May 23, 2013. *“Unculturable but not undiscoverable: identification (& enrichment) of novel chlorophototrophs from hot spring microbial mats by genomics, metagenomics, and metatranscriptomics.”*
9. Woods Hole Microbial Diversity Summer Course, July 6, 2013. “*Evolution of Photosynthesis: Sam Granick was right…”*
10. July 12-17, 2013. Singapore Centre on Environmental Life Sciences Engineering, Nanyang Technical University, Singapore. *Seven Lectures on microbial (eco)-physiology.*
11. Energy Innovation Symposium, Penn State University and Albert-Ludwigs-Universität Freiburg. July 31—August 2, 2013, University Park, PA. *“*Synechococcus *sp. PCC 7002: a robust cyanobacterial platform for biofuels and biotechnological development.”*
12. Invited speaker, Light Harvesting Satellite Meeting, 16^th^ International Congress on Photosynthesis, August 8-11, 2013. St. Louis, MO. “The synthesis and assembly of bacteriochlorophylls in chlorosomes.”
13. Biomolecular Materials Program, Department of Energy, PI Meeting, August 19-21, 2013, Gaithersburg, MD. *“A hybrid biological/organic half-cell for generating dihydrogen.”*
14. Invited speaker, International Conference on Tetrapyrrole Photoreceptors and Photosynthetic Organisms (ICTPPO 2013). September 11-15, 2013. The Huazhong Agricultural University, Wuhan, China. Cancelled due to teaching conflict.
15. Invited speaker and participant, Lorentz Workshop, University of Leiden, The Netherlands; October 28-November 1, 2013. Cancelled due to teaching conflict.
16. Invited speaker and participant, 2013 Photosynthetic Systems Research Biannual Meeting, Office of Basic Energy Sciences, Department of Energy. November 3-6, 2013, Annapolis, MD. *Biosynthesis and assembly of bacteriochlorophylls into chlorosomes: bacteriochlorophyll* f*, the “forbidden” bacteriochlorophyll.”*

### 2012

1. Invited speaker, Metabolomics-2012, February 20-22, 2012. Declined.
2. Dept. of Biology, University of California, San Diego, February 29, 2012. *“The phylum* Chlorobi *unexpected diversity among green bacteria revealed by genomics, metagenomics, and metatranscriptomics.”*
3. Dept. of Biological Sciences, University of Southern California, March 2, 2012. *“The phylum* Chlorobi *unexpected diversity among green bacteria revealed by genomics, metagenomics, and metatranscriptomics.”*
4. Dept. of Microbiology, Immunology, and Molecular Genetics, University of California, Los Angeles, March 5, 2012. *“The phylum* Chlorobi *unexpected diversity among green bacteria revealed by genomics, metagenomics, and metatranscriptomics.”*
5. Daniel I. Arnon Lecture; Dept. of Plant and Microbial Biology, University of California, Berkeley. March 7, 2012. *“The phylum* Chlorobi *unexpected diversity among green bacteria revealed by genomics, metagenomics, and metatranscriptomics.”*
6. State College Bird Club, March 28, 2012. “*Raptors of Australia.”*
7. Invited plenary speaker and participant, 3^rd^ Workshop on Microbial Sulfur Metabolism, Noordwijkerhout, The Netherlands, April 15-18, 2012. *“The phylum* Chlorobi *unexpected diversity among green bacteria.”*
8. Dept. of Chemistry, University of Leiden, April 20, 2012. *“The phylum* Chlorobi *unexpected diversity among green bacteria.”*
9. Nanyang Technical University, Singapore Centre on Environmental Life Sciences Engineering, Singapore. May 11, 2012 *“The phylum* Chlorobi: *unexpected diversity among green bacteria revealed by genomics, metagenomics, and metatranscriptomics.”*
10. American Society for Photobiology, Montreal, Quebec, Canada. June 23-27, 2012, (declined, scheduling conflict).
11. Institute for Hydrobiology, Wuhan, China. June 27, 2012. *“The phylum* Chlorobi: *unexpected diversity among green bacteria revealed by genomics, metagenomics, and metatranscriptomics.”*
12. Invited symposium speaker and participant. 7^th^ International Conference on Porphyrins and Phthalocyanines (ICPP-7), Jeju Island, South Korea, July 1-6, 2012. *“Bacteriochlorophyll* f*: properties of chlorosomes containing the “forbidden chlorophyll.”*
13. Invited plenary speaker and participant, 14^th^ International Symposium on Phototrophic Prokaryotes, Porto, Portugal, August 5-10, 2012. *“Genomics, metagenomics and metatranscriptomics: inferred metabolic interactions are the basis of community structure and function in chlorophototrophic hot spring microbial mats.”*
14. Invited Symposium Convener, Speaker, and Participant. *“The heated competition for light: chlorophototroph diversity in the microbial mats of alkaline siliceous hot springs in Yellowstone National Park.”* International Society for Microbial Ecology, Copenhagen, Denmark, August 24- 29, 2012.
15. DOE-Plant Research Laboratory, Michigan State University. Sept. 10, 2012. *“The phylum* Chlorobi: *unexpected diversity among green bacteria revealed by genomics, metagenomics, and metatranscriptomics.”*
16. Dept. of Plant Pathology and Environmental Microbiology, Penn State University. Sept. 24, 2012. *“The phylum* Chlorobi: *unexpected diversity among green bacteria revealed by genomics, metagenomics, and metatranscriptomics.”*
17. Institüt für Biologie, Albert-Ludwigs-Universität Freiburg, October 8, 2012. “*Synechococcus sp. PCC 7002: a robust cyanobacterial platform for biofuels and biotechnological development*.”
18. University of Tokyo, Section of Biological Sciences, November 27, 2012. *“The phylum* Chlorobi: *unexpected diversity among green bacteria revealed by genomics, metagenomics, and metatranscriptomics.”*
19. Invited plenary Speaker, First International symposium on Biofunctional Chemistry, Tokyo Institute of Technology, Tokyo, Japan. November 28-30, 2012. *“Photosynthesis in the (near) dark: biosynthesis of BChl* c *and its assembly into supramolecular nanotubes in chlorosomes.”*
20. 2^nd^ International Symposium on Biosynthesis of Tetrapyrroles (BSTP12), Dept. of Chemistry, Ritsumeikan University, Kusatsu, Shiga, Japan. November 30-December 2, 2012. *Biosynthesis and assembly of bacteriochlorophylls into chlorosomes: bacteriochlorophyll* f*, the “forbidden” bacteriochlorophyll.”*
21. Invited speaker and participant, India NSF Science and Technology Forum, *“Cyanobacteria: Molecular networks to Biofuels,”* Lonavala (Mumbai), India. December 16-20, 2012. “*Synechococcus sp. PCC 7002: a robust cyanobacterial platform for biofuels and biotechnological development*.”

### 2011

1. Invited speaker and participant. Yellowstone Research Coordination Network Workshop, Jackson, WY. January 14-16, 2011. *“Genomics, metagenomics and metatranscriptomics: how genomic methods have expanded our knowledge of chlorophototrophic bacteria.”*
2. University of Victoria, Dept. of Biochemistry and Microbiology, British Columbia, Canada. March 25, 2011. *“Photosynthesis in the (near) dark: biosynthesis, structure, and evolution of the most powerful light-harvesting antenna system, the chlorosomes of green chlorophototrophic bacteria”*
3. University of Victoria, Dept. of Biochemistry and Microbiology, British Columbia, Canada. March 25, 2011. “Synechococcus *sp. PCC 7002: a robust cyanobacterial platform for biofuels and biotechnological development*.”
4. Invited Plenary Speaker, Eastern Regional Photosynthesis Conference, Woods Hole, MA, April 1-3, 2011. *“Genomics, metagenomics and metatranscriptomics: how genomic methods have expanded our knowledge of chlorophototrophic bacteria.”*
5. Dept. of Biochemistry, University of Nebraska-Omaha, April 11, 2011. *“Photosynthesis in the (near) dark: how nature modified the structure of Chl a to produce BChl d and then proceeded to screw up the supramolecular nanotubes in chlorosomes to make even more efficient light- harvesting antennae.”*
6. Program in Integrative Microbiology and Biochemistry, University of Montana, Missoula. April 25, 2011. *“Photosynthesis in the (near) dark: biosynthesis, structure, and evolution of the most powerful light-harvesting antenna system, the chlorosomes of green chlorophototrophic bacteria”*
7. Lawrence Berkeley Laboratories, Berkeley, CA. May 31, 2011. *“Photosynthesis in the (near) dark: biosynthesis, structure, and evolution of the most powerful light-harvesting antenna system, the chlorosomes of green chlorophototrophic bacteria”*
8. Air Force Office of Scientific Research, Bioenergy Review. Arlington, VA. June 6-10. “Synechococcus *sp. PCC 7002: a robust cyanobacterial platform for biofuels and biotechnological development*.”
9. Gordon Research Conference, Session Chairman, *Biochemical Aspects of Photosynthesis*, Davidson College, Davidson, NC. June 12-17, 2011. *“Pigment biosynthesis and turnover.”*
10. Invited speaker and participant; Workshop on *“Complexity and Systems Biology Microbial Biofuels,”* Warwick Systems Biology Centre, Warwick, UK. June 20-24, 2011. “*Synechococcus sp. PCC 7002: a robust cyanobacterial platform for biofuels and biotechnological development*.”
11. Invited speaker and participant, “The Evolution of Photosynthesis and Oxygenation of the Earth.” Australian Academy of Science and Australian Center for Astrobiology, June 28-29, 2011. *“Genomics, metagenomics and metatranscriptomics: how genomic methods have expanded our knowledge of chlorophototrophic bacteria.”*
12. International Conference on Tetrapyrrole Photoreceptors and Photosynthetic Organisms (ICTPPO). Invited speaker and participant. Berlin, Germany, July 24-28, 2011. “*Biosynthesis,*

*structure, and evolution of the most powerful light-harvesting antenna system, the chlorosomes of green chlorophototrophic bacteria.”*

1. Invited Speaker, “*Photosynthesis Research for Sustainability*.” Baku, Azerbaijan, July 24-30, 2011. (declined; scheduling conflict).
2. *“Biological Systems Interactions: Overview of chlorophototroph models and communities.”*

Pacific Northwest National Laboratory, FSFA Review, September 8-9, 2011.

1. Participant (+Poster), PI meeting, Energy Biosciences, Basic Energy Sciences, DOE. November 6-9, 2011.
2. Joule Biotechnologies, Unlimited, Cambridge, MA, November 21, 2011. *“The phylum* Chlorobi*: unanticipated complexity among green bacteria.”*
3. Dept. of Biochemistry and Molecular Biology, The Pennsylvania State University, November 28, 2011. *“The phylum* Chlorobi*: unanticipated complexity among green bacteria.”*

### 2010

1. Invited speaker and participant, Gordon Research Conference, “Carotenoids.” Ventura, CA January 17-22, 2010. *“Carotenoid biosynthesis in chlorophototrophic bacteria: using genomics to identify new genes involved in carotenogenesis.”*
2. Dept. of Biophysics, Johns Hopkins University, Baltimore, MD February 1, 2010. *“Photosynthesis in the (near) dark: biosynthesis of BChl* c *and its assembly into supramolecular nanotubes in chlorosomes.”*
3. Biofuels interest group, Johns Hopkins University, Baltimore, MD February 2, 2010. “Synechococcus *sp. PCC 7002: a robust cyanobacterial platform for biofuels development*.”

4 Seminar, Plant Physiology program, Penn State, February 8, 2010. “*Photosynthesis in the (near) dark: biosynthesis of BChl* c *and its assembly into supramolecular nanotubes in chlorosomes”*

1. Seminar, University of California, Davis, March 23, 2010. “*Photosynthesis in the (near) dark: biosynthesis of BChl* c *and its assembly into supramolecular nanotubes in chlorosomes”*
2. Seminar, Massachusetts Institute of Technology, May 13, 2010. *“Carotenoid biosynthesis in chlorophototrophic bacteria: using genomics to identify new genes involved in carotenogenesis.”*
3. Pacific Northwest National Laboratory, FSFA/BSFA groups. “Synechococcus *sp. PCC 7002: a robust cyanobacterial platform for biofuels development*.” June 8, 2010
4. 10^th^ Cyanobacterial Molecular Biology Workshop, Lake Arrowhead, CA, June 11-15, 2010. “Synechococcus *sp. PCC 7002: a robust cyanobacterial platform for biofuels development*.”
5. Invited Speaker and participant, Quantum Effects in Biological Sciences, Harvard University, June 17-20, 2010. *“Photosynthesis in the (near) dark: Light harvesting in green chlorophototrophic bacteria.”*
6. Invited symposium keynote speaker and participant. 6^th^ International Conference on Porphyrins and Phthalocyanines (ICPP-6), July 4-9, 2010. Santa Ana Pueblo, New Mexico. *“Biosynthesis and structural organization of bacteriochlorophyll c in green bacteria.”*
7. Sandia National Laboratory, Center for Integrated Nano Technologies (CINT), July 12, 2010.

*“Biosynthesis and structural organization of bacteriochlorophyll c in green bacteria.”*

1. AFOSR Annual Bioenergy Review, Arlington, VA, July 26-30, 2010. “Synechococcus *sp. PCC 7002: a robust cyanobacterial platform for biofuels development*.”
2. AFOSR Annual Bioenergy Review, Arlington, VA, July 26-30, 2010. *“A light-driven, hybrid electrochemical half-cell for solar biofuels production: CO2 reduction to formic acid.”*
3. Invited session convener and speaker, ISME13 (13^th^ International Symposium on Microbial Ecology), Seattle, WA, August 22-27, 2010. *“Genomics, metagenomics and metatranscriptomics: how genomic methods have expanded our knowledge of chlorophototrophic bacteria.”*
4. University of Sydney, Biological Sciences, Sydney, Australia. Sept. 2, 2010. “Synechococcus *sp. PCC 7002: a robust cyanobacterial platform for biofuels development*.”
5. University of New South Wales, Sydney, Australia. Sept. 25, 2010. *“Genomics, metagenomics and metatranscriptomics: how genomic methods have expanded our knowledge of green chlorophototrophic bacteria.”*
6. Utah State University, Dept. of Chemistry and Biochemistry, Logan, Utah. October 4, 2010. *“Photosynthesis in the (near) dark: biosynthesis, structure, and evolution of the most powerful light-harvesting antenna system, the chlorosomes of green chlorophototrophic bacteria”*
7. Utah State University, Dept. of Chemistry and Biochemistry, Logan, Utah. October 5, 2010.

*“*Synechococcus *sp. PCC 7002: a robust cyanobacterial platform for biofuels development.”*

1. Anaerobic Phototrophic Ecosystems, Ancient and Modern. October 11-13, 2010. Fayetteville, NY. “*The green bacteria: anoxygenic chlorophototrophs.*
2. State University of New York College of Environmental Science and Forestry, Syracuse, NY, October 15, 2010. *“*Synechococcus *sp. PCC 7002: a robust cyanobacterial platform for biofuels development.”*
3. University of Louisville, Dept. of Biology, Brown and Williamson Distinguished Lecturer Series. October 21, 2010. *“*Synechococcus *sp. PCC 7002: a robust cyanobacterial platform for biofuels development.”*
4. University of Louisville, Dept. of Biology, Brown and Williamson Distinguished Lecturer Series. October 22, 2010. *“Photosynthesis in the (near) dark: biosynthesis, structure, and evolution of the most powerful light-harvesting antenna system, the chlorosomes of green chlorophototrophic bacteria”*
5. Dept. of Microbiology, Montana State Univ., Bozeman, MT, Nov. 5, 2010. *“*Synechococcus *sp. PCC 7002: a robust cyanobacterial platform for biofuels development.”*
6. Montana State University, Thermal Biology Institute, Bozeman, MT, Nov. 15, 2010. *““Phishing for phototrophs in Yellowstone: characterization of novel phototrophs and inferences about metabolic patterns from global metatranscriptome analyses by nextGen sequencing of cDNAs.”*
7. Pacific Northwest National Laboratory, Environmental Microbiology Science Laboratory. Dec. 15, 2010. *“Genomics, metagenomics and metatranscriptomics: how genomic methods have expanded our knowledge of chlorophototrophic bacteria.”*

### 2009

1. AFOSR-NREL meeting on carbon-based biofuels; January 8-9, 2009, Golden, CO; invited speaker and participant. Had to cancel due to illness and weather.
2. Utah State University, Center for Integrated Biosystems, January 15, 2009. Had to cancel due to illness and weather.
3. Montana State University, Thermal Biology Institute, March 2, 2009. “Phishing for phototrophs in Yellowstone: metagenomics and the discovery of *Candidatus* Chloracidobacterium thermophilum.”
4. International Symposium on Microbial Sulfur Metabolism, Invited Plenary Speaker and participant. Tomar, Portugal. “–omics” analyses of chlorophototrophic sulfur bacteria.” March 15-18, 2009.
5. University of Leiden, The Netherlands. “Phishing for phototrophs in Yellowstone: metagenomics and the discovery of *Candidatus* Chloracidobacterium thermophilum.” March 20, 2009.
6. Dept. of Molecular Biology, Cell Biology and Biochemistry, Brown University. Symposium to honor the retirement of Samuel I. Beale, invited speaker, March 27, 2009. Title: “Tilting at tetrapyrroles…”
7. State College Bird Club, April 29, 2009. “(Mostly) Raptors of the Western U. S.”
8. American Society for Microbiology, Invited Symposium speaker: Shaking the Tree of Life: “Photosynthesis (and Evolution…).” Philadelphia, PA, May 17-21, 2009.
9. American Society for Microbiology, Invited Symposium speaker: Division K, Philadelphia, PA, May 17-21, 2009 (declined; talk was given by my Ph. D. student Amaya M. Garcia Costas)
10. Harvard University, Microbial Sciences Initiative Symposium, June 18, 2009. “Phishing for phototrophs in Yellowstone: metagenomics and metatranscriptomics of hot spring chlorophototrophs.”
11. World Congress on Industrial Biotechnology and Bioprocessing. Invited symposium speaker. Montreal, Quebec, Canada, July 19-22. “*Synechococcus* sp. PCC 7002: a robust and versatile cyanobacterial platform for biofuels development.”
12. International Conference on Tetrapyrrole Photoreceptors and Photosynthetic Organisms, invited plenary speaker. Asilomar, CA, July 26-30, 2009. Title: “*Synechococcus* sp. PCC 7002: a robust and versatile cyanobacterial platform for biofuels development.”
13. FIBR Workshop, Montana State University Bozeman, MT, August 4, 2009. “Phishing for phototrophs in Yellowstone: Discovery of *Candidatus* Chloracidobacterium thermophilum.”
14. FIBR Workshop, Montana State University Bozeman, MT, August 5, 2009. “Metatranscriptomic analyses of the phototrophic mat community of an alkaline siliceous hot spring.”
15. International Symposium on Phototrophic Prokaryotes, Montreal, Canada, August 9-14, 2009. Invited speaker: “Bacteriochlorophylls in chlorosomes of *Chlorobaculum tepidum* form coxial nanotubes from helical *syn-anti* monomer stacks.”
16. AFOSR Annual Meeting, Arlington, VA. BioSolar Hydrogen MURI. August 18-21, 2009. “Transcriptional profiling and metabolic engineering of a robust cyanobacterium: *Synechococcus* sp. PCC 7002, a model organism for biofuels production.”
17. DOE-Basic Energy Sciences, Photosynthetic Systems Research Annual Review Conference, October 25-28, 2009, Annapolis, MD. “Light energy transduction in green bacteria”

### 2008

1. Research Coordination Network Symposium, Mammoth Hot Springs, Yellowstone National Park, January 10-13, 2008. Invited speaker and participant.
2. Dept. of Chemistry and Biochemistry, Montana State University, January 18, 2008. “From apo- to holo- (and back…): iron-sulfur cluster assembly and other post-translational modifications of the cyanobacterial photosynthetic apparatus.”
3. Department of Biochemistry and Plant Biology, Michigan State University, February 4, 2008.
4. Department of Microbiology, Michigan State University, February 5, 2008.
5. ASM Microbiology Club, Penn State University, April 1, 2008.
6. Massachusetts Institute of Technology, May 15, 2008, Dept. of Civil and Environmental Engineering.
7. Department of Energy EERE Hydrogen Review Meeting, Arlington, VA, June 11, 2008.
8. Gordon Research Conference, Biochemical aspects of photosynthesis, June 22-27, 2008, Mt. Holyoke, MA.
9. Woods Hole, Microbial Diversity Course, July 18, 2008. “Phishing for phototrophs in Yellowstone: how metagenomics revealed a 6^th^ bacterial phylum with chlorophotrophic members.”
10. Gordon Research Conference, Chemistry and biology of tetrapyrroles, July 20-24, 2008. Newport, R. I. Invited Speaker: The biosynthesis of bacteriochlorophyll *c* and its assembly into supramolecular aggregates in chlorosomes.
11. Pacific Northwest National Laboratory, August 11, 2008.
12. University of Copenhagen, Dept. of Biology, Copenhagen, Denmark, October 20, 2008.
13. Nordic Photosynthesis Conference, invited plenary speaker and participant, Copenhagen, Denmark, October 21-24, 2008.

### 2007

1. Plant and Animal Genome Meeting XV, NSF-USDA Microbial Genomes Symposium, January 13-17, 2007; San Diego, CA. Invited speaker and participant.
2. Department of Microbiology, University of British Columbia. January 23, 2007
3. Department of Biology, University of Oregon, January 29, 2007.
4. Department of Biology, University of Oregon, January 30, 2007
5. Center for Comparative Genomics and Bioinformatics, Penn State University, Feb. 21, 2007.
6. PS2007 Drymen, Light-harvesting Satellite Meeting, Drymen, Scotland, July 19-22, 2007.
7. PS2007, 14^th^ International Congress on Photosynthesis, Glasgow, Scotland, July 22-27, 2007.
8. Massachusetts Institute of Technology, August 20, 2007, Dept. of Civil and Environmental Engineering
9. Flagship Ventures, Boston, MA, August 21, 2007.
10. CrossOver 2007, Bioenergy: From Fields to Wheels, The Pennsylvania State University, September 4, 2007.
11. AFOSR MURI Review, Princeton, NJ, October 31, 2007.
12. Midwest Photosynthesis Meeting, Keynote speaker, Turkey Run, IN. Nov. 9-11, 2007.

### 2006

1. Plant and Animal Genome Meeting XIV, January 14-18, 2006. San Diego, California. Invited speaker and participant. “Adventures in genomics: the clarifying light of comparative genomics illuminates those denizens of the deep, the green bacteria.
2. Department of Microbiology, Montana State University, Feb. 17, 2006.
3. Yellowstone Research Coordination Network, Invited Speaker and Participant. February 17-19, 2006. Sponsored by the Thermal Biology Institute, Montana State University.
4. Joint Genome Institute-Department of Energy, First Annual User Meeting, March 30 to April 1, 2006. Invited speaker and attendee. Walnut Creek, CA.
5. Frontiers in Metallobiochemistry, 25^th^ Summer Symposium in Molecular Biology, The Pennsylvania State University, June 7-10, 2006. Speaker and participant.
6. International Symposium on Microbial Sulfur Metabolism, June 29 to July 2, 2006. Münster, Germany. Invited speaker and participant (declined).
7. Princeton University, 2^nd^ Bio-Solar Hydrogen Symposium, July 29-30, 2006. Speaker and participant.
8. XII^th^ International Symposium on Phototrophic Prokaryotes, invited keynote closing speaker. August 27 to September 1, 2006. Pau-Palais Beaumont, France.
9. Institute of Marine and Coastal Sciences, Cook College, Rutgers University, New Brunswick, NJ. Adventures in Genomics: Bioprospecting for new phototrophs in Yellowstone National Park.
10. Bartramian Audubon Society, Slippery Rock, PA, October 9, 2006. Raptors of Western North America and Central America.
11. Juniata Valley Audubon Society, October 17, 2006; Raptors of Western North America and Central America
12. Microbiologists at Penn State, November 1, 2006. Bioprospecting for phototrophs in Yellowstone National Park.

### 2005

1. School of Oceanography, University of Washington, Seattle. February 14, 2005. “Photosynthesis in the (near) dark: Genomics-enabled biochemical genetics in the green sulfur bacterium *Chlorobium tepidum.”*
2. Dept. of Biology, Indiana University, Bloomington, IN. April 8, 2005. “Photosynthesis in the (near) dark: Genomics-enabled biochemical genetics in the green sulfur bacterium *Chlorobium tepidum.”*
3. Dept. of Biological Sciences, Purdue University, West Lafayette, IN. April 11, 2005. “Photosynthesis in the (near) dark: Genomics-enabled biochemical genetics in the green sulfur bacterium *Chlorobium tepidum.”*
4. College of Life Sciences, Beijing University, Beijing, China. “Photosynthesis in the (near) dark: Genomics-enabled biochemical genetics in the green sulfur bacterium *Chlorobium tepidum.”*
5. Invited Participant and speaker, 3rd Japanese/German Binational Symposium, Chiba, Japan, June 5-10, 2005. Functional Genomics of Cyanobacteria- New Approaches for Investigation of Diversity. Title: “The complete genome sequence of the marine unicellular cyanobacterium *Synechococcus* sp. PCC 7002. Characterization of a small multigene family of phycobiliprotein lyases.
6. Kanazawa Institute of Technology, Kanazawa, Japan. June 13, 2005 “Photosynthesis in the (near) dark: Genomics-enabled biochemical genetics in the green sulfur bacterium *Chlorobium tepidum.”*
7. Kanazawa University, Kanazawa, Japan. June 13, 2005. “Photosynthesis in the (near) dark: Genomics-enabled biochemical genetics in the green sulfur bacterium *Chlorobium tepidum.”*
8. Princeton University, Department of Chemistry and U. S. Airforce Office of Scientific Research. June 23-26, 2005. Participant and invited speaker. “Complete genome sequence of the euryhaline unicellular cyanobacterium *Synechococcus* sp. PCC 7002: Genetic manipulations and prospects for biohydrogen production.”
9. Gordon Research Conference, Biochemical Aspects of Photosynthesis, “The clarifying light of comparative genomics illuminates those denizens of the deep, the green sulfur bacteria.” July 3- 8, 2005. Bryant University, Rhode Island.
10. Montana State University, Thermal Biology Institute, August 17, 2005. “Genome Annotation: what, how, and why.”
11. Montana State University, Thermal Biology Institute, August 23, 2005. “Adventures in Genomics: The clarifying light of comparative genomics illuminates those denizens of the deep, the green sulfur bacteria.”
12. University of Colorado-Boulder, Dept. of Molecular, Cellular, and Developmental Biology, August 29, 2005. “Adventures in Genomics: The clarifying light of comparative genomics illuminates those denizens of the deep, the green sulfur bacteria.”
13. University of Missouri-Columbia, Department of Biochemistry. September 1, 2005. “Adventures in Genomics: The clarifying light of comparative genomics illuminates those denizens of the deep, the green sulfur bacteria.”
14. Juniata College, Biological Sciences. October 25, 2005. “Adventures in Genomics: The clarifying light of comparative genomics illuminates those denizens of the deep, the green sulfur bacteria.”
15. State College Bird Club, October 26, 2005. Raptors of North and Central America.

### 2004

1. Department of Microbiology, University of Illinois. February 10, 2004. “Genomics-enabled biochemical genetics in the green sulfur bacterium *Chlorobium tepidum*.”
2. State College Bird Club, “Raptors of Kenya.” February 25, 2004.
3. The Institute for Genomic Research, May 4, 2004. “Genomics-enabled biochemical genetics in the green sulfur bacterium *Chlorobium tepidum*.”
4. The Institute for Genomic Research, May 6, 2004. “Raptors of Kenya.”
5. Woods Hole Oceanographic Institute, Woods Hole, MA. 3 lectures on phototrophs and photosynthesis for the Microbial Diversity Summer Course. 1. Phototrophs and Photosynthesis;

2. Cyanobacterial quinomics; 3. Photosynthesis in the (near) dark: Genomics-enabled biochemical genetics in the green sulfur bacterium *Chlorobium tepidum.*

1. Max-Planck-Institute for Developmental Biology, Tübingen, Germany. “Photosynthesis in the (near) dark: Genomics-enabled biochemical genetics in the green sulfur bacterium *Chlorobium tepidum”*
2. SFB533 “Light-induced Dynamics in Chromoproteins,” Freising, Germany. July 9-11, 2004. Invited speaker and participant. “A novel family of phycobiliprotein lyases.”
3. Light-harvesting Antennae Satellite Meeting, International Congress on Photosynthesis, Montreal, Canada. August 26-29, 2004. “A novel family of phycobiliprotein lyases.”
4. International Congress on Photosynthesis, Montreal, Canada, August 29 to September 3, 2004. Invited Speaker. Bacteriochlorophyll and carotenoid biosynthesis in *Chlorobium tepidum*.
5. Juniata Valley Audubon Society, Altoona PA, Sept. 21, 2004. “Raptors of Kenya.”
6. Lycoming Audubon Society, Williamsport, PA, Sept. 22, 2004. “Raptors of Kenya.”
7. 12^th^ Small Genomes Conference, Lake Arrowhead, CA. Sept. 26-30, 2004. “Genomics-enabled biochemical genetics in the green sulfur bacterium *Chlorobium tepidum*.”
8. Todd (PA) Bird Club, Indiana, PA. October 5, 2004, “Raptors of Kenya.”
9. Montana State University, Thermal Biology Institute, October 18, 2004. “Photosynthesis in the (near) dark: Genomics-enabled biochemical genetics in the green sulfur bacterium *Chlorobium tepidum.”*
10. West Chester Bird Club, West Chester, PA, November 1, 2004. “Raptors of Kenya.”
11. Bake Oven Knob Fall Festival, November 13, 2004. “Raptors of Kenya.”

### 2003

1. Department of Biology, Georgia Institute of Technology. February 4, 2003.
2. Department of Biology-I, Botanical Institute, Ludwig Maximilian University, Munich, Germany. May 6, 2003.
3. School of Cell Biology and Plant Physiology, Regensburg University, Regensburg, Germany. May 8, 2003.
4. Institut für Botanik III, Heinrich-Heine University, Düsseldorf, Germany. May 12, 2003.
5. Photosystem I Workshop, Cooperative Research Center” (Sfb 498), Freie Universität, Berlin, Germany. May 15-17, 2003.
6. American Society for Microbiology, Colloquium Convener and Speaker, National meeting, Washington, D. C. (May 18-22, 2003)
7. Department of Microbiology and Cell Science, University of Florida, June 9, 2003.
8. Discussion Leader, Gordon Research Conference, Biophysical Aspects of Photosynthesis, June 22-26, 2003.
9. Invited speaker and participant, International Workshop on Green and Heliobacteria, Kazusa DNA Research Institute, Kazusa, Japan. August 22-24, 2003.
10. Invited Plenary Speaker, International Symposium on Phototrophic Prokaryotes, Tokyo, Japan, August 24-29, 2003
11. Department of Bioscience and Biotechnology, Drexel University, Philadelphia, PA, October 9, 2003.
12. Department of Biology, University of Pennsylvania, October 10, 2003.
13. Invited keynote speaker, Allegheny Branch Meeting, American Society for Microbiology, October 24-25, 2003.
14. Department of Biochemistry, Michigan State University. November 13, 2003.
15. Department of Chemistry and Biochemistry, University of Southern Mississippi, November 21, 2003.

### 2002

1. Dept. of Chemistry and Biochemistry, Utah State University, two talks, April 16 and 17, 2002.
2. EMBO Workshop on Green and Heliobacteria, Passau, Germany. April 19-24, 2002. Invited speaker and session chairman.
3. Institut Pasteur, Dept. of Basic and Medical Microbiology, April 25, 2002.
4. Penn State University, Plant Physiology Seminar Series. September 4, 2002.
5. U. S.-Japan Workshop: Microbial and Plant Metabolism—Function through Genomes. Invited speaker and participant, Maui, Hawaii. November 22-26, 2002.

### 2001

1. Western Regional Photosynthesis Meeting, January 4-7, 2001. Plenary Lecture.
2. University of California, Berkeley. Dept. of Plant and Microbial Biology. January 8, 2001.
3. University of California, Davis. Department of Microbiology. January 10, 2001.
4. Plant Physiology Seminar Series, The Pennsylvania State University. February 14, 2001.
5. Dept. of Biological Sciences, University of New Orleans. March 6, 2001.
6. Kazusa DNA Research Institute, Chiba, Japan. Workshop on Genomics of Phototrophic Organisms. March 19-20, 2001 (co-organizer and speaker).
7. Dept. of Chemistry and Biochemistry, Arizona State University. March 23, 2001.
8. "Light-induced dynamics in biopolymers," Freising Germany. Invited speaker. April 2-6, 2001.
9. Ludwig Maximillian’s Universität, Munich Germany, Dept. of Microbiology. April 9, 2001.
10. American Society for Photobiology, invited speaker. July 8, 2001.
11. Washington State University, July 10, 2001
12. Life Sciences Consortium, Slice of Science, July 17, 2001.
13. VIIth Cyanobacterial Molecular Biology Workshop, Asilomar, CA. July 27-30, 2001
14. Light-harvesting Antenna, August 15-18, 2001, Surfers Paradise, Australia
15. 12^th^ International Congress on Photosynthesis, Brisbane, Australia, August 18-23, 2001. Invited symposium speaker.

### 2000

1. U. S.-Japan Binational NSF Meeting, Atami, Japan. January 11-14, 2000.
2. University of California, Los Angeles, (two lectures). January 28, 2000.
3. University of California, Irvine. January 31, 2000.
4. Carnegie Institution of Washington, Stanford University. February 3, 2000.
5. Bowling Green University, Bowling Green, OH. Paskarnis-Buchanan Lecturer, (two lectures). March 15-16, 2000.
6. Japanese Society for Plant Physiology, Nagoya, Japan. March 29, 2000.
7. Dept. of Plant Molecular Biology, College of Life Sciences, Peking University, Beijing, China. April 3, 2000.
8. Institute of Botany, Academia Sinica, Beijing, China April 4, 2000.
9. Mahidol University, Inst. of Molecular Biology & Genetics, Bangkok, Thailand. (two lectures) April 10-11, 2000.
10. International Photobiology Congress, San Francisco. Symposium Chairman and Speaker, July 1- 6, 2000.
11. International Congress on Phototrophic Procaryotes, Plenary Lecture, Barcelona, Spain. August 26-31, 2000.
12. Scandinavian Photosynthesis Conference, Invited Lecturer. October 2000.

### 1999

1. University of California, Berkeley, Department of Plant Biology and Microbiology, February 8, 1999
2. University of New Orleans, Dept. of Biological Sciences, March 22, 1999
3. Gordon Conference, Biochemical Aspects of Photosynthesis, June 13-18, 1999.
4. ESF Workshop on Green Bacteria, Girona, Spain. August 1999.

### 1998

1. Western Regional Photosynthesis Conference, Asilomar CA. January 11, 1998
2. Arizona State University, Department of Botany. January 29, 1998.
3. Washington State University, Departments of Biochemistry and Biophysics, Chemistry, and Plant Biochemistry, Pullman, WA. Two lectures.
4. Eastern Regional Photosynthesis Conference, Woods Hole, MA, March 13-15, 1998.
5. Massachusetts Institute of Technology, Department of Biology.
6. University of Western Ontario, Dept. of Plant Sciences, Dave Laudenbach Memorial Lecture, April 27, 1998
7. International Workshop on Light-harvesting Systems, Tata, Hungary, August 14-16, 1998.
8. XIth International Congress on Photosynthesis, Budapest, Hungary, August 17-22, Discussion Leader.
9. ESF Advanced Summer School in Photosynthesis, "Low temperature physiology and nitrate metabolism in cyanobacteria," Szeged, Hungary, 23 August to 1 September 1998.
10. ESF Advanced Summer School in Photosynthesis, "The directionality of electron transport in Photosystem I," Szeged, Hungary, 23 August to 1 September 1998.

### 1997

1. Massey University, Department of Biological Sciences, Palmerston North, New Zealand. "Structural and functional studies on cyanobacterial Photosystem I." March 24, 1997.
2. University of Sydney, School of Biological Sciences, "Structural and functional studies on cyanobacterial Photosystem I." April 11, 1997.
3. University of Sydney, School of Biological Sciences. "The role of lipid desaturation in adaptation to growth at low temperature in cyanobacteria." April 14, 1997.
4. Australian National University, Dept. of Biochemistry and Molecular Biology. "Structural and functional studies on cyanobacterial Photosystem I." April 28, 1997.
5. International Phycological Congress, Leiden, The Netherlands. "The role of lipid desaturation in adaptation to growth at low temperature in cyanobacteria." August 10-15, 1997. Invited symposium chairman and speaker.
6. European Science Foundation Workshop on Green Bacteria and Heliobacteria, Urbino, Italy. The roles of proteins in chlorosomes of the green sulfur bacterium *Chlorobium tepidum*." Sept. 1-4, 1997.
7. IXth International Symposium on Phototrophic Procaryotes, Vienna, Austria. "Structural and functional analyses of cyanobacterial photosystem I: the directionality of electron transfer." September 7-12, 1997.
8. Plant Physiology lecture series, Penn State University. "The role of lipid desaturation in adaptation to growth at low temperature in cyanobacteria." November 5, 1997.
9. University of California, Los Angeles, Genomics Lecture Series. "Comparative analyses of the plastid genome of *Cyanophora paradoxa* and *Synechocystis* sp. PCC 6803." November 14, 1997.

### 1996

1. German Botanical Congress, Düsseldorf, August 25-31, 1996. Plenary lecture on cyanobacterial photosynthetic apparatus.
2. Discussion leader, Gordon Conference (Biochemical Aspects of Photosynthesis). August 5-9, 1996.
3. The Australian National University, Kialoa Retreat. "Origins of algal and higher plant chloroplasts." November 28, 1996.

### 1995

1. U. S.-Japan, Binational Information Exchange Seminar on Photosynthesis, Tokyo, Japan (January 17-20, 1995).
2. University of Pennsylvania, Dept. of Biochemistry, March 3, 1995. "Structural and functional studies on cyanobacterial Photosystem I."
3. University of Illinois, Dept. of Plant Biology, March 9, 1995. "Structural and functional studies on cyanobacterial Photosystem I."
4. Microbiology at Penn State, "Shedding new light on photosynthesis," April 5, 1995
5. Biomolecular Structure/Function Group, PSU. "Shedding new light on photosynthesis," May 18, 1995
6. American Society for Microbiology, May 21-25, 1995. Symposium speaker (The cyanelle genome of *Cyanophora paradoxa*: clues to chloroplast origins.).
7. Calvin Laboratory, University of California, Berkeley. June 14, 1995. "Structural and functional studies on cyanobacterial Photosystem I."
8. Intl. Workshop on Light-Harvesting Systems, Dourbie, France, August 16-20, 1995 (The role of proteins in the chlorosomes of green sulfur bacteria).
9. Xth International Congress on Photosynthesis, Montpellier, France. August 20-25, 1995. The role of proteins in the chlorosomes of *Chlorobium* sp. and the production of interposon mutants in genes encoding chlorosome proteins.
10. Dept. of Biology, Temple University, November 27, 1995. (Shedding new light on photosynthesis: structural and functional studies on cyanobacterial Photosystem I)

### 1994

1. Department of Biochemistry, Ohio State University. March 2, 1994.
2. VIIIth International Symposium on Phototrophic Procaryotes, plenary session speaker (Photosynthetic apparatus: biogenesis and regulation) Urbino, Italy. Sept. 10, 1994.

### 1993

1. Department of Biochemistry and Plant Research Laboratory, Michigan State University, March 1993. "Structural and functional studies on cyanobacterial Photosystem I."
2. Dept. of Molecular and Cell Biology, University of California, Berkeley. May 28, 1993. "Structural and functional studies on cyanobacterial Photosystem I."
3. Cyanobacterial Workshop on Molecular Genetics, Asilomar, CA, June 1993. Session chairman and speaker. "Alternative paths of electron transport in cyanobacteria."
4. Invited Symposium speaker, American Society for Photobiology, Chicago, IL. June 1993. "Molecular Biology of Photosystem I."
5. Gordon Research Conferences, “Biochemical Aspects of Photosynthesis.” New Hampton, NH, August 1993. Invited speaker. "Alternative pathways of electron transport in cyanobacteria."
6. EMBO Workshop on Green Bacteria and Heliobacteria. Nyborg, Denmark, August 1993. Invited speaker and participant. "Protein composition of chlorosomes from *Chlorobium tepidum*. Molecular cloning and characterization of the genes encoding four chlorosome proteins."
7. Dept. of Plant Biology, Royal Agricultural University, Copenhagen, Denmark. August 1993. "Structural and functional studies on cyanobacterial PS I."
8. XVth International Botanical Congress, Tokyo, Japan, September 1993. Invited symposium speaker (declined), "Molecular biology of Photosystem I."
9. 6th International Conference on Applied Algology, "Progress in Biotechnology of Photoautotrophic Microorganisms, Trebon, Czech Republic Sept. 6-11, 1993. Invited speaker and participant. "Resolution and reconstitution of the Photosystem I complex using proteins overproduced in *E. coli*."
10. Max-Volmer-Institute for Biophysical and Physical Chemistry, Technical University, Berlin, Germany; "Structural and functional studies on cyanobacterial PS I." Sept. 13, 1993.
11. Department of Botany, University of Georgia. "Structural and functional studies on cyanobacterial Photosystem I." Sept. 30, 1993.
12. Department of Biological Sciences, SUNY Binghamton, November 19, 1993. "Structural and functional studies on cyanobacterial PS I."

### 1992

1. Dept. of Botany and Center for Early Events in Photosynthesis, Arizona State University, Tempe, AZ. April 6, 1992.
2. Depts. of Microbiology and Biochemistry, University of British Columbia. May 14-15, 1992.
3. Dept. of Microbiology, Ohio State University, Columbus, OH. June 3-5, 1992.
4. Invited Symposium speaker, IXth International Congress on Photosynthesis, Nagoya, Japan (August 30-Sept. 4, 1992).
5. Molecular Structure and Regulation of Photosynthetic Pigment Systems, Satellite meeting (Aug. 27-30, 1992) of the IXth International Congress, Sanda, Japan.

### 1991

1. University of Massachusetts, Dept. of Biochemistry
2. 37th Harden Conference of The Biochemical Society, Wye College, Ashford, Kent, United Kingdom. Invited Lecturer and Participant.
3. Third Congress of the International Society for Plant Molecular Biology: Invited Symposium Lecturer and Participant.
4. Keynote Speaker, Midwest Plant Physiology Meeting (April 1991)
5. Brown University, Molecular Biology, Cell Biology and Biochemistry Programs
6. Invited Symposium Lecturer, VIIth International Symposium on Photosynthetic Prokaryotes, Amherst, MA (July 1991)
7. Keynote Speaker, 75th Anniversary Celebration for the Institute for Marine Biosciences, National Research Council of Canada, Halifax, Nova Scotia, August 1991.
8. University College of Wales, Department of Biochemistry, Aberystwyth, Dyfed SY23 3DD, Wales, United Kingdom
9. Department of Biological Sciences, University of Warwick, Coventry, UK
10. ISPMB Satellite Conference, “The Molecular Biology of Photosynthesis,” Tempe AZ (October 1991).

### 1990

1. Lecture Series “Photosynthesis” (7 lectures) Eidgenössische Technische Hochschule, Zürich, Switzerland.
2. Agriculture and Food Research Council Meeting on Photosynthesis, Imperial College of Science and Technology, London, England.
3. Ecole Normale Superior, Laboratoire des Biomembranes, Paris, France.
4. University of Vienna, Institute of Biochemistry, Vienna, Austria.
5. Max-Planck Institute for Biochemistry, Martinsried, West Germany
6. University of Munich, Botanical Institute, Munich, West Germany.
7. University of Geneva, Dept. of Biological Sciences, Geneva, Switzerland.
8. Discussion leader, Gordon Research Conference, "Biochemical Aspects of Photosynthesis."
9. Research Forum, Dept. of Molecular and Cell Biology, The Pennsylvania State University.
10. American Society for Microbiology, Allegheny Branch Meeting, State College, PA.
11. Department of Biochemistry, University of Nebraska-Lincoln

### 1989

1. Department of Chemistry, Portland State University.
2. University of California, Berkeley (Invited Lecturer, Annual Photosynthesis Retreat).
3. VIIIth International Symposium on Photosynthesis, Invited Lecturer
4. Department of Biology, University of Western Ontario.
5. Workshop on Light-Harvesting Systems in Photosynthetic Bacteria, Freiburg, West Germany.
6. University of Freiburg, Department of Microbiology, Freiburg, West Germany.
7. Lecture series (3 lectures) Glasgow University, Department of Botany, Glasgow, Scotland

### 1988

1. Plant Physiology Program Seminar, Penn State University.
2. University of Munich and Deutsche Forschung Gemeinschaft, Botanical Institute, Meeting on Plant Tetrapyrroles.
3. Institut für Molekularbiologie und Biophysik, Eidgenössische Technische Hochschule, Zürich, Switzerland.
4. Penn State Symposium in Plant Physiology: Light-Energy Transduction in Photosynthesis: Higher Plant and Bacterial Models.
5. VIth International Symposium on Phototrophic Prokaryotes, Amsterdam, The Netherlands (Plenary Speaker, Genetics of Cyanobacteria).
6. Gordon Research Conference: "Biochemistry and Genetic Engineering of Microalgal Products (Invited Lecturer).
7. Purdue University, Department of Biological Sciences.
8. Research forum, Dept. of Molecular and Cell Biology, The Pennsylvania State University.

### 1987

1. University of California, Los Angeles, Dept. of Biology, (McKnight Foundation Lecturer)
2. Cornell University, Department of Plant Biology.
3. East Coast Regional Photosynthesis Conference, Woods Hole, MA (Keynote Speaker).
4. Canadian Society for Plant Physiology and Canadian Society for Plant

Molecular Biology (Symposium Lecturer); Queen's University, Kingston, Ontario, Canada.

1. American Society for Plant Physiology, Symposium Speaker, St. Louis, MO.
2. Invited lecturer, Gordon Research Conference, "Biochemical Aspects of Photosynthesis."
3. Second Workshop on Cyanobacterial Molecular Genetics, St. Louis, MO.
4. Invited Lecturer at Workshop on "Photosynthetic Antennae," Munich (Freising), Germany.
5. University of Paris, Institute of Physico-Chemical Biology, Paris, France
6. University of Kentucky, Dept. of Agronomy and Plant Physiology Program.
7. Indiana University of Pennsylvania, Department of Biology.
8. University of Toronto, Department of Botany, Toronto, Ontario, Canada.

### 1986

1. Banbury Conference on Procaryotic Photosynthetic Apparatus, Cold Spring Harbor Laboratory, invited lecturer and participant.
2. Symposium Organizer and Lecturer: The Cyanobacterial Photosynthetic Apparatus: A Molecular Genetics Analysis. National Meeting, American Society of Microbiology, Washington, D. C.
3. Invited lecturer, Marine Biology Course, Hopkins Marine Station.
4. Invited symposium lecturer, VIIth International Congress on Photosynthesis, Providence, R. I.
5. University of Warwick, Dept. of Biological Sciences, Coventry, England.
6. Institut Pasteur, Departement de Biochimie and Genetique Moleculaire, Paris, France.
7. Centre d'Etudes Nucleaires de Saclay, Departement de Biologie, Gif-sur-Yvette, France.

### 1985

1. Invited lecturer, Symposium on "Phycobiliproteins: Biology and Future,"Seattle, WA.
2. Eastern Regional Photosynthesis Conference, Woods Hole, MA.
3. E. I. DuPont de Nemours & Co, Experiment Station/Central Research and Development, Wilmington, DE.
4. Affiliates Program in Recombinant DNA Technology, Winter Workshop, The Pennsylvania State University.
5. Invited lecturer and participant, NATO Advanced Study Institute on "Physiological Ecology of Picoplankton," San Miniato, Italy.
6. Invited lecturer, Vth International Symposium on Photosynthetic Procaryotes, Grindelwald, Switzerland.
7. Invited lecturer, Eastern Regional Meeting, American Society for Microbiology, Huntingdon, WV.
8. Arizona State University, Department of Chemistry.
9. Texas A & M University, Dept. of Biochemistry and Biophysics.
10. Genetics 590, Interdepartmental Program in Genetics, The Pennsylvania State University.
11. Research Forum, Dept. of Molecular and Cell Biology, The Pennsylvania State University.
12. Alcoa Technical Services Seminar, "Genes and Genetic Engineering."

### 1984

1. University of California, Los Angeles, Depts. of Bacteriology and Botany.
2. University of California, Davis, Dept. of Bacteriology.
3. University of California, Berkeley, McKnight Foundation Lecturer.
4. University of Chicago: First Workshop on "Gene Transfer Mechanisms in Cyanobacteria."
5. Plant Physiology 590, The Pennsylvania State University.

### 1981

1. Michigan State University, DOE-Plant Research Laboratory.
2. Oklahoma State University, Dept. Biochemistry.
3. Cornell University, Dept. of Microbiology.
4. University of Wisconsin, Dept. of Bacteriology.
5. The Pennsylvania State University, Dept. of Microbiology, Biochemistry, Molecular and Cell Biology.

### 1980

1. University of Western Ontario, Dept. of Biology.
2. University of Georgia, Dept. of Microbiology.
3. University of Chicago, Dept. of Biology.
4. Arizona State University, Dept. of Botany and Microbiology.
5. University of California, Los Angeles, Dept. of Bacteriology.
6. University of Tennessee, Dept. of Microbiology.
7. Smithsonian Institution, Radiation Biology Laboratory.

### 1979

1. III^rd^ International Symposium on Photosynthetic Procaryotes, invited speaker and participant, Oxford, England.

# Referee Activities

## Manuscripts

1981 *Journal of Bacteriology, Science*.

1982 *Biochimica Biophysica Acta, Plant Physiology, Proceedings of National Academy of Science USA.*

1983 *Archives of Microbiology; Biochimica Biophysica Acta; Journal of Biological Chemistry; Plant Physiology*.

1984 *Biochimica Biophysica Acta*.

1985 *Journal of Applied and Environmental Microbiology, Journal of General Microbiology, Photosynthesis Research, Plant Science Letters*.

1986 *Biochimica Biophysica Acta Journal of Bacteriology, Gene, Journal of Biological Chemistry, Photosynthesis Research*.

1987 *Biochimica Biophysica Acta, Journal of Bacteriology, Photosynthesis Research*.

1988 *Archives of Microbiology; Biochimica Biophysica Acta; Journal of Bacteriology; Journal of Biological Chemistry; Molecular and General Genetics; Photosynthesis Research; Science.*

1989 *Archives of Microbiology, Biochemistry, Biochimica Biophysica Acta, Journal of Bacteriology, Journal of Biological Chemistry, Journal of General Microbiology, Photosynthesis Research, Plant Physiology, Phycologia.*

1990 *Archives of Microbiology; Biochemistry; Biochimica Biophysica Acta; Journal of Bacteriology; Journal of Biological Chemistry; Molecular and General Genetics; Photosynthesis Research; Plant Physiology.*

1991 *Archives of Microbiology; Biochemistry; Biochimica Biophysica Acta; Current Microbiology; Journal of Bacteriology; Journal of Biological Chemistry; Photosynthesis Research; Photochemistry and Photobiology; Physiologia Plantarum; Plant Molecular Biology; Plant Physiology.*

1992 *Archives of Microbiology; Biochemistry; Biochimica Biophysica Acta; Journal of Bacteriology; Journal of Biological Chemistry; Molecular and General Genetics; Photochemistry and Photobiology; Photosynthesis Research; Plant Molecular Biology; Plant Physiology; Proceedings of National Academy of Science USA.*

1993 *Archives of Microbiology; Biochemistry; Biochimica Biophysica Acta; Journal of Bacteriology; Journal of Biological Chemistry; Journal of Phycology; Nature; Photosynthesis Research; Physiologia Plantarum; The Plant Cell.*

1994 *Archives of Microbiology; Biochemistry; FEBS Letters; Journal of Bacteriology; Photosynthesis Research; Planta; Plant Physiology; The Plant Cell.*

1995 *Archives of Microbiology; Biochemistry; Journal of Bacteriology; Journal of Biological Chemistry; Photosynthesis Research; Physiologia Plantarum.*

1996 *Archives of Microbiology; Biochemistry; FEBS Letters; Journal of Bacteriology; Journal of Biological Chemistry; Photosynthesis Research; Proceedings of the National Academy of Sciences USA.*

1997 *Archives of Microbiology; Biochemistry International; EMBO Journal; Journal of Bacteriology; Journal of Phycology; Molecular Microbiology; Photosynthesis Research; Plant Physiology.*

1998 *Archives of Microbiology; Biochemistry; Journal of Molecular Biology; Nucleic Acids Research; Photosynthesis Research; Plant Physiology; Proceedings of the National Academy of Science USA.*

1999 *Biochemistry; FEBS Letters; FEMS Microbiological Letters; Journal of Bacteriology; Molecular Microbiology; Proceedings of the National Academy of Science USA*

2000 *Journal of Bacteriology; Journal of Phycology; Photochemistry and Photobiology; Photosynthesis Research; Plant Physiology; Trends in Plant Sciences.*

2001 *Biochemistry, Bioinformatics Journal; Journal of Bacteriology; The Plant Cell, Plant Molecular Biology; Proceedings of National Academy of Sciences, USA.*

2002 *Biochemistry; FEBS Letters; Journal of Bacteriology; Journal of Biological Chemistry; Molecular Microbiology; Photosynthesis Research; Proceedings of the National Academy of Sciences USA,*

2003 *Archives of Microbiology; Biochemistry; FEBS Letters; Journal of Bacteriology; Journal of Phycology; Molecular Microbiology; Photochemistry and Photobiology; Photosynthesis Research; Proceedings of the National Academy of Sciences USA.*

2004 *Applied and Environmental Microbiology; Biochemistry; Biochimica Biophysica Acta; Journal of Bacteriology; Photochemistry and Photobiology; Plant Molecular Biology; Molecular Microbiology; Photosynthesis Research; The Plant Cell; Plant Physiology.*

2005 *Archives of Microbiology; FEBS Letters; Journal of Bacteriology; Journal of Biological Chemistry; Journal of Molecular Evolution; Photosynthesis Research; Plant Physiology; Science.*

2006 *Applied and Environmental Microbiology; Archives of Microbiology; Biochemistry; Biochimica Biophysica Acta; Environmental Microbiology; FEBS Letters; Journal of Bacteriology (2); Journal of Biological Chemistry (2); Journal of Molecular Biology and Evolution; Molecular Microbiology; Nature; Photosynthesis Research (2); The Plant Cell; Plant Physiology; Proceedings of the National Academy of Sciences USA* (other invitations declined: *Archives of Microbiology, Biochemistry, Journal of Bioenergetics and Biomembranes, Marine Biotechnology; Microbiology*)

2007 *Biochemistry* (1); *Biochimica Biophysica Acta* (declined); BMC *Microbiology* (1); *Environmental Microbiology* (1); *Genome Biology* (1); *International Journal of Hydrogen Energy* (1) *Journal of Bacteriology* (3); *Journal of Biological Chemistry; Journal of Plant Physiology (declined); Langmuir* (1); *Microbiology* (1); *Microbiology and Molecular Biology Reviews; Molecular Microbiology* (2); *Plant Biology* (1); *Proceedings of the National Academy of Sciences, USA* (2), *Trends in Microbiology* (1).

2008 *Applied and Environmental Microbiology* (1 declined); *Applied Microbiology and Biotechnology*

(1); *Aquatic Microbial Ecology* (1 declined); *Biochimica Biophysica Acta* (2 declined); *BMC Evolutionary Biology* (1 declined); *BMC Plant Biology* (1); *Environmental Microbiology* (2; 1 declined); *European Journal of Phycology* (1 declined); *Genome Research* (1 declined); *Journal of Applied Microbiology* (1 declined); *Journal of Bacteriology* (4; 3 declined); *Journal of Biological Chemistry* (41, 1 declined); *Journal of Photochemistry and Photobiology,* Part B (1 declined); *Journal of Plant Physiology* (1); *Microbiology*, (declined invitation to join editorial board); *Molecular Biology and Evolution* (1); *Photosynthesis Research* (2 declined); *Plant Physiology* (4 declined); *Proceedings of the National Academy of Sciences USA* (4; 1 served as editor and reviewer; 2 declined); *Science* (2);

2009 *Analytical Biochemistry* (1 declined); *Applied and Environmental Microbiology* (1); *Astrobiology* (1 declined); *Biochimica Biophysica Acta* Bioenergetics (1 declined); *BMC Evolutionary Biology* (1); BMC Genomics (1 declined); *Environmental Microbiology* (1), *Genome Biology and Evolution* (1 declined); *International Journal of Hydrogen Energy* (1). *Journal of Bacteriology* (4; 4 declined); *Journal of Biological Chemistry* (26; 2 declined);

*Journal of Natural Products* (1); Microbiology (1 declined); *Microbiology and Molecular Biology Reviews* (1 declined); Molecular Microbiology (1 declined); *Photosynthesis Research* (1; 1 declined); *Plant Physiology* (1; 1 declined); *PLOS One* (2); *Proceedings of the National Academy of Sciences USA* (5; 1 as editor; 1 declined). Invitation to join editorial boards of *Molecular Biology International* and several Bentham journals (declined); Invitation to review *The Physiology and Biochemistry of Prokaryotes* David White, 4^th^ edition, Oxford Press, (declined).

2010 *Applied and Environmental Microbiology* (2; 2 declined); *Archives of Microbiology* (1); *Biochemistry* (1; 1 declined); *Biochimica Biophysica Acta Bioenergetics* (1 declined); *FEMS Microbiological Letters* (1, declined); *International Journal of Hydrogen Energy* (1 declined); *Journal of the American Chemical Society* (2); *Journal of Bacteriology* (2 declined); *Journal of Biological Chemistry* (14; 2 declined); *Journal of Lipids* (one declined); Marine Drugs (1 declined); *Molecular Microbiology* (1); *Photosynthesis Research* (1; 2 declined); *The Plant Cell* (2 declined); *Plant Physiology* (1; 3 declined); *PLOS One* (1, declined); *Proceedings of the National Academy of Sciences USA* (3; 2 as editor; 3 declined); *Science* (3);.

2011 *African Journal of Biotechnology* (3 declined); *Applied and Environmental Microbiology* (1; 2 declined); *Applied Microbiology and Biotechnology* (1 declined); *Archives of Microbiology* (1); *Biochemistry* (3); *Biochimica Biophysica Acta Bioenergetics* (3; 1 declined); *ChemSusChem* (1 declined); *Chiang Mai Journal of Science* (1 declined); *Comparative and Functional Genomics* (1 declined); *Environmental Microbiology* (2); *Frontiers in Microbiology* (4); *Geobiology* (1 declined); *ISME Journal* (2); *Journal of Bacteriology* (2 declined); *Journal of Biological Chemistry* (2; 1 declined); *Journal of Phycology* (1); *Marine Drugs* (1 declined); *Molecular*

*Biotechnology* (1 declined)*Photosynthesis Research* (1; 2 declined); *Physiologia Plantarum* (1 declined); *Phycological Research* (1); *The Plant Cell* (1 declined); *Plant Cell Letters* (1 declined); *The Plant Journal* (1 declined);*Plant Physiology* (1; 1 declined); *PLoS Genetics* (1

declined); *PLoS One* (2; 1 declined); *Proteomics* (1 declined);.

2012 *African Journal of Biochemistry* (1 declined); *African Journal of Microbiology* (1 declined); *Applied and Environmental Microbiology* (2; 1 declined); *Biochemical Journal* (1); *Biochimica Biophysica Acta* (1); *Canadian Journal of Microbiology* (1 declined); *FEBS Letters* (1; 1 declined); *FEBS OpenBio* (1 declined); *Frontiers of Microbiology* (1); *International Journal of Hydrogen Energy* (1 declined); *Journal of Bacteriology* (2 declined); *Journal of Biological Chemistry* (1); *Journal of Phycology* (1); *Environmental Microbiology* (1); *Metallomics* (1 declined); *Molecular Microbiology* (2); *Plant Physiology* (2 declined); *PLoS One* (2 declined); *Proceedings of the National Academy of Sciences USA* (1).

2013 *Biochemistry* (1); *Bioscience* (1 declined); *Frontiers of Microbiology* (2); *Journal of Bacteriology* (1); *Journal of Biological Chemistry* (1 declined); *Journal of Physical Chemistry* (2 declined); *ISME Journal* (1); *Metabolic Engineering* (1 declined); *Photochemistry and Photobiology* (1); *Photosynthesis Research* (2; 1 declined); *PLoS One* (1; 3 declined); *Proceedings of the National Academy of Sciences USA* (2; 1 declined); *Sensors & Actuators: B. Chemical* (1 declined);

2014 *Applied Environmental Microbiology* (1); *Archives of Microbiology* (1 declined); *Biochemistry*

(1); *Biochimica Biophysica Acta* (2; 2 declined); *Biotechnology and Bioengineering* (1 declined); *Cell Research* (1); *Computational and Structural Biotechnology Journal* (1 declined); *Current Biology* (1 declined); *Environmental Microbiology* (1 declined); *Frontiers in Microbiology* (1; 11 declined); *Geobiology* (1); *ISME Journal* (1; 2 declined); *Journal of Biological Chemistry* (3; 1 declined); *Journal of Computational Biology and Bioinformatics Research* (1 declined);

*Journal of Industrial and Engineering Chemistry* (1 declined); *Life*, (1 declined); *Microbiology* (1 declined). *Molecular and Cellular Proteomics* (1 declined); *Molecular Microbiology* (1 declined); *Molecular Phylogenetics and Evolution* (1 declined); *Nature Scientific Reports* (3 declined); *Photosynthesis Research* (2; 1 declined); *PLoS One* (1; 1 declined); *Preparative Biochemistry and Biotechnology* (1 declined); *Proceedings of the National Academy of Sciences USA* (7; 1 declined); *Science* (1).

2015 *Algal Research* (1 declined); *Applied Environmental Microbiology* (2 declined); *Biochemistry* (1; 1 declined); *Ecotoxicology and Environmental Safety* (1 declined); *Frontiers in Microbiology* (20 declined); *ISME Journal* (2); *Journal of Bacteriology* (1 declined); *Journal of Biological Chemistry* (5); *Journal of Biological and Food Science Research* (1 declined); *Microbiology* (1); *Molecular Biology and Evolution* (1 declined); *mSysterms* (1); *Nature Communications* (1); *Photosynthesis Research* (1); *The Plant Cell* (1); *PLoS One* (1 declined); *Proceedings of the National Academy of Sciences USA* (3; 2 declined); *Process Biochemistry* (1 declined); *Science Advances* (1 declined).

2016 *Algal Research* (2 declined); *Applied and Environmental Microbiology* (2 declined); *Biochimica Biophysica Acta* (3 declined); *Biologia* Part C (1 declined); *Biotechnology for Biofuels* (1 declined); *Environmental Microbiology* (1 declined); *Frontiers of Microbiology* (2; 6 declined); *ISME Journal* (1 declined); *Journal of Biological Chemistry* (13; 1 declined); *mBio* (1 declined due to COI); *Microbiology* (1); *Molecular Plant* (1); *Photochemical & Photobiological Sciences*

(1); *Photochemistry and Photobiology* (1; 1 declined); *Photosynthesis Research* (3); *Plant and Cell Physiology* (2); *Plant Physiology* (2 declined); *PLoS One* (3 declined); *Proceedings of the National Academy of Sciences USA* (1; 1 declined); *Phyiologia Plantarum* (1 declined).

2017 *ACS Applied Nano Materials* (1 declined); *Applied and Environmental Microbiology* (1); *Applied Microbiology and Biotechnology* (2 declined; 1 COI); *Biotechnology Journal* (1 declined); *Current Bioactive Compounds* (1 declined); *Earth Science* (1 declined); *Environmental Engineering and Management Journal* (1 declined); *Environmental and Experimental Botany* (1 declined); *Environmental Microbiology* (2); *FEMS Microbiology Ecology* (1); *Food Chemistry* (1 declined); *Frontiers of Microbiology* (1; 7 declined); *Frontiers in Neuroscience* (1 declined); *Frontiers in Plant Sciences* (1 declined); *ISME Journal* (1); *Journal of Biological Chemistry* (17; 3 declined); *Journal of Photochemistry and Photobiology A: Chemistry* (1); *Journal of Physical Chemistry, B* (1 declined); *Journal of Physical Chemistry Letters* (1); *Limnologica* (1 declined); *Marine Drugs* (1 declined); *Microbiology* (2); *Molecular Biology and Evolution* (1 declined); Molecular Microbiology (1 declined); *Photochemical & Photobiological Sciences* (1 declined); *Photosynthesis Research* (3; 2 declined); *Photosynthetica*

(1); *Planta* (1 declined); *Plant and Cell Physiology* (1); *Proceedings of the National Academy of Sciences USA* (2; 2 declined (COI)); *RSC Advances* (1 declined); *Science* (1); *Scientific Reports* (2 declined); *The Plant Cell* (1 declined). **71 requests: 35 reviews completed; 36 invitations declined for 34 total journals.**

2018 *Algal Research* (1 declined); *Applied and Environmental Microbiology* (1; 2 declined); *Aquatic*

*Botany* (1 declined); *Archives of Microbiology* (1); *Biochimica Biophysica Acta* (1; 3 declined); *Biotechnology Advances* (1 declined); *Biotechnology for Biofuels* (1 declined); *BMC Genomics* (1 declined); *Current Bioactive Compounds* (1 declined); *Frontiers of Microbiology* (2; 13 declined); *Gene* (1 declined); *Genome Biology and Evolution* (1); *International Journal of Systematic and Evolutionary Microbiology* (1); *Journal of Applied Phycology* (1 declined); *Journal of Biological Chemistry* (11; 4 declined (2 coi)); *Journal of Physical Chemistry* (1 declined); *Journal of Physical Chemistry Letters* (1); *mBio* (2); *Metabolic Engineering* (1

declined); *Molecular Plant* (1); *New Phytologist* (1); *Nucleic Acids Research* (1 declined); *Photochemistry and Photobiology* (1 declined); *Photosynthesis Research* (2); *Plant Molecular Biology* (1); *PLoS One* (3 declined); *Preparative Biochemistry and Biotechnology* (1 declined); *Proceedings of the National Academy of Sciences* (3 declined; 1 coi); *Process Biochemistry* (1 declined); *Scientific Reports* (1; 1 declined); *The Plant Cell* (1 declined). **70 total requests from 31 journals; 27 reviewed; 43 declined.**

2019 *ACS Synthetic Biology* (1); *Algal Research* (1 declined); *Applied and Environmental Microbiology* (1 declined); *Bergey’s Manual of Systematics of Archaea and Bacteria* (2); *ChemBioChem* (1 declined); *Chemical Science* (1 declined); *Computational Biology and Chemistry* (1 declined); *Current Biology* (1 declined); *Environmental Microbiology* (1); *Food Chemistry* (1 declined); *Frontiers of Microbiology* (2; 1 declined); *Genome Biology and Evolution* (1 declined); *International Journal of Systematic and Evolutionary Microbiology* (1 declined); *iScience* (2 declined); *ISME Journal* (1); *Israel Journal of Chemistry* (1 declined); *Journal of Applied Algology* (1 declined); *Journal of Biological Chemistry* (11; 1 declined)); *mSystems* (1); *Nature* (2); *Nature Communications* (1 declined); *Proceedings of the National Academy of Sciences* (4 declined (2 coi)); *Science* (1 declined); *Scientific Reports* (2 declined); **44 total requests from 24 journals; 21 reviewed; 23 declined.**

2020 *Algal Research* (1 declined); *Archives of Microbiology* (2); *Biochemical Journal* (1); *Biochemistry* (1 declined coi); *Biochimica Biophysica Acta—Bioenergetics* (4 declined); *BMC Microbiome* (1); *Communications Biology* (1); *Computational and Structural Biotechnology Journal* (1 declined); *Environmental Technology & Innovation* (1 declined); *Enzyme and Microbial Technology* (1 declined); *FEBS Letters* (2 declined); *Frontiers in Genetics* (1 declined); *Frontiers in Microbiology* (4; 5 declined); *Industrial Crops and Products* (1 declined); *International Journal of Hydrogen Energy* (1 declined); *ISME J* (1); *Journal of Biological Chemistry* (9; 4 declined); *Journal of Genomics* (1); *Journal of Photochemistry and Photobiology A:Chemistry* (1 declined); *Journal of the Saudi Society of Agricultural Sciences* (1 declined); *Metabolic Engineering* (1 declined); *Nature Communications* (2; 1 declined); *New Phytologist* (1 declined); *Photochemical and Photobiological Sciences* (2 declined; 1 coi); *Photosynthesis Research* (2); *Plant Physiology and Biochemistry* (1 declined); *Proceedings of the National Academy of Sciences* (1; 5 declined (3 coi)); *Process Biochemistry* (1 declined); *Science Advances*, (1); *Toxicon* (2 declined); *Trends in Plant Sciences* (1). **65 total requests from 30 journals: 27 reviewed; 38 declined.**

2021 *Applied and Environmental Microbiology* (1 declined); *Biochimica Biophysica Acta— Bioenergetics* (1 declined); *Communications Biology* (2); *Computational and Structural Biotechnology Journal* (1 declined); *eLife* (1 declined); *Environmental and Experimental Botany* (1 declined); *Environmental Research* (1 declined); *Expert Review of Proteomics* (1 declined); *FEMS Microbiological Letters* (1 declined); *Frontiers in Microbiology* (1; 1 declined); *Frontiers in Plant Science* (1 declined); *iScience* (1 declined); *Journal of Bacteriology* (1); *Journal of Experimental Marine Biology and Ecology* (2 declined); *Journal of Integrative Plant Biology (*1 declined); *Journal of Pure and Applied Microbiology* (1 declined); *Journal of the Royal Society Interface* (1 declined); *Journal of Visualized Experiments* (1 declined); *mBio* (2 declined); *Metabolic Engineering* (2 declined); *Microbial Ecology* (1 declined); *Microbiology Spectrum* (1 declined); *Microbiome* (1); *Molecular Biology and Evolution* (1 declined); *mSphere* (1; 1

declined (coi)); *Nature* (2); *Nature Communications* (1; 1 declined); *New Phytologist*, (1); *Photosynthesis Research* (1 declined, coi); *PLoS One* (1, declined); *Proceedings of the National Academy of Sciences USA* (2 declined); *Royal Society Open Science* (1 declined); *Science*

*Advances* (2; 1 declined); *Scientific Reports* (2 declined); *The Plant Cell* (1). **47 total requests**

### from 35 journals: 13 reviewed; 34 declined.

2022 *ACS Central Science* (1 declined); *Advanced Biology* (1 declined); *Applied and Environmental Microbiology* (1 declined); *Bioconjugate Chemistry* (1 declined); *Biomolecular Concepts* (1 declined); *Bioscience Reports* (1 declined); *BMC Biology* (1 declined); *Chemistry of Materials* (1 declined); *FEBS Open Bio* (1 declined); *FEMS Microbiology Ecology* (2); *Frontiers in Astronomy and Space Sciences* (2 declined); *Frontiers in Marine Science* (1 declined); *Journal of Applied Phycology* (1 declined); *Journal of Pure and Applied Microbiology* (1 declined); *mBio* (1; 2 declined); *Nature Communications* (2; 1 declined); *Photochemical & Photobiological Sciences* (1 declined); *Photosynthesis Research* (1 declined); *Photosynthetica* (1 declined); *PLoS One* (2; 2 declined); *Proceedings of the National Academy of Sciences USA* (4 declined); *Protein Science* (1 declined); *Science Advances* (1). **35 total requests from 23 journals: 09 reviewed;**

### 26 declined.

2023 *ACS Synthetic Biology* (1 declined; coi); *Communications Biology* (1); *Ecotoxicology and Environental Safety* (1 declined); *Environmental and Experimental Botany* (1 declined); *Frontiers of Microbiology* (1 declined); *Journal of Pure and Applied Microbiology* (1 declined); *Letters in Applied Microbiology* (1 declined); *Microbial Cell* (1 declined); *Molecular Ecology Resources* (1 declined); *Nature Plants* (1 declined); *Open Biology* (1 declined); *Plant Physiology* (1 declined); *Plant Physiology and Biochemistry* (3 declined); *Proceedings of the National Academy of Sciences USA* (1 declined); *Progress in Biophysics and Molecular Biology* (1 declined); *Science* (2); *The Plant Journal* (1 declined). **20 total requests from 17 journals: 03**

### reviewed; 17 declined.

2024 *Plant Physiology* (1 declined). **01 total requests from 01 journals: 00 reviewed; 01 declined.**

## Grants

| 1980 | National Science Foundation |
| --- | --- |
| 1983 | National Science Foundation |
| 1984 | U.S. Dept. of Agriculture; National Science Foundation; Petroleum Research Fund. |
| 1985 | U. S. Dept. of Agriculture; National Science Foundation; Ad Hoc Review Committee, National |
|  | Cancer Institute |
| 1986 | U. S. Dept. of Agriculture; National Science Foundation; National Cancer Institute; Department |
|  | of Energy. |
| 1987 | U. S. Dept. of Agriculture; National Science Foundation; National Sciences and Engineering |
|  | Research Council (Canada). |
| 1988 | U. S. Dept. of Agriculture (U.S.D.A. Photosynthesis Panel Member); National Institutes of |
|  | Health; National Science Foundation; Department of Energy; National Sciences and Engineering |
|  | Research Council (Canada). |
| 1989 | U. S. Dept. of Agriculture; National Science Foundation; National Institutes of Health; Dept. of |
|  | Energy; State of Louisiana; State of Washington Sea Grants Program. |
| 1990 | U. S. Dept. of Agriculture; National Science Foundation; National Institutes of Health; Dept of |
|  | Energy (*ad hoc* reviewer and panel member); National Sciences and Engineering Research |
|  | Council (Canada). |

1991 National Science Foundation; Dept. of Energy; U. S. Dept. of Agriculture; U. S. Army Research Office; North Carolina Biotechnology Center; National Sciences and Engineering Research Council (Canada); Agriculture and Food Research Council, United Kingdom.

1992 National Science Foundation; National Institutes of Health; Dept. of Energy; U. S. Dept. of Agriculture.

1993 National Science Foundation; National Institutes of Health; Dept. of Energy; U. S. Dept. of Agriculture; National Sciences and Engineering Research Council (Canada); Human Frontier Science Program.

1994 National Science Foundation; National Institutes of Health; U. S. Dept. of Agriculture; National Sciences and Engineering Research Council (Canada); Binational Science Foundation; Dept. of Energy; Human Frontier Science Program.

1995 National Science Foundation; National Institutes of Health; U. S. Dept. of Agriculture; National Sciences and Engineering Research Council (Canada); Dept. of Energy.

1996 National Institutes of Health; Natural Environment Research Council (U. K.).

1997 National Institutes of Health; Dept. of Energy; National Science Foundation, Human Frontier Science Program.

1998 National Institutes of Health; American Chemical Society; National Science Foundation; U. S. Dept. of Agriculture, Dept. of Energy

1999 National Science Foundation; U. S. Dept. of Agriculture; Dept. of Energy; National Institutes of Health

2000 National Institutes of Health; National Science Foundation; Dept. of Energy; U. S. Dept. of Agriculture; NASA Exobiology Program; Ohio Incentive Grant

2001 National Science Foundation, Israel Science Foundation

2002 National Science Foundation, Israel Science Foundation, U. S. Dept. of Agriculture

2003 National Science Foundation, U. S. Dept. of Agriculture, Ohio Plant Biotechnology Consortium, National Institutes of Health,

2004 National Science Foundation, U. S. Department of Agriculture; Department of Energy 2005 National Science Foundation, U. S. Department of Agriculture, Department of Energy

2006 National Science Foundation, Department of Energy, Ohio Incentive Grant, U. S. Dept. of Agriculture

2007 Department of Energy (~10), Natural Environment Research Council (UK; 1), National Science Foundation (3), Foundation for Fundamental Research on Matter (FOM; The Netherlands); Biotechnology and Biological Sciences Research Council (UK; 1).

2008 National Science Foundation (6; 2 declined), Marsden Fund (1; (New Zealand)), Dept. of Defense (1 declined)

2009 Agence Nationale de la Recherche (1 declined); National Science Foundation (6); Joint Genome Institute/DOE (13); Dept. of Energy (1); Marsden Fund (New Zealand) (1 declined); Province of British Columbia, Natural Resources and Applied Sciences (NRAS) Endowment (1 declined); DOE-ARRA (~10 *ad hoc* declined—not within my area of expertise)

2010 Joint Genome Institute/DOE (13); National Science Foundation (3; 1 declined); Natural Environmental Research Council (UK; 1 declined).

2011 Joint Genome Institute (13); Academia Sinica (Taiwan) (1); NASA Astrobiology/Exobiology (10); National Science Foundation (3; 1 declined); Biotechnology and Biochemistry Sciences Research Council-UK (1); US-Israel Binational Science Foundation (1 declined).

2012 Dept. of Energy (1 declined).

2013 UK University Research Fellowship (1 declined); National Science Foundation (2; 1 declined); Stanford University Global Climate and Energy Project (1); Joint Genome Institute/DOE (6).

2014 Deutsche Forschungsgemeinschaft (1 declined); National Science Foundation (1); Austrian Science Fund (1 declined); Bilateral NSF/BIO-BBSRC (1 declined).

2015 National Science Foundation (1 declined); Czech Science Foundation (1 declined); Icelandic Research Fund (1 declined)

2016 National Science Foundation (3; 1 declined); National Aeronautic and Space Administration Exobiology (1); Netherlands Organisation for Scientific Research (1 declined); Rutherford Discovery Fellowship/New Zealand (1).

2017 National Science Foundation (13 as panel member; 1 *ad hoc*; 2 *ad hoc* declined); Israel National Science Foundation (1 declined); Netherlands Organisation for Scientific Research (1 declined);

2018 Graduate Women in Science (1); National Science Foundation (1 declined (COI)); Chilean National Science Foundation (1 declined); Hudson River Foundation (1 declined). European Research Council (1 declined).

2019 US-Israel Binational Science Foundation (1 declined); Austrian Science Fund (1 declined); DOE Early Career Program (3 *ad hoc*); European Research Council (1 declined); Polish National Science Centre (1 declined).

2020 UK Biotechnology and Biomedical Science Research Council (1 declined, COI); Austrian Science Fund (1 declined, COI); National Science Foundation (1 proposal, *ad hoc*). Lewis and Clark Fund in Astrobiology, American Philosophical Society (2 proposals, *ad hoc*).

2021 Austrian Science Fund (1 declined); Einstein International Postdoctoral Fellowship (1); Biotechnology and Biological Sciences Research Council UK (1 declined).

2022 Deutsche Forschungsgemeinschaft (2 declined); Einstein International Postdoctoral Fellowship

(1); Polish National Science Foundation (1 declined).

2023 Research Support Office, Nanyang Technological University (1); Dept. of Energy Panel invitation (declined); Czech Science Foundation (1 declined, COI).

# Grant Support (Completed and Current)

1977-1979 N.S.F (NATO)-C.N.R.S. (U.S.--France Exchange Program) postdoctoral fellowship.

1982-1983 *Regulation of Phycocyanin Biosynthesis in Cyanobacteria.* PI, Donald A. Bryant*.* Research Initiation Grant Award, The Pennsylvania State University. $5000, total costs.

1982-1986 *Metabolic Regulation in Cyanobacteria.* PI, Donald A. Bryant*.* Agriculture Experiment Station, The Pennsylvania State University. Project #2612. $16,000 annual costs.

1983-1986 *Regulation of Biliprotein Biosynthesis in Cyanobacteria.* PI, Donald A. Bryant *(*with Co- PIs: Drs. R. D. Porter and S. E. Stevens, Jr.) USPHS GM-31625-(01-03). $208,601 total direct costs.

1983-1986 *Structure-function Studies on Cyanobacterial Phycobilisomes and Chlorophyll-Proteins.*

PI, Donald A. Bryant*.* U. S. Dept. of Agriculture, 83-CRCR-1-1336. $62,000 total costs.

1985-1986 *The Development and Production of Novel Polymeric Materials Using Recombinant DNA Technology and Synthetic Polymer Chemistry,* with six other faculty from three colleges. The Pennsylvania State University, Colleges of Science, Engineering, and Earth and Mineral Sciences. $14,500 total costs.

1985-1986 *Isolation and Characterization of the Genes Encoding the HU Proteins of* E. coli*.* Co-PI, Donald A. Bryant (With Dr. R. D. Porter). Biomedical Research Grants Program, The Pennsylvania State University. $10,000 total costs.

1985-1988 *Development of Merodiploid Analysis in Cyanobacteria.* Co-PI, Donald A. Bryant *(*with Co-PIs: Drs. R. D. Porter and S. E. Stevens, Jr.) NSF/DMB-8511132. $225,000 total costs.

1986-1989 *Genes for Photosystems Components in Cyanobacteria and Cyanelles.* PI, Donald A. Bryant*.* N.S.F. DMB-8504294. $100,000 total costs.

1986-1989 *Regulation of Biliprotein Biosynthesis in Cyanobacteria.* PI, Donald A. Bryant*.* USPHS NIH GM-31625-(04-06). $222,009 total direct costs.

1986-1988 *Gene Regulation in Cyanobacteria.* PI, Donald A. Bryant*.* NSF/NATO (U.S.--France Exchange Program, INT-8514249. $8,110 total costs.

1986-1987 Anonymous donation for research on cyanobacterial physiology and genetics, with Drs. S.

E. Stevens, Jr. and R. D. Porter. $38,988 total direct costs.

1986-1990 *Control of Photosynthetic Gene Expression in Synechococcus PCC 7002.* PI, Donald A. Bryant*.* Agriculture Experiment Station, The Pennsylvania State University, $22,500 annual costs.

USPHS/NIH GM-31625-(07-11). $597,153 total direct costs.

1989-1992 *Genes for Photosystems Components in Cyanobacteria and Cyanelles.* PI, Donald A. Bryant*.* NSF DMB-8818997. $240,000 total costs.

1990-1993 *Control of Photosynthetic Gene Expression in Synechococcus sp PCC 7002.* PI, Donald A. Bryant*.* Agriculture Experiment Station, The Pennsylvania State University, $22,500 annual total costs.

1991 Tenth Penn State Summer Symposium in Molecular Biology. U. S. Department of Agriculture. R. J. Frisque (PI), J. Medford (Co-PI), T. Kao (Co-PI), and D. A. Bryant (Co- PI). $4000 total costs.

1991-1994 *The Cyanelle Genome: An Evolutionary Legacy of Plant Genes.* Co-PI, Donald A. Bryant*.* USDA (Subcontract to University of Arizona, with PI: Dr. Hans J. Bohnert). $150,000 total costs; Penn State subcontract, $70,000 total costs.

1992-1997 *Structural and functional analysis of Photosystem I.* PI, Donald A. Bryant*.* NSF MCB-92- 06851. Total costs, $415,000. Period: Sept. 1, 1992 to Aug. 31, 1997.

1994-1998 *Regulation of Biliprotein Biosynthesis in Cyanobacteria.* PI, Donald A. Bryant*.* USPHS/NIH GM-31625-(12-15). $611,987 total direct costs. Period: March 1, 1994 to Feb. 28, 1998.

1994-1996 *Light-Energy Transduction in Green Sulfur Bacteria.* PI, Donald A. Bryant*.* DOE.

$228,350 total costs. April 1, 1994 to March 31, 1997.

1997-2000 *Light-Energy Transduction in Green Sulfur Bacteria.* DOE. PI, Donald A. Bryant*.* Total costs, $270,000. Period: April 1, 1997 to March 31, 2000.

1997-2000 *Structural and Functional Analysis of Photosystem I.* PI, Donald A. Bryant*.* NSF MCB- 9723469. Total costs, $240,000. Period: Sept. 1, 1997 to Aug. 31, 2000. REU supplement:

$5,000 (1998) REU and ROA Supplements: $20,000 (1999). NSF Eastern European Program and REU supplement to grant MCB-9723469. $24,124. (2000).

1997-2000 *Photosynthetic and Respiratory Electron Transport in Cyanobacteria.* Co-PI, Donald A. Bryant. Human Frontier Science Program RG 0051/1997 M (W. Vermaas, Arizona State, PI). Total costs, $120,000. Period: July 1, 1997 to December 31, 2000.

1998-1999 *Regulation of Biliprotein Biosynthesis in Cyanobacteria.* PI, Donald A. Bryant*.* USPHS/NIH GM-31625-(12-15). $73,100 total costs. Period: March 1, 1998 to Feb. 28, 1999.

USPHS/NIH GM-31625-(16-19). Total costs, $1,158,713. Period: March 1, 1999 to Feb. 28, 2004.

2000-2002 *A Photosystem I/organometallic hybrid complex for hydrogen reduction.* Co-PI, Donald A. Bryant*.* The Pennsylvania State University Seed Grant Program (with Co-PI John H. Golbeck). Total costs, $33,500. Period: July 1, 2000 to June 30, 2002.

2000-2001 *Light-energy transduction in green sulfur bacteria.* PI, Donald A. Bryant*.* DOE. Total Costs, $25,000. Administrative supplement to existing grant. April 1, 2000 to March 31, 2001.

2000-2005 *Structure, Function, and Biogenesis of Cyanobacterial Photosystem I.* PI, Donald A. Bryant*.* NSF-MCB-0077586. Total Costs, $600,000. Period: Sept. 1, 2000 to August 31, 2005. REU supplement, $5,000 (2001). REU supplement, $10,000 (2002). REU supplement, $10,000 (2003). ROA supplement, $15,000 (2004); REU supplement, $10,000 (2004).

2001-2004 *Light-energy Transduction in Green Sulfur Bacteria.* PI, Donald A. Bryant*.* DOE DE- FG02-94ER20137. Total Costs $360,000. Period: April 1, 2001 to March 31, 2004.

$15,000 equipment supplement, (2001).

2002-2003 *Biochemical Aspects of Photosynthesis,* Gordon Research Conference. PI, Donald A. Bryant*.* Dept. of Energy. Total Costs, $4,000. Period: 5-1-02 to 4-30-03.

2002-2003 *Biochemical Aspects of Photosynthesis,* Gordon Research Conference. PI, Donald A. Bryant*.* U. S. Dept. of Agriculture, Total costs, $8,000. Period: 5-1-02 to 4-30-03.

2002-2003 *Genome sequence analysis of the phototactic bacterial consortium “Chlorochromatium aggregatum.”* PI, Donald A. Bryant (with Co-PI: Dr. Jörg Overmann). Joint Genome Institute-Department of Energy. Approved. Funds to JGI/DOE.

2003-2004 *Genome Sequence Analysis of Eight Green Sulfur Bacteria.* Joint Genome Institute- Department of Energy. PI, Donald A. Bryant (with Co-PIs: Drs. Jörg Overmann and R. E. Blankenship). Approved. Funds to JGI/DOE.

2003-2006 *Establishment of a Center for Metallobiochemistry*, Huck Institutes of the Life Sciences, Penn State University. J. H. Golbeck, PI. D. A. Bryant, J. M. Bollinger, C. Krebs, S. Booker, M. Green, Co-PIs. $300,000 total costs.

2004-2005 *Genome Sequence Analysis of Seven Strains of Chloroflexi, Filamentous Anoxygenic Phototrophs.* Joint Genome Institute, Department of Energy. PI, Donald A. Bryant (with Co-PI, Dr. Jason Raymond). Approved. Funds to JGI/DOE.

2004-2008 *Light-energy Transduction in Green Sulfur Bacteria.* PI, Donald A. Bryant*.* Department of Energy, Basic Energy Sciences, Energy Biosciences. DE-FG02-94ER20137. Total costs,

$560,000. Period: April 1, 2004 to March 31, 2008.

2004-2009 *Biogenesis of Photosystem I in Cyanobacteria and Higher Plants.* PI, Donald A. Bryant. USDA 2005-35318-15284. John H. Golbeck, PI; Donald A. Bryant, Co-PI. Total costs,

$442,000. Period: October 1, 2004 to November 30, 2008. No-cost extension to November

30, 2009.

2005-2011 *Renewable Bio-solar Hydrogen Production from Robust Oxygenic Phototrophs.* PI: G. Charles Dismukes, Princeton University. Co-PIs: Edward Stiefel (Princeton University), Donald A. Bryant, Matthew Posewitz (Colorado School of Mines), Eric Hegg (Michigan State University), and Robert Bidigare (University of Hawaii). Air Force Office of Scientific Research, FA9550-05-1-0365. Total Costs, $5,500,000 (Supplement awarded January, 2008: $357,388). Total costs to PSU/DAB: $1,158,908. Period: May 15, 2005 to May 14, 2010. No-cost extension granted from May 16, 2010 to May 14, 2011.

2005-2008 *A Hybrid Biological/Organic Half-Cell for Generating Dihydrogen.* John H. Golbeck, PI; Donald A. Bryant, Co-PI. Department of Energy, Basic Energy Sciences DE-FG-02-05- ER46222. Total costs, $525,000. Period: September 15, 2005 to September 14, 2008.

2005-2010 *Photosystem I: Biogenesis, Broken Symmetry, and Hydrogenase Chimeras.* National Science Foundation, MCB-0519743. Donald A. Bryant, PI; John H. Golbeck, Co-PI. Total costs, $1,230,000. Period: September 1, 2005 to October 31, 2010. 2006 REU Supplement, $10,000. 2007 REU supplement, $5000. 2008 REU supplement, $5000.

2005-2009 *Complete Genome Sequences of Green Bacteria*. Donald A. Bryant, PI. Co-PI: Stephan

C. Schuster. National Science Foundation, MCB-0523100. Total Costs: $822,038. Period: September 1, 2005 to August 31, 2008. (Supplement: 2007-2009: $63,479). No-

cost extension to August 31, 2009.

2007-2008 *Genome sequence analysis of two strains of Cyanobacteria for biosolar hydrogen production and biomass/biofuel applications*. PI, Donald A. Bryant. Joint Genome Institute, Dept. of Energy. Total costs: none—all costs to JGI-DOE. Period: 01/01-2007 to 12/31/08.

2007-2009 *The Yellowstone metagenome project: Biological metagenomics and bioinspired energy and technology development from extreme microbial habitats across the Yellowstone geothermal ecosystem.* William Inskeep, (Montana State University), PI. (Donald A. Bryant is a member of the YNP Metagenomics Working Group and NSF Yellowstone Research Coordination Network). JGI-DOE. Period: July 1, 2007 to June 30, 2009.

2008-2012 *Light Energy Transduction in Green Sulfur Bacteria.* PI, Donald A. Bryant*.* Department of Energy, Basic Energy Sciences, Energy Biosciences. DE-FG-02-94ER20137. Total costs, $1,117,812. Period: April 1, 2008 to March 31, 2012.

2008-2011 *A Hybrid Biological/Organic Half-Cell for Generating Dihydrogen.* John H. Golbeck, PI; Donald A. Bryant, Co-PI. Department of Energy, Basic Energy Sciences. DE-FG-02-05- ER46222. Period: September 15, 2008 to September 14, 2011. Total costs, $750,000.

2009-2012 *Molecular and geochemical analysis of anoxygenic phototrophic bacteria in hot spring mats as stromatolite analogs.* NASA Astrobiology: Exobiology and Evolutionary Biology, Award NNX09AM87G. David M. Ward (Montana State University), PI; Donald A. Bryant, Co-PI; Period: June 22, 2009 to June 21, 2012. Total costs, $684,194; total costs to D.A.B, $330,706.

2009-2012 *Solar to liquid fuels production: light-driven reduction of carbon dioxide to formic acid.* Air Force Office of Scientific Research. John H. Golbeck, PI; Donald A. Bryant, Co-PI. Period: September 1, 2009 to August 31, 2012. Total Costs, $1,072,262.

2009-2011 *Genome sequencing of representative photosynthetic purple sulfur bacteria.* Co-PIs: Niels-Ulrik Frigaard and Donald A. Bryant. Community sequencing program, JGI-DOE. Total costs: none—all costs to JGI-DOE.

2009-2011 *Species diversity: the fundamental basis for efficient energy capture in a model photosynthetic microbial community.* PI: David M. Ward; Co-PIs: Donald A. Bryant, Frederick M. Cohan, and Douglas B. Rusch. Community sequencing program, JGI-DOE. Total costs: none—all costs to JGI-DOE.

2010-2013 *Biological systems interactions and genome-enabled studies of photosynthetic microorganisms for bioenergy applications.* U. S. Dept. of Energy, Genomics:GTL Foundational Science Focus Area (FSFA) and Biofuels Scientific Focus Area (BSFA), Pacific Northwest National Laboratory. PIs: James K. Fredrickson and Alexander S. Beliaev. Collaborator and Co-PI, Donald A. Bryant. Total costs to Penn State, $750,000 ($250,000 per year).

2010-2016 *Type-1 photochemical reaction centers: paradigm, variations, and applications.* National Science Foundation MCB-1021725. Donald A. Bryant, PI; John H. Golbeck, Co-PI. Period: Sept. 1, 2010 to August 31, 2015. No cost extension to August 31, 2016. Total costs $1,774,000.

2010-2012 *Metagenomic and metatranscriptomic analysis of anoxygenic, chlorophototrophic microbial mat communities in Yellowstone National Park.* Co-PIs: Donald A. Bryant and David M. Ward (Montana State University). Period: September 1, 2010 to August 31, 2012. All costs to Joint Genome Institute, Dept. of Energy.

2011-2014 *Renewable Bio-solar hydrogen production from robust oxygenic phototrophs: second generation.* Air Force Office of Scientific Research, FA9550-11-1-0148. PI: Donald A. Bryant. Period: May 15, 2011 to December 31, 2014. Total costs, $750,000. [This is part of a larger project with Co-PIs G. Charles Dismukes (Rutgers University), John W. Peters (Montana State University), and Matthew C. Posewitz (Colorado School of Mines). Total costs, $3,266,477].

2011-2017 *A Hybrid Biological/Organic Half-Cell for Generating Dihydrogen.* John H. Golbeck, PI; Donald A. Bryant, Co-PI. Department of Energy, Basic Energy Sciences. DE-FG-02-05- ER46222. Period: September 15, 2011 to September 14, 2014; no-cost extension until

September 14, 2016. Total costs: $915,003.

2011-2015 *PNNL Foundational Scientific Focus Area: Biological Systems Interactions.* PIs: Jim Fredrickson and Margaret Romine. Donald A. Bryant, External Co-PI (with others). Department of Energy, BER Genomics: GTL. Proposed period: October 1, 2011 to 2015. Totals Costs to PSU: $150,000 in 2011, 2012, 2013; $220,000 in 2014.

2012-2015 *Light Energy Transduction in Green (Sulfur) Bacteria.* PI, Donald A. Bryant*.* Department of Energy, Basic Energy Sciences, Energy Biosciences. Renewal of DE-FG02- 94ER20137. Basic Energy Sciences. Period: June 1, 2012 to May 31, 2015 (No-cost extension to October 31, 2015). Total Costs: $892,000.

2014-2018 *Photosynthetic Antenna Research Center 2 (PARC2).* Lead PI: Robert E. Blankenship. Department of Energy, Energy Frontier Research Center. Period: August 1, 2014 to July 31, 2018. This project will provide $540,000 in total costs over four years to D.A.B./PSU.

2015-2019 *Light Energy Transduction in Green (Sulfur) Bacteria.* PI, Donald A. Bryant*.* Department of Energy, Basic Energy Sciences, Energy Biosciences. Renewal of DE-FG02- 94ER20137. Basic Energy Sciences. Period: November 1, 2015 to October 31, 2018. Total requested costs: $550,000. No-cost extension, November 1, 2018 to October 31, 2019. $50,000 Administrative Supplement, 2019-2020.

2016-2020 *Molecular and geochemical analyses of metabolisms and carbon isotopic fractionations in laminated, anoxygenic phototrophic microbial mats and isolates representative of their native taxa*. NASA-Exobiology NNX16AJ62G. May 1, 2016 to April 30, 2020. Total requested costs: $1,080,000; total costs to PSU/Bryant: $242,488.

2016-2022 *Acclimation responses that optimize the photosynthetic apparatus in cyanobacteria: from ecophysiology to biophysics.* National Science Foundation MCB-1613022. Proposed Period: July 15, 2016 to June 30, 2021. No-cost extension to June 30, 2022. Total costs:

$1,900,000. ($1,165,562 to D. A. B.)

2016-2017 *A Hybrid Biological/Organic Half-Cell for Generating Dihydrogen.* John H. Golbeck, PI; Donald A. Bryant Co-PI. Department of Energy, Basic Energy Sciences. DE-FG-02-05- ER46222. Proposed period: September 15, 2016 to September 14, 2017. Total costs:

$100,000.

2017-2020 *Fundamental research aimed at diverting excess reducing power in photosynthesis to orthogonal metabolic pathways.* John H. Golbeck, PI; Donald A. Bryant Co-PI. Department of Energy, Basic Energy Sciences. DE-SC00118087. Proposed period: August 1, 2017 to July 31, 2020. Total costs, $650,000.

2019 *Light Energy Transduction in Green (Sulfur) Bacteria.* PI, Donald A. Bryant*.* Department of Energy, Basic Energy Sciences, Energy Biosciences. January 1, 2019 to October 31, 2019. Administrative Supplement, $49,513.

2021 *Using protein-stable isotope probing to link taxa and metabolic function in photic microbial communities.* PI, David M. Ward (Montana State University). Co-PI, Donald

A. Bryant. This award provides no funding but provides 100 hours of mass spectrometry time and computing time at the Environmental Molecular Sciences Laboratory (EMSL) of Pacific Northwest National Laboratory (PNNL), a DOE national laboratory.

# Research Synopsis

I have studied chlorophototrophic bacteria for 52 years, and I have published (or submitted) ~465 papers and books since 1972, including ~150 papers over the past decade. These research products have received ~35,329 total citations (>9,125 since 2019; per Google Scholar). The *h-index* for these publications is currently 101 (101 publications with ≥101 citations; 48 since 2019) and the *i10*-*index* is 392 (*i.e.*, 392 papers with ≥10 citations) and 235 with 10 new citations since 2019. The book *“The Molecular Biology of Cyanobacteria,”* published in 1994, has received >4,780 total citations.

I am a microbial (eco)physiologist and biochemist, who has rigorously applied cutting-edge genomics, genetics, biochemistry, and molecular biology to study chlorophototrophic bacteria—predominantly *Cyanobacteria*, *Chloroflexota*, *Chlorobia*, and *Acidobacteriota*—for 52 years. My recent studies demonstrate that cyanobacteria extensively remodel their photosynthetic apparatus by synthesizing new chlorophylls, phycobiliproteins, and reaction centers in far-red light, which explains how these organisms can grow at the bottom of dense microbial mats [*Science* **345**:1312-17 (2014) and *Science* **353**:aaf9178 (2016)]. Introduction of this capability into crop plants could significantly increase their productivity by expanding the usable light for photosynthesis into the far-red range (700–800 nm). In an elegant example of using my broad expertise in solving previously intractable problems, our elucidation of (bacterio-)chlorophyll and carotenoid biosynthetic pathways directly led to structures for the BChl supramolecular structures in chlorosomes, light-harvesting complexes of green bacteria. My broad, deep knowledge of photosynthesis and metabolism is illustrated by studies of microbial mats in Yellowstone hot springs, where ~17 chlorophototrophic members from six phyla and ~325 total OTUs occur. Using systems biology/-omics approaches, my collaborators and I have made impressive progress in advancing our understanding of the structure, interdependence, and integration of members of such communities. My discovery of *Chloracidobacterium thermophilum* [*Science* **317**:523-526 (2007)], the first chlorophototrophic member of the phylum *Acidobacteriota*, is already featured in microbiology textbooks. My demonstration that cyanobacteria have a complete tricarboxylic acid cycle decisively overturned the belief, accepted for ~50 years, that the cyanobacterial TCA cycle is branched [*Science* **334**:1551-1553 (2011)] and has provided new opportunities for metabolic engineering. Finally, over the past five years, a collaboration with Dr. Chris Gisriel has led to the structures of Photosystem I from two cyanobacteria, Photosystem II (monomeric and dimeric forms), and two phycobiliprotein complexes specifically produced for harvesting and utilizing far-red light.

# Patents

1. Clonage et sequençage du gene codant pour des proteines de structure des vesicules gazeuses de

*Calothrix* sp. PCC 7601. Brevet Europeen No. 85.401.861.1 (09/24/1985).

1. PSU Invention Disclosure 2010-3692: Recombinant phycobiliproteins with enhanced fluorescence and photochemical properties including methods for production and use thereof. Inventors: R. M. Alvey, D. A. **Bryant**, A. Biswas, and W. M. Schluchter.

# Publications

1. Glazer, A. N. and **Bryant**, D. A. 1975. Allophycocyanin B (A_max_ 671, 618 nm): A new cyanobacterial phycobiliprotein. *Arch. Microbiol.* **104**, 15–22.
2. Glazer, A. N., Apell, G. S., Hixson, C. S., **Bryant**, D. A., Simon, S., and Brown, D. M. 1976. Biliproteins of cyanobacteria and Rhodophyta: A homologous family of photosynthetic accessory pigments. *Proc. Nat. Acad. Sci. USA* **73**, 428–431.
3. **Bryant**, D. A., Glazer, A. N. and Eiserling, F. A. 1976. Characterization and structural properties of the major biliproteins of *Anabaena* sp. *Arch. Microbiol.* **110**, 61–75.
4. Ley, A. C., Butler, W. L., **Bryant**, D. A., and Glazer, A. N. 1977. The isolation and function of allophycocyanin B of *Porphyridium cruentum*. *Plant Physiol.* **59**, 974–980.
5. **Bryant**, D. A. 1977. Comparative studies on cyanobacterial and rhodophytan biliproteins. Ph. D. thesis, University of California, Los Angeles, 425 pp.
6. **Bryant**, D. A., Hixson, C. S., and Glazer, A. N. 1978. Structural studies on phycobiliproteins.

III. Comparison of bilin-containing peptides from the � subunit of C-phycocyanin, R- phycocyanin, and phycoerythrocyanin. *J. Biol. Chem.* **253**, 220–225.

1. **Bryant**, D. A., Guglielmi, G., Tandeau de Marsac, N., Castets, A. M., and Cohen-Bazire, G. 1979. The structure of cyanobacterial phycobilisomes: A model. *Arch. Microbiol.* **123**, 113–127.
2. Guglielmi, G., Cohen-Bazire, G., and **Bryant**, D. A. 1981. The structure of *Gloeobacter violaceus* and its phycobilisomes. *Arch. Microbiol.* **129**, 181–189. doi: 10.1007/BF00425248
3. **Bryant**, D. A., Cohen-Bazire, G., and Glazer, A. N. 1981. Characterization of the biliproteins of *Gloeobacter violaceus*. Chromophore content of a cyanobacterial phycoerythrin carrying phycourobilin chromophore. *Arch. Microbiol.* **129**, 190–198.
4. **Bryant**, D. A. and Cohen-Bazire, G. 1981. Effects of chromatic illumination on cyanobacterial phycobilisomes. Evidence for the specific induction of a second pair of phycocyanin subunits in *Pseudanabaena* 7409 grown in red light. *Eur. J. Biochem.* **119**, 415–424.
5. **Bryant**, D. A. 1981. The photoregulated expression of multiple phycocyanin species. A general mechanism for the control of phycocyanin synthesis in chromatically adapting cyanobacteria. *Eur. J. Biochem.* **119**, 424–429.
6. **Bryant**, D. A. 1982. Phycoerythrocyanin and phycoerythrin: Properties and occurrence in cyanobacteria. *J. Gen. Microbiol.* **128**, 835–844.
7. Cohen-Bazire, G. and **Bryant**, D. A. 1982. Phycobiliproteins and phycobilisomes. In: **The Biology of the Cyanobacteria**, 2nd edition. Carr, N. G. and Whitton, B. A., eds., pp. 143–190. Blackwell Scientific Publications, Oxford, England.
8. Kipe-Nolt, J. A., Stevens, S. E., Jr., and **Bryant**, D. A. 1982. Growth and chromatic adaptation of *Nostoc* sp. Strain MAC and the pigment mutant R-MAC. *Plant Physiol.* **70**, 1549–1553.
9. de Lorimier, R., **Bryant**, D. A., Porter, R. D., Liu, W.-Y., Jay, E. and Stevens, S. E., Jr. 1984. Genes for the α and β subunits of phycocyanin. *Proc. Nat. Acad. Sci. USA* **81**, 7946–7950.
10. **Bryant**, D. A., de Lorimier, R., Porter, R. D., Lambert, D. H., Dubbs, J. M., Stirewalt, V. L., Fields, P. I., Stevens, S. E. Jr., Liu, W.-Y., Tam, J. and Jay, E. 1985. Phycobiliprotein genes in cyanobacteria and cyanelles. In: **Molecular Biology of the Photosynthetic Apparatus**. Arntzen, C., Bogorad, L., Bonitz, S., and Steinbeck, K., eds., pp. 249–258. Cold Spring Harbor Laboratory, Cold Spring Harbor, NY.
11. **Bryant**, D. A., de Lorimier, R., Lambert, D. H., Dubbs, J. M., Stirewalt, V. L., Stevens, S. E. Jr., Porter, R. D., Tam, J. and Jay, E. 1985. Molecular cloning and nucleotide sequence of the α and

β subunits of allophycocyanin from the cyanelle genome of *Cyanophora paradoxa*. *Proc. Nat. Acad. Sci. USA* **82**, 3242–3246.

1. Lambert, D. H., **Bryant**, D. A., Stirewalt, V. L., Dubbs, J. M., Stevens, S. E. Jr., and Porter, R.

D. 1985. Gene map for the *Cyanophora paradoxa* cyanelle genome. *J. Bacteriol.* **164**, 659–664.

1. **Bryant**, D. A., Porter, R. D., Fields, P. I., Dubbs, J. M., and de Lorimier, R. 1985. Expression of phycobiliprotein genes in *Escherichia coli*. *FEMS Microbiol. Lett.* **29**, 343–349.
2. Tandeau de Marsac, N., Mazel, D., **Bryant**, D. A., and Houmard, J. 1985. Molecular and nucleotide sequence of a developmentally regulated gene from the cyanobacterium *Calothrix* PCC 7601: A gas vesicle protein gene. *Nucl. Acids Res.* **13**, 7223–7236.
3. **Bryant**, D. A., de Lorimier, R., Guglielmi, G., Stirewalt, V. L., Dubbs J. M., Illman, B., Gasparich, G., Buzby, J. S., Cantrell, A., Murphy, R. C., Gingrich, J., Porter, R. D., and Stevens,

S. E. Jr. 1986. The cyanobacterial photosynthetic apparatus: A molecular genetics analysis. In: **Current** C**ommunications in Molecular Biology**, "Microbial Energy Transduction: Genetics, Structure, and Function." Youvan, D. C. and Daldal, F., eds., pp. 39–46. Cold Spring Harbor Laboratory, Cold Spring Harbor, NY.

1. Mazel, D., Guglielmi, G., Houmard, J., Sidler, W., **Bryant**, D. A., and Tandeau de Marsac, N. 1986. Green light induces transcription of the phycoerythrin operon in the cyanobacterium *Calothrix* 7601. *Nucl. Acids Res.* **14**, 8279–8290.
2. Houmard, J., Mazel, D., Moguet, C., **Bryant**, D. A., and Tandeau de Marsac, N. 1986. Organization and nucleotide sequence of gene encoding core components of the phycobilisomes from *Synechococcus* 6301. *Mol. Gen. Genet.* **205**, 404–410.
3. **Bryant**, D. A. 1987. The cyanobacterial photosynthetic apparatus: comparisons to those of higher plants and photosynthetic bacteria. In: **Photosynthetic Picoplankton**," Platt, T. and Li,

W. K. W., eds., Canadian Bulletin of Fisheries and Aquatic Sciences, Vol. 214, pp. 423–500. Department of Fisheries and Oceans, Ottawa, Ontario, Canada.

1. **Bryant**, D. A., de Lorimier, R., Guglielmi, G., Stirewalt, V. L., Cantrell, A., and Stevens, S. E. Jr. 1987. The cyanobacterial photosynthetic apparatus: a structural and functional analysis employing molecular genetics. In: **Progress in Photosynthesis Research**, Vol. 4, Biggins, J., ed., pp. 749–755. Martinus Nijhoff, Dordrecht, The Netherlands.
2. Buzby, J. S., Mumma, R. O., **Bryant**, D. A., Gingrich, J., Hamilton, R. H., Porter, R. D., Mullin,

C. A., and Stevens, S. E. Jr. 1987. Genes with mutations causing herbicide resistance from the cyanobacterium *Synechococcus* PCC 7002. In: *Progress in Photosynthesis Research*, Vol. 4, Biggins, J., ed., pp. 757–760. Martinus Nijhoff, Dordrecht, The Netherlands.

1. Gasparich, G. E., Buzby, J. S., **Bryant**, D. A., Porter, R. D., and Stevens, S. E. Jr. 1987. The effects of light intensity and nitrogen starvation on the phycocyanin promoter in the cyanobacterium *Synechococcus* PCC 7002. In: *Progress in Photosynthesis Research*, Vol. 4, Biggins, J., ed., pp. 761–764. Martinus Nijhoff, Dordrecht, The Netherlands.
2. Dubbs, J. M. and **Bryant**, D. A. 1987. Organization of the genes encoding phycoerythrin and the two differentially expressed phycocyanins in the cyanobacterium *Pseudanabaena* PCC 7409. In: *Progress in Photosynthesis Research*, Vol. 4, Biggins, J., ed., pp. 765–768. Martinus Nijhoff, Dordrecht, The Netherlands.
3. Murphy, R. C., **Bryant**, D. A., and Porter, R. D. 1987. Molecular cloning and preliminary characterization of a *recA* gene from the cyanobacterium *Synechococcus* PCC 7002. In: *Progress in Photosynthesis Research*, Vol. 4, Biggins, J., ed., pp. 769–772. Martinus Nijhoff, Dordrecht, The Netherlands.
4. Cantrell, A. and **Bryant**, D. A. 1987. Molecular cloning and nucleotide sequences of the genes encoding cytochrome *b*-559 from the cyanelle genome of *Cyanophora paradoxa*. In: *Progress in Photosynthesis Research*, Vol. 4, Biggins, J., ed., pp. 659–662. Martinus Nijhoff, Dordrecht, The Netherlands.
5. de Lorimier, R., Guglielmi, G., **Bryant**, D. A., and Stevens, S. E. Jr. 1987. Functional expression of plastid allophycocyanin genes in a cyanobacterium. *J. Bacteriol.* **169**, 1830–1835.
6. Murphy, R. C., **Bryant**, D. A., Porter, R. D., and Tandeau de Marsac, N. 1987. Molecular cloning and characterization of the *recA* gene from cyanobacterium *Synechococcus* sp. PCC 7002. *J. Bacteriol.* **169**, 2739–2747.
7. Singh, R. K., Stevens, S. E. Jr., and **Bryant**, D. A. 1987. Molecular cloning and physical mapping of the nitrogenase structural genes from the filamentous, non-heterocystous cyanobacterium *Pseudanabaena* sp. PCC 7409. *FEMS Microbiol. Lett.* **48**, 53–58.
8. Cantrell, A. and **Bryant**, D. A. 1987. Molecular cloning and nucleotide sequence of the *psaA* and

*psaB* genes of the cyanobacterium *Synechococcus* sp. PCC 7002. *Plant Mol. Biol.* **9**, 453–468.

1. Gingrich, J. C., Buzby, J. S., Stirewalt, V. L., and **Bryant**, D. A. 1988. Genetic analysis of two new mutations resulting in herbicide resistance in the cyanobacterium *Synechococcus* sp. PCC 7002. *Photosynth. Res.* **16**, 83–99.
2. Cantrell, A. and **Bryant**, D. A. 1988. Nucleotide sequence of the genes encoding cytochrome *b*- 559 from the cyanelle genome of *Cyanophora paradoxa*. *Photosynth. Res.* **16**, 65–81.
3. **Bryant**, D. A. 1988. Phycobilisomes of *Synechococcus* sp. PCC 7002, *Pseudanabaena* sp. PCC 7409, and *Cyanophora paradoxa*: An analysis by molecular genetics. In: **Photosynthetic Light- harvesting Systems--Structure and Function**, Scheer, H. and Schneider, S., eds., pp. 217–232.

W. de Gruyter & Co., Berlin.

1. **Bryant**, D. A. and Tandeau de Marsac, N. 1988. Isolation of genes encoding components of the photosynthetic apparatus. In: *Methods Enzymol.* **167**, 755–765.
2. Shively, J. M., **Bryant**, D. A., Fuller, R. C., Konopka, A. E., Stevens, S. E. Jr., and Strohl, W. R. 1988. Inclusions of Procaryotes. *Int. J. Cytol.* **113**, 35–100.
3. Arciero, D. M., **Bryant**, D. A., and Glazer, A. N. 1988. *In vitro* attachment of bilins to apophycocyanin. I. Specific covalent adduct formation at cysteinyl residues involved in phycocyanobilin binding in C-phycocyanin. *J. Biol. Chem.* **263**, 18343–18349.
4. Govindjee, Bohnert, H. J., Bottomley, W., **Bryant**, D. A., Mullet, J. E., Ogren, W. L., Pakrasi, H., and Somerville, C. R. 1988. ***Molecular Biology of Photosynthesis***. Kluwer Academic Publishers, Dordrecht, The Netherlands. 815 pp.
5. Stevens, S. E. and **Bryant**, D. A. 1988. ***Light-Energy Transduction in Photosynthesis: Higher Plant and Bacterial Models****.* American Society for Plant Physiologists, Rockville, MD. 388 pp.
6. **Bryant**, D. A. 1988. Cyanobacterial phycobilisomes: Structure, function, and assembly as analyzed by molecular genetics. In: **Light-Energy Transduction in Photosynthesis: Higher Plant and Bacterial Models**, Stevens, S. E. Jr. and Bryant, D. A., eds., pp. 62–90. American Society for Plant Physiologists, Rockville, MD.
7. Rhiel, E. and **Bryant**, D. A. 1988. Preliminary results concerning the *psaC*, *psaD*, *psaE*, and *psaF* genes and their products in the cyanobacteria *Synechococcus* sp. PCC 7002 and *Nostoc* sp. PCC 8009. In: **Light-Energy Transduction in Photosynthesis: Higher Plant and Bacterial Models**, Stevens, S. E. Jr. and Bryant, D. A., eds., pp. 320–323. American Society for Plant Physiologists, Rockville, MD.
8. Gasparich, G. E. and **Bryant**, D. A. 1988. Regulation of phycocyanin expression in *Synechococcus* sp. PCC 7002. In: **Light-Energy Transduction in Photosynthesis: Higher Plant and Bacterial Models**, Stevens, S. E. Jr. and Bryant, D. A., eds., pp. 337–339. American Society for Plant Physiologists, Rockville, MD.
9. Zhou, J., Stirewalt, V. L., and **Bryant**, D. A. 1988. Molecular cloning, nucleotide sequencing and mutagenesis of the *apcD* gene of *Synechococcus* sp. PCC 7002. In: **Light-Energy Transduction in Photosynthesis: Higher Plant and Bacterial Models**, Stevens, S. E. Jr. and Bryant, D. A., eds., pp. 340–343. American Society for Plant Physiologists, Rockville, MD.
10. Dubbs, J. M. and **Bryant**, D. A. 1988. Genes for the phycobilisome rod components of the chromatically adapting cyanobacterium *Pseudanabaena* sp. PCC 7409. In: **Light-Energy Transduction in Photosynthesis: Higher Plant and Bacterial Models**, Stevens, S. E. Jr. and Bryant, D. A., eds., pp. 344–346. American Society for Plant Physiologists, Rockville, MD.
11. Bruce, D., Brimble, S., and **Bryant**, D. A. 1989. State transitions in a phycobilisome-less mutant of the cyanobacterium *Synechococcus* sp. PCC 7002. *Biochim. Biophys. Acta* **974**, 66–73.
12. Maxson, P., Sauer, K., **Bryant**, D. A., and Glazer, A. N. 1989. Spectroscopic studies of cyanobacterial phycobilisomes lacking core polypeptides. *Biochim. Biophys. Acta* **974**, 40–51.
13. **Bryant**, D. A., Rhiel, E., de Lorimier, R., Zhou, J., Gasparich, G. E., Dubbs, J. M., Stirewalt, V. L., and Snyder, W. 1989. Characterization of cyanobacterial phycobilisomes and Photosystem I complexes. **Current Research in Photosynthesis**, Vol. II. Baltscheffsky, M., ed., pp. 1–11. Kluwer Academic Publishers, Dordrecht.
14. **Bryant**, D. A., Zhou, J., Gasparich, G. E., de Lorimier, R., Guglielmi, G., and Stirewalt, V. L. 1989. Phycobilisomes of the cyanobacterium *Synechococcus* sp. PCC 7002: Structure function, assembly, and expression. In: **Molecular Biology of Membrane-bound Complexes in Phototrophic Bacteria**, Drews, G., ed., pp. 129–141. Plenum, Berlin.
15. Stirewalt, V. L., and **Bryant**, D. A. 1989. Nucleotide sequence of the *petG* gene of the cyanelle genome of *Cyanophora paradoxa*. *Nucl. Acids Res.* **17**, 10095.
16. Stirewalt, V. L., and **Bryant**, D. A. 1989. Nucleotide sequence of the *psbK* gene of the cyanelle genome of *Cyanophora paradoxa*. *Nucl. Acids Res.* **17**, 10096.
17. **Bryant**, D. A. and Stirewalt, V. L. 1990. The cyanelle genome of *Cyanophora paradoxa* encodes ribosomal proteins not encoded by the chloroplast genomes of higher plants. *FEBS Lett.* **259**, 273–280.
18. Murphy, R. C., Gasparich, G. E., **Bryant**, D. A., and Porter, R. D. 1990. Nucleotide sequence and further characterization of the *Synechococcus* sp. PCC 7002 *recA* gene: complementation of a cyanobacterial *recA* mutation by the *E. coli recA* gene. *J. Bacteriol.* **172**, 967–976.
19. **Bryant**, D. A., de Lorimier, R., Guglielmi, G., and Stevens, S. E. Jr. 1990. Structural and compositional analyses of the phycobilisomes of *Synechococcus* sp. PCC 7002. Analyses of the wild-type strain and a phycocyanin-less mutant constructed by interposon mutagenesis. *Arch. Microbiol.* **153**, 550–560.
20. de Lorimier, R., Guglielmi, G., **Bryant**, D. A., and Stevens, S. E. Jr. 1990. Structure and mutation of a gene encoding a 33 kDa phycocyanin-associated linker polypeptide. *Arch*. *Microbiol*. **153**, 541–549.
21. Gingrich, J. C., Gasparich, G. E., Sauer, K., and **Bryant**, D. A. 1990. Nucleotide sequence and expression of the two genes encoding the D2 protein and the single gene encoding CP43 protein of Photosystem II in the cyanobacterium *Synechococcus* sp. PCC 7002. *Photosynth. Res.* **24**, 137–150.
22. de Lorimier R., **Bryant**, D. A., and Stevens, S. E. Jr. 1990. Genetic analysis of a 9 kDa phycocyanin-associated linker polypeptide. *Biochim. Biophys. Acta* **1019**, 29–41.
23. Zhao, J., **Warre**n, P. V., Li, N., **Bryant**, D. A., Golbeck, J. H. 1990. Reconstitution of electron transport in Photosystem I with PsaC and PsaD proteins expressed in *Escherichia coli*. *FEBS Lett*. **276**, 175–180.
24. **Bryant**, D. A. 1991. Cyanobacterial phycobilisomes: Progress towards a complete structural and functional analysis via molecular genetics. In: *Cell Culture and Somatic Cell Genetics of Plants*, Volume 7B: *The Photosynthetic Apparatus: Moleular Biology and Operation* (Bogorad, L. and Vasil, I. K., eds.), pp. 257–300. Academic Press, New York, New York.
25. Golbeck J. H. and **Bryant**, D. A. 1991. Photosystem I. In: *Current Topics in Bioenergetics: Light-Driven Reactions in Bioenergetics* (Lee C. P., ed.), pp. 83–177. Academic Press, New York, New York.
26. **Bryant**, D. A., Schluchter, W. M. and Stirewalt, V. L. 1991. Ferredoxin and ribosomal protein S10 are encoded on the cyanelle genome of *Cyanophora paradoxa*. *Gene* **98**, 169–175.
27. Li, N., Warren, P. V., Golbeck, J. H., Frank, G., Zuber, H., and **Bryant**, D. A. 1991. Polypeptide composition of the photosystem I complex and the photosystem I core protein from *Synechococcus* sp. PCC 6301. *Biochim. Biophys. Acta* **1059**, 215–225.
28. Li, N., Zhao, J., Warren, P. V., Warden, J. T., **Bryant**, D. A., and Golbeck, J. H. 1991. PsaD is required for the stable binding of PsaC to the Photosystem I core protein of *Synechococcus* sp. PCC 6301. *Biochemistry* **30**, 7863–7872.
29. **Bryant**, D. A., Stirewalt, V. L., Glauser, M., Frank, G., Sidler, W., and Zuber, H. 1991. A small multigene family encodes the rod-core linker polypeptides of *Anabaena* sp. PCC 7120 phycobilisomes. *Gene* **107**, 91–99.
30. Dubbs, J. M. and **Bryant**, D. A. 1991. Molecular cloning and transcriptional analysis of the *cpeBA* operon of the cyanobacterium *Pseudanabaena* sp. PCC 7409. *Mol. Microbiol.* **5**, 3073- 3085.
31. **Bryant**, D. A. 1992. Molecular Biology of Photosystem I. In: **Topics in Photosynthesis** (Barber, J., ed.), Vol. 11 (**The Photosystems: Structure, Function and Molecular Biology**), pp. 501–

549. Elsevier, Amsterdam, The Netherlands.

1. Lockhart, P. J., Howe, C. J., **Bryant**, D. A., Beanland, T. J., and Larkum, A. W. D. 1992. Substitutional bias confounds inference of cyanelle origins from sequence data. *J. Mol. Evol.* **34**, 153–162.
2. Rhiel, E., Stirewalt, V. L., Gasparich, G. E. and **Bryant**, D. A. 1992. The *psaC* genes of *Synechococcus* sp. PCC 7002 and the cyanelle genome of *Cyanophora paradoxa*. *Gene* **112**, 123–128.
3. Glauser, M., Sidler, W., Graham, K., **Bryant**, D. A., Frank, G., Wehrli, E., and Zuber, H. 1992. Three C-phycoerythrin associated linker polypeptides in the phycobilisomes of the cyanobacterium *Calothrix* sp. PCC 7601 grown in green light. *FEBS Lett.* **297**, 19–23.
4. Schluchter, W. M. and **Bryant**, D. A. 1992. Molecular characterization of ferredoxin-NADP+ reductase in cyanobacteria: cloning and sequence of the *petH* gene of *Synechococcus* sp. PCC 7002 and studies on the gene product. *Biochemistry* **31,** 3092–3102.
5. Glauser, M., **Bryant**, D. A., Frank, G., Wehrli, E., Sidler, W., and Zuber, H. 1992. Phycobilisome structure in the cyanobacteria *Mastigocladus laminosus* and *Anabaena* sp. PCC 7120: a new model. *Eur. J. Biochem*. **205**, 907–915.
6. Glauser, M., Stirewalt, V. L., **Bryant**, D. A., Sidler, W., and Zuber, H. 1992. Structure of the genes encoding the rod-core linker polypeptides of *Mastigocladus laminosus* phycobilisomes and functional aspects of the phycobiliprotein-linker polypeptide interactions. *Eur. J. Biochem.* **205**, 927–937.
7. **Bryant**, D. A. 1992. Puzzles of chloroplast ancestry. *Curr. Biol.* **2**, 240–242.
8. Zhao, J., Li, N., Warren, P. V., Golbeck, J. H., and **Bryant**, D. A. 1992. Site-directed conversion of a cysteine to aspartate leads to the assembly of a [3Fe-4S] cluster in PsaC of Photosystem I. The photoreduction of F_A_ is independent of F_B_ at low temperature. *Biochemistry* **31**, 5093–5099.
9. Zhou, J., Gasparich, G. E., Stirewalt, V. L., de Lorimier, R., and **Bryant**, D. A. 1992. The *cpcE* and *cpcF* genes of *Synechococcus* sp. PCC 7002: Construction and phenotypic characterization of interposon mutants. *J. Biol. Chem.* **267**, 16138–16145.
10. Swanson, R. V., Zhou, J., de Lorimier, R., Leary, J. A., Williams, T., **Bryant**, D. A., and Glazer,
    1. N. 1992. Characterization of phycocyanin produced by *cpcE* and *cpcF* mutants and identification of an intergenic suppressor of the defect in bilin attachment. *J. Biol. Chem.* **267**, 16146–16154.
11. Fairchild, C. D., Zhao, J., Zhou, J., Colson, S. E., **Bryant**, D. A., and Glazer, A. N. 1992. Phycocyanin a subunit phycocyanobilin lyase. *Proc. Natl. Acad. Sci. USA* **89**, 7017–7021.
12. Gindt, Y. M., Zhou, J., **Bryant**, D. A., and Sauer, K. 1992. Core mutations of *Synechococcus* sp. PCC 7002 phycobilisomes: a spectroscopic study. *J. Photochem. Photobiol. B: Biol.* **15**, 75–89.
13. Chung, S. and **Bryant**, D. A. 1992. Genes encoding chlorosome components in the green sulfur bacteria *Chlorobium vibrioforme* 8327D and *Chlorobium tepidum*. In: **Research in Photosynthesis** (Murata, N., ed.), Kluwer, Dordrecht, The Netherlands, pp. 69–72.
14. Zhao, J., Zhou, J., and **Bryant**, D. A. 1992. Energy transfer processes in phycobilisomes as deduced from analyses of mutants of *Synechococcus* sp. PCC 7002. In: **Research in Photosynthesis, Vol. I** (Murata, N., ed.), Kluwer, Dordrecht, The Netherland, pp. 25–32.
15. Yu, L., Golbeck, J. H., Zhao, J., Schluchter, W. M., Mühlenhoff, U., and **Bryant**, D. A. 1992. The PsaE protein is required for cyclic electron flow around Photosystem I in *Synechococcus* sp. PCC 7002. In: **Research in Photosynthesis, Vol. I** (Murata, N., ed.), Kluwer, Dordrecht, The Netherlands, pp. 565–568.
16. Rhiel, E. and **Bryant**, D. A. 1993. Nucleotide sequence of the *psaE* gene of *Synechococcus* sp. PCC 6301. *Plant Physiol.* **101**, 701–702.
17. Dubbs, J. M. and **Bryant**, D. A. 1993. Organization and transcription of the genes encoding two differentially expressed phycocyanins in the cyanobacterium *Pseudanabaena* sp. PCC 7409. *Photosynth. Res.* **36**, 169–183.
18. Schluchter, W. M., Zhao, J. and **Bryant**, D. A. 1993. Isolation and characterization of the *ndhF* gene of *Synechococcus* sp. PCC 7002 and initial characterization of an interposon mutant. *J. Bacteriol.* **175**, 3343–3352.
19. Zhao, J., Snyder, W. B., Mühlenhoff, U., Rhiel, E., Warren, P. V., Golbeck, J. H. and **Bryant,** D.
    1. 1993. Cloning and characterization of the *psaE* gene of the cyanobacterium *Synechococcus* sp. PCC 7002: Overproduction of the protein in *Escherichia coli* and characterization of a *psaE* mutant. *Mol. Microbiol.* **9**, 183–194.
20. Yu, L., Zhao, J., Mühlenhoff, U., Chitnis, P. R., **Bryant**, D. A., and Golbeck, J. H. 1993. PsaE is required for cyclic electron flow around photosystem I in the cyanobacterium *Synechococcus* sp. PCC 7002 and *Synechocystis* sp. PCC 6803. *Plant Physiol.* **103**, 171–180.
21. Yu, L., Zhao, J., Liu, W., **Bryant**, D. A., and Golbeck, J. H. 1993. Characterization of the [3Fe- 4S] and [4Fe-4S] clusters in unbound PsaC mutants C14D and C51D. The midpoint potentials of the single [4Fe-4S] clusters are identical to F_A_ and F_B_ in bound PsaC of photosystem I. *Biochemistry* **32**, 8251–8258.
22. Debreczeny, M. P., Sauer, K., Zhou, J., and **Bryant**, D. A. 1993. Monomeric C-phycocyanin at room temperature and 77 K: resolution of the absorption and fluorescence spectra of the individual chromophores and the energy transfer rate constants. *J. Phys. Chem.* **97**, 9852–9862.
23. Chung, S., Frank, G., Zuber, H. and **Bryant**, D. A. 1994. Genes encoding two chlorosome proteins from the green sulfur bacteria *Chlorobium vibrioforme* strain 8327D and *Chlorobium tepidum*. *Photosynth. Res.* **41**, 261–275.
24. **Bryant**, D. A. 1994. Gene nomenclature recommendations for green photosynthetic bacteria and heliobacteria. *Photosynth. Res.* **41**, 27–28.
25. Falzone, C. J., Kao, Y.-H., Zhao, J., MacLaughlin, K. L., and **Bryant**, D. A., and Lecomte, J. T.

J. 1994. 1H and 15N NMR assignments of the PsaE, a Photosystem I subunit from the cyanobacterium *Synechococcus* sp. strain PCC 7002. *Biochemistry* **33**, 6043–6051.

1. Falzone, C. J., Kao, Y.-H., Zhao, J., **Bryant**, D. A., and Lecomte, J. T. J. 1994. The three- dimensional solution structure of PsaE from the cyanobacterium *Synechococcus* sp. strain PCC 7002: A photosystem I protein structurally homologous with SH3 domains. *Biochemistry* **33**, 6052–6062.
2. Gindt, Y. M., Zhou, J., **Bryant**, D. A., and Sauer, K. 1994. Spectroscopic studies of phycobilisome subcore preparations lacking key core chromophores: assignment of excited state energies to the L_CM_, �18, and aAP-B chromophores. *Biochim. Biophys. Acta* **1186**, 153–162.
3. **Bryant**, D. A. 1994. ***The Molecular Biology of Cyanobacteria****, Advances in Photosynthesis and Respiration*, Volume 1, 908 pp. Kluwer Academic Publishers, Dordrecht, The Netherlands.
4. Shen, G. and **Bryant**, D. A. 1995. Characterization of a *Synechococcus* sp. strain PCC 7002 mutant lacking Photosystem I. Protein assembly and energy distribution in the absence of the photosystem I reaction center core complex. *Photosynth. Res.* **44,** 41–53.
5. Debreczeny, M. P., Sauer, K., Zhou, J., and **Bryant**, D. A. 1995. Comparison of calculated and experimentally resolved rate constants for excitation energy transfer in C-phycocyanin. Part I: Monomers. J. Phys. Chem. **99**, 8412–8419. https://doi.org/10.1021/j100020a080
6. Debreczeny, M. P., Sauer, K., Zhou, J., and **Bryant**, D. A. 1995. Comparison of calculated and experimentally resolved rate constants for excitation energy transfer in C-phycocyanin. Part II: Trimers. *J. Phys. Chem.* **99**: 8420–8431. https://doi.org/10.1021/j100020a081
7. Yu, L., **Bryant**, D. A. and Golbeck, J. H. 1995. Identification of a mixed ligand [4Fe-4S] cluster in the C14D mutant of PsaC on rebinding to the P700-FX core. Altered reduction potentials and EPR spectral properties of both the FA and FB clusters. *Biochemistry* **34**, 7861–7868.
8. Mehari, T., Qiao, F., Scott, M. P., Nellis, D. F., Zhao, J., **Bryant**, D. A., and Golbeck, J. H. 1995. Modified ligands to FA and FB in Photosystem I: I. Structural constraints for the formation of iron-sulfur clusters in free and rebound PsaC. *J. Biol. Chem.* **270**, 28108–28117.
9. Yu, L., Vassiliev, I. R., Jung, Y.-S., **Bryant**, D. A. and Golbeck, J. H. 1995. Modified ligands to FA and FB in Photosystem I: II. Identification of a mixed-ligand [4Fe-4S] cluster in the C51D mutant of PsaC after rebinding to the P700-FX core. *J. Biol. Chem.* **270**, 28118–28125.
10. Chung, S., Jakobs, C. U., Ormerod, J. G., and **Bryant**, D. A. 1995. Protein components of chlorosomes from *Chlorobium tepidum* and interposon mutagenesis of *csmA* and *csmC* from *Chlorobium vibrioforme* 8327D. **Photosynthesis: from Light to Biosphere** (P. Mathis, ed.), Vol. I, pp. 11–16. Kluwer, Dordrecht, The Netherlands.
11. Jung, Y.-S., J. Yu, Yu, L., Zhao, J., **Bryant**, D. A., McIntosh, L., and Golbeck, J. H. 1995. *In vivo* site-directed mutations of the cysteine ligands to FA and FB in *Synechocystis* sp. PCC 6803: a comparison with *in vitro* reconstituted photosystem I complexes. **Photosynthesis: from Light to Biosphere** (P. Mathis, ed.), Vol. II, pp. 127–130. Kluwer, Dordrecht, The Netherlands.
12. Xia, Z., Broadhurst, R. W., Laue, E. D., **Bryant**, D. A., Golbeck, J. H., and Bendall, D. S. 1995. Spectroscopic evidence for the flexibility of PsaD in solution. **Photosynthesis: from Light to Biosphere** (P. Mathis, ed.), Vol. II, pp. 741–744. Kluwer, Dordrecht, The Netherlands.
13. Stirewalt, V. L., Michalowski, C. B., Löffelhardt, W., Bohnert, H. J. and **Bryant**, D. A. 1995. Nucleotide sequence of the cyanelle DNA from *Cyanophora paradoxa*. *Plant Mol. Biol. Reporter* **13**, 327–332.
14. Shevelev, E., D. A. **Bryant**, W. Löffelhardt, and H. J. Bohnert 1995. Ribonuclease-P RNA gene of the plastid chromosome from *Cyanophora paradoxa*. *DNA Res.* **2**, 231–234.
15. Mühlenhoff, U., Kruip, J., Nitschke, W., **Bryant**, D. A., Rögner, M., Sétif, P., and Boekema, E. 1996. Characterization of a redox active cross-linking complex between cyanobacterial photosystem I and its physiological acceptor flavodoxin. *EMBO J.* **15**, 488–497.
16. Mühlenhoff, U., Zhao, J., and **Bryant**, D. A. 1996. Interaction of Photosystem I and flavodoxin from the cyanobacterium *Synechococcus* sp. PCC 7002 as revealed by chemical cross-linking. *Eur. J. Biochem.* **325**, 324–331.
17. Caslake, L. and **Bryant**, D. A. 1996. Cloning and characterization of the *sigA* gene encoding the major sigma factor of RNA polymerase from the marine cyanobacterium *Synechococcus* sp. PCC 7002. *Microbiology* **142**, 347–357.
18. Schluchter, W. M., Shen, G., Zhao, J. and **Bryant**, D. A. 1996. Characterization of *psaI* and *psaL* mutants of *Synechococcus* sp. PCC 7002: a new model for state transitions in cyanobacteria. *Photochem. Photobiol.* **64**, 53–66.
19. Chung, S. and **Bryant**, D. A. 1996. Characterization of *csmB* genes from *Chlorobium vibrioforme* 8327D and *Chlorobium tepidum* and overproduction of the *Chlorobium tepidum* CsmB protein in *Escherichia coli*. *Arch. Microbiol.* **166**, 23422–22244.
20. Sültemeyer, D., G. D. Price, **Bryant**, D. A. and Badger, M. R. 1996. PsaE- and NdhF-mediated electron transport affect bicarbonate transport rather than carbon dioxide uptake in the cyanobacterium *Synechococcus* sp. PCC 7002. *Planta* **201**, 36–42.
21. Jung, Y.-S., Vassiliev, I. R., Qiao, F., Yang, F., **Bryant**, D. A. and Golbeck, J. H. 1996. Modified ligands to FA and FB in Photosystem I. III. Chemical rescue of a [4Fe-4S] cluster using an external thiolate in alanine, glycine, and serine mutants of PsaC. *J. Biol. Chem.* **271**, 31135– 31144.
22. Chung, S. and **Bryant**, D. A. 1996. Characterization of the *csmD* and *csmE* genes from *Chlorobium tepidum*. The CsmA, CsmC, CsmD, and CsmE proteins are components of the chlorosome envelope. *Photosynth. Res.* **50**, 41–59.
23. Löffelhardt, W., Stirewalt, V. L., Michalowski, C. B., Annarella, M., Farley, J. Y., Schluchter,

W. M., Chung, S., Newmann-Spallart, C., Steiner, J. M., Jakowitsch, J., Bohnert, H. J., and **Bryant**, D. A. 1997. The complete sequence of the cyanelle genome of *Cyanophora paradoxa*: the genetic complexity of a primitive plastid. In: Eukaryotism and Symbiosis (Schenk, H. E. A., Herrmann, R., Jeon, K. W., Müller, N. E. and Schwemmler, W., eds.), pp. 40–48. Springer, Heidelberg, Germany.

1. Sakamoto, T., Stirewalt, V. L., and **Bryant**, D. A. 1997. Two acyl-lipid �9 desaturase genes of the cyanobacterium *Synechococcus* sp. PCC 7002. In: Williams, J. P., Khan, M. U., and Lem, N.

W. (eds.) Physiology, Biochemistry, and Molecular Biology of Plant Lipids, pp. 380–382. Kluwer, Dordrecht, The Netherlands.

1. Löffelhardt, W., Bohnert, W. J. and **Bryant**, D. A. 1997. The cyanelles of *Cyanophora paradoxa*. *Crit. Rev. Plant Physiol.* **16**, 393–413.
2. Gruber, T. M. and **Bryant**, D. A. 1997. Molecular systematic studies of eubacteria using a70-type sigma factors of Group 1 and Group 2. *J. Bacteriol.* **179**, 1734–1747.
3. Sakamoto, T. and **Bryant**, D. A. 1997. Temperature-regulated mRNA accumulation and stabilization for fatty acid desaturase genes in the cyanobacterium *Synechococcus* sp. strain PCC 7002. *Mol. Microbiol.* **23**, 1281–1292.
4. Löffelhardt, W., Bohnert, H. J., and **Bryant**, D. A. 1997. The complete sequence of the *Cyanophora paradoxa* cyanelle genome. In: *Origins of the Algae and Their Plastids*, D. Bhattacharya, ed. *Pl. Syst. Evol.* [suppl.] **11**, 149–162.
5. Sakamoto, T., Higashi, S., Wada, H., Murata, N., and **Bryant**, D. A. 1997. Low-temperature induced desaturation of fatty acids and expression of desaturase genes in the cyanobacterium *Synechococcus* sp. strain PCC 702. *FEMS Microbiol. Lett.* **52**, 313–320.
6. Caslake, L., Gruber, T. M. and **Bryant**, D. A. 1997. Expression of two alternative sigma factors of *Synechococcus* sp. PCC 7002 is modulated by carbon and nitrogen stress. *Microbiology* **143**, 3807–3818.
7. Sakamoto, T. and **Bryant**, D. A. 1998. Growth at low temperature causes nitrogen limitation in the cyanobacterium *Synechococcus* sp. PCC 7002. *Arch. Microbiol.* **169**, 10–19.
8. Sakamoto, T., Shen, G., Higashi, S., Murata, N., and **Bryant**, D. A. 1998. Alteration of low- temperature susceptibility of the cyanobacterium *Synechococcus* sp. PCC 7002 by genetic manipulation of membrane lipid unsaturation. *Arch. Microbiol.* **169**, 20–28.
9. Gruber, T. M. and **Bryant**, D. A. 1998. Characterization of the alternative sigma factors SigD and SigE in *Synechococcus* sp. strain PCC 7002. SigE is implicated in transcription of post- exponential-phase-specific genes. *Arch. Microbiol.* **169**, 211–219.
10. Gruber, T. M., Eisen, J. A., Gish, K. and **Bryant**, D. A. 1998. The phylogenetic relationships of *Chlorobium tepidum* and *Chloroflexus aurantiacus* based upon their RecA sequences. *FEMS Microbiol. Lett.* **162**, 53–60.
11. Sakamoto, T. Delgaizo, V. B. and **Bryant**, D. A. 1998. Growth on urea can trigger peroxidative death of the cyanobacterium *Synechococcus* sp. strain PCC 7002. *Appl. Environ. Microbiol.* **64**, 2361–2366.
12. Xia, Z., Broadhurst, R. W., Laue, E. D., **Bryant**, D. A., Golbeck, J. H., and Bendall, D. S. 1998. Structure and properties of PsaD in solution. *Eur. J. Biochem.* **255**, 309–316.
13. Chung, S., Shen, G., Ormerod, J. G. and **Bryant**, D. A. 1998. Insertional inactivation studies of the *csmA* and *csmC* genes of the green sulfur bacterium *Chlorobium vibrioforme* 8327: the chlorosome protein CsmA is required for viability, but CsmC is dispensable. *FEMS Microbiol. Lett.* **164**, 353–361.
14. Yang, F., Shen, G., Schluchter, W. M., Zybailov, B., Ganago, A., **Bryant**, D. A. and Golbeck, J.

H. 1998. Deletion of the PsaF polypeptide modifies the environment of the redox-active phylloquinone (A1). Evidence for unidirectionality of electron transfer in photosystem I. *J. Phys. Chem.* **102**, 8288–8299.

1. Zhao, J., Li, R. and **Bryant**, D. A. 1998. Measurement of Photosystem I activity by photoreduction of recombinant flavodoxin. *Analyt. Biochem.* **264**, 263–270.
2. Gruber, T. M. and **Bryant**, D. A. 1998. Characterization of the group 1 and group 2 sigma factors of the green sulfur bacterium *Chlorobium tepidum* and the green gliding bacterium *Chloroflexus aurantiacus*. *Arch. Microbiol.* **170**, 285–296.
3. Shen, G., Antonkine, M. L., Vassiliev, I. R., Golbeck, J. H. and **Bryant**, D. A. 1998. A rubredoxin-like protein plays an essential role in assembly of the FA, FB and FX iron-sulfur clusters in photosystem I. In: Photosynthesis: Mechanisms and Effects (G. Garab, ed.), Vol. IV,

pp. 3147–3150. Kluwer, Dordrecht, The Netherlands.

1. Vassilieva, E. V. and **Bryant**, D. A. 1998. Selective extraction of proteins from chlorosomes of *Chlorobium tepidum*. In: Photosynthesis: Mechanisms and Effects (G. Garab, ed.), Vol. I, pp. 105–108. Kluwer, Dordrecht, The Netherlands.
2. Zybailov, B. L., Shen, G., Vassiliev, I. R., **Bryant**, D. A., Reategui, R., Johnson, W., Xu, W., Chitnis, P. R. and Golbeck, J. H. 1998. Mutations in the phylloquinone biosynthetic pathway: a foreign quinone is recruited into the A1 binding site after interruption of the *menA* and *menB* genes in *Synechocystis* sp. PCC 6803. In: Photosynthesis: Mechanisms and Effects (G. Garab, ed.), Vol. I, pp. 647–650. Kluwer, Dordrecht, The Netherlands.
3. Gruber, T. M. and **Bryant**, D. A. 1999. Tracing the relationships among the eubacteria using a70- type sigma factors. In: *Enigmatic Microorganisms and Life in Extreme Environments*, Seckbach,

J. (ed.), pp. 3–13. Kluwer, Dordrecht, The Netherlands.

1. Nomura, C. and **Bryant**, D. A. 1999. Cytochrome *c*6 from *Synechococcus* sp. PCC 7002. In: Peschek, G. A., Löffelhardt, W. and Schmetterer, G. (eds.), The Phototrophic Prokaryotes, pp. 269–274. Plenum, Vienna, Austria.
2. Gruber, T. M. and **Bryant**, D. A. 1999. An overview of sigma factors of RNA polymerase in phototrophic bacteria. In: Peschek, G. A., Löffelhardt, W. and Schmetterer, G. (eds.), The Phototrophic Prokaryotes, pp. 791–798. Plenum, Vienna, Austria.
3. Yang, F., Shen, G., Schluchter, W. M., Zybailov, B., Ganago, A., Golbeck, J. H. and **Bryant**, D.
   1. 1999. Structural and functional analyses of cyanobacterial photosystem I: the directionality of electron transfer." In: Peschek, G. A., Löffelhardt, W. and Schmetterer, G. (eds.), The Phototrophic Prokaryotes, pp. 21–33. Plenum, Vienna, Austria.
4. Sakamoto, T. and **Bryant**, D. A. 1999. Nitrate transport and not photoinhibition is the rate- limiting step for growth of the freshwater cyanobacterium *Synechococcus* sp. PCC 6301 at low temperature. *Plant Physiol.* **119**, 785–794.
5. Mayer, K. L., Shen, G., **Bryant**, D. A., J. T. J. Lecomte, and Falzone, C. J. 1999. The solution structure of Photosystem I accessory protein E from the cyanobacterium *Nostoc* sp. strain PCC 8009. *Biochemistry* **38**, 13736–13746.
6. Sakamoto, T. and **Bryant**, D. A. 1999. A novel nitrate/nitrite permease in the marine cyanobacterium *Synechococcus* sp. strain PCC 7002. *J. Bacteriol.* **181**, 7363–7372.
7. Johnson, W., Shen, G., Zybailov, B., Kolling, D., Reategui, R., Beauparlant, S., Vassiliev, I. R., **Bryant**, D. A., Jones, A. D., Golbeck, J. H., and Chitnis, P. 2000. Mutagenesis of the *menA* and *menB* genes in the biosynthetic pathway to phylloquinone in *Synechocystis* sp. PCC 6803. I. Recruitment of a foreign quinone into the A_1_ site of photosystem I. *J. Biol. Chem.* **275**, 8523– 8530.
8. Antonkine, M. L., Bentrop, D., Bertini, I., Luchinat, C., Shen, G., **Bryant**, D. A., Stehlik, D. and Golbeck, J. H. 2000. Paramagnetic 1H NMR spectroscopy of the reduced unbound Photosystem I subunit PsaC: sequence specific assignment of contact shifted resonances and identification of mixed and equal valence Fe-Fe pairs in [4Fe-4S] centers FA- and FB-. *J. Bioinorg. Chem.* **5**, 381– 392.
9. Bhaya, D., Bianco, N. R., **Bryant**, D. A., and Grossman, A. R. 2000. Type IV pilus biogenesis and motility in the cyanobacterium *Synechocystis* sp. PCC 6803. *Mol. Microbiol.* **37**, 941–951.
10. Vassilieva, E. V., Frigaard, N.-U. and **Bryant**, D. A. 2000. Chlorosomes: the light-harvesting complexes of the green bacteria. *The Spectrum* **13**, 7–13.
11. Vassilieva, E. V., Antonkine, M. L., Zybailov, B., Yang, F., Golbeck, J. H. and **Bryant**, D. A. 2001. Electron transport may occur in the chlorosome envelope: the CsmI and CsmJ proteins of chlorosomes are 2Fe-2S ferredoxins. *Biochemistry* **40**, 464–473.
12. Frigaard, N.-U. and **Bryant**, D. A. 2001. Chromosomal gene inactivation in the green sulfur bacterium *Chlorobium tepidum* by natural transformation. *Appl. Environ. Microbiol.* **67**, 2538– 2544.
13. Zhao, J., Shen, G. and **Bryant**, D. A. 2001. Photosystem stoichiometry and state transitions in a mutant of the cyanobacterium *Synechococcus* sp. PCC 7002 lacking phycocyanin. *Biochim. Biophys. Acta.* **1505**, 248–257.
14. Bertini, I., **Bryant**, D. A., Ciurli, S., Dikiy, A., Fernández, C. O., Luchinat, C., Safarov, N., Vila,
    1. J., and Zhao, J. 2001. Backbone dynamics of plastocyanin in both oxidation states. Solution structure of the reduced form and comparison with the oxidized state. *J. Biol. Chem.* **276**, 47217– 47226.
15. Sakamoto, T. and **Bryant**, D. A. 2001. Requirement of nickel as an essential micronutrient for the utilization of urea in the marine cyanobacterium *Synechococcus* sp. PCC 7002. *Microbes Environ.* **16**, 177–184.
16. Schluchter, W. M. and **Bryant**, D. A. 2002. Analysis and reconstitution of phycobiliproteins: methods for the characterization of bilin attachment reactions. In: (Witty, M and Smith, A., eds.) Analytical Methods for Chlorophyll, Heme, and Related Molecules. Humana Press, Totowa, New Jersey, USA. pp. 311–334.
17. Vassilieva, E. V., Ormerod, J. G., and **Bryant**, D. A. 2002. Biosynthesis of chlorosome proteins is not inhibited in acetylene-treated cultures of *Chlorobium vibrioforme*. *Photosynth. Res.* **71**, 69–81.
18. Frigaard, N.-U., Vassilieva, E. V., Li, H., Milks, K. J., Zhao, J. and **Bryant**, D. A. 2002. The remarkable chlorosome. PS2001 Proceedings, Proceedings of the 12^th^ International Congress on Photosynthesis, Brisbane, Australia. Article S1–003, 6 pp. CSIRO Publishing, Canberra, Australia.
19. Antonkine, M. L., Liu, G., Bentrop, D., Bertini, I., Luchinat, C., **Bryant**, D. A., Golbeck, J. H. and Stehlik, D. 2002. Solution structure of the unbound, oxidized photosystem I subunit PsaC, containing [4Fe-4S] clusters FA and FB. Conformational change upon binding to Photosystem I.

*J. Biol Inorg. Chem.* **7**, 461–472.

1. Sakuragi, Y., Zybailov, B., Shen, G., Chitnis, P. R., van der Est, A., Bittl, R., Zech, S., Stehlik, D., Golbeck, J. H. and **Bryant**, D. A. 2002. Insertional inactivation of the *menG* gene, encoding 2-phytyl-1,4-naphthoquinone methyltransferase of *Synechocystis* sp. PCC 6803, results in the incorporation of 2-phytyl-1,4-naphthoquinone into the A1 site and alteration of the equilibrium constant between A1 and FX in Photosystem I. *Biochemistry* **41**, 394–405.
2. Eisen, J. A., Nelson, K. E., Paulsen, I. T., Heidelberg, J. F., Wu, M., Dodson, R. J., Deboy, R.,

Gwinn, M. L., Nelson, W. C., Haft, D. H., Hickey, E. K., Peterson, J. D., Durkin, A. S., Kolonay,

J. L., Yang, F., Holt, I., Umayam, L. A., Mason, T., Brenner, M., Shea, T. P., Parksey, D.,

Feldblyum, T. V., Hansen, C. L., Craven, M. B., Radune, D., Khouri, H., Fujii, C. Y., White, O., Venter J. C., Volfovsky, N., Gruber, T. M., Ketchum, K. A., Tettelin, H., **Bryant**, D. A., and Fraser, C. M. 2002. The complete genome sequence of the green sulfur bacterium *Chlorobium tepidum*. *Proc. Natl. Acad. Sci. U.S.A.* **99**, 9509–9514.

1. Vassilieva, E. V., Stirewalt, V. L., Jakobs, C. U., Frigaard, N.-U., Baker, M. A., Sotak, A., and **Bryant**, D. A. 2002. Cellular localization of chlorosome proteins in *Chlorobium tepidum*. Cloning and characterization of genes encoding CsmH, CsmF, and three addtional chlorosome- associated polypeptides. *Biochemistry* **41**, 4358–4370.
2. Safarov, N., Miletti, S., Ciurli, S., Christensen, S. K., Kornetzky, K., **Bryant**, D. A., Vendenberghe, I., Devreese, B., Remaut, H., and Van Beeumen, J. V. 2002. Molecular characterization of *Bacillus pasteurii* UreE, a metal-binding chaperone for assembly of the urease active site. *J. Biol. Inorg. Chem.* **7**, 623–631.
3. Sakamoto, T. and **Bryant**, D. A. 2002. Synergistic effect of high light and low temperature on cell growth of the i112 fatty acid desaturase mutant in *Synechococcus* sp. PCC 7002. *Photosynth. Res.* **42**, 231–242.
4. Camarero, J. A., Shekhtman, A., Campbell, E., Chlenov, M., Gruber, T. M., **Bryant**, D. A., Darst, S. A., Cowburn, D. and Muir, T. W. 2002. Autoregulation of a bacterial sigma factor explored using segmental isotopic labeling and NMR. *Proc. Natl. Acad. Sci. USA* **99**, 8536– 8541.
5. Frigaard, N.-U., Voigt, G. D. and **Bryant**, D. A. 2002. A bacteriochlorophyll *c*-less mutant of *Chlorobium tepidum* made by inactivation of the *bchK* gene encoding bacteriochlorophyll *c* synthase. *J. Bacteriol.* **184**, 3368–3376.
6. Scott, N. L., Falzone, C. J., Vuletich, D. A., Zhao, J., **Bryant**, D. A., and Lecomte, J. T. J. 2002. Truncated hemoglobin from the cyanobacterium *Synechococcus* sp. PCC 7002. Evidence for hexacoordination and covalent adduct formation in the ferric recombinant protein. *Biochemistry* **41**, 6902–6910.
7. Shen, G., Zhao, J., Antonkine, M. L., Reimer, S. K., Weiland, S., van der Est, A., Stehlik, D., Bittl, R., Golbeck, J. H. and **Bryant**, D. A. 2002. Assembly of the [4Fe-4S] Clusters in Photosystem I. I. The *rubA* gene product is required for assembly of the [4Fe-4S] cluster FX of photosystem I. *J. Biol. Chem.* **277**, 20343–20354.
8. Shen, G., Antonkine, M. L., van der Est, A., Vassiliev, I. R., Brettel, K., Bittl, R., Zhao, J., Stehlik, D., **Bryant**, D. A. and Golbeck, J. H. 2002. Assembly of the [4Fe-4S] Clusters in Photosystem I. II. Rubredoxin RubA is required for the *in vivo* assembly of FX as shown by optical and EPR spectroscopy. *J. Biol. Chem.* **277**, 20355–20366.
9. **Bryant**, D. A., Vassilieva, E. V., Frigaard, N.-U., and Li, H. 2002. Selective protein extraction from *Chlorobium tepidum* chlorosomes using detergents. Evidence that CsmA forms multimers and binds bacteriochlorophyll *a*. *Biochemistry* **41**, 14403–14411.
10. Frigaard, N.-U., Li, H., Gomez Maqueo Chew, A., Maresca, J. A. and **Bryant**, D. A. 2003. *Chlorobium tepidum*: insights into the physiology and biochemistry of green sulfur bacteria from the complete genome sequence. *Photosynth. Res.* **78**, 93–117.
11. Yu, J., Shen, G., Wang, T., **Bryant**, D. A., Golbeck, J. H., and McIntosh, L. 2003. Suppressor mutations in the study of Photosystem I biogenesis: *sll0088* is a previously unidentified gene involved in reaction center accumulation in *Synechocystis* sp. Strain PCC 6803. *J. Bacteriol.* **185**, 3878–3887.
12. **Bryant**, D. A. 2003. The beauty in small things revealed. *Proc. Natl. Acad. Sci. USA* **100**, 9647– 9649.
13. Huang, C., Yuan, X., Zhao, J. and **Bryant**, D. A. 2003. Kinetic analyses of state transitions of the cyanobacterium *Synechococcus* sp. PCC 7002 and its mutant strains impaired in electron transport. *Biochim. Biophys. Acta* **1607**, 121–130.
14. Gomez-Lojero, C., Perez-Gomez, B., Shen, G., Schluchter, W. M., and **Bryant**, D. A. 2003. Interaction of ferredoxin:NADP^+^ oxidoreductase with phycobilisomes and phycobilisome substructures of *Synechococcus* sp. strain PCC 7002. *Biochemistry* **42**, 13800–13811.
15. Cheng, Z., Sattler, S., Maeda, H., Sakuragi, Y., **Bryant**, D. A., and DellaPenna, D. 2003. Highly divergent methyltransferases catalyze a conserved reaction in tocopherol and plastoquinone synthesis in cyanobacteria and photosynthetic eukaryotes. *The Plant Cell* **15**, 2343–2356.
16. Frigaard, N.-U., Sakuragi, Y., and **Bryant**, D. A. 2004. Gene inactivation in the cyanobacterium *Synechococcus* sp. PCC 7002 and the green sulfur bacterium *Chlorobium tepidum* using *in vitro*- made DNA constructs and natural transformation. In: Photosynthesis Research Protocols, R. Carpentier, ed. Humana Press, Totowa, NJ. *Meth. Mol. Biol.* **274**, 325–340.
17. Frigaard, N.-U., Li, H., Milks, K. J. and **Bryant**, D. A. 2004. Nine mutants of *Chlorobium tepidum* each unable to synthesize a different chlorosome protein still assemble functional chlorosomes. *J. Bacteriol.* **186**, 646–653.
18. Wang, T., Shen, G., Balasubramanian, R., McIntosh, L., **Bryant**, D. A., and Golbeck, J. H. 2004. SufR (Sll0088 in *Synechocystis* sp. PCC 6803) functions as a repressor of *sufBCDS* operon involved in iron-sulfur cluster biogenesis. *J. Bacteriol.* **186**, 956–967.
19. van der Est, A., Valieva, A. I., Kandrashin, Yu. E., Shen, G., **Bryant**, D. A. and Golbeck, J. H. 2004. Forward electron transfer in Photosystem I from subunit deletion mutants of *Synechococcus* sp. PCC 7002. *Biochemistry* **43**, 1264–1275.
20. Maresca, J. A., Gomez Maqueo Chew, A., Ros Ponsatí, M., Frigaard, N.-U., Ormerod, J. G., Jones, A. D., and **Bryant**, D. A. 2004. The *bchU* gene of *Chlorobium tepidum* encodes the bacteriochlorophyll C-20 methyltransferase. *J. Bacteriol.* **186**, 2558-2566.
21. Frigaard, N.-U., Maresca, J. A., Yunker, C. E., Jones, A. D. and **Bryant**, D. A. 2004. Genetic manipulation of carotenoid biosynthesis in the green sulfur bacterium *Chlorobium tepidum*. *J. Bacteriol.* **186**, 5210–5220.
22. Frigaard, N-U. and **Bryant**, D. A. 2004. Seeing green bacteria in a new light: genomics-enabled studies of the photosynthetic apparatus in green sulfur bacteria and filamentous anoxygenic phototrophic bacteria. *Arch. Microbiol.* **182**, 265–276.
23. Zambelli, B., Stola, M., De Vriendt, K., Samyn, B., Devreese, B., Van Beeumen, J., Turano, P., Dikiy, A., **Bryant**, D. A. and Ciurli, S. 2005. UreG, a chaperone in the urease assembly process, is an intrinsically unstructured GTPase that specifically binds Zn^2+^. *J. Biol. Chem.* **280**, 4684– 4695.
24. Maresca, J. A., Frigaard, N.-U., and **Bryant**, D. A. 2005. Identification of a novel class of lycopene cyclases in photosynthetic bacteria. Photosynthesis: Fundamental Aspects to Global Perspectives, Proceedings of the XIIIth International Congress on Photosynthesis, Montreal, (August 2004), pp. 884–886. A. van der Est and D. Bruce, eds. Allen Press, Lawrence, Kansas, USA.
25. Gomez Maqueo Chew, A., Frigaard, N.-U., and **Bryant**, D. A. 2005. Characterization of BchV, a C-3^1^ hydratase specific for hypermethylated bacteriochlorophyll *c* in *Chlorobium tepidum*.

Photosynthesis: Fundamental Aspects to Global Perspectives, Proceedings of the XIIIth International Congress on Photosynthesis, Montreal, (August 2004), pp. 875–877. A. van der Est and D. Bruce, eds. Allen Press, Lawrence, Kansas, USA.

1. Li, H., Frigaard, N.-U. and **Bryant**, D. A. 2005. Locations and interactions of chlorosome proteins on the chlorosome envelope in *Chlorobium tepidum*: Insights from cross-linking experiments. Photosynthesis: Fundamental Aspects to Global Perspectives, Proceedings of the XIIIth International Congress on Photosynthesis, Montreal, (August 2004), pp. 116–119. A. van der Est and D. Bruce, eds. Allen Press, Lawrence, Kansas, USA.
2. Shen, G., Balasubramanian, R., Wang, T., Tirupati, B., Bollinger, J. M., Golbeck, J. H., and **Bryant**, D. A. 2005. Functional genomics of genes for biogenesis of Fe-S proteins in cyanobacteria. Photosynthesis: Fundamental Aspects to Global Perspectives, Proceedings of the XIIIth International Congress on Photosynthesis, Montreal, (August 2004), pp. 882–884. A. van der Est and D. Bruce, eds. Allen Press, Lawrence, Kansas, USA.
3. Balasubramanian, R., Shen, G., Golbeck, J. H., and **Bryant**, D. A. 2005. SufR is a [4Fe-4S] protein that functions as a negative transcriptional regulator of the *suf* regulon in cyanobacteria. Photosynthesis: Fundamental Aspects to Global Perspectives, Proceedings of the XIIIth International Congress on Photosynthesis, Montreal, (August 2004), pp. 66–68. A. van der Est and D. Bruce, eds. Allen Press, Lawrence, Kansas, USA.
4. Stehlik, D., Pushkar, J., Karyagina, I., Brown, S., **Bryant**, D., Golbeck, J. 2005. Control of function by protein-cofactor interaction in the electron transfer reactions of photosystem I. In: Photosynthesis: Fundamental Aspects to Global Perspectives, Proceedings of the XIIIth International Congress on Photosynthesis, Montreal, (August 2004), pp. 25–27. A. van der Est and D. Bruce, eds. Allen Press, Lawrence, Kansas, USA.
5. **Bryant**, D. A., Frigaard, N.-U., Maresca, J. A., Gomez Maqueo Chew, A., and Li, T. 2005. Chlorophyll and carotenoid biosynthesis in green sulfur bacteria: a genomic perspective. Photosynthesis: Fundamental Aspects to Global Perspectives, Proceedings of the XIIIth International Congress on Photosynthesis, Montreal, (August 2004), pp. 866–869. A. van der Est and D. Bruce, eds. Allen Press, Lawrence, Kansas, USA.
6. Maeda, H., Sakuragi, Y., **Bryant**, D. A. and DellaPenna, D. 2005. Tocopherols are essential in protecting *Synechocystis* sp. strain PCC 6803 from lipid peroxidation. *Plant Physiol.* **138**, 1422– 1435.
7. Sakuragi, Y., Zybailov, B., Shen, G., **Bryant**, D. A., Golbeck, J. H., Diner, B. A., Karygina, I., Pushkar, Y., and Stehlik, D. 2005. Recruitment of a foreign quinone into the A1 Site of Photosystem I. Characterization of a *menB rubA* double mutant in *Synechococcus* sp. PCC 7002 devoid of FX, FA and FB and containing plastoquinone or exchanged 9,10-anthraquinone. *J. Biol. Chem*. **280**, 12371–12381.
8. Frigaard, N.-U., Li, H., Martinsson, P., Das, S. K., Frank, H. A., Aartsma, T. J. and **Bryant**, D. A. 2005. Isolation and characterization of carotenosomes from a bacteriochlorophyll *c*-less mutant of *Chlorobium tepidum*. *Photosynth. Res.* **86**, 101–111.
9. Mimuro, M., Tsuchiya, T., Itoh, Y., Inoue, H., Gotoh, T., Sakuragi, Y., Miyashita, H., Yamashita, T., **Bryant**, D. A. and Kobayashi, M. 2005. The secondary electron acceptor of photosystem I in *Gloeobacter violaceus* PCC 7421 is menaquinone-4. *FEBS Lett.* **279**, 3493– 3496.
10. Sakuragi, Y. and **Bryant**, D. A. 2006. Genetic manipulation of quinone biosynthesis in cyanobacteria. In: Advances in Photosynthesis and Respiration, Vol. 24, J. H. Golbeck (ed.) Photosystem I: The light-driven plastocyanin:ferredoxin oxidoreductase in photosynthesis, pp. 205–222. Springer, Dordrecht, The Netherlands.
11. Frigaard, N.-U., Gomez Maqueo Chew, A., Maresca, J. A. and **Bryant**, D. A. 2006. Bacteriochlorophyll biosynthesis in green bacteria. In: Advances in Photosynthesis and Respiration, Vol. 25, B. Grimm, R. Porra, W. Rüdiger, and H. Scheer (eds.), Chlorophylls and Bacteriochlorophylls: Biochemistry, Biophysics, Functions and Applications, pp. 201–221. Springer, Dordrecht, The Netherlands.
12. Nomura, C. T., Persson, S., Shen, G. Inoue-Sakamoto, K. and **Bryant**, D. A. 2006. Characterization of two cytochrome oxidase operons in the marine cyanobacterium *Synechococcus* sp. PCC 7002. Inactivation of *ctaDI* affects the PS I:PS II ratio. *Photosynth. Res.* **87**, 215–228.
13. Shively, J. B., Cannon, G. C., **Bryant**, D. A., DasSarma, S., Bazylinski, D., Preiss, J., Steinbüchel, A., and Docampo, R. 2006. Bacterial Inclusions. In: Encyclopedia of Life Sciences, Nature Publishing Group, Macmillan Reference Ltd., London, United Kingdom. <http://www.els.net/>
14. Frigaard, N.-U. and **Bryant**, D. A. 2006. Chlorosomes: antenna organelles in green photosynthetic bacteria. *In:* Complex Intracellular Structures in Prokaryotes (Shively, J. M., ed.), Microbiology Monographs, Vol. 2, pp. 79–114, Springer, Berlin, Germany.
15. Balasubramanian, R., Shen, G., **Bryant**, D. A, and Golbeck, J. H. 2006. Assembly of iron sulfur clusters in cyanobacteria: regulatory roles of *sufA* and *iscA* genes in redox sensing and iron metabolism in the cyanobacterium *Synechococcus* sp. PCC 7002. *J. Bacteriol.* **188**, 3182–3191.
16. Sakuragi, Y., Maeda, H., DellaPenna, D. and **Bryant**, D. A. 2006. α-Tocopherol plays a role in photosynthesis and macronutrient homeostasis that is independent of its antioxidant function in the cyanobacterium *Synechocystis* sp. PCC 6803. *Plant Physiol.* **141**, 508–521.
17. Ley, R. E., Harris, J. K., Wilcon, J., Spear, J. R., Miller, S. R., Bebout, B. M., Maresca, J. A., **Bryant**, D. A. and Pace, N. R. 2006. Unexpected diversity and complexity from the Guerrero Negro hypersaline microbial mat. *Appl. Env. Microbiol.* **72**, 3685–3695.
18. Nomura, C. T., Sakamoto, T. and **Bryant**, D. A. 2006. Roles for heme-copper oxidases in extreme high light and oxidative stress response in the cyanobacterium *Synechococcus* sp. PCC 7002. *Arch. Microbiol.* **185**, 471–479.
19. Shen, G., Saunée, N. A., Williams, S. R., Gallo, E. F., Schluchter, W. M. and **Bryant**, D. A. 2006. Identification and characterization of a new class of bilin lyase: the *cpcT* gene encodes a bilin lyase responsible for attachment of phycocyanobilin to Cys-153 on the �-subunit of phycocyanin in *Synechococcus* sp. PCC 7002. *J. Biol. Chem.* **281**, 17768–17778.
20. Li, H., Frigaard, N.-U., and **Bryant**, D. A. 2006. Molecular contacts for chlorosome envelope proteins revealed by cross-linking studies with chlorosomes from *Chlorobium tepidum*. *Biochemistry* **45**, 9095–9103.
21. **Bryant**, D. A. and Frigaard, N.-U. 2006. Prokaryotic photosynthesis and phototrophy illuminated. *Trends Microbiol.* **14**, 488–496.
22. Maresca, J. A., and **Bryant**, D. A. 2006. Identification of two genes encoding new carotenoid- modifying enzymes in the green sulfur bacterium *Chlorobium tepidum*. *J. Bacteriol.* **188**, 6217– 6223.
23. Gomez Maqueo Chew, A. and **Bryant**, D. A. 2007. Characterization of a plant-like protochlorophyllide *a* divinyl reductase in green sulfur bacteria. *J. Biol. Chem.* **282**, 2967–2975.
24. Woodger, F. J., **Bryant**, D. A. and Price, G. D. 2007. Transcriptional regulation of the CO2- concentrating mechanism in the euryhaline, coastal-marine cyanobacterium, *Synechococcus* sp. PCC7002: role of NdhR/CcmR. *J. Bacteriol.* **189**, 3335–3347.
25. Antonkine, M. L., Maes, E. M., Czernuszewicz, R. S., Brietenstein, C., Bill, E., Falzone, C. F., Balasubramanian, R., Yang, F., **Bryant**, D. A. and Golbeck, J. H. 2007. Chemical rescue of a site-modified ligand to a [4Fe-4S] cluster in a bacterial di-cluster ferredoxin. *Biochim. Biophys. Acta* **1767**, 712–724.
26. Klatt, C. G., **Bryant**, D. A. and Ward, D. M. 2007. Comparative genomics provides evidence for the 3-hydroxypropionate autotrophic pathway in filamentous anoxygenic phototrophic bacteria and in hot spring microbial mats. *Environ. Microbiol.* **9**, 2067–2078.
27. Kim, H., Li, H., Maresca, J. A., **Bryant**, D. A. and Savikhin, S. 2007. Triplet exciton formation as a novel photoprotection mechanism in chlorosomes of *Chlorobium tepidum*. *Biophys. J.* **93**, 192–201.
28. Ikonen, T. P., Li, H., Psencik, J., Laurinmaki, P., Butcher, S. J., Frigaard, N.-U., Serimaa, R. E., **Bryant**, D. A. and Tuma, R. 2007. X-ray scattering and electron cryomicroscopy study on the effect of carotenoid biosynthesis to the structure of *Chlorobium tepidum* chlorosomes. *Biophys.*

*J.* **93**, 620–628.

1. Gomez Maqueo Chew, A., and **Bryant**, D. A. 2007. Chlorophyll biosynthesis in bacteria: the origins of structural and functional diversity. *Annu. Rev. Microbiol.* **61**, 113–129.
2. Inoue-Sakamoto, K., Gruber, T. M., Christensen, S. K., Sakomoto, T. and **Bryant**, D. A. 2007. Group 3 sigma factors in the marine cyanobacterium *Synechococcus* sp. PCC 7002 are required for growth at low temperature. *J. Gen. Appl. Microbiol.* **53**, 89–104.
3. **Bryant**, D. A., Garcia Costas, A. M., Maresca, J. A., Gomez Maqueo Chew, A., Klatt, C. G., Bateson, M. M., Tallon, L. J. Hostetler, J., Nelson, W. C., Heidelberg, J. F., Ward, D. M. 2007. “*Candidatus* Chloracidobacterium thermophilum”: an aerobic phototrophic acidobacterium. *Science* **317**, 523–526.
4. Maresca, J. A., Graham, J. E., Wu, M., Eisen, J. A. and **Bryant**, D. A. 2007. Identification of a fourth family of lycopene cyclases in photosynthetic bacteria. *Proc. Natl. Acad. Sci. USA* **104**, 11784–11789.
5. Gomez Maqueo Chew, A., Frigaard, N.-U., and **Bryant**, D. A. 2007. Bacteriochlorophyllide *c* C- 8^2^ and C-12^1^ methyltransferases are essential for adaptation to low light in *Chlorobaculum tepidum*. *J. Bacteriol.* **189**, 6176–6184.
6. Shen, G., Balasubramanian, R., Wu, Y., Wang, T., Hoffart, L. M., Krebs, C., **Bryant**, D. A. and Golbeck, J. H. 2007. The SufR transcriptional repressor binds to the promoter region of the *sufBCDS* operon as a homodimer and coordinates [4Fe-4S]^1+,^ ^2+^ cluster. *J. Biol. Chem.* **282**, 31909–31919.
7. Oostergetel, G. T., Reus, M., Gomez Maqueo Chew, A., **Bryant**, D. A., Boekema, E. J., and Holzwarth, A. R. 2007. Long-range organization of bacteriochlorophyll in chlorosomes of *Chlorobium tepidum* investigated by cryo-electron microscopy. *FEBS Lett.* **581**, 5435–5439.
8. Gomez Maqueo Chew, A., Frigaard, N.-U., and **Bryant**, D. A. 2008. Identification of the gene encoding geranylgeranyl reductase, BchP, in *Chlorobaculum tepidum*. *J. Bacteriol.* **190**, 747– 749.
9. Sakamoto, T., Inoue-Sakamoto, K. Persson, S., and **Bryant**, D. A. 2008. The transcriptional activator NtcB specifically controls the nitrate assimilation genes in the marine cyanobacterium *Synechococcus* sp. PCC 7002. *Phycol. Res.* **56**, 223–237.
10. Garcia Costas, A. M., Graham, J. E., and **Bryant**, D. A. 2008. Ketocarotenoids in chlorosomes of *Candidatus* Chloracidobacterium thermophilum. In: *Energy from the Sun*, (J. F. Allen, E. Gantt, J. H. Golbeck and B. Osmond, eds.), pp. 1161–1164. Springer, Dordrecht, The Netherlands.
11. Ganapathy, S., Reus, M., Gomez Maqueo Chew, A., **Bryant**, D. A., Holzwarth, A. R., and de Groot, H. J. M. 2008. Structural assessment of the bacteriochlorophyll *d* stacking in chlorosomes from a *C. tepidum* mutant with MAS NMR spectroscopy. In: *Energy from the Sun*, (J. F. Allen,

E. Gantt, J. H. Golbeck and B. Osmond, eds.), pp. 247–251. Springer, Dordrecht, The Netherlands.

1. Ganapathy, S., Reus, M., Gomez Maqueo Chew, A., **Bryant**, D. A., Holzwarth, A. R., and de Groot, H. J. M. 2008. A comparative MAS NMR study of Bchl *d* and Bchl *c* producing mutants of *C. tepidum*. In: *Energy from the Sun*, (J. F. Allen, E. Gantt, J. H. Golbeck and B. Osmond, eds.), pp. 257–260. Springer, Dordrecht, The Netherlands.
2. Shen, G., Schluchter, W. M. and **Bryant**, D. A. 2008. Biogenesis of phycobiliproteins. I. *cpcS-I* and *cpcU* mutants of the cyanobacterium *Synechococcus* sp. PCC 7002 identify a heterodimeric phycocyanobilin lyase specific for β–phycocyanin and allophycocyanin subunits. *J. Biol. Chem.* **283**, 7503–7512.
3. Saunée, N. A., Williams, S. R., **Bryant**, D. A. and Schluchter, W. M. 2008. Biogenesis of phycobiliproteins. II. CpcS-I and CpcU comprise the heterodimeric bilin lyase that attaches phycocyanobilin to Cys-82 of β–phycocyanin and Cys-81 of allophycocyanin subunits in *Synechococcus* sp. PCC 7002. *J. Biol. Chem.* **283**, 7513–7522.
4. Frigaard, N.-U. and **Bryant**, D. A. 2008. Genomic insights into the sulfur metabolism of phototrophic sulfur bacteria. In: *Advances in Photosynthesis and Respiration*, Vol. 27, *Sulfur Metabolism in Phototrophic Organisms*, R. Hell, C. Dahl, D. B. Knaff, and T. Leustek, eds., pp. 343–361. Springer, Dordrecht, The Netherlands.
5. Frigaard, N.-U. and **Bryant**, D. A. 2008. Genomic and evolutionary perspectives on sulfur metabolism in green sulfur bacteria. In: *Microbial Sulfur Metabolism* (Proceedings of the International Symposium on Microbial Sulfur Metabolism), C. G. Friedrich and C. Dahl, eds., Münster, Germany, June 29-July 02, 2006, pp. 60–76. Springer, Dordrecht, The Netherlands.
6. Grimme, R. A., Lubner, C. E., **Bryant**, D. A. and Golbeck, J. H. 2008. Efficient photohydrogen production from Photosystem I and noble metal nanoparticles joined by a molecular wire. *J. Am. Chem. Soc.* **130**, 6308–6309.
7. Carrieri, D., Ananyev, G., Garcia Costas, A. M., **Bryant**, Donald A. and Dismukes, G. C. 2008. Renewable hydrogen production by cyanobacteria: nickel requirements for optimal hydrogenase activity. *Int. J. Hydrogen Energy* **33**, 2014–2022.
8. Zhao, F., Zhao, F., Li, T. and **Bryant**, D. A. 2008. A new pheromone-based genetic algorithm for comparative genome assembly. *Nucl. Acids Res.* **36**, 3455–3462.
9. Maresca, J. A., Graham, J. E., and **Bryant**, D. A. 2008. Carotenoid biosynthesis in chlorophototrophs: the biochemical and genetic basis for structural diversity. *Photosynth. Res.* **97**, 121–140.
10. Shen, G., Leonard, H. S., Schluchter, W. M. and **Bryant**, D. A. 2008. CpcM post-translationally methylates asparagine-71/72 of phycobiliprotein beta subunits in *Synechococcus* sp. PCC 7002 and *Synechocystis* sp. PCC 6803. *J. Bacteriol.* **190**, 4808–4817.
11. Miller, C. A., Leonard, H. S., Pinsky, I. G., Turner, B. M., Williams, S. R., Harrison, L., Jr., Fletcher, A. F., Shen, G., **Bryant**, D. A. and Schluchter, W. M. 2008. Biogenesis of phycobiliproteins. III. CpcM is the asparagine methyltransferase for phycobiliprotein beta subunits in cyanobacteria. *J. Biol. Chem.* **283**, 19293–19300.
12. Jin, Z., Heinnickel, M., Krebs, C., Shen, G., Golbeck, J. H. and **Bryant**, D. A. 2008. Biogenesis of iron-sulfur clusters in photosystem I: holo-NfuA from the cyanobacterium *Synechococcus* sp. PCC 7002 rapidly and efficiently transfers [4Fe-4S] clusters to apo-PsaC *in vitro*. *J. Biol. Chem.* **283**, 28426–28435.
13. Agalarov, R., Byrdin, M., Rappaport, F., Shen, G., **Bryant**, D. A., van der Est, A., and Golbeck,

J. H. 2008. Removal of the PsaF polypeptide alters the relative amplitudes of the fast and slow kinetic phases attributed to A1B- and A1A- oxidation in Photosystem I from *Synechococcus* sp. PCC 7002. *Photochem. Photobiol.* **84**, 1371–1380.

1. Graham, J. E., Lecomte, J. T. J., and **Bryant**, D. A. 2008. Synechoxanthin, an aromatic C40 xanthophyll that is a major carotenoid in the cyanobacterium *Synechococcus* sp. PCC 7002. *J. Nat. Prod.* **71**, 1647–1650.
2. Maresca, J. A., Romberger, S. P., and **Bryant**, D. A. 2008. Isorenieratene biosynthesis in green sulfur bacteria requires the cooperative actions of two carotenoid cyclases. *J. Bacteriol.* **190**, 6384–6391.
3. Graham, J. E. and **Bryant**, D. A. 2008. The biosynthetic pathway for synechoxanthin, an aromatic carotenoid synthesized by the euryhaline, unicellular cyanobacterium *Synechococcus* sp. strain PCC *Synechococcus* sp. PCC 7002. *J. Bacteriol.* **190**, 7966–7974.
4. Wu, D., Raymond, J., Wu, M., Chatterji, S., Ren, Q., Graham, J. E., **Bryant**, D. A., Robb, F., Colman, A., Tallon, L. J., Badger, J. Madupu, R., Ward, N. and Eisen, J. A. 2009. Complete genome sequence of the aerobic CO-oxidizing thermophile, *Thermomicrobium roseum*. *PLoS One* **4**, e4207.
5. Shively, J. M., Cannon, G. C., Heinhorst, S., Fuerst, J. A., **Bryant**, D. A., Gantt, E., Maupin- Furlow, J. A., Schüler, D., Pfeifer, F., Docampo, R., Dahl, C., Preiss, J., Steinbüchel, A., and Federici, B. A. 2009. Intracellular structures of prokaryotes: inclusions, compartments, and assemblages. Encyclopedia of Microbiology (Schaechter, M., ed.), pp. 404–424. Elsevier, Amsterdam, The Netherlands.
6. Graham, J. E., and **Bryant**, D. A. 2009. The biosynthetic pathway for the synthesis of the myxol- 2'-fucoside in the cyanobacterium *Synechococcus* sp. strain PCC 7002. *J. Bacteriol.* **191**, 3292– 3300.
7. Ganapathy, S., Oostergetel, G. T., Wawrzyniak, P. K., Reus, M., Gomez Maqueo Chew, A., Buda, F., Boekema, E. J., **Bryant**, D. A., Holzwarth, A. R., and de Groot, H. J. M. 2009.

Alternating *syn-anti* bacteriochlorophylls form concentric helical nanotubes in chlorosomes.

*Proc. Natl. Acad. Sci. USA* **106**, 8525–8530.

1. Dong, C., Tang, A., Zhao, J., Mullineaux, C. W., Shen, G. and **Bryant**, D. A. 2009. Direct energy transfer from ApcD of phycobilisomes to photosystem I and its role in protection from photoinhibition in the cyanobacterium *Synechococcus* sp. PCC 7002. *Biochim. Biophys. Acta* **1787**, 1122–1128.
2. Gomez Maqueo Chew, A., Frigaard, N.-U., and **Bryant**, D. A. 2009. Mutational analysis of three *bchH* paralogs in (bacterio)-chlorophyll biosynthesis in *Chlorobaculum tepidum*. *Photosynth. Res.* **101**, 21–34.
3. Li, H., Jubelirer, S., Garcia Costas, A. M., Frigaard, N.-U., and **Bryant**, D. A. 2009. Multiple antioxidant proteins protect *Chlorobaculum tepidum* against oxygen and reactive oxygen species. *Arch. Microbiol.* **191**, 853–867.
4. Li, H. and **Bryant**, D. A. 2009. Envelope proteins of the CsmB/CsmF and CsmC/CsmD motif families help determine the size, shape and composition of chlorosomes in *Chlorobaculum tepidum*. *J. Bacteriol.* **191**, 7109–7120.
5. Lubner, C. E., Grimme, R. G., **Bryant**, D. A. and Golbeck, J. H. 2010. Wiring photosystem I for light-induced hydrogen production. *Biochemistry* **49**, 404–414.
6. Schluchter, W. M., Shen, G., Alvey, R. M., Biswas, A., Saunée, N. A., Williams, S. R., Miller,

C. A., and **Bryant**, D. A. 2010. Phycobiliprotein biosynthesis in cyanobacteria: structure and function of enzymes involved in post-translational modification. *Adv. Exp. Med. Biol.* **675**, 211– 228.

1. Tsukatani, Y., Wen, J., Blankenship, R. E., and **Bryant**, D. A. 2010. Characterization of the bacteriochlorophyll *a*-binding, Fenna-Matthews-Olson protein from *Candidatus* Chloracidobacterium thermophilum. *Photosynth. Res.* **104**, 201–209.
2. Biswas, A., Vasquez, Y. M., Dragomani, T. M., Kronfel, M. L., Williams, S. R., Alvey, R. M., **Bryant**, D. A. and Schluchter, W. S. 2010. Biosynthesis of cyanobacterial phycobiliproteins in *Escherichia coli*: chromophorylation efficiency and specificity of all bilin lyases from *Synechococcus* sp. strain PCC 7002. *Appl. Environ. Microbiol.* **76**, 2729–2739.
3. Wenter, R., Hütz, K., Dibbern, D., Li, T., Reisinger, V., Plöscher, M., Eichacker, L., Eddie, B., Hanson, T. E., **Bryant**, D. A., and Overmann, J. 2010. Expression based identification of genetic determinants of the bacterial symbiosis in *‘Chlorochromatium aggregatum.’ Environ. Microbiol.* **12**, 2259–2276.
4. van der Meer, M. T. J., Klatt, C. G., Wood, J., **Bryant**, D. A., Bateson, M. M., Lammerts, L., Schouten, S., Sinninghe Damsté, J. S., Madigan, M. T. and Ward, D. M. 2010. Cultivation and genomic, nutritional and lipid biomarker characterization of *Roseiflexus* sp. strains closely related to predominant *in situ*. *J. Bacteriol.* **192**, 3033–3042.
5. McNeely, K., Xu, Y., Bennette, N. Ananyev, G., **Bryant**, D. A. and Dismukes, G. C. 2010. Metabolic engineering of fermentative carbon metabolism stimulates solar hydrogen production in the cyanobacterium *Synechococcus* sp. PCC 7002. *Appl. Environ. Microbiol.* **76**, 5032–5038.
6. Zhu, Y., Graham, J. E., Ludwig, M., Xiong, W., Alvey, R. M., Shen, G., and **Bryant**, D. A. 2010. Roles of xanthophyll carotenoids in protection against photoinhibition and oxidative stress in the cyanobacterium *Synechococcus* sp. strain PCC 7002. *Arch. Biochem. Biophys.* **504**, 86–99.
7. Scott, N. L., Xu, Y., Shen, G., Vuletich, D. A., Falzone, C. J., Li, Z., Ludwig, M., Pond, M., Preimesberger, M. R., **Bryant**, D. A., and Lecomte, J. T. J. 2010. Functional and structural characterization of the 2/2 hemoglobin of *Synechococcus* sp. PCC 7002. *Biochemistry* **49**, 7000– 7011.
8. Brown, I. I., **Bryant**, D. A., Casamatta D., Thomas-Keprta, K., Sarkisova, S. A., Shen, G., Graham, J. E., Boyd, E. S., Garrison, D. H., Peters, J. W., and McKay D. S. 2010. Polyphasic characterization of a thermotolerant, siderophilic filamentous cyanobacterium that produces intracellular and extracellular iron deposits. *Appl. Environ. Microbiol.* **76**, 6664–6672.
9. Carrieri, D., Momot, D., Brasg, I. A., Ananyev, G., Lenz, O., **Bryant**, D. A. and Dismukes, G. C. 2010. Boosting autofermentation rates and product yields with sodium stress cycling: Application to renewable fuel production by cyanobacteria. *Appl. Environ. Microbiol.* **76**, 6455– 6462.
10. Lubner, C. E., Knörzer, P., Silva, P., Vincent, K. A., Happe, T., **Bryant**, D. A. and Golbeck, J.

H. 2010. Wiring and [FeFe]-hydrogenase with photosystem I for light-induced hydrogen production. *Biochemistry* **49**, 10264–10266.

1. Wen, J., Tsukatani, Y., Cui, W., Zhang H., Gross, M. L., **Bryant**, D. A. and Blankenship, R. E. 2011. Structural model and spectroscopic characteristics of the FMO antenna protein from the aerobic chlorophototroph *Candidatus* Chloracidobacterium thermophilum. *Biochim. Biophys. Acta* **1807**, 157–164.
2. Xu, Y., Alvey, R. M., Byrne, P. O., Graham, J. E., Shen, G. and **Bryant**, D. A. 2011. Expression of genes in cyanobacteria: adaptation of endogenous plasmids as platforms for high-level gene expression in *Synechococcus* sp. PCC 7002. *Methods Mol. Biol.* **684**, 273–293.
3. McNeely, K., Xu, Y., Ananyev, G., Bennette, N., **Bryant**, D. A., and Dismukes, G. C. 2011. *Synechococcus* sp. strain PCC 7002 *nifJ* mutant lacking pyruvate:ferredoxin oxidoreductase. *Appl. Environ. Microbiol.* **77**, 2435–2444.
4. Alvey, R. M., Biswas, A., Schluchter, W. M, and **Bryant**, D. A. 2011. Effects of modified phycobilin biosynthesis in the cyanobacterium *Synechococcus* sp. strain PCC 7002. *J. Bacteriol.* **193**, 1663–1671.
5. Cannon, W. R., Rawlins, M. M., Baxter, D. J., Lipton, M., Callister, S., and **Bryant**, D. A. 2011. Large improvements in MS/MS-based peptide identification rates using a hybrid analysis. *J. Proteomic Res.* **10**, 2306–2317.
6. Ludwig, M. and **Bryant**, D. A. 2011. Transcription profiling of the cyanobacterium

*Synechococcus* sp. PCC 7002 using high-throughput cDNA sequencing. *Front. Microbiol.* **2**, 41.

1. Klatt, C. G., Wood, J. M., Rusch, D. B., Bateson, M. M., Hamamura, N., Heidelberg, J. F.,

Grossman, A. R., Bhaya, D., Cohan, F. M., Kühl, M., **Bryant**, D. A., and Ward, D. M. 2011. Community ecology of hot spring cyanobacterial mats: predominant populations and their functional potential. *ISME J.* **5**, 1262–1278.

1. Liu, Z., Klatt, C. G., Wood, J. M., Rusch, D. B., Wittekindt, N., Tomsho, L. P., Schuster, S. C., Ward, D. M., and **Bryant**, D. A. 2011. Metatranscriptomic analyses of chlorophototrophs of a hot-spring microbial mat. *ISME J.* **5**,1279–1290. doi: 10.1038/ismej.2011.37
2. Lubner, C. E., **Bryant**, D. A. and Golbeck, J. H. 2011. Wired reaction centers. In: *Molecular Solar Fuels*, (Hillier, W. and Wydrzynski, T., eds.), pp. 464–505. Angus & Robertson, London, United Kingdom.
3. Furumaki, S., Vacha, F., Habuchi S., Tsukatani, Y., **Bryant**, D. A. and Vacha, M. 2011. Absorption linear dichroism measured directly on a single light-harvesting system: the role of disorder in chlorosomes of green photosynthetic bacteria. *J. Am. Chem. Soc.* **133**, 6703–6710.
4. Lubner, C. E., Heinnickel, M., **Bryant**, D. A. and Golbeck, J. H. 2011. Wiring photosystem I for electron transfer to a tethered redox dye. *Energy Environ. Sci.* **4**, 2428–2434.
5. Gregersen, L. H., **Bryant**, D. A. and Frigaard, N.-U. 2011. Components and evolution of oxidative sulfur metabolism in green sulfur bacteria. *Front. Microbiol.* **2**, 116.
6. Alvey, R. M., Biswas, A., Schluchter, W. M., and **Bryant**, D. A. 2011. Attachment of non- cognate chromophores to CpcA of *Synechocystis* sp. PCC 6803 and *Synechococcus* sp. PCC 7002 by heterologous expression in *Escherichia coli*. *Biochemistry* **50**, 4890–4902.
7. Liu, Z. and **Bryant**, D. A. 2011. Identification of a gene essential for the first committed step in the synthesis of bacteriochlorophyll *c*. *J. Biol. Chem.* **286**, 22393-22402.
8. Liu, Z. and **Bryant**, D. A. 2012. Biosynthesis and assembly of bacteriochlorophyll *c* in green bacteria: theme and variations. In: *Handbook of Porphyrin Science*, Vol. 20, pp. 108–142. Kadish, K. M., Smith, K. M., Guilard, R., (eds.). World Scientific Publishing, Hackensack, NJ, USA.
9. Hamilton, T. L., Ludwig, M., Dixon, R., E. S., Dos Santos, P. C., Setubal, J. C., **Bryant**, D. A., Dean, D. R. and Peters, J. W. 2011. Transcriptional profiling of nitrogen fixation in *Azotobacter vinelandii*. *J. Bacteriol.* **193**, 4477–4486.
10. Hamilton, T. L., Jacobson, M., Ludwig, M., Boyd, E. S., **Bryant**, D. A., Dean, D. R. and Peters,

J. W. 2011. Differential accumulation of *nif* structural gene mRNA in *Azotobacter vinelandii*. *J. Bacteriol.* **193**, 4534–4536.

1. Liu, Z. and **Bryant**, D. A. 2011. Multiple types of 8-vinyl reductases for (bacterio)chlorophyll biosynthesis occur in some green sulfur bacteria. *J. Bacteriol.* **193**, 4996–4998.
2. Carrieri, D., Ananyev, G., Lenz, O., **Bryant**, D. A., and Dismukes, G. C. 2011. A sodium ion gradient contributes to energy conservation during fermentation in the cyanobacterium *Arthrospira (Spirulina) maxima* CS-328. *Appl. Environ. Microbiol.* **77**, 7185–7194.
3. Zhang, S. and **Bryant**, D. A. 2011. The cyanobacterial tricarboxylic acid cycle. *Science* **334**, 1551–1553.
4. Biswas, A., Boutaghou, M. N., Alvey, R. M., Kronfel, C. M., Cole, R. M., **Bryant**, D. A. and Schluchter, W. M. 2011. Characterization of the CpeY, CpeZ, and CpeS bilin lyases involved in phycoerythrin biosynthesis in *Fremyella diplosiphon* strain UTEX 481 (*Tolypothrix* tenuis strain PCC 7601). *J. Biol. Chem.* **286**, 35509–35521.
5. Vogl, K. and **Bryant**, D. A. 2011. Elucidation of the biosynthetic pathway for okenone in *Thiodictyon* sp. CAD16 leads to the discovery of two novel carotene ketolases. *J. Biol. Chem.* **286**, 38521–38532.
6. Kiss, H., Nett, M., Domin, N., Martin, K., Maresca, J. A., Copeland, A., Lapidus, A., Lucas, S., Berry, K. W., Glavina Del Rio, T., Dalin, E., Tice, H., Pitluck, S., Richardson, P., Bruce, D., Goodwin, L., Han, C., Detter, J. C., Schmutz, J., Brettin, T., Larimer, F., Land, M., Hauser, L., Kyrpides, N. C., Ivanova, N., Göker, M, Woyke, T., Klenk, H.-P. and **Bryant**, D. A. 2011. Complete genome sequence of the filamentous gliding predator *Herpetosiphon aurantiacus* type strain (114-95^T^/DSM 785). *Stand. Genom. Sci.* **5**, 356–370.
7. Shively, J. M., Cannon, G. C., Heinhorst, S., **Bryant**, D. A., DasSarma, S., Bazylinski, D., Preiss, J., Steinbüchel, A., Docampo, R., and Dahl, C., 2011. Bacterial Inclusions. In: Encyclopedia of Life Sciences, in press. Nature Publishing Group, Macmillan Reference Ltd., London, United Kingdom. <http://www.els.net/>
8. Garcia Costas, A. M., Tsukatani, Y., Romberger, S. P., Oostergetel, G., Boekema, E., Golbeck, J. H., and **Bryant**, D. A. 2011. Ultrastructural analysis and identification of envelope proteins of “*Candidatus* Chloracidobacterium thermophilum” chlorosomes. *J. Bacteriol.* **193**, 6701–6711.
9. Lubner, C. E., Applegate, A. M., Knörzer P., Ganago, A., **Bryant**, D. A., Happe, T., and Golbeck, J. H. 2011. A solar hydrogen-producing bio-nanodevice that outperforms natural photosynthesis. *Proc. Natl. Acad. Sci. USA* **108**, 20988–20991.
10. Klotz, M. G., **Bryant**, D. A. and Hanson, T. E. 2011. The microbial sulfur cycle. *Front. Microbiol.* **2**, 241.
11. Vogl, K. and **Bryant**, D. A. 2012. The biosynthetic pathway of the important biomarker okenone: x-ring formation. *Geobiology* **10**, 205-215.
12. Hamilton, T. L., Vogl, K., **Bryant**, D. A., Boyd, E. S. and Peters, J. W. 2012. Physico-chemical parameters affecting the diversity of chlorophototrophs in the Yellowstone Geothermal Complex. *Geobiology* **10**, 236-249.
13. Tsukatani, Y., Romberger, S. P., Golbeck, J. H. and **Bryant**, D. A. 2012. Isolation and characterization of homodimeric type-1 reaction center complex from “*Candidatus* Chloracidobacterium thermophilum,” an aerobic chlorophototroph. *J. Biol. Chem.* **287**, 5720- 5732. doi: 10.1074/jbc.M111.323329
14. Cao, L., Schepmoes, A. A., Vogl, K., **Bryant**, D. A., Smith, R. D., Lipton, M. S., and Callister,

S. J. 2012. Comparison of aerobic and photoheterotrophic proteomes of *Chloroflexus aurantiacus* j-10-fl proteomes. *Photosynth. Res.* **110**, 153–168.

1. Garcia Costas, A. M., Liu, Z., Tomsho, L. P., Schuster, S. C., Ward, D. M. and **Bryant**, D. A. 2012. Complete genome of *Candidatus* Chloracidobacterium thermophilum, a chlorophyll-based photoheterotroph belonging to the phylum *Acidobacteria*. *Environ. Microbiol.* **14**, 177–190.
2. Ward, D. M., Klatt, C. G., Wood, J., Cohan, F. M., and **Bryant**, D. A. 2012. Functional genomics in an ecological and evolutionary context: maximizing the value of genomes in systems biology. In: *Advances in Photosynthesis and Respiration*, Vol. 35, *Functional Genomics and Evolution of Photosynthetic Systems* (Burnap, R. L. and Vermaas, W., eds.), pp. 1–16, Springer, Dordrecht, The Netherlands.
3. **Bryant**, D. A., Liu, Z., Li, T., Zhao, F., Garcia Costas, A. M., Klatt, C. G., Ward, D. M., Frigaard, N.-U., and Overmann, J. 2012. Comparative and functional genomics of anoxygenic green bacteria from the taxa *Chlorobi*, *Chloroflexi*, and *Acidobacteria.* In: *Advances in Photosynthesis and Respiration*, Vol. 35, *Functional Genomics and Evolution of Photosynthetic Systems*, (Burnap, R. L. and Vermaas, W., eds.), pp. 47–102, Springer, Dordrecht, The Netherlands.
4. Garcia Costas, A. M., Tsukatani, Y., Rijpstra, W. I. C., Schouten, S., Welander, P. V., Summons,

R. E. and **Bryant**, D. A. 2012. Identification of the bacteriochlorophylls, carotenoids, quinones, lipids, and hopanoids of “*Candidatus* Chloracidobacterium thermophilum.” *J. Bacteriol.* **194**, 1158–1168.

1. Wong, C. Y., Alvey, R. M., Wilk, K. E., **Bryant**, D. A., Curmi, P. M. G., Silbey, R. J., and Scholes, G. D. 2012. Quantum beat line shapes reveal hidden energy correlations in cryptophytan light-harvesting protein phycoerythrin 545. *Nature Chem.* **4**, 396–404.
2. Ganapathy, S., Oostergetel, G. T., Reus, M., Tsukatani, Y., Gomez Maqueo Chew, A., Buda, F., **Bryant**, D. A., Holzwarth, A. R., and de Groot, H. J. M. 2012. Self-assembly of BChl *c* in chlorosomes of the green sulfur bacterium, *Chlorobaculum tepidum*: a comparison of the *bchQR* mutant and the wild type. *Biochemistry* **51**, 4488–4498.
3. Liu, Z., Klatt, C. G., Ludwig, M., Rusch, D. B., Jensen, S. I., Kühl, M., Ward, D. M. and **Bryant**,

D. A. 2012. “*Candidatus* Thermochlorobacter aerophilum”: an aerobic chlorophotoheterotrophic member of the phylum *Chlorobi*. *ISME J.* **6**, 1869–1882.

1. Ludwig, M. and **Bryant**, D. A. 2012. Acclimation of the global transcriptome of the cyanobacterium *Synechococcus* sp. strain PCC 7002 to nutrient limitations and alternative nitrogen sources. *Front. Microbiol.* **3**, 145.
2. Liu, Z., Frigaard, N.-U., Vogl, K., Overmann, J., Iino, T., Ohkuma, M., and **Bryant**, D. A. 2012. *Ignavibacterium album*: complete genome sequence of a non-chlorophototrophic member of the phylum *Chlorobi*. *Front. Microbiol.* **3**, 185.
3. Vogl, K., Tank, M., Orf, G. S., Blankenship, R. E., and **Bryant**, D. A. 2012. Bacteriochlorophyll

*f*: properties of chlorosomes containing the “forbidden chlorophyll.” *Front. Microbiol.* **3**, 298.

1. Ludwig, M. and **Bryant**, D. A. 2012. Acclimation of the *Synechococcus* sp. strain PCC 7002 transcriptome to temperature, salinity and mixotrophic growth conditions. *Front. Microbiol.* **3**, 354.
2. Furumaki, S., Yabiku, Y., Habuchi, S., Tsukatani, Y., **Bryant**, D. A., and Vacha, M. 2012. Circular dichroism measured on single chlorosomal light-harvesting complexes of green photosynthetic bacteria. *J. Phys. Chem. Lett.* **3**, 3545–3549.
3. **Bryant**, D. A. 2013. Green bacteria: chlorophyll biosynthesis, light harvesting, reaction centers and electron transport. *In:* Lennarz, W. and Lane, D. M. (eds.), The Encyclopedia of Biological Chemistry, Volume 2, pp. 501–509. Academic Press, Waltham, Massachusetts, USA.
4. Xu, Y., Guerra, L. T., Li, Z., Dismukes, G. C. and **Bryant**, D. A. 2013. Altered carbohydrate metabolism in glycogen synthase mutants of *Synechococcus* sp. strain PCC 7002. *Metab. Eng.* **16**, 56–67.
5. Klatt, C. G., Liu, Z., Ludwig, M., Kühl, M., Jensen, S. I., **Bryant**, D. A. and Ward, D. M. 2013. Temporal metatranscriptomic patterning in phototrophic *Chloroflexi* inhabiting microbial mat in a geothermal spring. *ISME J.* **7**, 1775–1789.
6. Orf, G. S., Tank, M., Vogl, K., Niedzweidzki, D. M., **Bryant**, D. A., and Blankenship, R. E. 2013. Spectroscopic insights into the decreased efficiency of chlorosomes containing bacteriochlorophyll *f*. *Biochim. Biophys. Acta* **1827**, 493–501.
7. Li, H., Frigaard, N.-U., and **Bryant**, D. A. 2013. [2Fe-2S] proteins in chlorosomes. I. Construction and characterization of mutants lacking CsmI, CsmJ, and CsmX in the chlorosome envelope of *Chlorobaculum tepidum*. *Biochemistry* **52**, 1321–1330.
8. Johnson, T. W., Li, H., Frigaard, N.-U., Golbeck, J. H., and **Bryant**, D. A. 2013. [2Fe-2S] proteins in chlorosomes. II. Redox titration of the [2Fe-2S] clusters in the proteins CsmI, CsmJ, and CsmX in the chlorosome envelope of *Chlorobaculum tepidum. Biochemistry* **52**, 1331–1343.
9. **Bryant**, D. A. and Liu, Z. 2013. Green bacteria: insights into green bacterial evolution through genomic analyses. Advances in Botanical Research, Volume 66, *Genome evolution of photosynthetic bacteria* (Beatty, J. T., ed.), pp. 99–150. Elsevier, New York, NY.
10. Inskeep, W. P., Jay, Z. J., Tringe, S. G., Herrgard, M., Rusch, D. B., and the YNP Metagenome Project Steering Committee and Working Group Members. 2013. The YNP metagenome project: environmental parameters responsible for microbial distribution in the Yellowstone geothermal ecosystem. *Front. Microbiol.* **4**, 67.
11. Klatt, C. G., Inskeep, W. P., Herrgard, M., Jay, Z. J., Rusch, D. B., Tringe, S. G., Parenteau, M. N., Ward, D. M., Boomer, S. M., **Bryant**, D. A., and Miller, S. R. 2013. Community structure and function of high-temperature phototrophic microbial mats inhabiting diverse geothermal environments. *Front. Microbiol.* **4**, 106.
12. Guerra, L. T., Xu, Y., Bennette, N., McNeely, K., **Bryant**, D. A., Dismukes, G. C. 2013. Metabolic analysis of an ADP-glucose pyrophosphorylase deficient mutant of *Synechococcus* sp. PCC 7002: Evidence that glycogen is the preferred substrate during auto-fermentation. *J. Biotech.* **166**, 65–75.
13. Adams, P. G., Cadby, A. J., Robinson, B., Tsukatani, Y., Tank, M., Wen, J., Blankenship, R. E., **Bryant**, D. A. and Hunter, C. N. 2013. Comparison of the physical characteristics of chlorosomes from three different phyla of green phototrophic bacteria. *Biochim. Biophys. Acta* **1827**, 1235–1244.
14. Kumaraswamy, G. K., Guerra, T., Qian, X., Zhang, S., **Bryant**, D. A. and G. C. Dismukes. 2013. Reprogramming the glycolytic pathway for increased hydrogen production in cyanobacteria: metabolic engineering of NAD^+^-dependent GAPDH. *Energy Environ. Sci.* **6**, 3722–3731.
15. Saunders, A. M., Golbeck, J. H. and **Bryant**, D. A. 2013. Characterization of BciB, a ferredoxin- dependent 8-vinyl protochlorophyllide reductase in (bacterio)chlorophyll biosynthesis. *Biochemistry* **52**, 8442–8451.
16. Kronfel, C. M., Kuzin, A. P., Forouhar, F., Biswas, A., Su, M., Lew, S., Seetharaman, J., Xiao,

R., Everett, J. K., Ma, L.-C., Acton, T. B., Montelione, G. T., Hunt, J. F., Paul, C. E. C.,

Dragomani, T. M., Boutaghou, M. N., Cole, R. B., Riml, C., Alvey, R. M., **Bryant**, D. A., and Schluchter, W. M. 2013. Structural and biochemical characterization of the bilin lyase CpcS from *Thermosynechococcus elongatus*. *Biochemistry* **52**, 8663–8676.

1. Liu, Z., Müller, J., Li, T., Alvey, R. M., Vogl, K., Frigaard, N.-U., Rockwell, N. C., Tomsho, L. P., Schuster, S. C., Henke, P., Rohde, M., Overmann, J. and **Bryant**, D. A. 2013. Genomic analysis reveals key aspects of prokaryotic symbiosis in the phototrophic consortium “*Chlorochromatium aggregatum*.” *Genome Biol.* **14**, R127.
2. Rodionova, I. A., Li, X., Thiel, V., Stolyar, S., Stanton, K., Frederickson, J. K., **Bryant**, D. A., Osterman, A. L., Best A. A., and Rodionov, D. A. 2013. Comparative genomics and functional analysis of rhamnose catabolic pathways and regulons in Bacteria. *Front. Microbiol.* **4**, 407.
3. Niedzwiedzki, D. M., Orf, G. S., Tank, M., Vogl, K., **Bryant**, D. A., and Blankenship, R. E. 2014. Photophysical properties of the excited states of bacteriochlorophyll *f* in solvents and in chlorosomes. *J. Phys. Chem. Part B* **118**, 2295–2305.
4. Stolyar, S., Liu, Z., Thiel, V., Tomsho, L. P., Pinel, N., Nelson, W., Lindemann, S., Romine, M., Haruta, S., Schuster, S. C., **Bryant**, D. A. and Fredrickson, J. K. 2014. Genome sequence of the thermophilic cyanobacterium *Thermosynechococcus* sp. strain NK55a. *Genome Announc.* **2**, e01060-13.
5. **Bryant**, D. A. 2014. A brief history of cyanobacterial research: past, present and future prospects… In: *The Cell Biology of Cyanobacteria* (Herrero, A. and Flores, E., eds.), pp. 1–5. Caister Academic Press, Norfolk, United Kingdom.
6. Miloslavina, Y., Sankar Gupta, K. B. S., Tank, M., **Bryant**, D. A., and de Groot, H. J. M. 2014. wPMLG-5 spectroscopy of self-aggregated BChl *e* in natural chlorosomes of *Chlorobaculum limnaeum*. *Israel J. Chem.* **54**, 147–153.
7. Beliaev, A. S., Romine, M. F., Serres, M., Bernstein, H. C., Linggi, B. E., Markille, L. M., Isern,

N. G., Chrisler, W. B., Kucek, L. A., Hill, E. A., Pinchuk, G. E., **Bryant**, D. A., Wiley, H. S., Frederickson, J. K., and Konopka, A. 2014. Inference of interactions in cyanobacterial- heterotrophic co-cultures via transcriptome sequencing. *ISME J.* **8,** 2243–2255.

1. Hartzler, D., Niedzwiedzki, D. M., **Bryant**, D. A., Blankenship, R. E., Pushkar, Y., and Savikhin,

S. 2014. Triplet excited state energies and phosphorescence spectra of (bacterio)chlorophylls. *J. Phys. Chem. Part B* **118**, 7221–7232.

1. Hamilton, T. L., Bovee, R. J., Thiel, V., Sattin, S. R., Mohr, W., Schaperdoth, I., Gillhooly, W.

P. 3^rd^, Lyons, T. W., Tomsho, L. P., Schuster, S. C., Overmann, J., **Bryant**, D. A., Pearson, A., and Macalady, J. L. 2014. Coupled reductive and oxidative sulfur cycling in the phototrophic plate of a meromictic lake. *Geobiology* **12**, 451–468.

1. Zhang, S., Li, Z., Shen, G., Golbeck, J. H. and **Bryant**, D. A. 2014. Vipp1 in *Synechococcus* sp. PCC 7002 is not essential but is required for photosystem I assembly. *J. Biol. Chem.* **289**, 15904–15914.
2. Ludwig, M., Pandelia, M.-E., Chew, C. Y., Golbeck, J. H., Krebs, C., and **Bryant**, D. A. 2014. ChlR protein of *Synechococcus* sp. PCC 7002 is a transcription activator that uses an oxygen- sensitive [4Fe-4S] cluster to control genes involved in pigment biosynthesis. *J. Biol. Chem.* **289**, 16624–16639.
3. Sinninghe Damsté, J., Rijpstra, W. I. C., Hopmans, E. C., Foesel, B. U., Wüst, P. K., Overmann, J., Tank, M. **Bryant**, D. A., Dunfield, P. F., and Stott, M. B. 2014. Ether- and ester-bound *iso*- diabolic acid and other lipids in *Acidobacterium* of subdivision 4. *Appl. Environ. Microbiol.* **80**, 5207–5218.
4. Bernstein, H. C., Konopka, A., Melnicki, M. R., Hill, E. A., Kucek, L. A., Zhang, S., Shen, G., **Bryant**, D. A., and Beliaev, A. S. 2014. Effect of mono- and dichromatic light quality on growth rates and photosynthetic performance of *Synechococcus* sp. PCC 7002. *Front. Microbiol.* **5**, 488.
5. Gan, F., Zhang, S., Rockwell, N. C., Martin, S. S., Lagarias, J. C. and **Bryant**, D. A. 2014. Extensive remodeling of a cyanobacterial photosynthetic apparatus in far-red light. *Science* **345**, 1312–1317.
6. Shively, J. M., Cannon G. C., Heinhorst, S., Fuerst, J. A., **Bryant**, D. A., Maupin-Furlow, J. A., Schüler, D., Pfeifer, F., Docampo, R., Dahl, C., Preiss, J, Steinbüchel, A., and Federici, B. A. (2014). Intracellular structures of prokaryotes: inclusions, compartments, and assemblages. *Encyclopedia of Microbiology* (Schaechter, M., ed.), in press. Elsevier, Amsterdam, The Netherlands.
7. Therien, J. B., Zadvornyy, O., Posewitz, M. C., **Bryant**, D. A. and Peters, J. W. 2014. Growth of *Chlamydomonas reinhardtii* in acetate-free medium when co-cultured with alginate-encapsulated strains of *Synechococcus* sp. PCC 7002. *Biotech. Biofuels* **7**, 154.
8. Zhang, S. and **Bryant**, D. A. 2014. Learning new tricks from an old cycle: the TCA cycle of cyanobacteria, algae and plants. *Perspect. Phycol.* **1**, 73–86.
9. Thiel, V., Tomsho, L. P., Burhans, R., Gay, S. E., Ramaley, R. F., Schuster, S. C., Steinke, L., and **Bryant**, D. A. 2014. Draft genome sequence of the moderately thermophilic bacterium *Schleiferia thermophila* strain Yellowstone) (*Bacteroidetes*). *Genome Announc.* **2**, e00860-14.
10. Thiel, V., Tomsho, L. P., Burhans, R., Schuster, S. C., and **Bryant**, D. A. 2014. Draft genome of a sulfide-oxidizing, autotrophic *Chloroflexus* sp. strain MS-G (Yellowstone National Park, WY). *Genome Announc.* **2**, e00872-14.
11. Rockwell, N. C., Martin, S. S., Gan, F., **Bryant**, D. A., and Lagarias, J. C. 2015. NpR3784 is the prototype for a distinctive group of red/green cyanobacteriochromes using alternative Phe residues for photoproduct tuning. *Photochem. Photobiol. Sci.* **14**, 258–269.
12. Yang, M., Yang, Y., Chen, Z., Zhang, J., Lin, Y., Wang, Y., Xiong, Q., Li, T., Ge, F., **Bryant**, D.
    1. and Zhao, J. 2015. Proteogenomic analysis and global discovery of post-translational modifications in prokaryotes. *Proc. Natl. Acad. Sci. USA* **111**, E5633-42. doi: 10.1073/pnas.1412722111
13. Gan, F., Shen, G. and **Bryant**, D. A. 2015. Occurrence of far-red light photoacclimation (FaRLiP) in diverse cyanobacteria. *Life* (Basel) **5**, 4–24. doi: 10.3390/life5010004
14. Zhao, C., Li, Z., Li, T., Zhang, Y., **Bryant**, D. A. and Zhao, J. 2015. High-yield production of extracellular type-I cellulose by the cyanobacterium *Synechococcus* sp. PCC 7002. *Cell Discovery* **1**, 15004.
15. Tank, M. and **Bryant**, D. A. 2015. *Chloracidobacterium thermophilum* gen. nov., sp. nov.: an anoxygenic microaerophilic chlorophotoheterotrophic acidobacterium. *Int. J. Syst. Evol. Microbiol.* **65**, 1426–1430.
16. Tank, M. and **Bryant**, D. A. 2015. Nutrient requirements and growth physiology of the photoheterotrophic Acidobacterium, *Chloracidobacterium thermophilum*. *Front. Microbiol.* **6**, 226.
17. Kim, Y.-M., S., Olsen, M. T., Becraft, E. D., Thiel, V. **Bryant**, D. A., Fredrickson, J. K., Ward,

D. M. and Metz, T. O. 2015. Diel Metabolomics analysis of a Yellowstone National Park hot spring chlorophototrophic microbial community reveals *in situ* metabolisms of predominant mat inhabitants. *Front. Microbiol.* **6**, 209.

1. Becraft, E. D., Wood, J. M., Rusch, D. B., Kühl, M., Jensen, S. I., **Bryant**, D. A., Roberts, D. W., Cohan, F. M., and Ward, D. M. 2015. The molecular dimension of microbial species. 1. Ecological distinctions among, and homogeneity within, putative ecotypes of *Synechococcus* inhabiting the cyanobacterial mat of Mushroom Spring, Yellowstone National Park, *Front. Microbiol.* **6,** 590.
2. Nowack, S., Olsen, M. T., Schaible, G., Becraft, E. D., Shen, G., **Bryant**, D. A., Klapper, I. and Ward, D. M. 2015. The molecular dimension of microbial species. 2. *Synechococcus* strains representative of putative ecotypes inhabiting different depths in the Mushroom Spring microbial mat exhibit different adaptive and acclimative responses to light. *Front. Microbiol.* **6**, 626.
3. Olsen, M. T., Nowack, S., Wood, J. M., Becraft, E. D., LaButti, K., Lipzen, A., Martin, J., Schackwitz, W. S., Rusch, D. B., Cohan, F. M., **Bryant**, D. A., and Ward, D. M. 2015. The molecular dimension of microbial species. 3. Comparative genomics of *Synechococcus* strains with different light responses and *in situ* diel transcription patterns of associated putative ecotypes in the Mushroom Spring microbial mat. *Front. Microbiol.* **6**, 604.
4. Schieferdecker, S., Domin, N., Hoffmeier, C., **Bryant**, D. A., Roth, M. and Nett, M. 2015. Structure and absolute configuration of auriculamide, a natural product from the predatory bacterium *Herpetosiphon aurantiacus*. *Eur. J. Org. Chem.* **2015**, 3057–3062.
5. Jackson, S. A., Eaton-Rye, J. J., **Bryant**, D. A., Posewitz, M. C. and Davies, F. K. 2015. Absence of global nitrogen deprivation responses in the *Synechococcus* sp. PCC 7002 glycogen- deficient *ΔglgC* mutant. *Appl. Environ. Microbiol*. **81**, 6210–6222.
6. Thiel, V., Tomsho, L. P., Burhans, R., Schuster, S. C., and **Bryant**, D. A. 2015. Draft genome sequence of *Meiothermus ruber* strain A (*Deinococcus-Thermus*)). *Genome Announc.* **3**, e00202- 15.
7. Zhang, S. and **Bryant**, D. A. 2015. Biochemical validation of the glyoxylate cycle in the cyanobacterium *Chlorogloeopsis fritschii* strain PCC 9212. *J. Biol. Chem.* **290**, 14019–14030. doi: 10.1074/jbc.M115.648170
8. Gan, F., and **Bryant**, D. A. 2015. Adaptive and acclimative responses of the photosynthetic apparatus in cyanobacteria to far-red light. *Environ. Microbiol.* **17**, 3450–3465. doi: 10.1111/1462-2920.12992
9. Zhang, S., Liu, Y. and **Bryant**, D. A. 2015. Metabolic engineering of *Synechococcus* sp. PCC 7002 to produce poly-3-hydroxybutyrate and poly-3-hydroxybutyrate-co-4-hydroxybutryate. *Metab. Eng.* **32**, 174–183. doi: 10.1016/j.ymben.2015.10.001
10. Gorka, M., Pérez, A., Baker, C. S., Ferlez, B., van der Est, A., **Bryant**, D. A. and Golbeck, J. H. 2015. Electron transfer from the A1A and A1B sites to a tethered Pt nanoparticle requires FeS cluster for suppression of the recombination channel. *J. Photochem. Photobiol. B: Biology* **152** (Pt. B), 325–334. doi: 10.1016/j.jphotobiol.2015.08.015
11. Melendrez, M. C., Becraft, E., Wood, J. M., Olsen, M. T., **Bryant**, D. A., Heidelberg, J. F., Rusch, D., Cohan, F. M., and Ward, D. M. 2015. Recombination does not hinder formation or detection of ecological species of *Synechococcus* inhabiting a hot spring cyanobacterial mat. *Front. Microbiol.* **6**, 1540. doi: 10.3389/fmicb.2015.01540
12. Hamilton, T. L., **Bryant**, D. A. and Macalady, J. L. 2015. The role of biology in planetary evolution: cyanobacterial primary production in low oxygen Proterozoic oceans. *Environ. Microbiol.,* **18**, 325–340. doi: 10.1111/1462-2920
13. Ludwig, M., Chua, T. T., Chew, C. Y., **Bryant**, D. A. 2015. Fur-type transcription repressors and metal homeostasis in the cyanobacterium *Synechococcus* sp. PCC 7002. *Front. Microbiol.* **6**, 1217. doi: 10.3389/fmicb.2015.01217
14. Zhao, C., Gan, F., Shen, G. and **Bryant**, D. A. 2015. RfpA, RfpB, and RfpC are the master control elements for far-red light photoacclimation (FaRLiP). *Front. Microbiol.* **6**, 1303. doi: 10.3389/fmicb.2015.01303
15. Qian, X., Kumaraswamy, G. K., Zhang, S., Gates, C., Ananyev, G. M., **Bryant**, D. A., and Dismukes, G. C. 2016. Inactivation of nitrate reductase alters metabolic branching of carbohydrate fermentation in the cyanobacterium *Synechococcus* sp. PCC 7002. *Biotechnol. Bioeng.* **113**, 979–988. doi: 10.1002/bit.25862
16. Krishnan, A., Zhang, S., Liu, Y., **Bryant**, D. A. and Dismukes, C. G. 2016. Consequences of *ccmR* deletion on respiration, fermentation and H2 metabolism in cyanobacterium *Synechococcus* sp. PCC 7002. *Biotechnol. Bioeng.* **113**, 1448–1459. doi: 10.1002/bit.25913
17. Klotz, M. K., **Bryant**, D. A., Fredrickson, J. K., Inskeep, W. P. and Kühl, M. 2016. *Systems Biology and Ecology of Microbial Mat Communities. Front. Microbiol.,* e-book, 262 pp. doi: 10.3389/978-2-88919-793.4
18. Klotz, M. K., **Bryant**, D. A., Fredrickson, J. K., Inskeep, W. P. and Kühl, M. 2016. Editorial: Systems biology and ecology of microbial mat communities. *Front. Microbiol.* **7**, 115. doi: 10.3389/fmicb.2016.00115
19. Bernstein, H. C., McClure, R. S., Hill, E. A., Markillie, L. M., Romine, M. F., Posewitz, M. C., **Bryant**, D. A., Konopka, A., Fredrickson, J. K., and Beliaev, A. S. 2016. Unlocking the constraints of cyanobacterial productivity: adaptations enabling ultrafast growth. *mBio* **7**, e- 00949-16. doi: 10.1128/mBio.00949-16
20. Tsukatani, Y., Mizoguchi, T., Thweatt, J. L., Tank, M., **Bryant**, D. A. and Tamiaki, H. 2016. Glycolipid analyses of light-harvesting chlorosomes from envelope protein mutants of *Chlorobaculum tepidum*. *Photosynth. Res.* **128**, 235–241. doi: 10.1007/s11120-016-0228-z
21. McClure, R. S., Overall, C. C., McDermott, J. E., Hill, E., Markille, L. M., McCue, L. A., Taylor, R. C., Ludwig, M., **Bryant**, D. A., and Beliaev, A. S. 2016. Network analysis of transcriptomics expands regulatory landscapes in *Synechococcus* sp. PCC 7002. *Nucl. Acids Res.* **44**, 8810-8825. doi:10.1093/nar/gkw737.
22. Llorens-Marès, T., Liu, Z., Allen, L. Z., Rusch, D. B., Craig, M. T., Dupont, C. L., **Bryant**, D.
    1. and Casamayor, E. O. 2016. Speciation and ecological success by horizontal gene transfer in a green sulfur bacterial population: evidence for virus-mediated gene transmission. *ISME J.* **11***,* 201–211. doi:10.1038/ismej.2016.93
23. Günther, L., Jendrny, M., Bloemsma, E. A., Tank, M., Oostergetel, G. T., **Bryant**, D. A., Knoester, J. and Köhler, J. 2016. Structure of light-harvesting aggregates in individual chlorosomes. *J. Phys. Chem. B* **120**, 5367–5376. doi: 10.1021/acs.jpcb.6b03718
24. Xia, S., Cartron, M., Morby, J., **Bryant**, D. A., Hunter, C. N., and Leggett, G. J. 2016. Fabrication of nanometer and micrometer scale protein structures by site-specific immobilization of histidine-tagged proteins to aminosiloxane films with photoremovable protein-resistant protecting groups. *Langmuir* **32**, 1818–1827. doi: 10.1021/acs.langmuir.5b04368
25. Pérez, A. A., Liu, Z., Rodionov, D. A., Li, Z., and **Bryant**, D. A. 2016. Complementation of cobalamin auxotrophy in *Synechococcus* sp. PCC 7002 and validation of a putative cobalamin riboswitch *in vivo*. *J. Bacteriol.* **198**, 2743–2752. doi: 10.1128/JB.00475-16.
26. Pérez, A. A., Rodionov, D. A., and **Bryant**, D. A. 2016. Identification and regulation of genes for cobalamin transport in the cyanobacterium *Synechococcus* sp. PCC 7002. *J. Bacteriol.* **198**, 2753–2761. doi: 10.1128/JB.00476-16.
27. Shen, G., Gan, F., and **Bryant**, D. A. 2016. The siderophilic cyanobacterium *Leptolyngbya* sp. strain JSC-1 acclimates to iron starvation by expressing multiple *isiA*-family genes. *Photosynth. Res.* **128**, 325–340. doi: 10.1007/s11120-016-0257-7.
28. Thiel, V., Wood, J. M., Olsen, M. T., Ward, D. M., and **Bryant**, D. A. 2016. The dark side of the Mushroom Spring microbial mat: life in the shadow of chlorophototrophs. I: Microbial diversity based on 16S rRNA gene amplicons and metagenomic sequencing. *Front. Microbiol*. **7**, 919. doi: 10.3389/fmicb.2016.00919.
29. Ho, M.-Y., Shen, G., Canniffe, D. P., Zhao, C., and **Bryant**, D. A. 2016. Light-dependent chlorophyll *f* synthase is a highly divergent paralog of PsbA of Photosystem II. *Science* **353**, aaf9178. doi: 10.1126/science.aaf9178.
30. Zhang, S., Qian, X., Chang, S., Dismukes, G. C. and **Bryant**, D. A. 2016. Reconstructing the tricarboxylic acid cycle in a cyanobacterium: introduction of the GABA shunt into the cyanobacterium *Synechococcus* sp. PCC 7002. *Front. Microbiol.* **7***,* 1972*.* doi: 10.3389/fmicb.2016.01972
31. **Bryant**, D. A. (2016) Prospects for enhancing plant and algal biofuel production by expanding the wavelength range for photosynthesis. *Information Systems for Biotechnology News Report*, September 2016, pp. 1–6.
32. Nabhan, S., Bunk, B., Spörer C., Liu, Z., **Bryant**, D. A., and Overmann, J. 2016. Genome sequence of *Prosthecochloris phaeum* CIB2401 of the phylum *Chlorobi. Genome Announc*. **4**, e01222-16. doi: 10.1128/genomeA.01222-16
33. Fushimi, K., Rockwell, N. C., Enomoto, G., Win, N.-N., Martin, S. S., Gan, F., **Bryant**, D. A.,

M. Ikeuchi, Lagarias, J. C., and Narikawa, R. 2016. Cyanobacteriochrome photoreceptors lacking the canonical Cys residue. *Biochemistry* **55**, 6981–6995. doi: 10.1021/acs.biochem.6b00940.

1. Pérez, A. A., Gajewski, J. P., Ferlez, B. H., Ludwig, M., Baker, C. S., Golbeck, J. H., and **Bryant**, D. A. 2016. A Zn^++^-inducible expression platform for *Synechococcus* sp. strain PCC 7002 based on the *smtA* promoter/operator and SmtB repressor. *Appl. Environ. Microbiol.* 83, e02491-16. doi: 610.1128/AEM.02491-1. (Included the cover image for February 2017 (Part 3) of this journal.).
2. Tank, M., Thiel, V. Ward, D. M. and **Bryant**, D. A. 2017. A panoply of phototrophs: an overview of chlorophototrophs found in the microbial mats of alkaline siliceous hot springs in Yellowstone National Park, WY, USA. In: *“Modern Topics in the Phototrophic Prokaryotes: Environmental and Applied Aspects*,” (Hallenbeck, P. C., ed.), Springer, Berlin, Germany, pp. 87–137. doi: 10.1007/978-3-319-46261-5 (ISBN: 978-3-319-46259-2).
3. Ho, M.-Y., Gan, F., Shen, G., Zhao, C., and **Bryant**, D. A. 2017. Far-red light photoacclimation (FaRLiP) in *Synechococcus* sp. PCC 7335. I. Regulation of FaRLiP gene expression. *Photosynth. Res.* **131**, 173–186. doi: 10.1007/s11120-016-0309-z.
4. Ho, M.-Y., Gan, F., Shen, G., and **Bryant**, D. A. 2017. Far-red light photoacclimation (FaRLiP) in *Synechococcus* sp. PCC 7335. II. Characterization of phycobiliproteins produced during acclimation to far-red light. *Photosynth. Res.* **131**, 187–202. doi: 10.1007/s11120-016-0303-5.
5. Xiong, W., Shen, G. and **Bryant**, D. A. 2017. *Synechocystis* sp. PCC 6803 CruA (sll0147) encodes lycopene cyclase and requires bound chlorophyll *a* for activity. *Photosynth. Res.* **131**, 267–280. doi: 10.1007/s11120-016-0316-0.
6. Thweatt, J. L., Ferlez, B. H., Golbeck, J. H., and **Bryant**, D. A. 2017. BciD is a radical-S- adenosyl-L-methionine (SAM) enzyme that completes bacteriochlorophyllide *e* biosynthesis by oxidizing a methyl group into a formyl group at C-7. *J. Biol. Chem.* **292**, 1361–1373. doi: 10.1074/jbc.M116.767665.
7. Bernstein, H. C., McClure, R. S., Thiel, V., Sadler, N. C., Kim, Y.-M., Chrisler, W. B., Hill, E. A., **Bryant**, D. A., Romine, M. F., Jansson, J. K., Fredrickson, J. K., and Beliaev, A. S. 2017. Indirect interspecies regulation: transcriptional and physiological responses of a cyanobacterium to a heterotrophic partnership. *mSystems* 2, e00181-16. doi: 10.1128/mSystems.00181-16.
8. Mancini, J. A., Kodali, G., Jiang J., Reddy, K. R., Lindsey, J. S., **Bryant**, D. A., Dutton, P. L., and Moser, C. C. 2017. Multistep excitation energy transfer engineered in genetic fusions of natural and synthetic light-harvesting proteins. *J. R. Soc. Interface* **14**, 20160896. doi: 10.1098/rsif.2016.0896
9. Ho, M.-Y., Soulier, N. T., Canniffe, D. P., Shen, G. and **Bryant**, D. A., 2017. Light regulation of cyanobacterial pigment and photosystem biosynthesis. *Curr. Opin. Plant Biol.* **37**, 24-33. doi: 10.1016/j.pbi.2017.03.006
10. Thiel, V., Hügler, M., Ward, D. M., and **Bryant**, D. A. 2017. The dark side of the Mushroom Spring microbial mat: life in the shadow of chlorophototrophs. II. Metabolic functions of abundant community members predicted from metagenomic analyses. *Front. Microbiol.* **8**, 943*.* doi: 10.3389/fmicb.2017.00943
11. Tank, M., Liu, Z., Frigaard, N.-U., Thomsho, L. P., Schuster, S. C., and **Bryant**, D. A. 2017. Complete genome sequence of a bacteriochlorophyll *e*-producing green sulfur bacterium, *Chlorobaculum limnaeum* DSM strain 1677^T^. *Genome Announc.* **5**, e00529-17. doi: 10.1128/genomeA.00529-17
12. Thiel, V., Drautz-Moses, D. I., Schuster, S. C., Lindemann, S. and **Bryant**, D. A. 2017. Genome sequence of *Prosthecochloris* sp. strain HL-130-GSB (*Chlorobi*). *Genome Announc.* **5**, e00538-

17. doi: 10.1128/genomeA.00538-17

1. Thiel, V., Tomsho, L. P., Burhans, R., Gay, S. E., Tank, M., Hamilton, T. L., S. C. Schuster, S. C., and **Bryant**, D. A. 2017. Draft genome sequence of *Anoxybacillus ayderensis* strain MT-Cab (*Firmicutes*). *Genome Announc.,* **5**, e00547-17. doi: 10.1128/genomeA.00547-17
2. Therien, J. B., Artz, J. H., Poudel, S., Hamilton, T. L., Liu, Z., Noone, S. M., Adams, M. W. W., King, P. W., **Bryant**, D. A., Boyd, E. S., and Peters, J. W. 2017. The physiological functions and structural determinants of catalytic bias in the [FeFe]-hydrogenases of *Clostridium pasteurianum* W5. *Front. Microbiol.* **8***,* 1305. doi: 10.3389/ficb.2017.01305
3. Orf, G. S., Collins, A. M., Niedzwiedzki, D. M., Tank, M., Thiel, V., Kell, A., **Bryant**, D. A., Montaño, G. A., and Blankenship, R. E. 2017. Polymer-chlorosome nanocomposites consisting of non-native combinations of self-assemblying bacteriochlorophylls. *Langmuir* **33**, 6427–6438. doi: 10.1021/acs.langmuir.7b01761
4. Junqueira, A. C. M., Ratan, A., Acerbi, E., Drautz-Moses, D. I., Premkrishnan, B. N. V., Costea, P., Linz, B., Purbojati R. W., Paulo, D. F., Gaultier, N. E., Subramanian, P., Hasan, N, Colwell, R., Bork, P., Azeredo-Espin, A. M. L., **Bryant**, D. A., and Schuster, S. C. 2017. The microbiomes of blowflies and houseflies as bacterial transmission reserviors. *Sci. Rep*. **7**, 16324. doi: 10.1038/s41598-017-16353-x
5. Barnett, S. F. H., Hitchcock, A., Vasilev, C., Mandal, A. K., Yuen, J. M., Morby, J., Brindley A. A., Holten, Niedzwiedzki, D. M., Holten, D., **Bryant**, D. A., Cadby, A. J., and Hunter, C. N. 2017. Repurposing a photosynthetic antenna protein as a super-resolution microscopy label. *Sci. Rep.* **7**, 16807. doi: 10.1038/s41598-017-16834-z
6. Pérez, A. A., Ferlez, B. H., Applegate, A. M., Walters, K., He, Z., Shen, G., Golbeck, J. H., and **Bryant**, D. A. 2018. Presence of a [3Fe-4S] cluster in a PsaC variant as a functional component of the photosystem I electron transfer chain in *Synechococcus* sp. strain PCC 7002. *Photosynth. Res.* 136, 31–48. doi: 10.1007/s11120-017-0437-0
7. Martin, W. F., **Bryant**, D. A. and Beatty, J. T. 2018. A physiological perspective on the origin and evolution of photosynthesis. *FEMS Microbiol. Rev.* **42**, 201–231. doi: 10.1093/femsre/fux056
8. **Bryant**, D. A., and Canniffe, D. P. 2018. How Nature designs antenna proteins: design principles and functional realization of light-harvesting antenna systems in chlorophototrophic prokaryotes.

*J. Phys. B: At. Mol. Opt. Phys.* **51**, 033001. doi: 10.1088/1361-6455/aa9c3c

1. Chen, G. E., Canniffe, D. P, Barnett, S. F. H., Hollingshead, S., Brindley A. A., Vasilev, C., **Bryant**, D. A. Hunter, C. N. 2018. Complete enzyme set for chlorophyll biosynthesis in *Escherichia coli. Sci. Adv.* **4**, eaaq1407. doi: 10.1126/sciadv.aaq1407.
2. Thiel, V., Tank, M. and **Bryant**, D. A. 2018. Diversity of chlorophototrophic bacteria revealed in the omics era. *Annu. Rev. Plant Biol.* **69**, 21–49. doi: 10.1146/annurev-arplant-042817-040500
3. **Bryant**, D. A. 2019. Phototrophy and phototrophs. In: *Encyclopedia of Microbiology*, 4^th^ edition, Schmidt, T. editor-in-chief. Elsevier, Amsterdam, The Netherlands, pp. 527–537. doi.org/10.1016/B978-0-12-809633-8.20672-9
4. Mancini, J. A., Sheehan, M., Kodali, G., Chow, B. Y., **Bryant**, D. A., Dutton, P. L., and Moser,

C. C. 2018. *De novo* synthetic biliprotein design, assembly, and excitation energy transfer. *J. R. Soc. Interface* 20180021. doi: 10.1098/rsif.2018.0021

1. Ortega-Ramos, M., Canniffe, D. P., Radle, M. I., Hunter, C. N., **Bryant**, D. A., Golbeck, J. H. 2018. Engineered biosynthesis of bacteriochlorophyll *g*F in *Rhodobacter sphaeroides*. *Biochim. Biophys. Acta* **1859**, 501–509. doi: 10.1016/j.bbabio.2018.02.006
2. Zill, J. C., He, Z., Tank, M., Canniffe, D. P., Ferlez, B. H., Lahav, Y., Bellstedt, P., Alia, A., Schapiro, I., Golbeck, J. H., **Bryant**, D. A. and Matysik, J. 2018. ^15^N-Photo-CIDNP MAS NMR analysis of reaction centers of *Chloracidobacterium thermophilu*m. *Photosynth. Res.* **137**, 295–

305. doi: 10.1007/s11120-018-0504-1

1. Blain-Hartung, M., Rockwell, N. C., Moreno, M. V., Martin, S. S., Gan, F., **Bryant**, D. A., and Lagarias, J. C. 2018. Cyanobacteriochrome-based photoswitchable adenylyl cyclases (cPACs) for broad spectrum light regulation of cAMP levels in cells. *J. Biol. Chem.* **293**, 8473–8483. doi: 10.1074/jbc.RA118.002258
2. Günther, L. M., Löhner, A., Reiher, C., Kunsel, T., Jansen, T. L. C., Tank, M., **Bryant**, D. A., Knoester, J., and Köhler, J. 2018. Structural variations in chlorosomes from wild-type and a *bchQR* mutant of *Chlorobaculum tepidum* revealed by single-molecule spectroscopy. *J. Phys. Chem. Part B*, **122**, 6712–6723. doi: 10.1021/acs.jpcb.8b02875
3. Cherepanov, D. A., Milanovsky, G. E., Gopta, O. A., Balasubramanian, R., **Bryant**, D. A., Semenov, A. Yu., and Golbeck, J. H. 2018. Electron-phonon coupling in cyanobacterial photosystem I. *J. Phys. Chem. Part B*, **122**, 7943–7955. doi: 10.1021/acs.jpcb.8b03906
4. Canniffe, D. P., Thweatt, J. L., Gomez Maqueo Chew, A., Hunter, C. N. and **Bryant**, D. A. 2018. A paralog of phytol reductase catalyzes the formation of 1,2-dihydro-carotenoids in green sulfur bacteria. *J. Biol. Chem.* **293**, 15233–15242. doi:10.1074/jbc.RA118.004672
5. Tank, M., Garcia Costas, A. M., and **Bryant**, D. A. 2018. Genus: *Chloracidobacterium*. In: *Bergey’s Manual of Systematics of Bacteria and Archaea* (W. B. Whitman, supervising editor), John Wiley & Sons, New York, New York, USA. doi: 10.1002/9781118960608.
6. Swainsbury, D. K., Faries, K. M., Niedzwiedzki, D. M., Martin, E. C., Finders, A. J., Canniffe,

D. P., Shen, G., **Bryant**, D. A., Kirmaier, C., Holten, D. and Hunter, C. N. 2019. Engineering of B800 bacteriochlorphyll binding site specificity in *Rhodobacter sphaeroide*s LH2. *Biochim*. *Biophys. Acta* **1860**, 209-223. doi: 10.1016/j.bbabio.2018.11.008

1. Thiel, V., Garcia Costas, A. M., Fortney, N. W. W., Martinez, J. N., Roden, E. E., Boyd, E. S., Ward, D. M. and **Bryant**, D. A. 2019. “*Candidatus* Thermonerobacter thiotrophicus,” a non- phototrophic, sulfate-reducing member of the phylum *Chlorobi* that inhabits hot-spring communities. *Front. Microbiol.* **9**, 3159. doi: 10.3389/fmicb.2018.03159
2. Shen, G., Canniffe, D. P., Kurashov, V., Ho, M.-Y., van der Est, A., Golbeck, J. H. and **Bryant**,

D. A. 2019. Improved heterologous expression of chlorophyll *f* synthase in *Synechococcus* sp. PCC 7002: isolation and initial characterization. *Photosynth. Res.* **140**, 77-92. doi: 10.1007/s11120-018-00610-9

1. Kurashov, V., Ho, M.-Y., Shen, G., Piedl, K., Laremore, T. N., **Bryant**, D. A. and Golbeck, J. H. 2019. Energy transfer from chlorophyll *f* to the trapping center in naturally occurring and engineered Photosystem I complexes. *Photosynth. Res*. **141**, 151-163. doi: 10.1007/s11120-019-

006. 16-x

1. Kumaraswamy, G. K., Krishnan, A., Ananyev, G., Zhang, S., **Bryant**, D. A., Dismukes, G. C. 2019. Crossing the Thauer limit: rewiring cyanobacterial metabolism to maximize fermentative H2 production. *Energy Environ. Sci*. **12**, 1035–1045. doi: 10.1039/c8ee03606c.
2. Ho, M.-Y., **Bryant**, D. A. 2019. Global transcription profiling of the cyanobacterium *Chlorogloeopsis fritschii* PCC 9212 in far-red light: insights into the regulation of chlorophyll *d* biosynthesis. *Front. Microbiol.* **10**, 465. doi: 10.3389/fmicb.2019.00465
3. Thweatt, J. L., Canniffe, D. P., and **Bryant**, D. A. 2019. Biosynthesis of chlorophylls and bacteriochlorophylls in green bacteria. Grimm, B., ed., *Advances in Botanical Research*, Vol. **90**,

pp. 35-89. *Metabolism, structure and function of chlorophylls*, B. Grimm, ed. Elsevier, Amsterdam, The Neterlands. Invited review. doi: 10.1016/bs.abr.2019.03.002

1. Hastings, G., Makita, H., Agarwala, N., Rohani, L., Shen, G. and **Bryant**, D. A. 2019. Fourier- transform visible and infrared difference spectroscopy for the study of P700 in photosystem I of *Fischerella thermalis* PCC 7521 cells grown under white light and far-red light: evidence that cofactor A–1 is chlorophyll *f. Biochim. Biophys. Acta* **1860**, 452–460. doi: 10.1016/j.bbabio.2019.04.002
2. He, Z., Kurashov, V., Ferlez, B., Tank, M., Golbeck, J. H., and **Bryant**, D. A. 2019. Homodimeric type-1 reaction centers of *Chloracidobacterium thermophilum* (*Acidobacteria*): I. Biochemical and biophysical characterization. *Photosynth. Res.* **142**, 87-103. doi: 10.1007/s11120-019-00650-9
3. Ho, M.-Y., Niedzwiedzki, D. M., MacGregor-Chatwin C., Gerstenecker, G., Hunter, C. N., Blankenship, R. E., and **Bryant**, D. A. 2020. Extensive remodeling of the photosynthetic apparatus alters energy transfer among photosynthetic complexes when cyanobacteria acclimate to far-red light. *Biochim. Biophys. Acta*—*Bioenerg.* **1861**, 148064. doi: 10.1016/j.bbabio.2019.148064
4. Gisriel, C., Shen, G., Kurashov, V., Ho, M.-Y., Zhang, S., Williams, D., Golbeck, J. H., Fromme, P., **Bryant**, D. A. 2020. The structure of Photosystem I acclimated to far-red light illuminates an ecologically important acclimation process in photosynthesis. *Science Adv.* **6**, eaay6415. doi: 10.1126/sciadv.aay6415
5. **Bryant**, D. A., Shen, G., Turner, G. M., Soulier, N., Laremore, T. N., and Ho, M.-Y. 2020. Far- red-light allophycocyanin subunits play a role in chlorophyll *d* accumulation in far-red light. *Photosynth. Res.* **143**, 81-95. doi: 10.1007/s11120-019-00689-8
6. Steinke, L., Slysz, G. W., Lipton, M. S., Klatt, C., Moran, J. J., Romine, M. F., Wood, J. M., Anderson, G., **Bryant**, D. A. and Ward, D. M. 2020. Short-term stable isotope probing of

proteins reveals taxa incorporating inorganic carbon in a hot spring microbial mat. *Appl. Environ. Microbiol.* **86**, e01829-19. doi: 10.1128/AEM.01829-19

1. Charles, P., Kalendra, V., He, Z, Khatima, M. H., Golbeck, J. H., van der Est, A., Lakshmi, K. V., and **Bryant**, D. A. 2020. Two-dimensional ^67^Zn HYSCORE spectroscopy reveals that a Zn- bacteriochlorophyll *a*P' dimer is the primary donor (P840) in the type-1 reaction centers of *Chloracidobacterium thermophilum. Phys. Chem. Chem. Phys*. **22**, 6457–6467. doi: 10.1039/c9cp06556c
2. Cherepanov, D. A., Shelaev, I. V., Gostev, F. E., Aybush, A. V., Mamedov, M. D., Shen, G., Nadtochenko, V. A., **Bryant**, D. A., Semenov, A. Yu., and Golbeck, J. H. 2020. Evidence that chlorophyll *f* functions solely as an antenna pigment in far-red-light photosystem I from *Fischerella thermalis* PCC 7521. *Biochim. Biophys. Acta*—*Bioenerg.* **1861**, 148184. doi: 10.1016/j.bbabio.2020.148184
3. Nayfach, S., Roux, S., Seshadri R., Udwary, D., Varghese N., Schulz, F., Wu, D., Paez-Espino, D., Chen, I.-M., Hutemann, M., Palaniappan, K., Ladau, J., Mukherjee, S, Reddy, T. B. K., Nielsen, T., Kirton, E., Faria, J. P., Edirisinghe, J. N., Henry C. S., Junbluth, S. P., Chivian, D., Dehal, P., Wood-Charlson, E. M., Arkin, A. P., Tringe, S., Visel, A., IMG/M Data Consortium (including **Bryant**, D. A.), Woyke, T., Mouncey, N. J., Ivanova, N. N., Kyrpides, N. C., and Eloe-Fadrosh, E. A. 2020. A genomic catalog of Earth’s microbiomes. *Nat. Biotech*. **39**, 499–

509. doi: 10.1038/s41587-020-0718-6

1. **Bryant**, D. A., Hunter, C. N., and Warren, M. J. 2020. Biosynthesis of the modified tetrapyrroles—the pigments of life. *J. Biol. Chem.* **295**, 6888–6925. doi: 10.1074/jbc.REV120.006194
2. Tros, M., Bersanini, L., Shen, G., Ho, M.-Y., van Stokkum, I. H. M., **Bryant**, D. A. and Croce,

R. 2020. Harvesting far-red light: functional integration of chlorophyll *f* into Photosystem I complexes of *Synechococcus* sp. PCC 7002. *Biochim. Biophys. Acta–Bioenerg.* **1861**, 148206. doi:10.1016/j.bbabio.2020.148206

1. Gisriel, C. J., Wang, J., Brudvig, G. W., and **Bryant**, D. A. 2020. Opportunities and challenges for assigning cofactors in cryo-EM density maps of chlorophyll-containing proteins. *Commun. Biol.* **3**, 408. Doi: 10.1038/s42003-020-01139-1
2. Saini, M. K., Weng, C.-C., Soulier, N., Sebastian, A., Albert, I., Thiel, V., **Bryant**, D. A. Hanada, S., and Tank, M. 2020. *Caldichromatium japonicum* gen. nov., sp. nov., a novel themophilic photosynthetic purple sulfur bacterium of the *Chromatiaceae* isolated from Nakabusa hot springs, Japan. *Int. J. Syst. Evol. Microbiol.* **70**, 5701–5710. doi: 10.1099/ijsem.0.004465
3. Cui, X., Liu, X-L., Shen, G., Ma, J., Husain, F., Rocher, D., Zumberge, J. E., **Bryant**, D. A., and Summons, R. E. 2020. Niche expansion for phototrophic sulfur bacteria at the Proterozoic- Phanerozoic transition. *Proc. Natl. Acad. Sci. USA*, **117**, 17599–17606. doi: 10.1073/pnas.2006379117.
4. Soulier, N. T., Laremore, T. N., and **Bryant**, D. A. 2020. Characterization of cyanobacterial allophycocyanins absorbing far-red light. *Photosynth. Res.* **145***,* 189-207. doi: 10.1007/s11120- 020-00775-2
5. Tros, M., Mascoli, V., Shen, G. Ho, M.-Y., Bersanini, L., Gisriel, C. J., **Bryant**, D. A., and Croce, R. 2020. Breaking the red-limit: efficient trapping of long-wavelength excitations in chlorophyll *f*-containing Photosystem I. *CHEM***,** 155-173. doi: 10.1016/j.chempr.2020.10.024
6. Ho, M.-Y., and **Bryant**, D. A. 2021. Long-wavelength pigments in photosynthesis. In: Jez Joseph (ed.), *Encyclopedia of Biological Chemistry*, 3rd Edition. vol. 2, pp. 245–255. Oxford: Elsevier, Amsterdam, The Netherlands. doi: 10.1016/B978-0-12-819460-7.00009-8
7. Soulier, N. T., Laremore, T. N., and **Bryant**, D. A. 2021. Correction to: Characterization of cyanobacterial allophycocyanins absorbing far-red light. *Photosynth. Res.* **147**, 239-240. doi: 10.1007/s11120-020-00790-3
8. Soulier, N. T., and **Bryant**, D. A. 2021. The structural basis of far-red light absorbance by allophycocyanins. *Photosynth. Res.* **147**, 11-26. doi: 10.1007/s11120-020-00787-y
9. Berg, M., Goudeau, D., Olmsted, C., McMahon, K. D., Thweatt, J., **Bryant**, D. A., Eloe- Fadrosh, E. A., Malmstrom, R. R., and Roux, S. 2021. Host population diversity as a driver of viral infection cycle in wild populations of green sulfur bacteria with long-standing virus-host interactions. *ISME J.* **15**, 1669–1684. doi: 10.1038/s41396-020-00870-1
10. Ma, J., French, K. L., Cui, X., **Bryant**, D. A., and Summons, R. E. 2021. Carotenoid biomarkers from Namibian shelf sediments: anoxygenic photosynthesis during sulfide eruptions in the Benguela Upwelling System. *Proc. Natl. Acad. Sci. USA* **118**, e2106040118. doi: 10.1073/pnas.2106040118
11. Gisriel, C. J., Huang, H.-L., Reiss, K., Flesher, D. A., Batista, V. S., **Bryant**, D. A., and Brudvig,

G. W., and Wang, J. 2021. Quantitive assessment of chlorophyll types in cryo-EM maps of photosystem I acclimated to far-red light. *Biochim. Biophys. Acta–Advances* **1**, 100019. doi: 10.1016/j.bbadva.2021.100019

1. Saini, M. K., Sebastian, A., Siratori, Y., Soulier, N. T., Garcia Costas, A. M., Drautz-Moses, D. I., Schuster, S. C., Albert, I., Haruta, S., Hanada, S., Thiel, V., Tank, M., and **Bryant**, D. A. 2021. Genomic and phenotypic characterization of *Chloracidobacterium* species isolates provides evidence for multiple species. *Front. Microbiol*. (*Evol. Genom. Microbiol.*) **12**, 704168. doi: 10.3389/fmicb.2021.704168
2. MacGregor-Chatwin, C., Nurnberg, D. J., Jackson, P. J., Vasilev, C., Andrew Hitchcock, A., Ho, M.-Y., Shen, G., Gisriel, C. J., Wood, W. H. J., Mahbub, M., Selinger, V. M., Johnson, M. P., Dickman, M. J., Rutherford, A. W., **Bryant**, D. A., and Hunter. C. N. 2022. Changes in supramolecular organization of cyanobacterial thylakoid membrane complexes in response to far-red light photoacclimation. *Science Adv.* **8**, eabj4437. doi: 10.1126/sciadv.abj4437
3. Gisriel, C. J., Flesher, D. A., Shen, G., Wang, J., Ho, M.-Y., Brudvig, G. W., and **Bryant**, D. A. 2022. Structure of a photosystem I-ferredoxin complex from a marine cyanobacterium provides insights into far-red light photoacclimation. *J. Biol. Chem.* **298**, 101408. doi: 10.1016/jbc.2021.101408
4. Gisriel, C. J., Shen, G., Ho, M.-Y., Kurashov, V., Flesher, D. A., Wang, J., Armstrong, W. H.,

Golbeck, J. H., Gunner, M. R., Vinyard, D. J., Debus, R. J., Brudvig, G. W., and **Bryant**, D. A. 2022. Structure of a monomeric photosystem II core complex from a cyanobacterium acclimated to far-red light reveals the functions of chlorophylls *d* and *f*. *J. Biol. Chem.* **298**, 101424. doi: 10.1016/j.jbc.2021.101424

1. Fremin, B. J., Global Phage Small Open Reading Frame (GP-SmORF) Consortium (including **Bryant**, D. A.), Bhatt, A. S., and Kyrpides, N. 2022. Revealing thousands of small, novel genes in global phage genomes. *Cell Rep*. 39, 110984. doi: 10.1016/j.celrep.2022.110984
2. Saini, M. K., Yoshida, S., Sebastian, A., Hirose, S., Hara, E., Tamaki, H. Soulier, N. T., Albert, I., Hanada, S., Tank, M., and **Bryant**, D. A. 2022. *Elioraea tepida,* sp. nov., a moderately thermophilic aerobic anoxygenic phototrophic bacterium isolated from the mat community of an alkaline siliceous hot spring in Yellowstone National Park, WY, USA. *Microorganisms* **10***,* 80. doi: 10.3390/microorganisms10010080
3. Soulier, N. T., Walters, K., Laremore, T. N., Shen, G., Golbeck, J. H. and **Bryant**, D. A. 2022. Acclimation of the photosynthetic apparatus to low light in a thermophilic *Synechococcus* sp. *Photosynth. Res.* **153**, 21–42. doi: 10.1007/s11120-022-00918-7
4. Neri, U., Wolf, Y. I., Roux, S., Camargo, A. P., Lee, B. D., Kazlauskas, D., Chen, I. M., Ivanova, N., Allen, L. Z., Paez-Espino, D., **Bryant**, D. A., Bhaya, D., RNA Virus Discovery Consortium, Krupovic, M., Dolja, V. V., Kyrpides, N. C., Koonin, E. V., and Gophna, U. 2022. Expansion of the global RNA virome reveals diverse clades of bacteriophages. *Cell* **185**, 4023–4037. doi: 10.1016/j.cell.2022.08.023
5. Nien, T.-S., **Bryant**, D. A., Ho, M.-Y. 2022. Use of quartz sand columns to study far-red light photoacclimation (FaRLiP) in cyanobacteria. *Appl. Environ. Microbiol.* **88**, 005620-22. doi: 10.1128/aem.00562-22
6. Murray, B., Ertekin, E., Dailey, M., Soulier, N. T., Shen, G., **Bryant**, D. A., Perez-Fernandez, C., DiRuggiero, J. 2022. Acclimation of cyanobacteria to the endolithic light spectrum in hyper- arid deserts. *Microorganisms* **10**, 1198. doi: 10.3390/microorganisms10061198
7. Gisriel, C. J., Cardona, T., **Bryant**, D. A., and Brudvig, G. W. 2022. Molecular evolution of far- red light-acclimated photosystem II. *Microorganisms* **10**, 1270. doi: 10.3390/microorganisms10071270
8. Soulier, N. T. and **Bryant**, D. A. 2023. Long-wavelength phycobiliproteins. In: *Photosynthesis: From Plants to Nanomaterials*, Hou, H. J. M. and Allakhverdiev, S. I., Eds. Academic Press, Elsevier, Amsterdam, The Netherlands, pp. 9-32.
9. Gisriel, C. J., Shen, G., Flesher, D. A., Kurashov, V., Golbeck, J. H., Brudvig, G. W., Amin, M., and **Bryant**, D. A. 2023. Structure of a dimeric photosystem II complex from a cyanobacterium acclimated to far-red light. *J. Biol. Chem.* **299**, 102815. doi: 0.1016/j.jbc.2022.102815
10. Kurihara, M., Thiel, V., Takahashi, H., Kojima, K., Ward, D. M., **Bryant**, D. A., Sakai, M., Yoshizawa, S., and Sudo Y. 2023. Identification of a functionally efficient and thermally stable outward sodium-pumping rhodopsin (BeNaR) from a thermophilic bacterium. *Chem. Pharm. Bull.* **71**, 154-164. doi: 10.1248/cpb.c22-00774
11. Gisriel, C. J., Elias, E., Shen, G., Soulier, N. T., Yang, K. R., Flesher, D. A., Batista, V. S., Gunner, M. R., Brudvig, G. W., Croce, R., and **Bryant**, D. A. 2023. Helical allophycocyanin nanotubes absorb far-red light in a thermophilic cyanobacterium. *Sci. Adv.* **9**, eadg0251. doi: 10.1126/sciadv.adg0251
12. Liu, T.-S., Wu, K.-F., Jiang, H.-W., Chen, K.-W., Nien, T.-S., **Bryant**, D A., and Ho, M.-Y.

2023. Investigating far-red light-inducuble promoters in regulating gene expression in cyanobacteria. *ACS Synth. Biol.* **12**, 1320-1330. doi: 10.1021/acssynbio.3c00066.

1. Pavlopoulos, G. A., Baltoumas, F. A., Liu, S., Selvitopi, O., Camargo, A. P., Nayfach, S., Azad, A., Roux, S., Call, L., Ivanova, N. N., Chen, I. M., Paez-Espino, D., Karatzas, E., Novel Metagenome Protein Families Consortium (including **Bryant**, D. A.), Iliopolous, I., Konstantinidis, K., Tiedje, Pett-Ridge, J., J. M., Baker, D., Visel, A., Ouzounis, C. A., Ovchinnikov, S., Buluç, A., and Kyrpides, N. C. 2023. Unraveling the functional dark matter through global metagenomics. *Nature* **622**, 594–602. doi: 10.1038/s41586-023-06583-7
2. Agostini, A., Shen, G., **Bryant**, D. A., Golbeck, J. H., van der Est, A., Carbonera, D. 2023. Optically detected magnetic resonance and mutational analysis reveal significant differences in the photochemistry and structure of chlorophyll *f* synthase and photosystem II. *Biochim. Biophys. Acta Bioenerg.* **1864**, 149002. doi: 10.1016/j.bbabio.2023.149002
3. Gisriel, C. J., Flesher, D. A., Long, Z., Liu, J., Wang, J., **Bryant**, D. A., Batista, V. S., and Brudwig, G. W. 2023. A quantitative assessment of (bacterio)chlorophyll assignments in the cryoEM structure of the *Chloracidobacterium thermophilum* reaction center. *Photosynth. Res.* **162**, 187-196. doi: 10.1007/s11120-023-01047-5
4. Gisriel, C. J., Elias, E., Shen, G., Soulier, N. T., Brudwig, G. W., Croce, R., and **Bryant**, D. A. 2023. Structural comparison of allophycocyanin variants reveals the molecular basis for their spectral differences. *Photosynth. Res*. **157**, doi: 10.1007/s11120-023-01048-4
5. Jiang, H.-W., Wu, H.-Y., Wang, C.-H., Yang, C.-H., Ko, J.-T., Ho, H.-C., Tsai, M.-D., **Bryant**,

D. A., Li, F.-W., Ho, M.-C., and Ho, M.-Y. 2023. A structure of the relict phycobilisome from a thylakoid-free cyanobacterium. *Nat. Commun*. **14**, 8009. doi: 10.1038/s41467-023-43646-9

1. Silori, Y., Willow, R., Nguyen, H. H., Shen, G., Song, Y., Gisriel, C. J., Brudvig, G. W., **Bryant**,

D. A., and Ogilvie, J. P. 2023. Two-dimensional electronic spectroscopy of the far-red-light

photosystem II reaction center. *J. Phys. Chem*. *Lett.* **14**, 10300-10308. doi: 10.1021/acs.jpclett.3c02604

1. Gisriel, C. J., **Bryant**, D. A., Brudvig, G. W., and Cardona, T. 2023. Molecular diversity and evolution of far-red light-acclimated photosystem I. *Front*. *Plant Sci.* **14**, 1289199. doi: 10.3389/fpls.2023.1289199
2. Schluchter, R. M., Babin, C. H., Liu, X., Bieller, A., Shen, G., Alvey, R. M., and **Bryant**, D. A. 2023. Loss of biliverdin reductase increases oxidative stress in cyanobacterium *Synechococcus* sp. PCC 7002*. Microorganisms* **11**, 2593. doi: 10.3390/microorganisms11102593
3. Gisriel, C. J., Shen, G., Brudvig, G. W., **Bryant**, D. A. 2023. Structures of the far-red-light allophycocyanin bicylindrical core and RuBisCO from the FaRLiP cyanobacterium *Synechococcus* sp. PCC 7335. *J. Biol. Chem.*, **300**, 105590. “Editor’s Pick” doi: 10.1016/j.jbc.2023.105590
4. Cherepanov, D. A., Kurashov, V., Gostev, F. E., Shelaev, I. V., Zabelin, A. A., Shen, G., Mamedov, M. D., Aybush, A., Shkuropatov, A. Ya., Nadtochenko, V. A., **Bryant**, D. A., Golbeck, J. H., Semenov, A. Y. 2024. Femtosecond optical studies of the primary charge separation reactions in far-red photosystem II from *Synechococcus sp.* PCC 7335. *Biochim. Biophys. Acta Bioenerg.*, **1865** (3), 149044. [doi.org/10.1016/j.bbabio.2024.149044](https://doi.org/10.1016/j.bbabio.2024.149044)
5. **Bryant**, D. A., and Gisriel, C. J. 2024. The structural basis for light harvesting in organisms producing phycobiliproteins. *The Plant Cell*, **36**, 4036–4064. [doi.org/10.1093/plcell/koae126](https://doi.org/10.1093/plcell/koae126)
6. Ulrich, N. J., Shen, G., **Bryant**, D. A., and Miller, S. R. 2024. Ecological diversification of a cyanobacterium through divergence of its novel chlorophyll d-based light-harvesting system. *Current Biology*. **34**, 2972-2979. doi: [10.1016/j.cub.2024.05.022](https://doi.org/10.1016/j.cub.2024.05.022)
7. Dsouza, L., Li, X., Eríc V., Frehan, S. K., Huijser, A., Jansen, T. L. C., Halzwarth, A. R., Buda,

F., **Bryant**, D. A., Bahri, S., Gupta, K. G. S. S., Sevink, G. J. A., de Groot, H. J. M. 2024. An integrate approach towards extracting structural characteristic of chlorosomes in a *bchQ* mutant of *Chlorobaculum tepidum*. *Phys. Chem. Chem. Phys.*, **26**, 15856-15867. doi: [10.1039/D4CP00221K](https://doi.org/10.1039/D4CP00221K)

1. Olmsted, C. N, Gahler, M, Peterson, B., Roden, E. Lazarcik, J., Schramm, P., Tran, P. Q., Berg, M., **Bryant**, D. A. Goudeau, D., Malmstrom, R. R., Roux, S., Thweatt, J., Eloe-Fadrosh, E. A., Yitbarek, S., Qin, M., and McMahon, K. D. 2024. Cryptic cycling by electroactive bacterioplankton in Trout Bog Lake. *Environ. Microbiol*. 2024-7. doi: 10.1101/2024.07.11.603116
2. Dsouza, L., Gupta, K. B. S. S., Li, X., Erić, V., Luo, Y., Huijser, A., Jansen, T. L., Buda, F., Holzwarth, A. R., **Bryant**, D. A. and Gurinov, A. 2025. Conformational Dynamics of Bacteriochlorophyll c in Chlorosomes from the *bchQ* Mutant of *Chlorobaculum tepidum*. *The Journal of Physical Chemistry B*. **129**(8), 2129-2137. doi: 10.1021/acs.jpcb.4c04731

## Manuscripts in preparation

1. Saini, M. K., Thiel, V., Tank, M., and **Bryant**, D. A. 2024. *Chloracidobacterium validum*, spp. nov., a thermophilic bacterium of the phylum *Acidobacteriota* from alkaline hot spring mats, amended description of the genus *Chloracidobacterium,* and a proposal to create a new order, *Chloracidobacteriales* (ord. nov.) and a new family, *Chloracidobacteriaceae* (fam. nov.). *Int. J. Syst. Evol. Microbiol*. Manuscript in preparation.
2. Ho, M.-Y. Shen, G., and **Bryant**, D. A. 2024. Kinetics of cellular responses to chromatic illumination provide insights into light-niche adaptation in the cyanobacterium *Synechococcus* sp. PCC 7335. Manuscript in preparation.
3. Liu, Z., Li, T., Shen, G., Marquardt, J., Nomura, C. T., Ludwig, M., Gugger, M., Drautz-Moses,

D. I., Schuster, S. C., Zhao, J., and **Bryant**, D. A. 2024. Genome sequences for five strains of *Synechococcus* sp.: strains PCC 7002, 7003, 73109, 7117, and 8807. *Front. Microbiol.,* manuscript in preparation.
